# Species conservation profiles of the endemic spiders *Troglohyphantes* (Araneae, Linyphiidae) from the Alps and the north-western Dinarides

**DOI:** 10.3897/BDJ.10.e87261

**Published:** 2022-08-19

**Authors:** Filippo Milano, Luca Borio, Christian Komposch, Stefano Mammola, Paolo Pantini, Martina Pavlek, Marco Isaia

**Affiliations:** 1 Department of Life Sciences and Systems Biology, University of Turin, Turin, Italy Department of Life Sciences and Systems Biology, University of Turin Turin Italy; 2 ÖKOTEAM – Institute for Animal Ecology and Landscape Planning, Graz, Austria ÖKOTEAM – Institute for Animal Ecology and Landscape Planning Graz Austria; 3 Water Research Institute (IRSA), National Research Council (CNR), Verbania Pallanza, Italy Water Research Institute (IRSA), National Research Council (CNR) Verbania Pallanza Italy; 4 Finnish Museum of Natural History, University of Helsinki, Helsinki, Finland Finnish Museum of Natural History, University of Helsinki Helsinki Finland; 5 Museo Civico di Scienze Naturali “E. Caffi.”, Bergamo, Italy Museo Civico di Scienze Naturali “E. Caffi.” Bergamo Italy; 6 Ruđer Bošković Institute, Zagreb, Croatia Ruđer Bošković Institute Zagreb Croatia; 7 Croatian Biospeleological Society, Zagreb, Croatia Croatian Biospeleological Society Zagreb Croatia

**Keywords:** climate change, IUCN, cave, Red List, subterranean species, Linyphiidae, troglobiont, karst, spiders

## Abstract

**Background:**

The genus *Troglohyphantes* Joseph, 1882 (Araneae, Linyphiidae) includes 131 species, mainly distributed across the main European mountain ranges. The Alps and the north-western Dinarides account for 66 species, most of them showing narrow or even point-like distributions. The majority of *Troglohyphantes* spiders dwell in subterranean habitats including caves, mines, soil litter, rocky debris and other moist and shaded retreats. Despite being intensively studied from taxonomic, ecological and biogeographic standpoints, knowledge on the status of conservation and on the potential risk of extinction of these spiders is lagging. To date, only three species have been included in the global IUCN Red List, but their status has not been updated ever since their last assessment in 1996. The aim of this contribution is to assess the Alpine and north-western Dinaric species of the genus *Troglohyphantes* and to re-assess the species previously evaluated, according to the last version of the IUCN Red List Categories and Criteria.

**New information:**

Amongst the 66 species here considered, 62 had sufficient data to allow the quantification of their Extent Of Occurrence (EOO) and Area Of Occupancy (AOO). Most of the species have a narrow distribution range, with an estimated EOO < 20,000 km^2^ and AOO < 2,000 km^2^, meeting the thresholds for the inclusion in the threatened categories. Five species have a more widespread distribution (EOO > 20,000 km^2^), extending across multiple countries. The quality of the data on distribution of four species was not sufficient to provide a reliable estimation of the distribution range.

A continuing decline in EOO, AOO and habitat quality was inferred for 30 species. The majority of them were subterranean specialised species, with a reduced thermal tolerance and a low dispersal ability. Accordingly, changes in subterranean microclimatic conditions due to climate change represent a major threat for these species. Land-use change and habitat alteration were identified as additional relevant threats for several species.

A considerable proportion of the species here assessed was found in protected areas and in sites of the Natura 2000 network. In addition, 14 species are formally protected by national and sub-national legislation. At present, 25 species are listed in the regional Red Lists.

Long-term monitoring programmes, management plans for both the species and their habitats, expansion of the extant protected areas and designation of new ones, should be considered as the most effective approaches to species conservation.

## Introduction

The Alps and the Dinarides are known for their wide variety of habitats and they have been recognised as one of the major biodiversity hotspots in Europe (Nagy et al. 2003, Condé and Richard 2005). The combination of topographical, geomorphological and long-term climatic factors allowed the development of a very diverse and unique fauna, including spiders (Nentwig et al. 2022). Spiders (Arachnida, Araneae) are a mega-diverse group of arthropods comprising more than 50,000 currently described species (World Spider Catalog 2022). They are considered one of the most successful groups of organisms in terms of abundance, evolutionary radiation, biomass, functional roles and ecological plasticity (Turnbull 1973, Coddington and Levi 1991, Cardoso et al. 2008, Coddington et al. 2009, Foelix 2011, Jocqué et al. 2013, Nentwig 2013, Garrison et al. 2016, Mammola et al. 2017a, Dunlop et al. 2018) and they have colonised all terrestrial habitats, including subterranean ones (Mammola et al. 2018a).

Spiders have undergone a remarkable diversification in subterranean habitats (Mammola and Isaia 2017, Mammola et al. 2018a, Mammola et al. 2019b), including deep soil strata in forest, shallow voids in mountain screes and deep caves (Mammola and Isaia 2017); therein, they play an important functional role as apical predators in the subterranean food webs (Parimuchová et al. 2021).

The genus *Troglohyphantes* (Araneae, Linyphiidae) is predominantly distributed in the main European mountain ranges, including Pyrenees, Alps, Dinarides and Carpathians (Deeleman-Reinhold 1978, Pesarini 2001, Deltshev 2008, Isaia et al. 2011, Isaia et al. 2017, Mammola et al. 2018b). Currently, 131 species are described (Nentwig et al. 2022), most of them occurring in Europe. Their distribution is often restricted to very narrow areas, sometimes to just one or a few localities (Deeleman-Reinhold 1978, Isaia et al. 2011, Isaia et al. 2017, Mammola et al. 2018a).

Species of the genus *Troglohyphantes* generally show a remarkable preference for subterranean habitats (sensu Culver and Pipan 2019). They mainly occur in cold, wet and dark habitats, such as caves, bunkers, mines, but also in Shallow Subterranean Habitats (SSH, sensu Culver and Pipan 2019), including soil and leaf litter, rocky debris and other moist and shaded retreats (Fage 1919, Deeleman-Reinhold 1978, Isaia et al. 2010, Isaia et al. 2011, Isaia et al. 2017, Mammola et al. 2018a).

*Troglohyphantes* spiders display different levels of subterranean habitat specialisation. Species found in both caves and surface habitats are often able to withstand ecological variations, while others are almost exclusively found in caves and are characterised by behavioural, physiological and morphological adaptations to the stringent conditions of the subterranean habitat (Deeleman-Reinhold 1978, Isaia et al. 2017, Mammola et al. 2020, Mammola et al. 2022a). These adaptive traits include reduction or loss of eyes and cuticular pigmentation, thinning of the integument, heavier spination, appendage elongation, reduction in the metabolic rate leading to higher resistance to starvation, alteration of the circadian rhythm, reduction in fecundity, slower development, delayed maturation and extended longevity when compared with their surface relatives (for a review, see Mammola and Isaia 2017). Detailed descriptions and quantification of functional traits of *Troglohyphantes* spiders (hypogean affinity, upper thermal limits, conservation status and legal protection) are available with open access in the World Spider Trait database (Pekár et al. 2021).

As demonstrated by means of ecological niche modelling and physiological experiments (Mammola et al. 2018a, Mammola et al. 2019a), the increased specialisation to subterranean habitats seen in *Troglohyphantes* spiders, resulting from a long evolutionary history in a thermally stable environment, is accompanied by the concomitant narrowing of their thermal tolerance. While most species living close to the surface or in shallow subterranean environments have retained their ability to withstand temperature variations, specialised subterranean species of *Troglohyphantes* have lost such thermoregulatory mechanisms and are, therefore, particularly vulnerable to potential subterranean climatic variation induced by climate change (Mammola et al. 2019b).

Anthropogenic global warming is expected to significantly influence and modify the underground climate (Badino 2004, Domínguez-Villar et al. 2014), ultimately affecting subterranean biocoenosis and ecosystems (Mammola et al. 2019c, Sánchez-Fernández et al. 2021). This pressure is exacerbated by land-use change and habitat alteration, both subterranean and at the surface, which are, in general, regarded as the major threats to spider communities (Borges et al. 2016, Borges et al. 2019, Branco and Cardoso 2020, Milano et al. 2021, Mammola et al. 2022b). In addition, most of the occurrence localities of *Troglohyphantes* are highly isolated from each other, hindering the – already very poor – dispersal ability of these species (Cardoso et al. 2011, Mammola et al. 2015, Mammola and Isaia 2016).

Accordingly, recent analyses focusing on Western Italian Alps pointed towards a future decline in habitat suitability for specialised subterranean *Troglohyphantes* spiders and hypothesised potential high risk of local extinction for the most restricted endemic species (Mammola et al. 2018a). This prediction finds further confirmation in recent studies quantifying upper thermal limits of the western alpine species (Mammola et al. 2019a) showing very low ranges of thermal tolerance, especially for the most adapted species.

All in all, the existing wealth of threats facing subterranean biota (Mammola et al. 2022) and the ecological and biogeographical peculiarities of the *Troglohyphantes* spiders, strengthens the importance of considering these species in international and national conservation measures. Here, we aim to assess the Alpine and the north-western Dinaric species of the genus *Troglohyphantes* according to the International Union for Conservation of Nature (IUCN) Red List Criteria.

## Methods

We compiled a comprehensive dataset with georeferenced records of the 66 species of *Troglohyphantes* occurring in the Alps and the north-western Dinarides, based on scientific literature, grey literature and unpublished records. We performed spatial analyses in R (R Core Team 2021), with the package 'red' – IUCN red-listing tools (Cardoso 2017), using either observed occurrences or estimated ranges. The package includes functions to calculate the Extent of Occurrence (EOO) and the Area of Occupancy (AOO), to map species ranges and to perform species distribution modelling.

In this work, we calculated EOO and AOO as follows:

- for species with low levels of subterranean adaptation (sensu Mammola et al. 2019a, hereinafter "non-specialised species"), with at least 15 records and for which we do not have full confidence about range limits, we modelled the range via species distribution modelling. Due to the lack of reliable absence data for our model species, we constructed a species distribution model using a standard presence-background algorithm (MaxEnt), with the function maxent in the 'dismo' R package (Hijmans et al. 2021). We modelled the distribution of the species using a combination of present-day climatic data and altitude above sea level (Fick and Hijmans 2017) at a spatial resolution of 30 arc-seconds (approximately 1 km at the Equator). As recommended in the literature (Peterson et al. 2011, Saupe et al. 2012, Merow et al. 2013), we calibrated species distribution models within a geographic area that we hypothesised has been accessible to the species. Considering the low dispersal potential of *Troglohyphantes* spiders (Mammola et al. 2018a), we approximated this area by buffering each occurrence by a diameter of 60 km. For a few species for which the density of records was low and scattered across the known distribution range (i.e. when the maximum distance between two neighbouring records exceeds 60 km), we assumed this area by buffering each occurrence record by 100 km. We performed a Principal Components Analysis on the predictor variables to generate new axes that summarised variation in fewer dimensions, thereby avoiding collinearity amongst covariates. To avoid overfitting, we retained the first four principal components. One hundred models were run for each species, using both coordinates and the associated spatial error. For each modelled distribution, we calculated EOO and AOO using specific functions in 'red' and reported their lower and upper confidence limits and the consensus values, calculated as all the cells predicted to be suitable for the species in at least 97.5%, 2.5% and 50% of the runs, respectively. These values are respectively reported as three consequent numbers in the relative sections "Extent of occurrence" and "Area of occupancy".

- for high and intermediate subterranean specialised species (sensu Mammola et al. 2019a, hereinafter "subterranean specialised") with narrow distribution ranges, we classified EOO and AOO as "observed" (in this respect see Mammola and Leroy 2017). For non-specialised species with less than 15 records, due to the unreliability of the distribution predicted by models with limited known occurrences, we based EOO and AOO on the “observed” distribution, with comments on the relative uncertainty about range limits. Particular cases are treated individually in each species profile. We used the minimum convex polygon encompassing all observations to calculate EOO and the 2 x 2 km cells known to be occupied to calculate AOO. When EOO was smaller than AOO, it was made equal as per the IUCN guidelines (IUCN Standards and Petitions Committee 2019);

We checked and validated the final maps and related values with our own expert opinion. We also produced KMLs maps using the 'red' function "kml".

We calculated the generation length of the species on the basis of the ecological information available in literature. Deeleman-Reinhold (1978) observed that subadults of *Troglohyphantes* species may moult up to 14 months after their collection. However, some non-specialised species passed through two moults in 5 months. Accordingly, we estimated a minimum generation length of two years for non-specialised species and four years for subterranean specialised species.

The nomenclature used in this work refers to the latest version of the World Spider Catalog (2022). All the species traits are sourced from the World Spider Trait database (Pekár et al. 2021). The toponomastics and classification of the different sectors and sub-sectors of the Alps follows the partition of the Alpine chain (SOIUSA, Marazzi 2005).

## Species Conservation Profiles

### Troglohyphantes achillis

#### Species information

Scientific name: Troglohyphantesachillis

Species authority: Isaia & Mammola, 2022

Kingdom: Animalia

Phylum: Arthropoda

Class: Arachnida

Order: Araneae

Family: Linyphiidae

Taxonomic notes: In Mammola et al. (2018a) and Mammola and Isaia (2016), this species was treated as *T.vignai* (partim). Being a subterranean specialised species, results of ecological niche modelling used to project future habitat suitability in Mammola et al. (2018a) are equally applied to this species.

**Figure 1.**
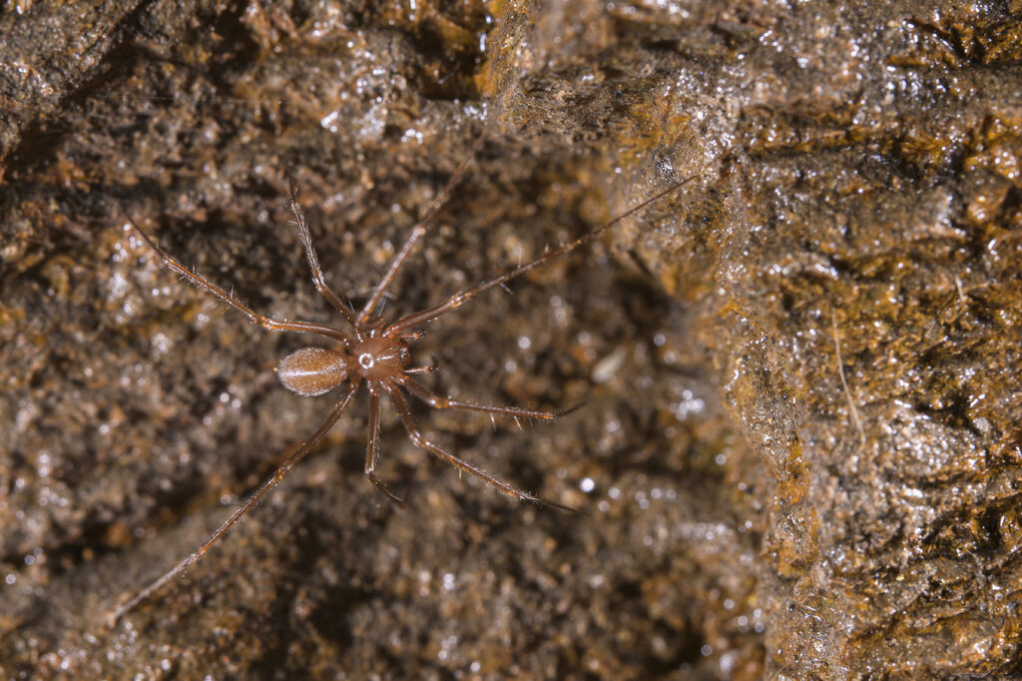
*Troglohyphantesachillis* Isaia & Mammola, 2022. Photo credit: Emanuele Biggi.

Figure(s) or Photo(s): Fig. 1

Region for assessment: Global

#### Editor & Reviewers

##### Reviewers

Reviewers: Marc MilnePaulo Borges

##### Editor

Editor: Pedro Cardoso

#### Geographic range

Biogeographic realm: Palearctic

Countries: Italy

Map of records (Google Earth): Suppl. material 1

Basis of EOO and AOO: Observed

Basis (narrative): Caves in Western Alps have been extensively sampled, allowing us to define EOO and AOO of this species with reasonable confidence.

Min Elevation/Depth (m): 990

Max Elevation/Depth (m): 1414

Range description: This species is known from a few localities of the Chisone and Germanasca Valleys, in the Northern Cottian Alps, (north-western Italy) (detailed occurrences and relative references in Suppl. material 67).

#### Extent of occurrence

EOO (km2): 57

Trend: Decline (inferred)

Justification for trend: As seen in Mammola et al. (2018a) for other subterranean specialised *Troglohyphantes* species of the Western Alps, climate change is expected to affect the distribution of this species in the future. Given the reduced thermal tolerance of this organism and its low dispersal ability (Mammola et al. 2019c, Isaia et al. 2022), a reduction of its geographic distribution range is expected in the future.

Causes ceased?: No

Causes understood?: Yes

Causes reversible?: No

Extreme fluctuations?: No

#### Area of occupancy

Trend: Decline (inferred)

Justification for trend: As seen in Mammola et al. (2018a) for other subterranean specialised *Troglohyphantes* species of the Western Alps, climate change is expected to affect the distribution of this species in the future. Given the reduced thermal tolerance of this organism and its low dispersal ability (Mammola et al. 2019c, Isaia et al. 2022), a reduction of its geographic distribution range is expected in the future.

Causes ceased?: No

Causes understood?: Yes

Causes reversible?: No

Extreme fluctuations?: No

AOO (km2): 16

#### Locations

Number of locations: 1

Justification for number of locations: The habitat where this species occurs is affected by changes in subterranean microclimatic conditions due to climate change, which is expected to impact the whole population (Mammola et al. 2018a).

Trend: Stable

Extreme fluctuations?: No

#### Population

Number of individuals: Unknown

Trend: Decline (inferred)

Justification for trend: In view of the reduced thermal tolerance of this species (Isaia et al. 2022), alterations of the microclimatic conditions of the habitat due to climate change are expected to impact the whole population of this species.

Basis for decline: (c) a decline in area of occupancy, extent of occurrence and/or quality of habitat

Causes ceased?: No

Causes understood?: Yes

Causes reversible?: No

Extreme fluctuations?: No

#### Subpopulations

Number of subpopulations: 4

Trend: Decline (inferred)

Justification for trend: Due to the subterranean adaptation and the narrow physiological tolerance of this species, hampering dispersal through non-subterranean habitats (see Mammola et al. 2015 about high genetic structuring in this species), each locality reasonably hosts a single highly isolated subpopulation. Accordingly, we identified four subpopulations, all likely to be impacted by climate change.

Extreme fluctuations?: No

Severe fragmentation?: No

#### Habitat

System: Terrestrial

Habitat specialist: Yes

Habitat (narrative): This species has been collected among the floor debris and on the walls of the twilight zone, in natural caves and mine prospects located at the medium alpine montane belt, from 1,000 up to 1,400 m above sea level, characterised by mean annual temperature values ranging from 6.1 to 8.9°C (Isaia et al. 2022). See Mammola and Isaia (2016) for a characterisation of the ecological niche of the species and additional information on its preferred habitat.

Trend in extent, area or quality?: Decline (inferred)

Justification for trend: As seen in Mammola et al. (2018a) for other species of *Troglohyphantes* of the Western Alps, a drastic decline in the habitat suitability of *T.achillis* as a consequence of climate change is expected.

##### Habitat

Habitat importance: Major Importance

Habitats: 7. Caves and Subterranean Habitats (non-aquatic)

#### Ecology

Size: 3 mm

Generation length (yr): 4

Dependency of single sp?: No

Ecology and traits (narrative): According to thermal tolerance tests, *T.achillis* shows intermediate thermal tolerance, reaching 50% mortality at temperature values 5°C above its cave temperature (Isaia et al. 2022). Females are present all year round, while males are found in small numbers in spring or early autumn and almost disappear in winter; annual sex ratio estimated on a population in a mine in the Chisone valley is 2.4:1 in favour of females (Mammola and Isaia 2016). See Mammola and Isaia (2016) and Isaia et al. (2022) for additional information on the ecological preferences of this species.

#### Threats

Justification for threats: This species is potentially exposed due to its extremely narrow geographic distribution range. As seen for other species of the genus *Troglohyphantes* of the Western Alps (Mammola et al. 2018a), climate warming is expected to reduce the currently suitable habitat for this spider. Moreover, in view of its reduced thermal tolerance (Isaia et al. 2022), this species has a limited dispersal ability, which represents an additional concern in face of the ongoing increase of temperature.

##### Threats

Threat type: Future

Threats: 11.1. Climate change & severe weather - Habitat shifting & alteration11.2. Climate change & severe weather - Droughts11.3. Climate change & severe weather - Temperature extremes

#### Conservation

Justification for conservation actions: One of the records of this species is located within the Special Area of Conservation and Special Protection Area of the Orsiera Rocciavré (SAC/SPA IT1110006).

##### Conservation actions

Conservation action type: In Place

Conservation actions: 1.1. Land/water protection - Site/area protection1.2. Land/water protection - Resource & habitat protection

#### Other

##### Use and trade

Use type: International

##### Ecosystem services

Ecosystem service type: Important

##### Research needed

Research needed: 1.2. Research - Population size, distribution & trends1.3. Research - Life history & ecology1.5. Research - Threats

Justification for research needed: Research on basic information such as distribution, ecology, life cycle and possible threats throughout the range are needed.

### Troglohyphantes albopictus

#### Species information

Scientific name: Troglohyphantesalbopictus

Species authority: Pesarini, 1989

Kingdom: Animalia

Phylum: Arthropoda

Class: Arachnida

Order: Araneae

Family: Linyphiidae

Region for assessment: Global

#### Editor & Reviewers

##### Reviewers

Reviewers: Marc MilnePaulo Borges

##### Editor

Editor: Pedro Cardoso

#### Geographic range

Biogeographic realm: Palearctic

Countries: Italy

Map of records (Google Earth): Suppl. material 2

Basis of EOO and AOO: Observed

Basis (narrative): This spider was collected in very few localities. Its low level of subterranean specialisation, together with the observed high altimetric range of its known distribution, possibly reflects a higher dispersal capacity when compared to subterranean specialised species. Consequently, it may be possible that the present known range of this species is underestimated.

Min Elevation/Depth (m): 22

Max Elevation/Depth (m): 1661

Range description: *Troglohyphantesalbopictus* is restricted to the Colli Euganei, the Colli Berici and the Prealps of Veneto (north-western Italy) (detailed occurrences and relative references in Suppl. material 67).

#### Extent of occurrence

EOO (km2): 2306

Trend: Stable

Justification for trend: This species is not strictly relegated to deep subterranean habitats, being collected both in epigean and shallow subterranean habitats. It is plausible that anthropogenic climate change may affect the habitat suitability of this species. However, in view of the relatively wide thermal tolerance and the relatively high dispersal ability of non-specialised *Troglohyphantes* species (Mammola et al. 2019a), the distribution range of *T.albopictus* is not expected to undergo significant reduction in the near future. A deeper study on the current distribution of this species and on the potential impacts of climate change is required.

Causes ceased?: Yes

Causes understood?: Yes

Causes reversible?: Yes

Extreme fluctuations?: No

#### Area of occupancy

Trend: Stable

Justification for trend: This species is not strictly relegated to deep subterranean habitats, being collected both in epigean and shallow subterranean habitats. It is plausible that anthropogenic climate change may affect the habitat suitability of this species. However, in view of the relatively wide thermal tolerance and the relatively high dispersal ability of non-specialised *Troglohyphantes* species (Mammola et al. 2019a), the distribution range of *T.albopictus* is not expected to undergo significant reduction in the near future. A deeper study on the current distribution of this species and on the potential impacts of climate change is required.

Causes ceased?: Yes

Causes understood?: Yes

Causes reversible?: Yes

Extreme fluctuations?: No

AOO (km2): 44

#### Locations

Number of locations: Not applicable

Justification for number of locations: No known threats to this species.

Trend: Stable

Extreme fluctuations?: No

#### Population

Number of individuals: Unknown

Trend: Stable

Justification for trend: There are no currently known threats to the species.

Causes ceased?: Yes

Causes understood?: Yes

Causes reversible?: Yes

Extreme fluctuations?: No

#### Subpopulations

Number of subpopulations: Unknown

Trend: Unknown

Extreme fluctuations?: No

Severe fragmentation?: No

#### Habitat

System: Terrestrial

Habitat specialist: Yes

Habitat (narrative): The species was collected both in epigean and shallow subterranean habitats. The record from Colli Berici was collected in a damp wood, in the vicinity of a small stream. No additional information about the habitat was provided.

Trend in extent, area or quality?: Stable

##### Habitat

Habitat importance: Major Importance

Habitats: 1.4. Forest - Temperate7.2. Caves and Subterranean Habitats (non-aquatic) - Other Subterranean Habitats

#### Ecology

Size: 3.9 mm

Generation length (yr): 2

Dependency of single sp?: No

Ecology and traits (narrative): Not much is known about the ecology and life history of *T.albopictus*. This spider shows minor specialisation to subterranean life (Mammola et al. 2022a).

#### Threats

Justification for threats: The existence of threats is unknown for this species.

##### Threats

Threat type: Past

Threats: 12. Other options - Other threat

#### Conservation

Justification for conservation actions: *Troglohyphantesalbopictus* has been recorded within several protected areas (EUAP0243 Parco Regionale dei Colli Euganei and SAC/SPA IT3260017 Colli Euganei - Monte Lozzo - Monte Ricco, SAC/SPA IT3210040 Monti Lessini - Pasubio - Piccole Dolomiti Vicentine, SAC/SPA IT3230022 Massiccio del Grappa).

##### Conservation actions

Conservation action type: In Place

Conservation actions: 1.1. Land/water protection - Site/area protection1.2. Land/water protection - Resource & habitat protection

#### Other

##### Use and trade

Use type: International

##### Ecosystem services

Ecosystem service type: Important

##### Research needed

Research needed: 1.2. Research - Population size, distribution & trends1.3. Research - Life history & ecology1.5. Research - Threats

Justification for research needed: Research on basic information such as distribution, natural history, ecology and possible threats of the species would be needed.

### Troglohyphantes apenninicus

#### Species information

Scientific name: Troglohyphantesapenninicus

Species authority: Isaia, Mammola & Pantini, 2017

Kingdom: Animalia

Phylum: Arthropoda

Class: Arachnida

Order: Araneae

Family: Linyphiidae

Region for assessment: Global

#### Editor & Reviewers

##### Reviewers

Reviewers: Marc MilnePaulo Borges

##### Editor

Editor: Pedro Cardoso

#### Geographic range

Biogeographic realm: Palearctic

Countries: Italy

Map of records (Google Earth): Suppl. material 3

Basis of EOO and AOO: Observed

Basis (narrative): This species was collected in two localities. Its low level of subterranean specialisation, possibly reflects a higher dispersal capacity when compared to subterranean specialised species. Given this situation, any modelling of the current habitat suitability is unreliable and the known distribution range should be taken with caution. Further research is needed on this species in order to assess its extinction risk.

Min Elevation/Depth (m): 1383

Max Elevation/Depth (m): 1401

Range description: The species is known only from two localities of the Tuscan Apennines: Abetone (Pistoiese Mountains) and Mount Sumbra (Apuan Alps) (detailed occurrences and relative references in Suppl. material 67). Remark: fig. 3 in Isaia et al. (2017):311 mistakenly reports three localities instead of two. However, localities are correctly reported in the Material (p. 312).

#### Extent of occurrence

EOO (km2): 8

Trend: Unknown

Causes ceased?: Unknown

Causes understood?: Unknown

Causes reversible?: Unknown

Extreme fluctuations?: No

#### Area of occupancy

Trend: Unknown

Causes ceased?: Unknown

Causes understood?: Unknown

Causes reversible?: Unknown

Extreme fluctuations?: No

AOO (km2): 8

#### Locations

Number of locations: Unknown

Justification for number of locations: The data available are not enough to estimate the number of locations for this species.

Trend: Unknown

Extreme fluctuations?: No

#### Population

Number of individuals: Unknown

Trend: Unknown

Justification for trend: The population size and trend are unknown.

Causes ceased?: Unknown

Causes understood?: Unknown

Causes reversible?: Unknown

Extreme fluctuations?: No

#### Subpopulations

Number of subpopulations: Unknown

Trend: Unknown

Extreme fluctuations?: No

Severe fragmentation?: No

#### Habitat

System: Terrestrial

Habitat specialist: No

Habitat (narrative): The species was collected in epigean localities. No additional information about the habitat was provided.

Trend in extent, area or quality?: Stable

##### Habitat

Habitat importance: Major Importance

Habitats: 18. Unknown

#### Ecology

Size: 3.1 mm

Generation length (yr): 2

Dependency of single sp?: No

Ecology and traits (narrative): Not much is known about the ecology and life history of this species. This spider shows minor specialisation to subterranean life (Mammola et al. 2022a).

#### Threats

Justification for threats: Unknown threats.

##### Threats

Threat type: Past

Threats: 12. Other options - Other threat

#### Conservation

Justification for conservation actions: One of the known records of this species is inside protected areas (SAC IT5120009 Monte Sumbra, SPA IT5120015 Praterie primarie e secondarie delle Apuane).

##### Conservation actions

Conservation action type: In Place

Conservation actions: 1.1. Land/water protection - Site/area protection1.2. Land/water protection - Resource & habitat protection2.1. Land/water management - Site/area management

#### Other

##### Use and trade

Use type: International

##### Ecosystem services

Ecosystem service type: Important

##### Research needed

Research needed: 1.2. Research - Population size, distribution & trends1.3. Research - Life history & ecology1.5. Research - Threats

Justification for research needed: Research on basic information such as distribution, natural history, ecology and possible threats of the species would be needed.

### Troglohyphantes bolognai

#### Species information

Scientific name: Troglohyphantesbolognai

Species authority: Brignoli, 1975

Kingdom: Animalia

Phylum: Arthropoda

Class: Arachnida

Order: Araneae

Family: Linyphiidae

Region for assessment: Global

#### Editor & Reviewers

##### Reviewers

Reviewers: Marc MilnePaulo Borges

##### Editor

Editor: Pedro Cardoso

#### Geographic range

Biogeographic realm: Palearctic

Countries: Italy

Map of records (Google Earth): Suppl. material 4

Basis of EOO and AOO: Observed

Basis (narrative): There are only two records known for this spider. In light of its high subterranean specialisation and in view of the intensive sampling conducted in the area in recent years, it is hardly expected that the range could be significantly expanded by new findings.

Min Elevation/Depth (m): 728

Max Elevation/Depth (m): 810

Range description: This species is known from two caves (Sgarbu du Ventu and Tana Bertrand) in the Province of Imperia (Liguria, north-western Italy) (detailed occurrences and relative references in Suppl. material 67).

#### Extent of occurrence

EOO (km2): 8

Trend: Decline (inferred)

Justification for trend: According to Mammola et al. (2018a), climate change will significantly affect the distribution of subterranean specialised *Troglohyphantes* in the future. Moreover, given the low tolerance to habitat changes of these species (see Mammola et al. 2018a) as well as their very low dispersal ability, a possible extreme reduction of the geographic range is expected in the future.

Causes ceased?: No

Causes understood?: Yes

Causes reversible?: No

Extreme fluctuations?: No

#### Area of occupancy

Trend: Decline (inferred)

Justification for trend: According to Mammola et al. (2018a), climate change will significantly affect the distribution of subterranean specialised *Troglohyphantes* in the future. Moreover, given the low tolerance to habitat changes of these species (see Mammola et al. 2018a) as well as their very low dispersal ability, a possible extreme reduction of the geographic range is expected in the future.

Causes ceased?: No

Causes understood?: Yes

Causes reversible?: No

Extreme fluctuations?: No

AOO (km2): 8

#### Locations

Number of locations: 1

Justification for number of locations: This species has been recorded in two caves, which are interpreted as a single location as they are both affected by changes in subterranean microclimatic conditions due to climate change.

Trend: Stable

Extreme fluctuations?: No

#### Population

Number of individuals: Unknown

Trend: Decline (inferred)

Justification for trend: In view of the reduced thermal tolerance of subterranean specialised *Troglohyphantes* species (Mammola et al. 2019a), alterations of the microclimatic conditions of the habitat due to climate change are expected to impact the whole population of this species.

Basis for decline: (c) a decline in area of occupancy, extent of occurrence and/or quality of habitat

Causes ceased?: No

Causes understood?: Yes

Causes reversible?: No

Extreme fluctuations?: No

#### Subpopulations

Number of subpopulations: 2

Trend: Decline (inferred)

Justification for trend: Due tothe adaptation to the subterranean medium and of the narrow physiological tolerance of this species, likely hampering dispersal through non-subterranean habitats, each cave reasonably hosts a single isolated subpopulation. Accordingly, for this species we identified two subpopulations, occurring in two different caves in the province of Imperia, Liguria. Both subpopulations are likely to be impacted by climate change.

Extreme fluctuations?: No

Severe fragmentation?: No

#### Habitat

System: Terrestrial

Habitat specialist: Yes

Habitat (narrative): *Troglohyphantesbolognai* was collected in deep hypogean habitats. No additional information on the habitat was provided.

Trend in extent, area or quality?: Decline (inferred)

Justification for trend: As seen in Mammola et al. (2018a) for other species of the genus *Troglohyphantes* of the Western Alps, a drastic decline in the habitat suitability of *T.bolognai* as a consequence of climate change is expected.

##### Habitat

Habitat importance: Major Importance

Habitats: 7. Caves and Subterranean Habitats (non-aquatic)

#### Ecology

Size: 2.8 mm

Generation length (yr): 4

Dependency of single sp?: No

Ecology and traits (narrative): Specimens show pronounced eye regression and absence of pigmentation (Mammola et al. 2022a).

#### Threats

Justification for threats: This species is potentially exposed due to its extremely narrow geographic distribution range, its low thermal tolerance and its low dispersal capacity. As seen for other species of the genus *Troglohyphantes* of the Western Alps (Mammola et al. 2018a), climate warming is expected to reduce the current suitable habitat for this spider.

##### Threats

Threat type: Future

Threats: 11.1. Climate change & severe weather - Habitat shifting & alteration11.2. Climate change & severe weather - Droughts11.3. Climate change & severe weather - Temperature extremes

#### Conservation

Justification for conservation actions: Part of the distribution of *T.bolognai* is included in a Special Area of Conservation (SAC IT1314723 Campasso – Grotta Sgarbu Du Ventu).

##### Conservation actions

Conservation action type: In Place

Conservation actions: 1.1. Land/water protection - Site/area protection1.2. Land/water protection - Resource & habitat protection

#### Other

##### Use and trade

Use type: International

##### Ecosystem services

Ecosystem service type: Important

##### Research needed

Research needed: 1.2. Research - Population size, distribution & trends1.3. Research - Life history & ecology1.5. Research - Threats

Justification for research needed: Research on basic information such as distribution, ecology, life cycle and possible threats throughout the range are needed.

### Troglohyphantes bornensis

#### Species information

Scientific name: Troglohyphantesbornensis

Species authority: Isaia & Pantini, 2008

Kingdom: Animalia

Phylum: Arthropoda

Class: Arachnida

Order: Araneae

Family: Linyphiidae

Region for assessment: Global

#### Editor & Reviewers

##### Reviewers

Reviewers: Marc MilnePaulo Borges

##### Editor

Editor: Pedro Cardoso

#### Geographic range

Biogeographic realm: Palearctic

Countries: Italy

Map of records (Google Earth): Suppl. material 5

Basis of EOO and AOO: Observed

Basis (narrative): Caves in Western Italian Alps have been extensively sampled, allowing us to define EOO and AOO of this species with reasonable confidence.

Min Elevation/Depth (m): 765

Max Elevation/Depth (m): 885

Range description: This species is restricted to four caves in the Pugnetto area, a calcschist cave complex in the province of Torino (Piemonte, north-western Italy) (detailed occurrences and relative references in Suppl. material 67).

#### Extent of occurrence

EOO (km2): 4

Trend: Decline (inferred)

Justification for trend: As seen in Mammola et al. (2018a) for all the subterranean specialised *Troglohyphantes* species of the Western Alps, climate change is expected to affect the distribution of this species in the future. Given the reduced thermal tolerance and the low dispersal ability (Mammola et al. 2019a) of this species, an extreme reduction of its geographic distribution range is expected in the future.

Causes ceased?: No

Causes understood?: Yes

Causes reversible?: No

Extreme fluctuations?: No

#### Area of occupancy

Trend: Decline (inferred)

Justification for trend: As seen in Mammola et al. (2018a) for all the subterranean specialised *Troglohyphantes* species of the Western Alps, climate change is expected to affect the distribution of this species in the future. Given the reduced thermal tolerance and the low dispersal ability (Mammola et al. 2019a) of this species, an extreme reduction of its geographic distribution range is expected in the future.

Causes ceased?: No

Causes understood?: Yes

Causes reversible?: No

Extreme fluctuations?: No

AOO (km2): 4

#### Locations

Number of locations: 1

Justification for number of locations: The habitat where this species occurs is affected by changes in subterranean microclimatic conditions due to climate change, which is expected to impact the whole population (see Mammola et al. 2018a).

Trend: Stable

Extreme fluctuations?: No

#### Population

Number of individuals: Unknown

Trend: Decline (inferred)

Justification for trend: In view of the reduced thermal tolerance of this species (Mammola et al. 2019a), alterations of the microclimatic conditions of the habitat due to climate change are expected to impact the whole population of this species.

Basis for decline: (c) a decline in area of occupancy, extent of occurrence and/or quality of habitat

Causes ceased?: No

Causes understood?: Yes

Causes reversible?: No

Extreme fluctuations?: No

#### Subpopulations

Number of subpopulations: 1

Trend: Stable

Justification for trend: Examining the known range of distribution of this species and taking into account habitat connectivity amongst caves in the hypogean complex of Pugnetto, it is possible to identify a single subpopulation occurring in the caves of the Pugnetto hypogean complex, in the Lanzo Valleys (Graian Alps).

Extreme fluctuations?: No

Severe fragmentation?: No

#### Habitat

System: Terrestrial

Habitat specialist: Yes

Habitat (narrative): Specimens have been found among stony debris in the deep cave habitat. The hypogean complex of Pugnetto consists of an isolated complex of natural calcschist caves in the Western Alps, at an elevation of approximately 800 m. All caves have openings into beech woods, with a prevalent northerly aspect to the cave opening. The entrance of the main cave, Borna Maggiore di Pugnetto, is gated to restrict visitors access.

Trend in extent, area or quality?: Decline (inferred)

Justification for trend: As seen in Mammola et al. (2018a) for other western alpine species of the genus *Troglohyphantes*, a drastic decline in the habitat suitability of *T.bornensis* as a consequence of climate change is expected.

##### Habitat

Habitat importance: Major Importance

Habitats: 7. Caves and Subterranean Habitats (non-aquatic)

#### Ecology

Size: 3 mm

Generation length (yr): 4

Dependency of single sp?: No

Ecology and traits (narrative): This spider shows morphological specialisation to the subterranean habitat, with depigmentation, appendage elongation, flattening of the cephalothorax and moderate eye regression (Mammola et al. 2022a). According to thermal tests, *T.bornensis* shows a narrow thermal tolerance, reaching 50% mortality at temperature values of approximatively 1°C above its cave temperature (Mammola et al. 2019a).

#### Threats

Justification for threats: This species is potentially exposed due to its extremely narrow geographic distribution range. As seen for other *Troglohyphantes* species of the Western Alps (Mammola et al. 2018a), climate warming is expected to reduce the currently suitable habitat for this spider. Moreover, in view of its reduced thermal tolerance (Mammola et al. 2019a), this species has a limited dispersal ability, which represents an additional concern in face of the ongoing increase of temperature.

##### Threats

Threat type: Future

Threats: 11. Climate change & severe weather11.1. Climate change & severe weather - Habitat shifting & alteration11.2. Climate change & severe weather - Droughts11.3. Climate change & severe weather - Temperature extremes

#### Conservation

Justification for conservation actions: The hypogean complex where the species occurs is protected (SAC IT1110048 Grotte del Pugnetto). Entrance to the caves is regulated by the protected area authority and is accessible by permission only. Opening to guided tours is restricted to 8 months a year, closed during winter.

##### Conservation actions

Conservation action type: In Place

Conservation actions: 1.1. Land/water protection - Site/area protection1.2. Land/water protection - Resource & habitat protection2.1. Land/water management - Site/area management

#### Other

##### Use and trade

Use type: International

##### Ecosystem services

Ecosystem service type: Important

##### Research needed

Research needed: 1.2. Research - Population size, distribution & trends1.3. Research - Life history & ecology1.5. Research - Threats3.1. Monitoring - Population trends3.4. Monitoring - Habitat trends

Justification for research needed: Further research on basic information such natural history, ecology and possible threats of the species are needed. Monitoring of population and habitat would be necessary to confirm future trends and to evaluate the effectiveness of regulating access to the caves.

### Troglohyphantes brignolii

#### Species information

Scientific name: Troglohyphantesbrignolii

Species authority: Deeleman-Reinhold, 1978

Kingdom: Animalia

Phylum: Arthropoda

Class: Arachnida

Order: Araneae

Family: Linyphiidae

**Figure 2.**
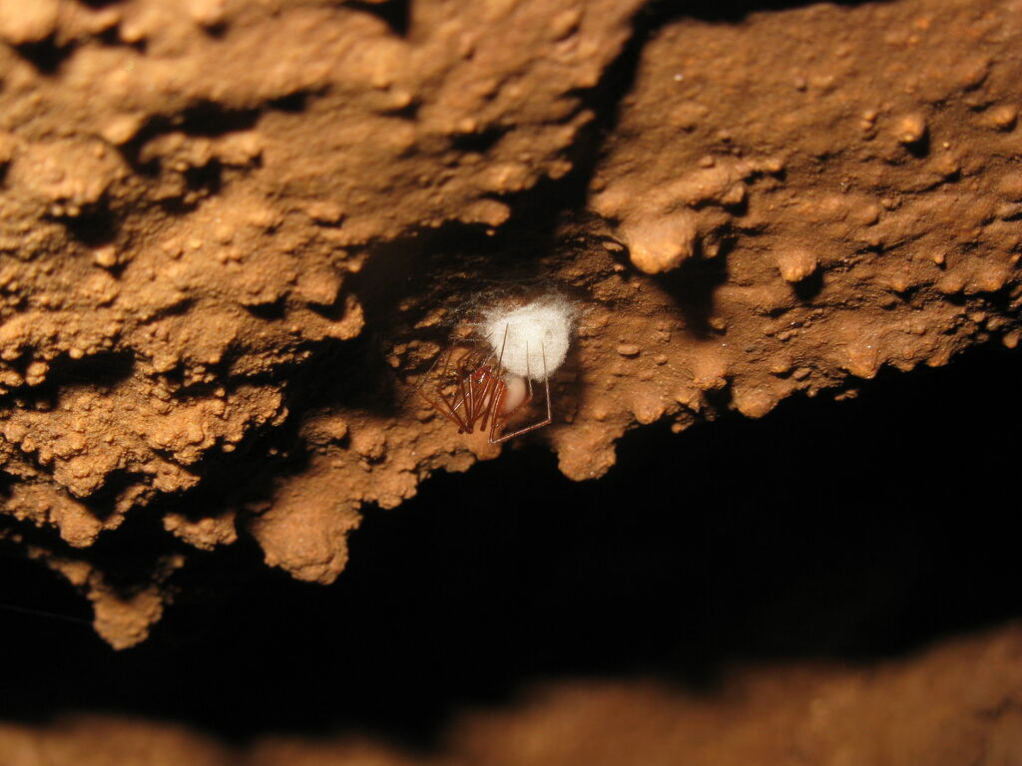
*Troglohyphantesbrignolii* Deeleman-Reinhold, 1978. Photo credit: Marko Lukić.

Figure(s) or Photo(s): Fig. 2

Region for assessment: Global

#### Editor & Reviewers

##### Reviewers

Reviewers: Marc MilnePaulo Borges

##### Editor

Editor: Pedro Cardoso

#### Geographic range

Biogeographic realm: Palearctic

Countries: Croatia

Map of records (Google Earth): Suppl. material 6

Basis of EOO and AOO: Species Distribution Model

Basis (narrative): Multiple sites are recorded for this non-specialised species. Therefore, it was possible to perform species distribution modelling to predict its potential range with confidence limits.

Min Elevation/Depth (m): 285

Max Elevation/Depth (m): 1086

Range description: This species has a small distribution range, restricted to Istria and Primorje-Gorski Kotar counties in north-western Croatia (detailed occurrences and relative references in Suppl. material 67).

#### Extent of occurrence

EOO (km2): 434-1026,691

Trend: Stable

Justification for trend: This species is not strictly relegated to deep subterranean habitats, being also collected at cave entrance and occasionally in surface habitats. It is plausible that anthropogenic climate change may affect the habitat suitability of this species. However, in view of the relatively wide thermal tolerance and the relatively high dispersal ability of non-specialised species of *Troglohyphantes* (Mammola et al. 2019a), the distribution range of *T.brignolii* is not expected to undergo significant reduction in the near future. A deeper study on the impacts of climate change on this species is required.

Causes ceased?: Yes

Causes understood?: Yes

Causes reversible?: Yes

Extreme fluctuations?: No

#### Area of occupancy

Trend: Stable

Justification for trend: This species is not strictly relegated to deep subterranean habitats, being also collected at cave entrance and occasionally in surface habitats. It is plausible that anthropogenic climate change may affect the habitat suitability of this species. However, in view of the relatively wide thermal tolerance and the relatively high dispersal ability of non-specialised species of *Troglohyphantes* (Mammola et al. 2019a), the distribution range of *T.brignolii* is not expected to undergo significant reduction in the near future. A deeper study on the impacts of climate change on this species is required.

Causes ceased?: Yes

Causes understood?: Yes

Causes reversible?: Yes

Extreme fluctuations?: No

AOO (km2): 432-824,600

#### Locations

Number of locations: Not applicable

Justification for number of locations: No known threats to this species.

Trend: Stable

Extreme fluctuations?: No

#### Population

Number of individuals: Unknown

Trend: Stable

Justification for trend: There are no currently known threats to the species.

Causes ceased?: Yes

Causes understood?: Yes

Causes reversible?: Yes

Extreme fluctuations?: No

#### Subpopulations

Number of subpopulations: Unknown

Trend: Unknown

Extreme fluctuations?: No

Severe fragmentation?: No

#### Habitat

System: Terrestrial

Habitat specialist: No

Habitat (narrative): This species has been collected both in deep-cave and in the vicinity of the cave entrance, sometimes in the twilight zone. In the type locality (Špilja iznad Velikog Bresta, Račja Vas), a few specimens were found just outside the entrance.

Trend in extent, area or quality?: Stable

Justification for trend: The habitats colonised by *T.brignolii* are as yet not threatened by direct human activities.

##### Habitat

Habitat importance: Major Importance

Habitats: 1.4. Forest - Temperate7. Caves and Subterranean Habitats (non-aquatic)

#### Ecology

Size: 3.2 mm

Generation length (yr): 2

Dependency of single sp?: No

Ecology and traits (narrative): Not much is known about the ecology and life history of this species. This spider shows minor specialisation to subterranean life (Mammola et al. 2022a).

#### Threats

Justification for threats: This species is potentially exposed due to its limited geographic distribution range. However, the existence of direct threats is unknown for this species.

##### Threats

Threat type: Past

Threats: 12. Other options - Other threat

#### Conservation

Justification for conservation actions: Most of the predicted range of *T.brignolii* is included in the Natura 2000 network (SCI HR2001215 Boljunsko polje, SCI HR2000601 Park prirode Učka, SPA HR1000018 Učka i Ćićarija).

##### Conservation actions

Conservation action type: In Place

Conservation actions: 1.1. Land/water protection - Site/area protection1.2. Land/water protection - Resource & habitat protection

#### Other

##### Use and trade

Use type: International

##### Ecosystem services

Ecosystem service type: Important

##### Research needed

Research needed: 1.2. Research - Population size, distribution & trends1.3. Research - Life history & ecology1.5. Research - Threats

Justification for research needed: Research on basic information such as distribution, natural history, ecology and possible threats of the species would be needed.

### Troglohyphantes caligatus

#### Species information

Scientific name: Troglohyphantescaligatus

Species authority: Pesarini, 1989

Kingdom: Animalia

Phylum: Arthropoda

Class: Arachnida

Order: Araneae

Family: Linyphiidae

Region for assessment: Global

#### Editor & Reviewers

##### Reviewers

Reviewers: Marc MilnePaulo Borges

##### Editor

Editor: Pedro Cardoso

#### Geographic range

Biogeographic realm: Palearctic

Countries: SwitzerlandItaly

Map of records (Google Earth): Suppl. material 7

Basis of EOO and AOO: Observed

Basis (narrative): This species was collected in very few localities. Its low level of subterranean specialisation, together with the high altimetric range of its known distribution, possibly reflects a higher dispersal capacity compared to subterranean specialised species. Consequently, it may be possible that the present known range of this species is underestimated.

Min Elevation/Depth (m): 580

Max Elevation/Depth (m): 1538

Range description: This species is known only from Monte San Primo (province of Como, Lombardia, northern Italy) and Monte Generoso (Ticino, southern Switzerland) (see Suppl. material 67).

#### Extent of occurrence

EOO (km2): 79

Trend: Stable

Justification for trend: This species is not strictly relegated to deep subterranean habitats, being collected both in epigean and hypogean habitats. In view of the relatively wide thermal tolerance and the relatively high dispersal ability of non-specialised species of *Troglohyphantes* (Mammola et al. 2019a), the distribution range of *T.caligatus* is not expected to undergo significant reduction in the near future. A deeper study on the current distribution of this species and on the potential impacts of climate change is required.

Causes ceased?: Yes

Causes understood?: Yes

Causes reversible?: Yes

Extreme fluctuations?: No

#### Area of occupancy

Trend: Stable

Justification for trend: This species is not strictly relegated to deep subterranean habitats, being collected both in epigean and hypogean habitats. In view of the relatively wide thermal tolerance and the relatively high dispersal ability of non-specialised species of *Troglohyphantes* (Mammola et al. 2019a), the distribution range of *T.caligatus* is not expected to undergo significant reduction in the near future. A deeper study on the current distribution of this species and on the potential impacts of climate change is required.

Causes ceased?: Yes

Causes understood?: Yes

Causes reversible?: Yes

Extreme fluctuations?: No

AOO (km2): 28

#### Locations

Number of locations: Not applicable

Justification for number of locations: No known threats to this species.

Trend: Stable

Extreme fluctuations?: No

#### Population

Number of individuals: Unknown

Trend: Stable

Justification for trend: There are no currently known threats to the species.

Causes ceased?: Yes

Causes understood?: Yes

Causes reversible?: Yes

Extreme fluctuations?: No

#### Subpopulations

Number of subpopulations: Unknown

Trend: Unknown

Extreme fluctuations?: No

Severe fragmentation?: No

#### Habitat

System: Terrestrial

Habitat specialist: Yes

Habitat (narrative): There is poor information on the habitat of this species. Specimens have been collected both in hypogean and epigean habitats.

Trend in extent, area or quality?: Stable

##### Habitat

Habitat importance: Major Importance

Habitats: 1.4. Forest - Temperate7. Caves and Subterranean Habitats (non-aquatic)

#### Ecology

Size: 2.2 mm

Generation length (yr): 2

Dependency of single sp?: No

Ecology and traits (narrative): The ecology and life history of this species is unknown. This spider shows minor specialisation to subterranean life (Mammola et al. 2022a).

#### Threats

Justification for threats: This species is potentially exposed due to its limited geographic distribution range. However, the existence of threats is unknown for this species.

##### Threats

Threat type: Past

Threats: 12. Other options - Other threat

#### Other

##### Use and trade

Use type: International

##### Ecosystem services

Ecosystem service type: Important

##### Research needed

Research needed: 1.2. Research - Population size, distribution & trends1.3. Research - Life history & ecology1.5. Research - Threats

Justification for research needed: Research on basic information such as distribution, ecology, life cycle and possible threats throughout the range would be needed.

### Troglohyphantes caporiaccoi

#### Species information

Scientific name: Troglohyphantescaporiaccoi

Species authority: Brignoli, 1971

Kingdom: Animalia

Phylum: Arthropoda

Class: Arachnida

Order: Araneae

Family: Linyphiidae

Region for assessment: Global

#### Editor & Reviewers

##### Reviewers

Reviewers: Marc MilnePaulo Borges

##### Editor

Editor: Pedro Cardoso

#### Geographic range

Biogeographic realm: Palearctic

Countries: Italy

Map of records (Google Earth): Suppl. material 8

Basis of EOO and AOO: Observed

Basis (narrative): In light of its high level of subterranean specialisation, we assume that the known records of *T.caporiaccoi* are good proxies for defining AOO and EOO.

Min Elevation/Depth (m): 461

Max Elevation/Depth (m): 1082

Range description: This species is restricted to four caves in the Bergamasque Prealps (Lombardia, northern Italy) (detailed occurrences and relative references in Suppl. material 67).

#### Extent of occurrence

EOO (km2): 30

Trend: Decline (inferred)

Justification for trend: According to Mammola et al. (2018a), climate change will significantly affect the distribution of subterranean specialised *Troglohyphantes* in the future. Moreover, given the low tolerance to habitat changes as well as the low dispersal ability, a possible extreme reduction of the geographic range of this species is expected in the future.

Causes ceased?: No

Causes understood?: Yes

Causes reversible?: No

Extreme fluctuations?: No

#### Area of occupancy

Trend: Decline (inferred)

Justification for trend: According to Mammola et al. (2018a), climate change will significantly affect the distribution of subterranean specialised *Troglohyphantes* in the future. Moreover, given the low tolerance to habitat changes as well as the low dispersal ability, a possible extreme reduction of the geographic range of this species is expected in the future.

Causes ceased?: No

Causes understood?: Yes

Causes reversible?: No

Extreme fluctuations?: No

AOO (km2): 16

#### Locations

Number of locations: 1

Justification for number of locations: Even though this species occurs in four caves, these are interpreted as a single location, as they are all affected by changes in subterranean microclimatic conditions due to climate change.

Trend: Stable

Extreme fluctuations?: No

#### Population

Number of individuals: Unknown

Trend: Decline (inferred)

Justification for trend: In view of the reduced thermal tolerance of subterranean specialised *Troglohyphantes* species (Mammola et al. 2019a), alterations of the microclimatic conditions of the habitat due to climate change are expected to impact the whole population of this species.

Basis for decline: (c) a decline in area of occupancy, extent of occurrence and/or quality of habitat

Causes ceased?: No

Causes understood?: Yes

Causes reversible?: No

Extreme fluctuations?: No

#### Subpopulations

Number of subpopulations: 4

Trend: Decline (inferred)

Justification for trend: Due to the adaptation to the subterranean medium and of the narrow physiological tolerance of this species, likely hampering dispersal through non-subterranean habitats, each locality can reasonably host a single isolated subpopulation. Accordingly, for this species we identified four subpopulations, corresponding to the four caves where the species has been collected. These subpopulation are likely to be impacted by climate change.

Extreme fluctuations?: No

Severe fragmentation?: No

#### Habitat

System: Terrestrial

Habitat specialist: Yes

Habitat (narrative): The species is strictly relegated to cave habitat.

Trend in extent, area or quality?: Decline (inferred)

Justification for trend: As seen in Mammola et al. (2018a) for the species of the genus *Troglohyphantes* of the Western Alps, a drastic decline in the habitat suitability of *T.caporiaccoi* as a consequence of climate change is expected.

##### Habitat

Habitat importance: Major Importance

Habitats: 7. Caves and Subterranean Habitats (non-aquatic)

#### Ecology

Size: 2 mm

Generation length (yr): 4

Dependency of single sp?: No

Ecology and traits (narrative): This species shows a high degree of specialisation to deep subterranean habitats, with absence of pigmentation and remarkable eye regression (Mammola et al. 2022a).

#### Threats

Justification for threats: This species is potentially exposed due to its extremely narrow geographic distribution range and its presumably low dispersal capacity. As seen for the *Troglohyphantes* species of the Western Alps (Mammola et al. 2018a), climate warming is expected to reduce the currently suitable habitat for this spider.

##### Threats

Threat type: Future

Threats: 11.1. Climate change & severe weather - Habitat shifting & alteration11.2. Climate change & severe weather - Droughts11.3. Climate change & severe weather - Temperature extremes

#### Other

##### Use and trade

Use type: International

##### Ecosystem services

Ecosystem service type: Important

##### Research needed

Research needed: 1.2. Research - Population size, distribution & trends1.3. Research - Life history & ecology1.5. Research - Threats

Justification for research needed: Research on basic information such as distribution, ecology, life cycle and possible threats throughout the range would be needed.

### Troglohyphantes cavadinii

#### Species information

Scientific name: Troglohyphantescavadinii

Species authority: Pesarini, 1989

Kingdom: Animalia

Phylum: Arthropoda

Class: Arachnida

Order: Araneae

Family: Linyphiidae

Region for assessment: Global

#### Editor & Reviewers

##### Reviewers

Reviewers: Marc MilnePaulo Borges

##### Editor

Editor: Pedro Cardoso

#### Geographic range

Biogeographic realm: Palearctic

Countries: Italy

Map of records (Google Earth): Suppl. material 9

Basis of EOO and AOO: Unknown

Basis (narrative): This subterranean specialised species was collected only in two localities and it has not been recorded since its description. It may be possible that the species occurs in other caves in the area. The true range is therefore unknown and not possible to model with confidence.

Min Elevation/Depth (m): 818

Max Elevation/Depth (m): 818

Range description: This species was found only in the cave of Pozzo di Cedrina and in an unspecified "cave in the nearby of Cene", both in Val Seriana (province of Bergamo, Lombardia, northern Italy) (see Suppl. material 67). It was never recorded after the original description (Pantini and Isaia 2019).

#### Extent of occurrence

EOO (km2): Unknown

Trend: Decline (inferred)

Justification for trend: The available data on the distribution range of this spider are not enough to estimate the species extinction risk. However, given the low tolerance to habitat changes of subterranean specialised *Troglohyphantes* species (see Mammola et al. 2018a) as well as their very low dispersal ability, a possible extreme reduction of the geographic range of *T.cavadinii* is expected in the future.

Causes ceased?: No

Causes understood?: Yes

Causes reversible?: No

Extreme fluctuations?: No

#### Area of occupancy

Trend: Decline (inferred)

Justification for trend: The available data on the distribution range of this spider are not enough to estimate the species extinction risk. However, given the low tolerance to habitat changes of subterranean specialised *Troglohyphantes* species (see Mammola et al. 2018a) as well as their very low dispersal ability, a possible extreme reduction of the geographic range of *T.cavadinii* is expected in the future.

Causes ceased?: No

Causes understood?: Yes

Causes reversible?: No

Extreme fluctuations?: No

AOO (km2): Unknown

#### Locations

Number of locations: Unknown

Justification for number of locations: The data available are not enough to estimate the number of locations for this species.

Trend: Unknown

Extreme fluctuations?: No

#### Population

Number of individuals: Unknown

Trend: Decline (inferred)

Justification for trend: The population size is unknown. However, in view of the reduced thermal tolerance of subterranean specialised *Troglohyphantes* species (Mammola et al. 2019a), alterations of the microclimatic conditions of the habitat due to climate change are expected to impact the whole population of this species.

Causes ceased?: No

Causes understood?: Yes

Causes reversible?: No

Extreme fluctuations?: No

#### Subpopulations

Number of subpopulations: Unknown

Trend: Unknown

Extreme fluctuations?: No

Severe fragmentation?: No

#### Habitat

System: Terrestrial

Habitat specialist: Yes

Habitat (narrative): The species was collected in deep cave habitat. No additional information on the habitat was provided.

Trend in extent, area or quality?: Decline (inferred)

Justification for trend: As seen in Mammola et al. (2018a) for the western alpine species of *Troglohyphantes*, a drastic decline in the habitat suitability of *T.cavadinii* as a consequence of climate change is expected.

##### Habitat

Habitat importance: Major Importance

Habitats: 7. Caves and Subterranean Habitats (non-aquatic)

#### Ecology

Size: 2 mm

Generation length (yr): 4

Dependency of single sp?: No

Ecology and traits (narrative): Not much is known about the ecology of this species. This species shows a high degree of specialisation to deep subterranean habitats, with absence of pigmentation and pronounced eye regression (Mammola et al. 2022a).

#### Threats

Justification for threats: As seen for the *Troglohyphantes* species of the Western Alps (Mammola et al. 2018a), climate warming is expected to reduce the currently suitable habitat for this spider.

##### Threats

Threat type: Future

Threats: 11.1. Climate change & severe weather - Habitat shifting & alteration11.2. Climate change & severe weather - Droughts11.3. Climate change & severe weather - Temperature extremes

#### Other

##### Use and trade

Use type: International

##### Ecosystem services

Ecosystem service type: Important

##### Research needed

Research needed: 1.2. Research - Population size, distribution & trends1.3. Research - Life history & ecology1.5. Research - Threats

Justification for research needed: Research on basic information such as distribution, ecology, life cycle and possible threats through out the range would be needed.

### Troglohyphantes comottii

#### Species information

Scientific name: Troglohyphantescomottii

Species authority: Pesarini, 1989

Kingdom: Animalia

Phylum: Arthropoda

Class: Arachnida

Order: Araneae

Family: Linyphiidae

Region for assessment: Global

#### Editor & Reviewers

##### Reviewers

Reviewers: Marc MilnePaulo Borges

##### Editor

Editor: Pedro Cardoso

#### Geographic range

Biogeographic realm: Palearctic

Countries: Italy

Map of records (Google Earth): Suppl. material 10

Basis of EOO and AOO: Unknown

Basis (narrative): This subterranean specialised species was collected only in one locality, and it has never been recorded again after its description. It may be possible that the species occurs in other caves in the area. The true range is therefore unknown and not possible to model with confidence.

Min Elevation/Depth (m): 396

Max Elevation/Depth (m): 396

Range description: This species is known only from one cave (Grotta Lacù di Casai), in the province of Bergamo (Lombardia, northern Italy) (see Suppl. material 67). It is uniquely known for the type locality, as it was never recorded after the original description (Pantini and Isaia 2019).

#### Extent of occurrence

EOO (km2): Unknown

Trend: Decline (inferred)

Justification for trend: The available data on the distribution range of this spider are not sufficient to estimate its extinction risk. However, given the low tolerance to habitat changes of subterranean specialised *Troglohyphantes* species (see Mammola et al. 2019a) as well as their very low dispersal ability, a possible extreme reduction of the geographic range of *T.comottii* is expected in the future.

Causes ceased?: No

Causes understood?: Yes

Causes reversible?: No

Extreme fluctuations?: No

#### Area of occupancy

Trend: Decline (inferred)

Justification for trend: The available data on the distribution range of this spider are not sufficient to estimate its extinction risk. However, given the low tolerance to habitat changes of subterranean specialised *Troglohyphantes* species (see Mammola et al. 2019a) as well as their very low dispersal ability, a possible extreme reduction of the geographic range of *T.comottii* is expected in the future.

Causes ceased?: No

Causes understood?: Yes

Causes reversible?: No

Extreme fluctuations?: No

AOO (km2): Unknown

#### Locations

Number of locations: Unknown

Justification for number of locations: The data available are not enough to estimate the number of locations for this species.

Trend: Unknown

Extreme fluctuations?: No

#### Population

Number of individuals: Unknown

Trend: Decline (inferred)

Justification for trend: The population size is unknown. However, in view of the reduced thermal tolerance of subterranean specialised *Troglohyphantes* species (Mammola et al. 2019a), alterations of the microclimatic conditions of the habitat due to climate change are expected to impact the whole population of this species.

Causes ceased?: No

Causes understood?: Yes

Causes reversible?: No

Extreme fluctuations?: No

#### Subpopulations

Number of subpopulations: Unknown

Trend: Unknown

Extreme fluctuations?: No

Severe fragmentation?: No

#### Habitat

System: Terrestrial

Habitat specialist: Yes

Habitat (narrative): There is poor information on species habitat, although specimens have been collected in caves.

Trend in extent, area or quality?: Decline (inferred)

Justification for trend: As seen in Mammola et al. (2018a) for the species of the genus *Troglohyphantes* of the Western Alps, a drastic decline in the habitat suitability of *T.comottii* as a consequence of climate change is expected.

##### Habitat

Habitat importance: Major Importance

Habitats: 7. Caves and Subterranean Habitats (non-aquatic)

#### Ecology

Size: 2 mm

Generation length (yr): 4

Dependency of single sp?: No

Ecology and traits (narrative): This species shows a high degree of specialisation to deep subterranean habitats, with absence of pigmentation and pronounced eye regression (Mammola et al. 2022a).

#### Threats

Justification for threats: As seen for the Western alpine *Troglohyphantes* species (Mammola et al. 2018a), climate warming is expected to reduce the currently suitable habitat for this spider.

##### Threats

Threat type: Future

Threats: 11.1. Climate change & severe weather - Habitat shifting & alteration11.2. Climate change & severe weather - Droughts11.3. Climate change & severe weather - Temperature extremes

#### Other

##### Use and trade

Use type: International

##### Ecosystem services

Ecosystem service type: Important

##### Research needed

Research needed: 1.2. Research - Population size, distribution & trends1.3. Research - Life history & ecology1.5. Research - Threats

Justification for research needed: Research on basic information such as distribution, ecology, life cycle and possible threats throughout the range would be needed.

### Troglohyphantes confusus

#### Species information

Scientific name: Troglohyphantesconfusus

Species authority: Kratochvíl, 1939

Kingdom: Animalia

Phylum: Arthropoda

Class: Arachnida

Order: Araneae

Family: Linyphiidae

Region for assessment: Global

#### Editor & Reviewers

##### Reviewers

Reviewers: Marc MilnePaulo Borges

##### Editor

Editor: Pedro Cardoso

#### Geographic range

Biogeographic realm: Palearctic

Countries: Slovenia

Map of records (Google Earth): Suppl. material 11

Basis of EOO and AOO: Observed

Basis (narrative): This non-specialised spider was collected in very few localities. Its low level of subterranean specialisation possibly reflects a higher dispersal capacity when compared to subterranean specialised species. Consequently, it may be possible that the present known range of this species is underestimated.

Min Elevation/Depth (m): 453

Max Elevation/Depth (m): 650

Range description: *Troglohyphantesconfusus* is restricted to a few localities in Inner Carniola (north-western Slovenia) (detailed occurrences and relative references in Suppl. material 67).

#### Extent of occurrence

EOO (km2): 202

Trend: Stable

Justification for trend: This species is not strictly relegated to deep subterranean habitats, being mostly collected in epigean habitats. It is plausible that anthropogenic climate change may affect the habitat suitability of this species. However, in view of the relatively wide thermal tolerance and the relatively high dispersal ability of non-specialised species of *Troglohyphantes* (Mammola et al. 2019a), the distribution range of *T.confusus* is not expected to undergo significant reduction in the near future. A deeper study on the current distribution of this species and on the potential impacts of climate change is required.

Causes ceased?: Yes

Causes understood?: Yes

Causes reversible?: Yes

Extreme fluctuations?: No

#### Area of occupancy

Trend: Stable

Justification for trend: This species is not strictly relegated to deep subterranean habitats, being mostly collected in epigean habitats. It is plausible that anthropogenic climate change may affect the habitat suitability of this species. However, in view of the relatively wide thermal tolerance and the relatively high dispersal ability of non-specialised species of *Troglohyphantes* (Mammola et al. 2019a), the distribution range of *T.confusus* is not expected to undergo significant reduction in the near future. A deeper study on the current distribution of this species and on the potential impacts of climate change is required.

Causes ceased?: Yes

Causes understood?: Yes

Causes reversible?: Yes

Extreme fluctuations?: No

AOO (km2): 24

#### Locations

Number of locations: Not applicable

Justification for number of locations: No known threats to this species.

Trend: Stable

Extreme fluctuations?: No

#### Population

Number of individuals: Unknown

Trend: Stable

Justification for trend: There are no currently known threats to the species.

Causes ceased?: Yes

Causes understood?: Yes

Causes reversible?: Yes

Extreme fluctuations?: No

#### Subpopulations

Number of subpopulations: Unknown

Trend: Unknown

Extreme fluctuations?: No

Severe fragmentation?: No

#### Habitat

System: Terrestrial

Habitat specialist: No

Habitat (narrative): This species was mainly found in beech forests, in burrows under litter. Some specimens have been collected in the anterior part of a cave, in ethylene-glycol pitfalls.

Trend in extent, area or quality?: Stable

##### Habitat

Habitat importance: Major Importance

Habitats: 1. Forest7.2. Caves and Subterranean Habitats (non-aquatic) - Other Subterranean Habitats

#### Ecology

Size: 2.6 mm

Generation length (yr): 2

Dependency of single sp?: No

Ecology and traits (narrative): Specimens show a low degree of morphological specialisation to subterranean life (Mammola et al. 2022a).

#### Threats

Justification for threats: The existence of threats is unknown for this species.

##### Threats

Threat type: Past

Threats: 12. Other options - Other threat

#### Conservation

Justification for conservation actions: Some of the known localities of this species fall within the Special Area of Conservation of the Trnovo Forest (SAC SI3000255).

##### Conservation actions

Conservation action type: In Place

Conservation actions: 1.1. Land/water protection - Site/area protection1.2. Land/water protection - Resource & habitat protection2.1. Land/water management - Site/area management

#### Other

##### Use and trade

Use type: International

##### Ecosystem services

Ecosystem service type: Important

##### Research needed

Research needed: 1.2. Research - Population size, distribution & trends1.3. Research - Life history & ecology1.5. Research - Threats

Justification for research needed: Research on basic information such as distribution, ecology, life cycle and possible threats throughout the range would be needed.

### Troglohyphantes croaticus

#### Species information

Scientific name: Troglohyphantescroaticus

Species authority: (Chyzer, 1894)

Kingdom: Animalia

Phylum: Arthropoda

Class: Arachnida

Order: Araneae

Family: Linyphiidae

**Figure 3.**
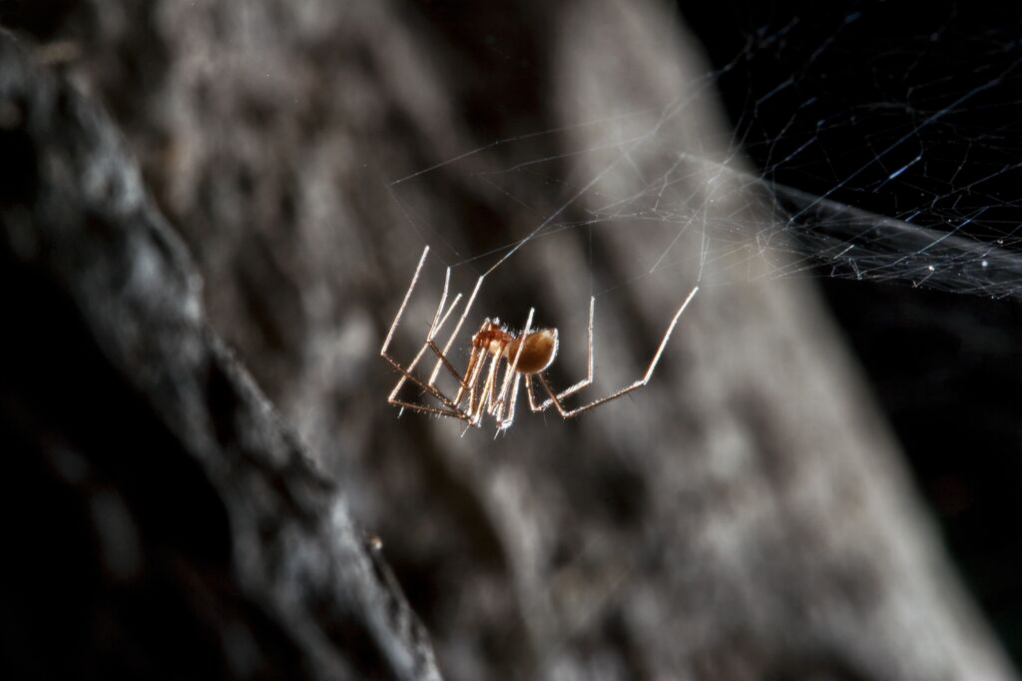
*Troglohyphantescroaticus* (Chyzer, 1894). Photo credit: Jana Bedek.

Figure(s) or Photo(s): Fig. 3

Region for assessment: Global

#### Editor & Reviewers

##### Reviewers

Reviewers: Marc MilnePaulo Borges

##### Editor

Editor: Pedro Cardoso

#### Geographic range

Biogeographic realm: Palearctic

Countries: Croatia

Map of records (Google Earth): Suppl. material 12

Basis of EOO and AOO: Species Distribution Model

Basis (narrative): This species is known from multiple localities. Therefore, it was possible to perform species distribution modelling to predict its potential range with confidence limits. See Methods for details.

Min Elevation/Depth (m): 203

Max Elevation/Depth (m): 1378

Range description: This species has been found in several caves in the Primorje-Gorski Kotar, Karlovac, and Lika-Senj counties (north-western Croatia) (detailed occurrences and relative references in Suppl. material 67). The species distribution model predicts the presence of *T.croaticus* outside the known range, in the southern part of Slovenia.

#### Extent of occurrence

EOO (km2): 2664-4664,3618

Trend: Stable

Justification for trend: This species is not strictly relegated to deep subterranean habitats, being collected in the twilight zone and in the deep parts of the caves. It is plausible that anthropogenic climate change may affect the habitat suitability of this species. However, in view of the relatively wide thermal tolerance and the relatively high dispersal ability of non-specialised species of *Troglohyphantes* (Mammola et al. 2019a), the distribution range of *T.croaticus* is not expected to undergo significant reduction in the near future. A deeper study on the impacts of climate change on this species is required.

Causes ceased?: Yes

Causes understood?: Yes

Causes reversible?: Yes

Extreme fluctuations?: No

#### Area of occupancy

Trend: Stable

Justification for trend: This species is not strictly relegated to deep subterranean habitats, being collected in the twilight zone and in the deep parts of the caves. It is plausible that anthropogenic climate change may affect the habitat suitability of this species. However, in view of the relatively wide thermal tolerance and the relatively high dispersal ability of non-specialised species of *Troglohyphantes* (Mammola et al. 2019a), the distribution range of *T.croaticus* is not expected to undergo significant reduction in the near future. A deeper study on the impacts of climate change on this species is required.

Causes ceased?: Yes

Causes understood?: Yes

Causes reversible?: Yes

Extreme fluctuations?: No

AOO (km2): 1700-3272,2536

#### Locations

Number of locations: Not applicable

Justification for number of locations: No known threats to this species.

Trend: Stable

Extreme fluctuations?: No

#### Population

Number of individuals: Unknown

Trend: Stable

Justification for trend: There are no currently known threats to the species.

Causes ceased?: Yes

Causes understood?: Yes

Causes reversible?: Yes

Extreme fluctuations?: No

#### Subpopulations

Number of subpopulations: Unknown

Trend: Unknown

Extreme fluctuations?: No

Severe fragmentation?: No

#### Habitat

System: Terrestrial

Habitat specialist: No

Habitat (narrative): Specimens have been collected both at the cave entrance and deeper in the cave, in webs amongst boulders on the cave floor and on the walls. Although, to our knowledge, for the majority of localities no threats are detectable, the species has apparently disappeared from the type locality, Bukovac Cave. It seems likely that extensive archaeological and paleontological excavations carried out in this cave have altered significantly the habitat of this species. Moreover, Cave Vrelo is a touristic cave with more than 10,000 visitors per year, in which concerts, exhibitions and filming of movies are organised, with the possible accumulation of litter that may alter the local microhabitat.

Trend in extent, area or quality?: Stable

Justification for trend: Most of the caves inhabited by *T.croaticus* are as yet not threatened by direct human activities. However two localities seems to be disturbed by past and ongoing human activities, i.e. excavations and touristic use.

##### Habitat

Habitat importance: Major Importance

Habitats: 7. Caves and Subterranean Habitats (non-aquatic)

#### Ecology

Size: 2.8 mm

Generation length (yr): 2

Dependency of single sp?: No

Ecology and traits (narrative): Specimens collected in the eastern part of the distribution (in the regions of Kordun and Ogulinsko-plaščansko područje) are depigmented and microphthalm. Specimens from the western part of the distribution (Gorski kotar region) appear to be rather variable, from eyeless depigmented individuals to fully pigmented ones (even with a pattern on the opisthosoma) and normal eyes. According to the latest population genetic study of the species (Pavlek et al. in press), there is a clear separation on eastern and western subpopulations, with no evidence of gene flow between them.

#### Threats

Justification for threats: This species is potentially exposed due to its limited geographic distribution range. However, the existence of direct threats is unknown for this species. One of the caves where this species occurs is a show cave and visitors access it frequently.

##### Threats

Threat type: Past

Threats: 12. Other options - Other threat

#### Conservation

Justification for conservation actions: Most of the predicted range of *T.croaticus* is included in the Risnjak National Park and in the Natura 2000 network (SCI HR2000447 Nacionalni park Risnjak, SCI HR2000592 Ogulinsko-plaščansko područje, SCI HR2000591 Klek, SCI HR2000643 Obruč, SCI HR2000707 Gornje Jelenje prema Platku, SCI HR2001041 Gomance, SCI HR2001340 Područje oko Kuštrovke, SCI HR2001353 Lokve-Sunger-Fužine, SCI/SPA HR5000019 Gorski kotar i sjeverna Lika). The species distribution modelling predicts that this species could also be present in the Slovenian protected site of Snežnik (SAC SI3000231 Javorniki - Snežnik, SPA SI5000002 Snežnik - Pivka).

##### Conservation actions

Conservation action type: In Place

Conservation actions: 1.1. Land/water protection - Site/area protection1.2. Land/water protection - Resource & habitat protection

#### Other

##### Use and trade

Use type: International

##### Ecosystem services

Ecosystem service type: Important

##### Research needed

Research needed: 1.2. Research - Population size, distribution & trends1.3. Research - Life history & ecology1.5. Research - Threats

Justification for research needed: Research on basic information such as distribution, natural history, ecology and possible threats of the species would be needed.

### Troglohyphantes cruentus

#### Species information

Scientific name: Troglohyphantescruentus

Species authority: Brignoli, 1971

Kingdom: Animalia

Phylum: Arthropoda

Class: Arachnida

Order: Araneae

Family: Linyphiidae

Region for assessment: Global

#### Editor & Reviewers

##### Reviewers

Reviewers: Marc MilnePaulo Borges

##### Editor

Editor: Pedro Cardoso

#### Geographic range

Biogeographic realm: Palearctic

Countries: Slovenia

Map of records (Google Earth): Suppl. material 13

Basis of EOO and AOO: Unknown

Basis (narrative): This non-specialised species was collected in a single locality. It may be possible that the species occurs in other localities in the area. The true range is therefore unknown and not possible to model with confidence.

Min Elevation/Depth (m): 500

Max Elevation/Depth (m): 500

Range description: The species is known only from one locality outside the cave of Smoganica, in the municipality of Most na Soči (Slovenian Littoral) (see Suppl. material 67).

#### Extent of occurrence

EOO (km2): Unknown

Trend: Unknown

Causes ceased?: Unknown

Causes understood?: Unknown

Causes reversible?: Unknown

Extreme fluctuations?: No

#### Area of occupancy

Trend: Unknown

Causes ceased?: Unknown

Causes understood?: Unknown

Causes reversible?: Unknown

Extreme fluctuations?: No

AOO (km2): Unknown

#### Locations

Number of locations: Unknown

Justification for number of locations: The data available are not enough to estimate the number of locations for this species.

Trend: Unknown

Extreme fluctuations?: No

#### Population

Number of individuals: Unknown

Trend: Unknown

Justification for trend: The population size and trend are unknown.

Causes ceased?: Unknown

Causes understood?: Unknown

Causes reversible?: Unknown

Extreme fluctuations?: No

#### Subpopulations

Number of subpopulations: Unknown

Trend: Unknown

Extreme fluctuations?: No

Severe fragmentation?: No

#### Habitat

System: Terrestrial

Habitat specialist: No

Habitat (narrative): The species was collected just outside a cave. No additional information about the habitat was provided.

Trend in extent, area or quality?: Unknown

##### Habitat

Habitat importance: Major Importance

Habitats: 7. Caves and Subterranean Habitats (non-aquatic)

#### Ecology

Size: 2.6 mm

Generation length (yr): 2

Dependency of single sp?: No

Ecology and traits (narrative): Not much is known about the ecology of this species. The female is unknown (Nentwig et al. 2022). Specimens are depigmented (Mammola et al. 2022a).

#### Threats

Justification for threats: Unknown threats.

##### Threats

Threat type: Past

Threats: 12. Other options - Other threat

#### Conservation

Justification for conservation actions: This species was considered as potentially threatened due to its rarity and included in the category R of the Slovenian Red List (Uradni list RS št. 82/02 in 42/10). The single known locality of this spider is currently protected (SAC SI3000209 Jama pod Smoganico) and not open to the public.

##### Conservation actions

Conservation action type: In Place

Conservation actions: 1.1. Land/water protection - Site/area protection1.2. Land/water protection - Resource & habitat protection2.1. Land/water management - Site/area management

#### Other

##### Use and trade

Use type: International

##### Ecosystem services

Ecosystem service type: Important

##### Research needed

Research needed: 1.2. Research - Population size, distribution & trends1.3. Research - Life history & ecology1.5. Research - Threats

Justification for research needed: Research on basic information such as distribution, natural history, ecology and possible threats of the species would be needed.

### Troglohyphantes delphinicus

#### Species information

Scientific name: Troglohyphantesdelphinicus

Species authority: Isaia & Mammola, 2022

Kingdom: Animalia

Phylum: Arthropoda

Class: Arachnida

Order: Araneae

Family: Linyphiidae

Taxonomic notes: In Mammola et al. (2018a), this species was treated as *T.vignai* (partim). As it is a subterranean specialised species, the results of ecological niche modelling used to project future habitat suitability in Mammola et al. (2018a) are likely transferrable to this species.

**Figure 4.**
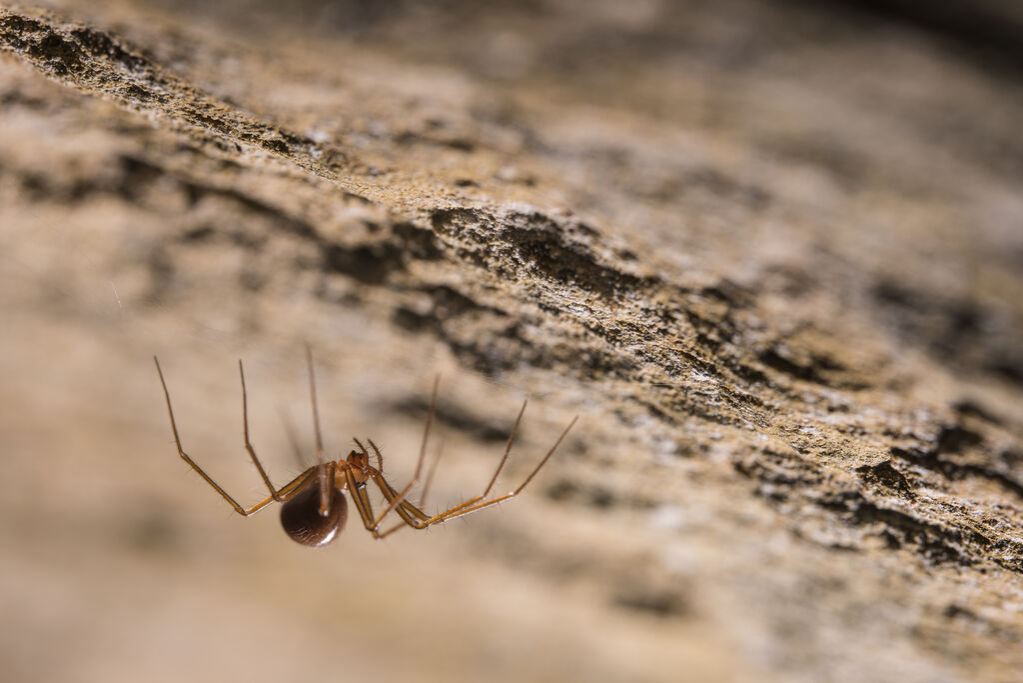
*Troglohyphantesdelphinicus* Isaia & Mammola, 2022. Photo credit: Emanuele Biggi.

Figure(s) or Photo(s): Fig. 4

Region for assessment: Global

#### Editor & Reviewers

##### Reviewers

Reviewers: Marc MilnePaulo Borges

##### Editor

Editor: Pedro Cardoso

#### Geographic range

Biogeographic realm: Palearctic

Countries: Italy

Map of records (Google Earth): Suppl. material 14

Basis of EOO and AOO: Observed

Basis (narrative): Caves in Western Alps have been extensively sampled, allowing to define EOO and AOO of this species with reasonable confidence.

Min Elevation/Depth (m): 1000

Max Elevation/Depth (m): 2480

Range description: The species is known in a few caves of Varaita Valley (Southern Cottian Alps) and in an isolated locality in Gesso Valley (Maritime Alps) (Piemonte, north-western Italy, detailed occurrences and relative references in Suppl. material 67).

#### Extent of occurrence

EOO (km2): 459

Trend: Decline (inferred)

Justification for trend: As seen in Mammola et al. (2018a) for other spiders of the genus *Troglohyphantes* of the Western Alps, climate change is expected to affect the distribution of this species in the future. Given the narrow thermal tolerance and its low dispersal ability (Isaia et al. 2022), a reduction of the distribution range of this species is expected in the future.

Causes ceased?: No

Causes understood?: Yes

Causes reversible?: No

Extreme fluctuations?: No

#### Area of occupancy

Trend: Decline (inferred)

Justification for trend: As seen in Mammola et al. (2018a) for other spiders of the genus *Troglohyphantes* of the Western Alps, climate change is expected to affect the distribution of this species in the future. Given the narrow thermal tolerance and its low dispersal ability (Isaia et al. 2022), a reduction of the distribution range of this species is expected in the future.

Causes ceased?: No

Causes understood?: Yes

Causes reversible?: No

Extreme fluctuations?: No

AOO (km2): 24

#### Locations

Number of locations: 1

Justification for number of locations: The habitat where this species occurs is affected by changes in subterranean microclimatic conditions due to climate change, which is expected to impact the whole population (see Mammola et al. 2018a).

Trend: Stable

Extreme fluctuations?: No

#### Population

Number of individuals: Unknown

Trend: Decline (inferred)

Justification for trend: In view of the reduced thermal tolerance of this species (Isaia et al. 2022), alterations of the microclimatic conditions of the habitat due to climate change are expected to impact the whole population.

Basis for decline: (c) a decline in area of occupancy, extent of occurrence and/or quality of habitat

Causes ceased?: No

Causes understood?: Yes

Causes reversible?: No

Extreme fluctuations?: No

#### Subpopulations

Number of subpopulations: 4

Trend: Decline (inferred)

Justification for trend: Due to the adaptation to the subterranean habitat and of the narrow physiological tolerance of this species, hampering dispersal through non-subterranean habitats (data on gene flow from Mammola et al. 2015), each locality reasonably hosts a single isolated subpopulation. Accordingly, for this species we identified four subpopulations, all of them occurring in the province of Cuneo (Piemonte, north-western Italy). All subpopulations are likely to be impacted by climate change.

Extreme fluctuations?: No

Severe fragmentation?: No

#### Habitat

System: Terrestrial

Habitat specialist: Yes

Habitat (narrative): *Troglohyphantesdelphinicus* was mainly collected in the dark zone of natural caves characterised by mean annual temperature values ranging from 0 to 8°C, at an altitude ranging from 1000 to 2480 m. In very few occasions, individuals were collected outside, in the vicinity of cave entrances in deep litter or under big stones (Isaia et al. 2022). A specimen was once collected in a pitfall trap in deep litter of a beech forest.

Trend in extent, area or quality?: Decline (inferred)

Justification for trend: As seen in Mammola et al. (2018a) for other western alpine species of the genus *Troglohyphantes*, a drastic decline in the habitat suitability of *T.delphinicus* as a consequence of climate change is expected.

##### Habitat

Habitat importance: Major Importance

Habitats: 7. Caves and Subterranean Habitats (non-aquatic)

#### Ecology

Size: 3 mm

Generation length (yr): 4

Dependency of single sp?: No

Ecology and traits (narrative): According to thermal tests, this species shows a narrow thermal tolerance, reaching 50% mortality at temperature values 1°C above its cave temperature (Isaia et al. 2022). Females are present all year, while males are rarely found in spring or early autumn and almost disappear in winter (Isaia et al. 2022).

#### Threats

Justification for threats: This species is potentially exposed due to its narrow geographic distribution range. As seen in Mammola et al. (2018a) for other species of the genus *Troglohyphantes* of the Western Alps, climate warming is expected to reduce the currently suitable habitat for this spider. Moreover, in view of its low thermal tolerance (Isaia et al. 2022), this species has a very limited dispersal ability, which represents an additional concern in face of the ongoing increase of temperature.

##### Threats

Threat type: Future

Threats: 11.1. Climate change & severe weather - Habitat shifting & alteration11.2. Climate change & severe weather - Droughts11.3. Climate change & severe weather - Temperature extremes

#### Conservation

Justification for conservation actions: One of the caves where this species has been collected is within the borders of the protected area of the Maritime Alps (SAC/SPA IT1160056).

##### Conservation actions

Conservation action type: In Place

Conservation actions: 1.1. Land/water protection - Site/area protection1.2. Land/water protection - Resource & habitat protection

#### Other

##### Use and trade

Use type: International

##### Ecosystem services

Ecosystem service type: Important

##### Research needed

Research needed: 1.2. Research - Population size, distribution & trends1.3. Research - Life history & ecology1.5. Research - Threats

Justification for research needed: Research on basic information such as distribution, ecology, life cycle and possible threats throughout the range would be needed.

### Troglohyphantes diabolicus

#### Species information

Scientific name: Troglohyphantesdiabolicus

Species authority: Deeleman-Reinhold, 1978

Kingdom: Animalia

Phylum: Arthropoda

Class: Arachnida

Order: Araneae

Family: Linyphiidae

Region for assessment: Global

#### Editor & Reviewers

##### Reviewers

Reviewers: Marc MilnePaulo Borges

##### Editor

Editor: Pedro Cardoso

#### Geographic range

Biogeographic realm: Palearctic

Countries: Slovenia

Map of records (Google Earth): Suppl. material 15

Basis of EOO and AOO: Observed

Basis (narrative): This species was collected in a few localities. Its low level of subterranean specialisation, possibly reflects a higher dispersal capacity when compared to subterranean specialised species. Consequently, it may be possible that the present known range of this species is underestimated.

Min Elevation/Depth (m): 302

Max Elevation/Depth (m): 728

Range description: This species is known from three caves of mount Tisnik in Carinthia, and from four caves in Styria (northern Slovenia) (detailed occurrences and relative references in Suppl. material 67). The species has been also collected in small mammal burrows in beech forests of the Savinian Alps (Styria, Slovenia).

#### Extent of occurrence

EOO (km2): 622

Trend: Unknown

Justification for trend: This species is not strictly relegated to deep subterranean environments, being collected also in shallow subterranean environments. It is plausible that anthropogenic climate change may affect the habitat suitability of this species. However, in view of the relatively wide thermal tolerance and the relatively high dispersal ability of non-specialised species of *Troglohyphantes* (Mammola et al. 2019a), the distribution range of *T.diabolicus* is not expected to undergo significant reduction in the near future. A deeper study on the current distribution of this species and on the potential impacts of climate change is required.

Causes ceased?: Yes

Causes understood?: Yes

Causes reversible?: Yes

Extreme fluctuations?: No

#### Area of occupancy

Trend: Stable

Justification for trend: This species is not strictly relegated to deep subterranean habitats, being collected also in shallow subterranean environments. It is plausible that anthropogenic climate change may affect the habitat suitability of this species. However, in view of the relatively wide thermal tolerance and the relatively high dispersal ability of non-specialised species of *Troglohyphantes* (Mammola et al. 2019a), the distribution range of *T.diabolicus* is not expected to undergo significant reduction in the near future. A deeper study on the current distribution of this species and on the potential impacts of climate change is required.

Causes ceased?: Yes

Causes understood?: Yes

Causes reversible?: Yes

Extreme fluctuations?: No

AOO (km2): 28

#### Locations

Number of locations: Not applicable

Justification for number of locations: No known threats to this species.

Trend: Stable

Extreme fluctuations?: No

#### Population

Number of individuals: Unknown

Trend: Stable

Justification for trend: There are no currently known threats to the species.

Causes ceased?: Yes

Causes understood?: Yes

Causes reversible?: Yes

Extreme fluctuations?: No

#### Subpopulations

Number of subpopulations: Unknown

Trend: Unknown

Extreme fluctuations?: No

Severe fragmentation?: No

#### Habitat

System: Terrestrial

Habitat specialist: No

Habitat (narrative): Specimens have been mainly collected in subterranean environments and occasionally in shallow subterranean habitats. In the Huda Luknja Cave, a high concentration of specimens of *T.diabolicus* has been found among the debris of a decayed wooden bridge in the front section, not far from the entrance opening. In Knapovska jama, a deserted artificial pit, the spiders had their webs amongst wood debris and in holes in the floor left by buttress poles, 10-30 m from the entrance. Specimens have been collected also in small mammal burrows in beech forests.

Trend in extent, area or quality?: Stable

##### Habitat

Habitat importance: Major Importance

Habitats: 1.4. Forest - Temperate7. Caves and Subterranean Habitats (non-aquatic)

#### Ecology

Size: 3.1 mm

Generation length (yr): 2

Dependency of single sp?: No

Ecology and traits (narrative): Not much is known about the ecology and life history of this species. This spider shows minor morphological specialisation to subterranean life (Mammola et al. 2022a).

#### Threats

Justification for threats: This species is potentially exposed due to its limited geographic distribution range. However, the existence of threats is unknown for this species.

##### Threats

Threat type: Past

Threats: 12. Other options - Other threat

#### Conservation

Justification for conservation actions: *Troglohyphantesdiabolicus* was considered potentially threatened and listed in the Slovenian Red List, in the category R (Uradni list RS št. 82/02 in 42/10). Part of the distribution of this species is included in the Ponikovski kras Regional Park and in the Special Area of Conservation of Huda luknja (SAC SI3000224).

##### Conservation actions

Conservation action type: In Place

Conservation actions: 1.1. Land/water protection - Site/area protection1.2. Land/water protection - Resource & habitat protection4. Education & awareness

#### Other

##### Use and trade

Use type: International

##### Ecosystem services

Ecosystem service type: Important

##### Research needed

Research needed: 1.2. Research - Population size, distribution & trends1.3. Research - Life history & ecology1.5. Research - Threats

Justification for research needed: Research on basic information such as distribution, natural history, ecology and possible threats of the species would be needed.

### Troglohyphantes dominici

#### Species information

Scientific name: Troglohyphantesdominici

Species authority: Pesarini, 1988

Kingdom: Animalia

Phylum: Arthropoda

Class: Arachnida

Order: Araneae

Family: Linyphiidae

Region for assessment: Global

#### Editor & Reviewers

##### Reviewers

Reviewers: Marc MilnePaulo Borges

##### Editor

Editor: Pedro Cardoso

#### Geographic range

Biogeographic realm: Palearctic

Countries: Italy

Map of records (Google Earth): Suppl. material 16

Basis of EOO and AOO: Species Distribution Model

Basis (narrative): Multiple collection sites are known for this species. Therefore, it was possible to perform species distribution modelling to predict its potential range with confidence limits. See Methods for details.

Min Elevation/Depth (m): 338

Max Elevation/Depth (m): 2200

Range description: This species has been found in several localities on the Bergamasque Alps and Prealps (Lombardia, northern Italy) (detailed occurrences and relative references in Suppl. material 67).

#### Extent of occurrence

EOO (km2): 1056-1859,1241

Trend: Stable

Justification for trend: This species has been mainly found in shallow subterranean and epigean environments. It is plausible that anthropogenic climate change may affect the habitat suitability of this species. However, in view of the relatively wide thermal tolerance and the relatively high dispersal ability of non-specialised species of *Troglohyphantes* (Mammola et al. 2019a), the distribution range of *T.dominici* is not expected to undergo significant reduction in the next future. A deeper study on the impacts of climate change on this species is required.

Causes ceased?: Yes

Causes understood?: Yes

Causes reversible?: Yes

Extreme fluctuations?: No

#### Area of occupancy

Trend: Stable

Justification for trend: This species has been mainly found in shallow subterranean and epigean environments. It is plausible that anthropogenic climate change may affect the habitat suitability of this species. However, in view of the relatively wide thermal tolerance and the relatively high dispersal ability of non-specialised species of *Troglohyphantes* (Mammola et al. 2019a), the distribution range of *T.dominici* is not expected to undergo significant reduction in the next future. A deeper study on the impacts of climate change on this species is required.

Causes ceased?: Yes

Causes understood?: Yes

Causes reversible?: Yes

Extreme fluctuations?: No

AOO (km2): 840-1632,1028

#### Locations

Number of locations: Not applicable

Justification for number of locations: No known threats to this species.

Trend: Stable

Extreme fluctuations?: No

#### Population

Number of individuals: Unknown

Trend: Stable

Justification for trend: There are no currently known threats to the species.

Causes ceased?: Yes

Causes understood?: Yes

Causes reversible?: Yes

Extreme fluctuations?: No

#### Subpopulations

Number of subpopulations: Unknown

Trend: Unknown

Extreme fluctuations?: No

Severe fragmentation?: No

#### Habitat

System: Terrestrial

Habitat specialist: No

Habitat (narrative): This species has been found in both shallow subterranean and epigean habitats, such as rocky lands, alpine prairies, *Rhododendron* scrublands, at high altitudes up to 2,200 m.

Trend in extent, area or quality?: Stable

##### Habitat

Habitat importance: Major Importance

Habitats: 3.4. Shrubland - Temperate4.4. Grassland - Temperate6. Rocky areas (e.g. inland cliffs, mountain peaks)7. Caves and Subterranean Habitats (non-aquatic)

#### Ecology

Size: 2.2 mm

Generation length (yr): 2

Dependency of single sp?: No

Ecology and traits (narrative): Not much is known about the ecology and life history of this species. This spider shows minor specialisation to subterranean life (Mammola et al. 2022a).

#### Threats

Justification for threats: The existence of direct threats is unknown for this species.

##### Threats

Threat type: Past

Threats: 12. Other options - Other threat

#### Conservation

Justification for conservation actions: This species has been recorded within the Natura 2000 network (SAC IT2060009 Val Nossana - Cima di Grem, SPA IT2060401 Parco Regionale Orobie Bergamasche).

##### Conservation actions

Conservation action type: In Place

Conservation actions: 1.1. Land/water protection - Site/area protection1.2. Land/water protection - Resource & habitat protection2.1. Land/water management - Site/area management

#### Other

##### Use and trade

Use type: International

##### Ecosystem services

Ecosystem service type: Important

##### Research needed

Research needed: 1.2. Research - Population size, distribution & trends1.3. Research - Life history & ecology1.5. Research - Threats

Justification for research needed: Research on basic information such as distribution, natural history, ecology and possible threats of the species would be needed.

### Troglohyphantes excavatus

#### Species information

Scientific name: Troglohyphantesexcavatus

Species authority: Fage, 1919

Kingdom: Animalia

Phylum: Arthropoda

Class: Arachnida

Order: Araneae

Family: Linyphiidae

**Figure 5.**
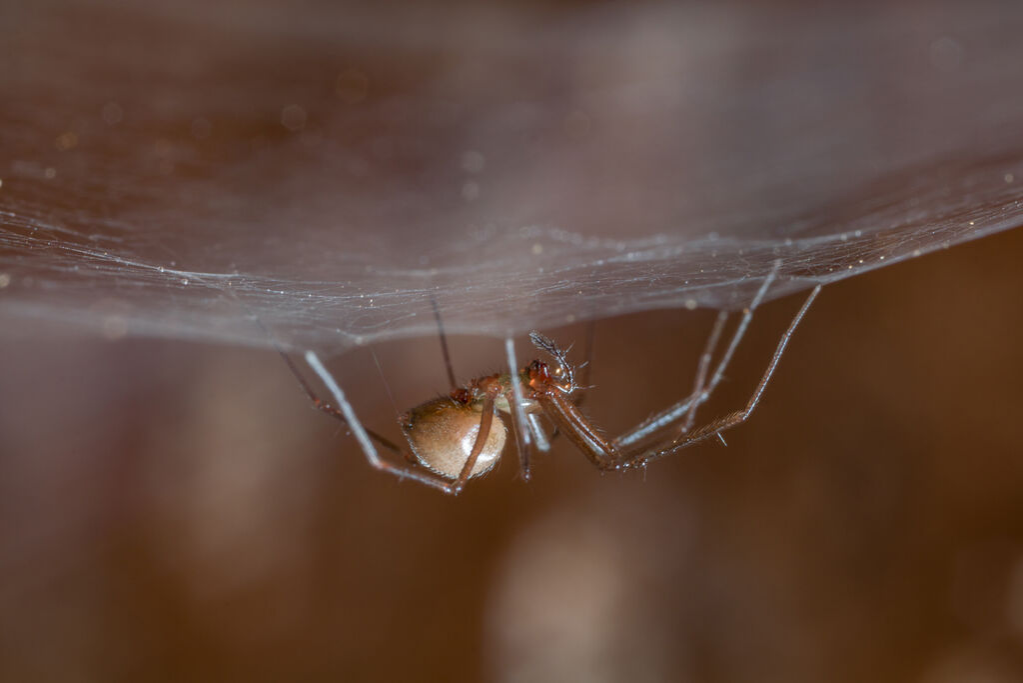
*Troglohyphantesexcavatus* Fage, 1919. Photo credit: Martina Pavlek.

Figure(s) or Photo(s): Fig. 5

Region for assessment: Global

#### Editor & Reviewers

##### Reviewers

Reviewers: Marc MilnePaulo Borges

##### Editor

Editor: Pedro Cardoso

#### Geographic range

Biogeographic realm: Palearctic

Countries: CroatiaSloveniaAustriaItaly

Map of records (Google Earth): Suppl. material 17

Basis of EOO and AOO: Species Distribution Model

Basis (narrative): Multiple collection sites are recorded for this species. Therefore, it was possible to perform species distribution modelling to predict its potential range with confidence limits. See Methods for details.

Min Elevation/Depth (m): 156

Max Elevation/Depth (m): 1500

Range description: This species has a wide distribution encompassing most of Slovenia and northern Croatia. It has also been recorded in the southern part of Carinthia (southern Austria) and in the area of Trieste in Friuli-Venezia Giulia (north-eastern Italy) (detailed occurrences and relative references in Suppl. material 67).

#### Extent of occurrence

EOO (km2): 26045-37659,31029

Trend: Stable

Justification for trend: *Troglohyphantesexcavatus* has been recorded in a wide range of habitats, both hypogean and epigean. It is plausible that anthropogenic climate change may affect the habitat suitability of this species. However, in view of the relatively wide thermal tolerance and the relatively high dispersal ability of non-specialised species of *Troglohyphantes* (Mammola et al. 2019a), the distribution range of *T.excavatus* is not expected to undergo significant reduction in the near future. A deeper study on the impacts of climate change on this species is required.

Causes ceased?: Yes

Causes understood?: Yes

Causes reversible?: Yes

Extreme fluctuations?: No

#### Area of occupancy

Trend: Stable

Justification for trend: *Troglohyphantesexcavatus* has been recorded in a wide range of habitats, both hypogean and epigean. It is plausible that anthropogenic climate change may affect the habitat suitability of this species. However, in view of the relatively wide thermal tolerance and the relatively high dispersal ability of non-specialised species of *Troglohyphantes* (Mammola et al. 2019a), the distribution range of *T.excavatus* is not expected to undergo significant reduction in the near future. A deeper study on the impacts of climate change on this species is required.

Causes ceased?: Yes

Causes understood?: Yes

Causes reversible?: Yes

Extreme fluctuations?: No

AOO (km2): 11712-21912,16100

#### Locations

Number of locations: Not applicable

Justification for number of locations: There are no currently known threats to this species.

Trend: Stable

Extreme fluctuations?: No

#### Population

Number of individuals: Unknown

Trend: Stable

Justification for trend: There are no currently known threats to the species.

Causes ceased?: Yes

Causes understood?: Yes

Causes reversible?: Yes

Extreme fluctuations?: No

#### Subpopulations

Number of subpopulations: Unknown

Trend: Unknown

Extreme fluctuations?: No

Severe fragmentation?: No

#### Habitat

System: Terrestrial

Habitat specialist: No

Habitat (narrative): In Slovenia, Croatia, and Italy specimens have been mainly collected in subterranean environments, both in deep caves and close to cave entrance, but also in shallow subterranean habitats, under big stones, usually in forests. On the contrary, in Austria this species has been recorded from surface localities, in moss and meadows, in spruce or mixed forests and on stream banks in forests.

Trend in extent, area or quality?: Stable

Justification for trend: The habitats colonised by *T.excavatus* are as yet not threatened by direct human activities.

##### Habitat

Habitat importance: Major Importance

Habitats: 1.4. Forest - Temperate5.1. Wetlands (inland) - Permanent Rivers/Streams/Creeks (includes waterfalls)7. Caves and Subterranean Habitats (non-aquatic)

#### Ecology

Size: 2.6 mm

Generation length (yr): 2

Dependency of single sp?: No

Ecology and traits (narrative): This spider shows minor morphological specialisation to subterranean life (Mammola et al. 2022a). Specimens display high variability in body colouration, ranging from sooty black to pale yellow-orange (Deeleman-Reinhold 1978).

#### Threats

Justification for threats: The existence of threats is unknown for this species.

##### Threats

Threat type: Past

Threats: 12. Other options - Other threat

#### Conservation

Justification for conservation actions: The potential distribution of *T.excavatus* is covered by several national parks, protected areas, and sites of the Natura 2000 network. In Austria, this species is listed in the category R of the Red List of endangered spiders for Carinthia (Komposch and Steinberger 1999). In addition, this species figures in the 59th Regulation of the Carinthian State Government of 2015 (LGBl. Nr. 59/2015) and it is fully protected from capture, collection, killing, and disturbance according to the Carinthian Nature Conservation Act 2002 (LGBl. Nr. 79/2002).

##### Conservation actions

Conservation action type: In Place

Conservation actions: 1.1. Land/water protection - Site/area protection1.2. Land/water protection - Resource & habitat protection2.1. Land/water management - Site/area management

#### Other

##### Use and trade

Use type: International

##### Ecosystem services

Ecosystem service type: Important

##### Research needed

Research needed: 1.2. Research - Population size, distribution & trends1.3. Research - Life history & ecology1.5. Research - Threats

Justification for research needed: Research on basic information such as distribution, ecology, life cycle and possible threats throughout the range would be needed.

### Troglohyphantes exul

#### Species information

Scientific name: Troglohyphantesexul

Species authority: Thaler, 1987

Kingdom: Animalia

Phylum: Arthropoda

Class: Arachnida

Order: Araneae

Family: Linyphiidae

Region for assessment: Global

#### Editor & Reviewers

##### Reviewers

Reviewers: Marc MilnePaulo Borges

##### Editor

Editor: Pedro Cardoso

#### Geographic range

Biogeographic realm: Palearctic

Countries: Italy

Map of records (Google Earth): Suppl. material 18

Basis of EOO and AOO: Observed

Basis (narrative): This spider is known exclusively from the type locality, where it has been repeatedly collected. In light of the high subterranean specialisation of this species and in view of the intensive sampling conducted in other caves in the same area, it is hardly expected that the range could be significantly expanded by new findings.

Min Elevation/Depth (m): 442

Max Elevation/Depth (m): 442

Range description: This species is known only from Cava di Sant'Ambrogio di Valpolicella, in the Lessini Mountains (province of Verona, Veneto, north-eastern Italy) (see Suppl. material 67).

#### Extent of occurrence

EOO (km2): 4

Trend: Decline (inferred)

Justification for trend: The single cave where this species was found is threatened by quarrying activities, which are expected to cause changes in microclimatic conditions and decrease of habitat quality. In addition, according to Mammola et al. (2018a), climate change will significantly affect the distribution of subterranean specialised *Troglohyphantes* in the future. Moreover, given the low tolerance to habitat changes of these species as well as their very low dispersal ability, a possible extreme reduction of the geographic range is expected in the future.

Causes ceased?: No

Causes understood?: Yes

Causes reversible?: No

Extreme fluctuations?: No

#### Area of occupancy

Trend: Decline (inferred)

Justification for trend: The single cave where this species was found is threatened by quarrying activities, which are expected to cause changes in microclimatic conditions and decrease of habitat quality. In addition, according to Mammola et al. (2018a), climate change will significantly affect the distribution of subterranean specialised *Troglohyphantes* in the future. Moreover, given the low tolerance to habitat changes of these species as well as their very low dispersal ability, a possible extreme reduction of the geographic range is expected in the future.

Causes ceased?: No

Causes understood?: Yes

Causes reversible?: No

Extreme fluctuations?: No

AOO (km2): 4

#### Locations

Number of locations: 1

Justification for number of locations: This species is known only from a single location, currently subjected to quarrying activities, which are likely to represent a major threat to the survival of the species. Moreover, the cave where this species occurs is exposed to possible changes in subterranean microclimatic conditions due to both quarrying activities and future climate change (see Mammola et al. 2018a).

Trend: Stable

Extreme fluctuations?: No

#### Population

Number of individuals: Unknown

Trend: Decline (inferred)

Justification for trend: In view of the reduced thermal tolerance of highly subterranean adapted *Troglohyphantes* (Mammola et al. 2019a), alterations of the microclimatic conditions of the habitat due to quarrying activities and due to climate change are expected to impact the whole population of this species.

Basis for decline: (c) a decline in area of occupancy, extent of occurrence and/or quality of habitat

Causes ceased?: No

Causes understood?: Yes

Causes reversible?: No

Extreme fluctuations?: No

Population Information (Narrative): No estimates of population size exist.

#### Subpopulations

Number of subpopulations: 1

Trend: Stable

Justification for trend: For this species we identified a single subpopulation.

Extreme fluctuations?: No

Severe fragmentation?: No

#### Habitat

System: Terrestrial

Habitat specialist: Yes

Habitat (narrative): This species has been found in an artificial subterranean habitat. Specimens have been collected in areas of complete darkness, were they are rather abundant.

Trend in extent, area or quality?: Decline (inferred)

Justification for trend: The nearby quarrying activities may cause environmental alterations of the cave where this species occurs. In addition, as seen in Mammola et al. (2018a) for the western alpine Troglohyphantes species, a drastic decline in the habitat suitability of *T.exul* as a consequence of climate change is expected.

##### Habitat

Habitat importance: Major Importance

Habitats: 7. Caves and Subterranean Habitats (non-aquatic)

#### Ecology

Size: 3 mm

Generation length (yr): 4

Dependency of single sp?: No

Ecology and traits (narrative): This species shows a high degree of specialisation to deep subterranean habitats, with absence of pigmentation and regression of the eyes (Mammola et al. 2022a).

#### Threats

Justification for threats: This species is potentially exposed due to its extremely narrow geographic distribution range and its presumably low dispersal capacity. Given the low tolerance to habitat changes of this subterranean organism, changes in the internal microclimate caused by the quarrying activities in the area of Cava di Sant'Ambrogio di Valpolicella may interfere with the species’ survival. Moreover, as seen for the species of the genus *Troglohyphantes* of the Western Alps (Mammola et al. 2018a), climate warming is expected to reduce the currently suitable habitat for this spider.

##### Threats

Threat type: Ongoing

Threats: 3.2. Energy production & mining - Mining & quarrying

##### Threats

Threat type: Future

Threats: 11.1. Climate change & severe weather - Habitat shifting & alteration11.2. Climate change & severe weather - Droughts11.3. Climate change & severe weather - Temperature extremes

#### Conservation

Justification for conservation actions: Cava di Sant'Ambrogio di Valpolicella falls within the Special Area of Conservation of Monte Pastello (SAC IT3210021). However, there are no conservation measures in place for this species. This species may benefit from effective protection with strategies aiming to reduce the environmental impacts of the nearby quarrying activities.

##### Conservation actions

Conservation action type: In Place

Conservation actions: 1.1. Land/water protection - Site/area protection1.2. Land/water protection - Resource & habitat protection

##### Conservation actions

Conservation action type: Needed

Conservation actions: 2.1. Land/water management - Site/area management

#### Other

##### Use and trade

Use type: International

##### Ecosystem services

Ecosystem service type: Important

##### Research needed

Research needed: 1.2. Research - Population size, distribution & trends1.3. Research - Life history & ecology1.5. Research - Threats3.1. Monitoring - Population trends3.4. Monitoring - Habitat trends

Justification for research needed: Research on basic information such as distribution, ecology, life cycle and possible threats throughout the range would be needed. Monitoring of population and habitat are important to confirm future trends and to assess the impacts of the nearby quarrying activities.

### Troglohyphantes fagei

#### Species information

Scientific name: Troglohyphantesfagei

Species authority: Roewer, 1931

Kingdom: Animalia

Phylum: Arthropoda

Class: Arachnida

Order: Araneae

Family: Linyphiidae

Region for assessment: Global

#### Editor & Reviewers

##### Reviewers

Reviewers: Marc MilnePaulo Borges

##### Editor

Editor: Pedro Cardoso

#### Geographic range

Biogeographic realm: Palearctic

Countries: GermanyAustriaItaly

Map of records (Google Earth): Suppl. material 19

Basis of EOO and AOO: Species Distribution Model

Basis (narrative): Multiple collection sites are recorded for this species. Therefore, it was possible to perform species distribution modelling to predict its potential range with confidence limits. See Methods for details.

Min Elevation/Depth (m): 164

Max Elevation/Depth (m): 2550

Range description: *Troglohyphantesfagei* is one of the most widespread species of the genus *Troglohyphantes*, being widely distributed in the Eastern Alps, from north-eastern Italy to Austria, with a few records from southern Germany (detailed occurrences and relative references in Suppl. material 67).

#### Extent of occurrence

EOO (km2): 50319-69323,60398

Trend: Stable

Justification for trend: This species has been recorded both in hypogean and epigean habitats. It is plausible that anthropogenic climate change may affect the habitat suitability of this species. However, in view of the relatively wide thermal tolerance and the relatively high dispersal ability of non-specialised species of *Troglohyphantes* (Mammola et al. 2019a), the distribution range of *T.fagei* is not expected to undergo significant reduction in the near future. A deeper study on the impacts of climate change on this species is required.

Causes ceased?: Yes

Causes understood?: Yes

Causes reversible?: Yes

Extreme fluctuations?: No

#### Area of occupancy

Trend: Stable

Justification for trend: This species has been recorded both in hypogean and epigean habitats. It is plausible that anthropogenic climate change may affect the habitat suitability of this species. However, in view of the relatively wide thermal tolerance and the relatively high dispersal ability of non-specialised species of *Troglohyphantes* (Mammola et al. 2019a), the distribution range of *T.fagei* is not expected to undergo significant reduction in the near future. A deeper study on the impacts of climate change on this species is required.

Causes ceased?: Yes

Causes understood?: Yes

Causes reversible?: Yes

Extreme fluctuations?: No

AOO (km2): 15012-35880,24896

#### Locations

Number of locations: Not applicable

Justification for number of locations: There are no currently known threats to this species.

Trend: Stable

Extreme fluctuations?: No

#### Population

Number of individuals: Unknown

Trend: Stable

Justification for trend: There are no currently known threats to the species.

Causes ceased?: Yes

Causes understood?: Yes

Causes reversible?: Yes

Extreme fluctuations?: No

#### Subpopulations

Number of subpopulations: Unknown

Trend: Unknown

Extreme fluctuations?: No

Severe fragmentation?: No

#### Habitat

System: Terrestrial

Habitat specialist: No

Habitat (narrative): At lower latitudes, this species was mainly collected in caves. At higher latitudes, it has been mainly found in alpine environments above the tree line, such as in rock crevices in alpine screes, in alpine grasslands, and in dwarf mountain pine shrubs.

Trend in extent, area or quality?: Stable

Justification for trend: The habitats colonised by *T.fagei* are as yet not threatened by direct human activities.

##### Habitat

Habitat importance: Major Importance

Habitats: 1.4. Forest - Temperate3.4. Shrubland - Temperate4.4. Grassland - Temperate6. Rocky areas (e.g. inland cliffs, mountain peaks)7. Caves and Subterranean Habitats (non-aquatic)

#### Ecology

Size: 2.8 mm

Generation length (yr): 2

Dependency of single sp?: No

Ecology and traits (narrative): This species shows a minor morphological specialisation to subterranean life (Mammola et al. 2022a). Specimens found in Bus de le Fade cave (north-eastern Italy) are characterised by pronounced microphthalmy (Isaia et al. 2017).

#### Threats

Justification for threats: The existence of major threats is unknown for this species.

##### Threats

Threat type: Past

Threats: 12. Other options - Other threat

#### Conservation

Justification for conservation actions: The potential distribution of this species is covered by several protected areas and sites of the Natura 2000 network. In Germany, due to its extreme rarity in the country, *T.fagei* was listed in the category R of the national Red List of spiders (Blick et al. 2016) and in the regional Red List of Bavaria (Blick and Scheidler 2004). In Austria, this species figures in the 59th Regulation of the Carinthian State Government of 2015 (LGBl. Nr. 59/2015), and it is fully protected from capture, collection, killing, and disturbance according to the Carinthian Nature Conservation Act 2002 (LGBl. Nr. 79/2002).

##### Conservation actions

Conservation action type: In Place

Conservation actions: 1.1. Land/water protection - Site/area protection1.2. Land/water protection - Resource & habitat protection2.1. Land/water management - Site/area management

#### Other

##### Use and trade

Use type: International

##### Ecosystem services

Ecosystem service type: Important

##### Research needed

Research needed: 1.2. Research - Population size, distribution & trends1.3. Research - Life history & ecology1.5. Research - Threats

Justification for research needed: Research on basic information such as distribution, ecology, life cycle and possible threats throughout the range would be needed.

### Troglohyphantes fatalis

#### Species information

Scientific name: Troglohyphantesfatalis

Species authority: Pesarini, 1988

Kingdom: Animalia

Phylum: Arthropoda

Class: Arachnida

Order: Araneae

Family: Linyphiidae

Region for assessment: Global

#### Editor & Reviewers

##### Reviewers

Reviewers: Marc MilnePaulo Borges

##### Editor

Editor: Pedro Cardoso

#### Geographic range

Biogeographic realm: Palearctic

Countries: Italy

Map of records (Google Earth): Suppl. material 20

Basis of EOO and AOO: Species Distribution Model

Basis (narrative): Multiple collection sites are recorded for this species. Therefore, it was possible to perform species distribution modelling to predict its potential range with confidence limits. See Methods for details.

Min Elevation/Depth (m): 76

Max Elevation/Depth (m): 1815

Range description: This species is known from a few localities in the Prealps of Belluno, in Colli Euganei, and Montello (Veneto, north-eastern Italy) (detailed occurrences and relative references in Suppl. material 67).

#### Extent of occurrence

EOO (km2): 3922-6572,5693

Trend: Stable

Justification for trend: The species has been collected both in epigean and hypogean environments. It is plausible that anthropogenic climate change may affect the habitat suitability of this species. However, in view of the relatively wide thermal tolerance and the relatively high dispersal ability of non-specialised species of *Troglohyphantes* (Mammola et al. 2019a), the distribution range of *T.fatalis* is not expected to undergo significant reduction in the near future. A deeper study on the impacts of climate change on this species is required.

Causes ceased?: Yes

Causes understood?: Yes

Causes reversible?: Yes

Extreme fluctuations?: No

#### Area of occupancy

Trend: Stable

Justification for trend: The species has been collected both in epigean and hypogean environments. It is plausible that anthropogenic climate change may affect the habitat suitability of this species. However, in view of the relatively wide thermal tolerance and the relatively high dispersal ability of non-specialised species of *Troglohyphantes* (Mammola et al. 2019a), the distribution range of *T.fatalis* is not expected to undergo significant reduction in the near future. A deeper study on the impacts of climate change on this species is required.

Causes ceased?: Yes

Causes understood?: Yes

Causes reversible?: Yes

Extreme fluctuations?: No

AOO (km2): 1532-3452,2732

#### Locations

Number of locations: Not applicable

Justification for number of locations: No known threats to this species.

Trend: Stable

Extreme fluctuations?: No

#### Population

Number of individuals: Unknown

Trend: Stable

Justification for trend: There are no currently known threats to the species.

Causes ceased?: Yes

Causes understood?: Yes

Causes reversible?: Yes

Extreme fluctuations?: No

#### Subpopulations

Number of subpopulations: Unknown

Trend: Unknown

Extreme fluctuations?: No

Severe fragmentation?: No

#### Habitat

System: Terrestrial

Habitat specialist: No

Habitat (narrative): *Troglohyphantesfatalis* has been found both in natural and artificial hypogean environments. Specimens have been collected also in surface habitats, such as damp and chestnut woods.

Trend in extent, area or quality?: Stable

Justification for trend: The habitats colonised by *T.fatalis* are as yet not threatened by direct human activities.

##### Habitat

Habitat importance: Major Importance

Habitats: 1.4. Forest - Temperate7. Caves and Subterranean Habitats (non-aquatic)

#### Ecology

Size: 2.9 mm

Generation length (yr): 2

Dependency of single sp?: No

Ecology and traits (narrative): The ecology of this species is unknown.

#### Threats

Justification for threats: The existence of direct threats is unknown for this species.

##### Threats

Threat type: Past

Threats: 12. Other options - Other threat

#### Conservation

Justification for conservation actions: There are several protected areas within the distribution range of this species (EUAP0243 Parco Regionale dei Colli Euganei and SAC/SPA IT3260017 Colli Euganei - Monte Lozzo - Monte Ricco, SAC/SPA IT3230022 Massiccio del Grappa, SAC/SPA IT3220036 Altopiano dei Sette Comuni, SAC/SPA IT3230090 Cima Campo - Monte Celado, SAC IT3240004 Montello). Species distribution modelling predicts that this species could be present in more protected areas.

##### Conservation actions

Conservation action type: In Place

Conservation actions: 1.1. Land/water protection - Site/area protection1.2. Land/water protection - Resource & habitat protection2.1. Land/water management - Site/area management

#### Other

##### Use and trade

Use type: International

##### Ecosystem services

Ecosystem service type: Important

##### Research needed

Research needed: 1.2. Research - Population size, distribution & trends1.3. Research - Life history & ecology1.5. Research - Threats

Justification for research needed: Research on basic information such as distribution, natural history, ecology and possible threats of the species would be needed.

### Troglohyphantes gamsi

#### Species information

Scientific name: Troglohyphantesgamsi

Species authority: Deeleman-Reinhold, 1978

Kingdom: Animalia

Phylum: Arthropoda

Class: Arachnida

Order: Araneae

Family: Linyphiidae

Region for assessment: Global

#### Editor & Reviewers

##### Reviewers

Reviewers: Marc MilnePaulo Borges

##### Editor

Editor: Pedro Cardoso

#### Geographic range

Biogeographic realm: Palearctic

Countries: Slovenia

Map of records (Google Earth): Suppl. material 21

Basis of EOO and AOO: Observed

Basis (narrative): This species was collected in very few localities. Its low level of subterranean specialisation, possibly reflects a higher dispersal capacity when compared to subterranean specialised species. Consequently, it may be possible that the present known range of this species is underestimated.

Min Elevation/Depth (m): 532

Max Elevation/Depth (m): 988

Range description: This species was recorded only in two localities, in north-western Slovenia: Kristalna jama (Bled) in Upper Carniola, and the tunnel 'Kluža' near Bovec in Slovenian Littoral (see Suppl. material 67).

#### Extent of occurrence

EOO (km2): 8

Trend: Unknown

Causes ceased?: Unknown

Causes understood?: Unknown

Causes reversible?: Unknown

Extreme fluctuations?: No

#### Area of occupancy

Trend: Unknown

Causes ceased?: Unknown

Causes understood?: Unknown

Causes reversible?: Unknown

Extreme fluctuations?: No

AOO (km2): 8

#### Locations

Number of locations: Unknown

Justification for number of locations: The data available are not enough to estimate the number of locations for this non-specialised species.

Trend: Unknown

Extreme fluctuations?: No

#### Population

Number of individuals: Unknown

Trend: Unknown

Justification for trend: The population size and trend are unknown.

Causes ceased?: Unknown

Causes understood?: Unknown

Causes reversible?: Unknown

Extreme fluctuations?: No

#### Subpopulations

Number of subpopulations: Unknown

Trend: Unknown

Extreme fluctuations?: No

Severe fragmentation?: No

#### Habitat

System: Terrestrial

Habitat specialist: No

Habitat (narrative): In the type locality (Kristalna jama), specimens were collected only in the most superficial parts of the cave, among stones and wood debris, in the descending entrance passage.

Trend in extent, area or quality?: Stable

##### Habitat

Habitat importance: Major Importance

Habitats: 7. Caves and Subterranean Habitats (non-aquatic)

#### Ecology

Size: 2.5 mm

Generation length (yr): 2

Dependency of single sp?: No

Ecology and traits (narrative): *Troglohyphantesgamsi* is one of the most pigmented *Troglohyphantes* (Deeleman-Reinhold 1978). This spider shows minor morphological specialisation to subterranean life (Mammola et al. 2022a).

#### Threats

Justification for threats: Unknown threats.

##### Threats

Threat type: Past

Threats: 12. Other options - Other threat

#### Conservation

Justification for conservation actions: Due to its rarity, *T.gamsi* is considered as potentially threatened and included in the category R of the Slovenian Red List of endangered plant and animal species (Uradni list RS št. 82/02 in 42/10). One of the known localities of this species falls within the Natura 2000 network (SAC SI3000253 Julijske Alps, SPA SI5000019 Julijci).

##### Conservation actions

Conservation action type: In Place

Conservation actions: 1.1. Land/water protection - Site/area protection1.2. Land/water protection - Resource & habitat protection2.1. Land/water management - Site/area management

#### Other

##### Use and trade

Use type: International

##### Ecosystem services

Ecosystem service type: Important

##### Research needed

Research needed: 1.2. Research - Population size, distribution & trends1.3. Research - Life history & ecology1.5. Research - Threats

Justification for research needed: Research on basic information such as distribution, natural history, ecology and possible threats of the species would be needed.

### Troglohyphantes gestroi

#### Species information

Scientific name: Troglohyphantesgestroi

Species authority: Fage, 1933

Kingdom: Animalia

Phylum: Arthropoda

Class: Arachnida

Order: Araneae

Family: Linyphiidae

Region for assessment: Global

#### Editor & Reviewers

##### Reviewers

Reviewers: Marc MilnePaulo Borges

##### Editor

Editor: Pedro Cardoso

#### Geographic range

Biogeographic realm: Palearctic

Countries: Italy

Map of records (Google Earth): Suppl. material 22

Basis of EOO and AOO: Observed

Basis (narrative): Despite the relatively high number of records of this non-specialised species, the distribution range predicted by the models was found to be unreliable by our own expert opinion. In view of this, only the observed distribution range is presented.

Min Elevation/Depth (m): 134

Max Elevation/Depth (m): 1878

Range description: This species is very common in the caves of the Brescia Prealps (Lombardia, northern Italy) while in the province of Bergamo its distribution is confined to the Sebino area and to the high valley of Scalve (detailed occurrences and relative references in Suppl. material 67).

#### Extent of occurrence

EOO (km2): 2380

Trend: Stable

Justification for trend: *Troglohyphantesgestroi* has been collected both in surface and subterranean environments. It is plausible that anthropogenic climate change may affect the habitat suitability of this species. However, in view of the relatively wide thermal tolerance and the relatively high dispersal ability of non-specialised *Troglohyphantes* (Mammola et al. 2019a), the distribution range of *T.gestroi* is not expected to undergo significant reduction in the near future. A deeper study on the current distribution of this species and on the potential impacts of climate change is required.

Causes ceased?: Yes

Causes understood?: Yes

Causes reversible?: Yes

Extreme fluctuations?: No

#### Area of occupancy

Trend: Stable

Justification for trend: *Troglohyphantesgestroi* has been collected both in surface and subterranean environments. It is plausible that anthropogenic climate change may affect the habitat suitability of this species. However, in view of the relatively wide thermal tolerance and the relatively high dispersal ability of non-specialised *Troglohyphantes* (Mammola et al. 2019a), the distribution range of *T.gestroi* is not expected to undergo significant reduction in the near future. A deeper study on the current distribution of this species and on the potential impacts of climate change is required.

Causes ceased?: Yes

Causes understood?: Yes

Causes reversible?: Yes

Extreme fluctuations?: No

AOO (km2): 140

#### Locations

Number of locations: Not applicable

Justification for number of locations: There are no currently known threats to this species.

Trend: Stable

Extreme fluctuations?: No

#### Population

Number of individuals: Unknown

Trend: Stable

Justification for trend: There are no currently known threats to the species.

Causes ceased?: Yes

Causes understood?: Yes

Causes reversible?: Yes

Extreme fluctuations?: No

#### Subpopulations

Number of subpopulations: Unknown

Trend: Unknown

Extreme fluctuations?: No

Severe fragmentation?: No

#### Habitat

System: Terrestrial

Habitat specialist: No

Habitat (narrative): This species has been mainly collected in natural and artificial caves, but it also occurs in epigean environments such as fir woods and open habitats.

Trend in extent, area or quality?: Stable

Justification for trend: The habitats colonised by *T.gestroi* are as yet not threatened by direct human activities.

##### Habitat

Habitat importance: Major Importance

Habitats: 1.4. Forest - Temperate7. Caves and Subterranean Habitats (non-aquatic)

#### Ecology

Size: 4.1 mm

Generation length (yr): 2

Dependency of single sp?: No

Ecology and traits (narrative): Not much is known about the ecology and life history of this species. This spider shows minor morphological specialisation to subterranean life (Mammola et al. 2022a).

#### Threats

Justification for threats: This species is potentially exposed due to its limited geographic distribution range. However, the existence of direct threats is unknown for this species.

##### Threats

Threat type: Past

Threats: 12. Other options - Other threat

#### Conservation

Justification for conservation actions: Part of the distribution of this species falls within several protected areas (SAC IT2060004 Alta Val di Scalve, SPA IT2060401 Parco Regionale Orobie Bergamasche, SAC IT2070018 Altopiano di Cariadeghe).

##### Conservation actions

Conservation action type: In Place

Conservation actions: 1.1. Land/water protection - Site/area protection1.2. Land/water protection - Resource & habitat protection2.1. Land/water management - Site/area management

#### Other

##### Use and trade

Use type: International

##### Ecosystem services

Ecosystem service type: Important

##### Research needed

Research needed: 1.2. Research - Population size, distribution & trends1.3. Research - Life history & ecology1.5. Research - Threats

Justification for research needed: Research on basic information such as distribution, natural history, ecology and possible threats of the species would be needed.

### Troglohyphantes giachinoi

#### Species information

Scientific name: Troglohyphantesgiachinoi

Species authority: Isaia & Mammola, 2018

Kingdom: Animalia

Phylum: Arthropoda

Class: Arachnida

Order: Araneae

Family: Linyphiidae

Region for assessment: Global

#### Editor & Reviewers

##### Reviewers

Reviewers: Marc MilnePaulo Borges

##### Editor

Editor: Pedro Cardoso

#### Geographic range

Biogeographic realm: Palearctic

Countries: Italy

Map of records (Google Earth): Suppl. material 23

Basis of EOO and AOO: Observed

Basis (narrative): There are only two records known for this spider, both referring to the MSS (Milieu Souterrain Superficiel, see Mammola et al. 2016) habitat. It may be possible that the species occurs in other localities in the area. The known distribution range should be taken with caution.

Min Elevation/Depth (m): 839

Max Elevation/Depth (m): 1332

Range description: This species is known only from MSS habitat in the area of the hypogean complex of the Pugnetto caves, in the municipality of Mezzenile, and from the Natural Park of Colle del Lys (Province of Torino, Piemonte, north-western Italy) (see Suppl. material 67).

#### Extent of occurrence

EOO (km2): 8

Trend: Decline (inferred)

Justification for trend: According to Mammola et al. (2018a), climate change will significantly affect the distribution of subterranean specialised *Troglohyphantes* in the future. Moreover, given the low tolerance to habitat changes of these species as well as their very low dispersal ability, a possible extreme reduction of the geographic range is expected in the future.

Causes ceased?: No

Causes understood?: Yes

Causes reversible?: No

Extreme fluctuations?: No

#### Area of occupancy

Trend: Decline (inferred)

Justification for trend: According to Mammola et al. (2018a), climate change will significantly affect the distribution of subterranean specialised *Troglohyphantes* in the future. Moreover, given the low tolerance to habitat changes of these species as well as their very low dispersal ability, a possible extreme reduction of the geographic range is expected in the future.

Causes ceased?: No

Causes understood?: Yes

Causes reversible?: No

Extreme fluctuations?: No

AOO (km2): 8

#### Locations

Number of locations: 1

Justification for number of locations: This species has been recorded in two localities, which are interpreted as a single location as they are both affected by changes in subterranean microclimatic conditions due to climate change.

Trend: Stable

Extreme fluctuations?: No

#### Population

Number of individuals: Unknown

Trend: Decline (inferred)

Justification for trend: In view of the reduced thermal tolerance of subterranean specialised *Troglohyphantes* species (Mammola et al. 2019a), alterations of the microclimatic conditions of the habitat due to climate change are expected to impact the whole population of this species.

Basis for decline: (c) a decline in area of occupancy, extent of occurrence and/or quality of habitat

Causes ceased?: No

Causes understood?: Yes

Causes reversible?: No

Extreme fluctuations?: No

#### Subpopulations

Number of subpopulations: 2

Trend: Decline (inferred)

Justification for trend: Because of the adaptation to the subterranean habitat and the possible consequent narrow thermal tolerance of this species, likely hampering dispersal through non-subterranean habitats, each occurrence reasonably represents a single isolated subpopulation. Accordingly, for this species we identified two subpopulations: the first one occurring in the Pugnetto hypogean complex, in the Lanzo Valleys (Northwestern Alps), and the second subpopulation in the Natural Park of Colle del Lys, an alpine pass located between the lower Valle di Susa and Valle di Viù (Graian Alps). These subpopulations are likely to be impacted by climate change.

Extreme fluctuations?: No

Severe fragmentation?: No

#### Habitat

System: Terrestrial

Habitat specialist: Yes

Habitat (narrative): Specimens of *T.giachinoi* were collected in MSS habitat, using subterranean sampling devices installed at depths between 0.40 and 0.80 m and pitfall traps placed in deep beech forest leaf litter [see Mammola et al. (2017) for more details on the habitat]. All the traps were installed at an altitude between 800 and 870 m. Material from the Natural Park of Colle del Lys, was collected by Konrad Thaler in 1972–1973 in unspecified habitat (see Mammola et al. 2018c).

Trend in extent, area or quality?: Decline (inferred)

Justification for trend: MSS habitats, like other superficial subterranean habitats, are likely to be affected by the global temperature increase. It is expected that the temperature increase in superficial subterranean habitats will parallel the external one almost synchronically. Compared with the deep subterranean sectors, effects on the fauna in superficial subterranean habitats are expected to be more immediate (Mammola et al. 2019b). Accordingly, a reduction in the future geographic distribution range of *T.giachinoi* is expected.

##### Habitat

Habitat importance: Major Importance

Habitats: 7.2. Caves and Subterranean Habitats (non-aquatic) - Other Subterranean Habitats

#### Ecology

Size: 3 mm

Generation length (yr): 4

Dependency of single sp?: No

Ecology and traits (narrative): *Troglohyphantesgiachinoi* is among the smaller species of alpine *Troglohyphantes*, and its short legs and overall small body size may reflect a specialisation for inhabiting small habitat pores, such as the air-filled spaces in the MSS, deep leaf litter, and soil strata. Although this species co-exists with other two *Troglohyphantes* (*T.bornensis* and *T.lucifuga*) in the same hypogean complex, they exploit different habitats (Mammola et al. 2018c).

#### Threats

Justification for threats: This species is potentially exposed due to its extremely narrow geographic distribution range and its low dispersal capacity. Superficial network of underground fissures where *T.giachinoi* occurs, are likely to be affected by the global temperature increase, which is expected to impact the current suitability of this adapted species. Compared with the deep subterranean sectors, effects of temperature increase on the shallow subterranean fauna are expected to be more immediate (Mammola et al. 2019b).

##### Threats

Threat type: Future

Threats: 11.1. Climate change & severe weather - Habitat shifting & alteration11.2. Climate change & severe weather - Droughts11.3. Climate change & severe weather - Temperature extremes

#### Conservation

Justification for conservation actions: The distribution range of this species is included in a Special Area of Conservation (SAC IT1110048 Grotte del Pugnetto) and in a Regional Park (EUAP0883 Parco Naturale del Colle del Lys).

##### Conservation actions

Conservation action type: In Place

Conservation actions: 1.1. Land/water protection - Site/area protection1.2. Land/water protection - Resource & habitat protection2.1. Land/water management - Site/area management

#### Other

##### Use and trade

Use type: International

##### Ecosystem services

Ecosystem service type: Important

##### Research needed

Research needed: 1.2. Research - Population size, distribution & trends1.3. Research - Life history & ecology1.5. Research - Threats

Justification for research needed: Research on basic information such as distribution, natural history, ecology and possible threats of the species would be needed.

### Troglohyphantes gracilis

#### Species information

Scientific name: Troglohyphantesgracilis

Species authority: Fage, 1919

Kingdom: Animalia

Phylum: Arthropoda

Class: Arachnida

Order: Araneae

Family: Linyphiidae

Region for assessment: Global

#### Editor & Reviewers

##### Reviewers

Reviewers: Marc MilnePaulo Borges

##### Editor

Editor: Pedro Cardoso

#### Geographic range

Biogeographic realm: Palearctic

Countries: Slovenia

Map of records (Google Earth): Suppl. material 24

Basis of EOO and AOO: Observed

Basis (narrative): In light of its high level of subterranean specialisation, we assume that the known records of *T.gracilis* are good proxies for defining the AOO and EOO of this species.

Min Elevation/Depth (m): 345

Max Elevation/Depth (m): 577

Range description: This species has been collected in three caves (Podpeška jama, Pasjica pri Predolah, and Jama pod cesto) in Lower Carniola (Central Slovenia) (detailed occurrences and relative references in Suppl. material 67).

#### Extent of occurrence

EOO (km2): 136

Trend: Decline (inferred)

Justification for trend: According to Mammola et al. (2018a), climate change will significantly affect the distribution of subterranean specialised *Troglohyphantes* in the future. Moreover, given the low tolerance to habitat changes of these species as well as their very low dispersal ability, a possible extreme reduction of the geographic range is expected in the future.

Causes ceased?: No

Causes understood?: Yes

Causes reversible?: No

Extreme fluctuations?: No

#### Area of occupancy

Trend: Decline (inferred)

Justification for trend: According to Mammola et al. (2018a), climate change will significantly affect the distribution of subterranean specialised *Troglohyphantes* in the future. Moreover, given the low tolerance to habitat changes of these species as well as their very low dispersal ability, a possible extreme reduction of the geographic range is expected in the future.

Causes ceased?: No

Causes understood?: Yes

Causes reversible?: No

Extreme fluctuations?: No

AOO (km2): 12

#### Locations

Number of locations: 1

Justification for number of locations: Even though this species occurs in three caves, these are interpreted as a single location, as they are all affected by changes in subterranean microclimatic conditions due to climate change.

Trend: Stable

Extreme fluctuations?: No

#### Population

Number of individuals: Unknown

Trend: Decline (inferred)

Justification for trend: In view of the reduced thermal tolerance of subterranean specialised *Troglohyphantes* (Mammola et al. 2019a), alterations of the microclimatic conditions of the habitat due to climate change are expected to impact the whole population of this species.

Basis for decline: (c) a decline in area of occupancy, extent of occurrence and/or quality of habitat

Causes ceased?: No

Causes understood?: Yes

Causes reversible?: No

Extreme fluctuations?: No

#### Subpopulations

Number of subpopulations: 3

Trend: Decline (inferred)

Justification for trend: Because of the adaptation to the subterranean habitat and of the narrow physiological tolerance of this species, likely hampering dispersal through non-subterranean environments, each locality can reasonably host a single isolated subpopulation. Accordingly, for this species we identified three different subpopulations, corresponding to the three caves where the species has been collected. These subpopulation are likely to be impacted by climate change.

Extreme fluctuations?: No

Severe fragmentation?: No

#### Habitat

System: Terrestrial

Habitat specialist: Yes

Habitat (narrative): There is poor information on the habitat of this species. Specimens have been collected in caves.

Trend in extent, area or quality?: Decline (inferred)

Justification for trend: As seen in Mammola et al. (2018a) for the western alpine species of *Troglohyphantes*, a drastic decline in the habitat suitability of *T.gracilis* as a consequence of climate change is also expected.

##### Habitat

Habitat importance: Major Importance

Habitats: 7. Caves and Subterranean Habitats (non-aquatic)

#### Ecology

Size: 3.3 mm

Generation length (yr): 4

Dependency of single sp?: No

Ecology and traits (narrative): This spider shows a high degree of specialisation to subterranean life, with absence of pigmentation (Mammola et al. 2022a).

#### Threats

Justification for threats: This species is potentially exposed due to its extremely narrow geographic distribution range and its presumably low dispersal capacity. As seen for the species of the genus *Troglohyphantes* of the Western Alps (Mammola et al. 2018a), climate warming is expected to reduce the currently suitable habitat for this spider.

##### Threats

Threat type: Future

Threats: 11.1. Climate change & severe weather - Habitat shifting & alteration11.2. Climate change & severe weather - Droughts11.3. Climate change & severe weather - Temperature extremes

#### Conservation

Justification for conservation actions: This species was listed in the first IUCN Red List (IUCN Conservation Monitoring Centre 1986) due to its restricted geographical distribution, and was assessed as Vulnerable in the 1996 IUCN Red List (World Conservation Monitoring Centre 1996a), but its status has not been updated since. In addition, this species figures in the Slovenian national Red List due to its rarity (category R) (Uradni list RS št. 82/02 in 42/10). The locus typicus is protected by the Natura 2000 network (SAC SI3000207 Podpeška jama) and it is not open to the public. Another cave where this species occurs is located in the Special Area of Conservation SAC SI3000232 Notranjski trikotnik, and in the Special Protection Area SPA SI5000002 Snežnik - Pivka.

##### Conservation actions

Conservation action type: In Place

Conservation actions: 1.1. Land/water protection - Site/area protection1.2. Land/water protection - Resource & habitat protection2.1. Land/water management - Site/area management

#### Other

##### Use and trade

Use type: International

##### Ecosystem services

Ecosystem service type: Important

##### Research needed

Research needed: 1.2. Research - Population size, distribution & trends1.3. Research - Life history & ecology1.5. Research - Threats3.1. Monitoring - Population trends3.4. Monitoring - Habitat trends

Justification for research needed: Research on basic information such as distribution, ecology, life cycle and possible threats throughout the range would be needed. Monitoring of population and habitat are important to confirm inferred trends.

### Troglohyphantes helsdingeni

#### Species information

Scientific name: Troglohyphanteshelsdingeni

Species authority: Deeleman-Reinhold, 1978

Kingdom: Animalia

Phylum: Arthropoda

Class: Arachnida

Order: Araneae

Family: Linyphiidae

Region for assessment: Global

#### Editor & Reviewers

##### Reviewers

Reviewers: Marc MilnePaulo Borges

##### Editor

Editor: Pedro Cardoso

#### Geographic range

Biogeographic realm: Palearctic

Countries: SloveniaAustria

Map of records (Google Earth): Suppl. material 25

Basis of EOO and AOO: Observed

Basis (narrative): This species was collected in three localities. Its low level of subterranean specialisation, possibly reflects a higher dispersal capacity when compared to subterranean specialised species. Consequently, it may be possible that the present known range of this species is underestimated.

Min Elevation/Depth (m): 480

Max Elevation/Depth (m): 480

Range description: This species is known from only two localities in Upper Carniola (Slovenia) and from another locality in Carinthia (southern Austria) (detailed occurrences and relative references in Suppl. material 67).

#### Extent of occurrence

EOO (km2): 128

Trend: Stable

Justification for trend: This species is not strictly relegated to deep subterranean habitats. It is plausible that anthropogenic climate change may affect the habitat suitability of this species. However, in view of the relatively wide thermal tolerance and the relatively high dispersal ability of non-specialised species of *Troglohyphantes* (Mammola et al. 2019a), the distribution range of *T.helsdingeni* is not expected to undergo significant reduction in the near future. A deeper study on the current distribution of this species and on the potential impacts of climate change is required.

Causes ceased?: Yes

Causes understood?: Yes

Causes reversible?: Yes

Extreme fluctuations?: No

#### Area of occupancy

Trend: Stable

Justification for trend: This species is not strictly relegated to deep subterranean habitats. It is plausible that anthropogenic climate change may affect the habitat suitability of this species. However, in view of the relatively wide thermal tolerance and the relatively high dispersal ability of non-specialised species of *Troglohyphantes* (Mammola et al. 2019a), the distribution range of *T.helsdingeni* is not expected to undergo significant reduction in the near future. A deeper study on the current distribution of this species and on the potential impacts of climate change is required.

Causes ceased?: Yes

Causes understood?: Yes

Causes reversible?: Yes

Extreme fluctuations?: No

AOO (km2): 12

#### Locations

Number of locations: Not applicable

Justification for number of locations: No known threats to this species.

Trend: Stable

Extreme fluctuations?: No

#### Population

Number of individuals: Unknown

Trend: Stable

Justification for trend: There are no currently known threats to the species.

Causes ceased?: Yes

Causes understood?: Yes

Causes reversible?: Yes

Extreme fluctuations?: No

#### Subpopulations

Number of subpopulations: Unknown

Trend: Unknown

Extreme fluctuations?: No

Severe fragmentation?: No

#### Habitat

System: Terrestrial

Habitat specialist: No

Habitat (narrative): This species was collected in small mammal burrows in mixed forest (*Fagus*-*Castaneus*). No additional information about the habitat was provided.

Trend in extent, area or quality?: Unknown

##### Habitat

Habitat importance: Major Importance

Habitats: 1.4. Forest - Temperate7.2. Caves and Subterranean Habitats (non-aquatic) - Other Subterranean Habitats

#### Ecology

Size: 2.3 mm

Generation length (yr): 2

Dependency of single sp?: No

Ecology and traits (narrative): This spider shows minor morphological adaptations to the subterranean life (Mammola et al. 2022a).

#### Threats

Justification for threats: The existence of threats is unknown for this species.

##### Threats

Threat type: Past

Threats: 12. Other options - Other threat

#### Conservation

Justification for conservation actions: Due to its rarity, *T.helsdingeni* was considered as potentially threatened and listed in the category R of the Slovenian Red List (Uradni list RS št. 82/02 in 42/10), and in the category G (generally threatened) of the Red List of endangered spiders for Carinthia (Komposch and Steinberger 1999). In addition, this species figures in the 59th Regulation of the Carinthian State Government of 2015 (LGBl. Nr. 59/2015), among the species that are fully protected from capture, collection, killing, and disturbance according to the Carinthian Nature Conservation Act 2002 (LGBl. Nr. 79/2002).

##### Conservation actions

Conservation action type: In Place

#### Other

##### Use and trade

Use type: International

##### Ecosystem services

Ecosystem service type: Important

##### Research needed

Research needed: 1.2. Research - Population size, distribution & trends1.3. Research - Life history & ecology1.5. Research - Threats

Justification for research needed: Research on basic information such as distribution, natural history, ecology and possible threats of the species would be needed.

### Troglohyphantes henroti

#### Species information

Scientific name: Troglohyphanteshenroti

Species authority: Dresco, 1956

Kingdom: Animalia

Phylum: Arthropoda

Class: Arachnida

Order: Araneae

Family: Linyphiidae

Region for assessment: Global

#### Editor & Reviewers

##### Reviewers

Reviewers: Marc MilnePaulo Borges

##### Editor

Editor: Pedro Cardoso

#### Geographic range

Biogeographic realm: Palearctic

Countries: France

Map of records (Google Earth): Suppl. material 26

Basis of EOO and AOO: Observed

Basis (narrative): This species has been found in a few localities. Due to the limited number of surveys carried out in this area, it seems likely that the species may occur elsewhere nearby. The known distribution range should be taken with caution.

Min Elevation/Depth (m): 1095

Max Elevation/Depth (m): 1280

Range description: This species is known from a few localities of the departments of Drôme and Isère (Auvergne-Rhône-Alpes, south-eastern France) (detailed occurrences and relative references in Suppl. material 67).

#### Extent of occurrence

EOO (km2): 19

Trend: Decline (inferred)

Justification for trend: According to Mammola et al. (2018a), climate change will significantly affect the distribution of subterranean specialised *Troglohyphantes* in the future. Moreover, given the low tolerance to habitat changes of these species as well as their very low dispersal ability, a possible extreme reduction of the geographic range is expected in the future.

Causes ceased?: No

Causes understood?: Yes

Causes reversible?: No

Extreme fluctuations?: No

#### Area of occupancy

Trend: Decline (inferred)

Justification for trend: According to Mammola et al. (2018a), climate change will significantly affect the distribution of subterranean specialised *Troglohyphantes* in the future. Moreover, given the low tolerance to habitat changes of these species as well as their very low dispersal ability, a possible extreme reduction of the geographic range is expected in the future.

Causes ceased?: No

Causes understood?: Yes

Causes reversible?: No

Extreme fluctuations?: No

AOO (km2): 16

#### Locations

Number of locations: 1

Justification for number of locations: Even though this species occurs in some caves, these are interpreted as a single location, as they are all affected by changes in subterranean microclimatic conditions due to climate change.

Trend: Stable

Extreme fluctuations?: No

#### Population

Number of individuals: Unknown

Trend: Decline (inferred)

Justification for trend: In view of the reduced thermal tolerance of subterranean specialised *Troglohyphantes* species (Mammola et al. 2019a), alterations of the microclimatic conditions of the habitat due to climate change are expected to impact the whole population of this species.

Basis for decline: (c) a decline in area of occupancy, extent of occurrence and/or quality of habitat

Causes ceased?: No

Causes understood?: Yes

Causes reversible?: No

Extreme fluctuations?: No

#### Subpopulations

Number of subpopulations: 2

Trend: Decline (inferred)

Justification for trend: Because of the adaptation to the subterranean habitat and the narrow thermal tolerance of this species, likely hampering dispersal through non-subterranean habitats, each subpopulation can reasonably occur in a single isolated or few contiguous localities. Accordingly, for this species we identified two subpopulations: a southern subpopulation in the commune of Bouvante, Drôme department, and a northern one occurring in a few caves in the commune of Presles, Isère department. Future climate change is expected to affect both of them.

Extreme fluctuations?: No

Severe fragmentation?: No

#### Habitat

System: Terrestrial

Habitat specialist: Yes

Habitat (narrative): *Troglohyphanteshenroti* has been collected in hypogean habitats. It has not been specified if in the deep portion or near the entrance, however in caves.

Trend in extent, area or quality?: Decline (inferred)

Justification for trend: As seen in Mammola et al. (2018a) for other species of the genus *Troglohyphantes* of the Western Alps, a drastic decline in the habitat suitability of *T.henroti* as a consequence of climate change is expected.

##### Habitat

Habitat importance: Major Importance

Habitats: 7. Caves and Subterranean Habitats (non-aquatic)

#### Ecology

Size: 4 mm

Generation length (yr): 4

Dependency of single sp?: No

Ecology and traits (narrative): Specimens show subterranean specialisation to the hypogean life, namely loss of pigmentation and reduction of the eye apparatus (Mammola et al. 2022a).

#### Threats

Justification for threats: This species is potentially exposed due to its extremely narrow geographic distribution range and its presumably low dispersal capacity. As seen for the *Troglohyphantes* species of the Western Alps (Mammola et al. 2018a), climate warming is expected to reduce the currently suitable habitat for this spider.

##### Threats

Threat type: Future

Threats: 11.1. Climate change & severe weather - Habitat shifting & alteration11.2. Climate change & severe weather - Droughts11.3. Climate change & severe weather - Temperature extremes

#### Other

##### Use and trade

Use type: International

##### Ecosystem services

Ecosystem service type: Important

##### Research needed

Research needed: 1.2. Research - Population size, distribution & trends1.3. Research - Life history & ecology1.5. Research - Threats

Justification for research needed: Research on basic information such as distribution, ecology, life cycle and possible threats throughout the range would be needed.

### Troglohyphantes iulianae

#### Species information

Scientific name: Troglohyphantesiulianae

Species authority: Brignoli, 1971

Kingdom: Animalia

Phylum: Arthropoda

Class: Arachnida

Order: Araneae

Family: Linyphiidae

Region for assessment: Global

#### Editor & Reviewers

##### Reviewers

Reviewers: Marc MilnePaulo Borges

##### Editor

Editor: Pedro Cardoso

#### Geographic range

Biogeographic realm: Palearctic

Countries: Italy

Map of records (Google Earth): Suppl. material 27

Basis of EOO and AOO: Observed

Basis (narrative): This subterranean specialised species was collected in a few localities spread in a relatively wide range. Consequently, it may be possible that the present known distribution range of this species is underestimated.

Min Elevation/Depth (m): 200

Max Elevation/Depth (m): 1380

Range description: This species has been recorded in a few collection sites spread in a wide distribution range, spanning from the Southwestern Alps to the Tuscanian Apennines (detailed occurrences and relative references in Suppl. material 67).

#### Extent of occurrence

EOO (km2): 8754

Trend: Decline (inferred)

Justification for trend: According to Mammola et al. (2018a), climate change will significantly affect the distribution of subterranean specialised *Troglohyphantes* in the future. Moreover, given the low tolerance to habitat changes of these species as well as their very low dispersal ability, a possible extreme reduction of the geographic range is expected in the future.

Causes ceased?: No

Causes understood?: Yes

Causes reversible?: No

Extreme fluctuations?: No

#### Area of occupancy

Trend: Decline (inferred)

Justification for trend: According to Mammola et al. (2018a), climate change will significantly affect the distribution of subterranean specialised *Troglohyphantes* in the future. Moreover, given the low tolerance to habitat changes of these species as well as their very low dispersal ability, a possible extreme reduction of the geographic range is expected in the future.

Causes ceased?: No

Causes understood?: Yes

Causes reversible?: No

Extreme fluctuations?: No

AOO (km2): 44

#### Locations

Number of locations: 1

Justification for number of locations: The habitat where this species occurs is affected by changes in subterranean microclimatic conditions due to climate change, which is expected to impact the whole population (see Mammola et al. 2018a).

Trend: Stable

Extreme fluctuations?: No

#### Population

Number of individuals: Unknown

Trend: Decline (inferred)

Justification for trend: In view of the reduced thermal tolerance of subterranean specialised *Troglohyphantes* species (Mammola et al. 2019a), alterations of the microclimatic conditions of the habitat due to climate change are expected to impact the whole population of this species.

Basis for decline: (c) a decline in area of occupancy, extent of occurrence and/or quality of habitat

Causes ceased?: No

Causes understood?: Yes

Causes reversible?: No

Extreme fluctuations?: No

#### Subpopulations

Number of subpopulations: Unknown

Trend: Unknown

Extreme fluctuations?: No

Severe fragmentation?: No

#### Habitat

System: Terrestrial

Habitat specialist: Yes

Habitat (narrative): This species was found both in caves and in shallow subterranean habitats. Specimens have been mainly collected close to the cave entrance, dwelling among debris. Cave openings are located in *Fagus* or *Castanea* woods.

Trend in extent, area or quality?: Decline (inferred)

##### Habitat

Habitat importance: Major Importance

Habitats: 1.4. Forest - Temperate7. Caves and Subterranean Habitats (non-aquatic)

#### Ecology

Size: 2.4 mm

Generation length (yr): 4

Dependency of single sp?: No

Ecology and traits (narrative): This spider shows an intermediate degree of morphological specialisation to subterranean life (Mammola et al. 2022a). According to thermal tests, *T.iulianae* shows a narrow thermal tolerance, reaching 50% mortality at temperature values 2°C above its cave temperature (Mammola et al. 2019a).

#### Threats

Justification for threats: As seen for the western alpine species of the genus *Troglohyphantes* (Mammola et al. 2018a), climate warming is expected to reduce the currently suitable habitat for this spider.

##### Threats

Threat type: Future

Threats: 11.1. Climate change & severe weather - Habitat shifting & alteration11.2. Climate change & severe weather - Droughts11.3. Climate change & severe weather - Temperature extremes

#### Conservation

Justification for conservation actions: This species has been recorded within several protected areas (EUAP0229 Parco Alpi Apuane and SPA IT5120015 Praterie primarie e secondarie delle Apuane, SAC IT1160020 Bosco di Bagnasco, SAC IT1343520 Zona Carsica Cassana, SAC IT5120014 Monte Corchia - Le Panie). Given the wide distribution of this species, it is reasonable to assume that it may occur in other protected areas and sites covered by the Natura 2000 network.

##### Conservation actions

Conservation action type: In Place

Conservation actions: 1.1. Land/water protection - Site/area protection1.2. Land/water protection - Resource & habitat protection2.1. Land/water management - Site/area management

#### Other

##### Use and trade

Use type: International

##### Ecosystem services

Ecosystem service type: Important

##### Research needed

Research needed: 1.2. Research - Population size, distribution & trends1.3. Research - Life history & ecology1.5. Research - Threats

Justification for research needed: Research on basic information such as distribution, ecology, life cycle and possible threats throughout the range would be needed.

### Troglohyphantes jamatus

#### Species information

Scientific name: Troglohyphantesjamatus

Species authority: Roewer, 1931

Kingdom: Animalia

Phylum: Arthropoda

Class: Arachnida

Order: Araneae

Family: Linyphiidae

Region for assessment: Global

#### Editor & Reviewers

##### Reviewers

Reviewers: Marc MilnePaulo Borges

##### Editor

Editor: Pedro Cardoso

#### Geographic range

Biogeographic realm: Palearctic

Countries: Slovenia

Map of records (Google Earth): Suppl. material 28

Basis of EOO and AOO: Observed

Basis (narrative): In light of its high level of specialisation, we assume that the known records of *T.jamatus* are good proxies for defining the EOO and AOO of this species.

Min Elevation/Depth (m): 491

Max Elevation/Depth (m): 705

Range description: This highly adapted subterranean species was recorded in seven caves in Inner Carniola and Littoral (western Slovenia) (detailed occurrences and relative references in Suppl. material 67).

#### Extent of occurrence

EOO (km2): 167

Trend: Decline (inferred)

Justification for trend: According to Mammola et al. (2018a), climate change will significantly affect the distribution of subterranean specialised *Troglohyphantes* in the future. Moreover, given the low tolerance to habitat changes of these species as well as their very low dispersal ability, a possible extreme reduction of the geographic range is expected in the future.

Causes ceased?: No

Causes understood?: Yes

Causes reversible?: No

Extreme fluctuations?: No

#### Area of occupancy

Trend: Decline (inferred)

Justification for trend: According to Mammola et al. (2018a), climate change will significantly affect the distribution of subterranean specialised *Troglohyphantes* in the future. Moreover, given the low tolerance to habitat changes of these species as well as their very low dispersal ability, a possible extreme reduction of the geographic range is expected in the future.

Causes ceased?: No

Causes understood?: Yes

Causes reversible?: No

Extreme fluctuations?: No

AOO (km2): 28

#### Locations

Number of locations: 1

Justification for number of locations: Even though this species occurs in seven caves, these are interpreted as a single location, as they are all affected by changes in subterranean microclimatic conditions due to climate change.

Trend: Stable

Extreme fluctuations?: No

#### Population

Number of individuals: Unknown

Trend: Decline (inferred)

Justification for trend: In view of the reduced thermal tolerance of subterranean specialised *Troglohyphantes* species (Mammola et al. 2019a), alterations of the microclimatic conditions of the habitat due to climate change are expected to impact the whole population of this species.

Basis for decline: (c) a decline in area of occupancy, extent of occurrence and/or quality of habitat

Causes ceased?: No

Causes understood?: Yes

Causes reversible?: No

Extreme fluctuations?: No

#### Subpopulations

Number of subpopulations: 6

Trend: Decline (inferred)

Justification for trend: Because of the adaptation to the subterranean habitat and the narrow thermal tolerance of highly adapted *Troglohyphantes*, likely hampering dispersal through non-subterranean habitats, each subpopulation of *T.jamatus* reasonably occurs in a single isolated or few contiguous localities. Accordingly, for this species we identified six subpopulations. These subpopulations are likely to be impacted by climate change.

Extreme fluctuations?: No

Severe fragmentation?: No

#### Habitat

System: Terrestrial

Habitat specialist: Yes

Habitat (narrative): The species is strictly relegated to cave habitat. No additional information about the habitat was available.

Trend in extent, area or quality?: Decline (inferred)

Justification for trend: As seen in Mammola et al. (2018a) for the species of the genus *Troglohyphantes* of the Western Alps, a drastic decline in the habitat suitability of *T.jamatus* as a consequence of climate change is also expected.

##### Habitat

Habitat importance: Major Importance

Habitats: 7. Caves and Subterranean Habitats (non-aquatic)

#### Ecology

Size: 3.1 mm

Generation length (yr): 4

Dependency of single sp?: No

Ecology and traits (narrative): This species shows a high degree of specialisation to deep subterranean habitats, with absence of pigmentation and eye reduction (Mammola et al. 2022a).

#### Threats

Justification for threats: This species is potentially exposed due to its extremely narrow geographic distribution range and its presumably low dispersal capacity. As seen for the *Troglohyphantes* species of the Western Alps (Mammola et al. 2018a), climate warming is expected to reduce the currently suitable habitat for this spider. One of the caves where the species occurs (Postojna Cave) is opened to tourism (more than 1,000,000 tourists per year). Consequently, a secondary impact for this subpopulation could derive from touristic activities.

##### Threats

Threat type: Future

Threats: 11.1. Climate change & severe weather - Habitat shifting & alteration11.2. Climate change & severe weather - Droughts11.3. Climate change & severe weather - Temperature extremes

#### Conservation

Justification for conservation actions: Due to its rarity, this species was considered as potentially threatened and listed in the Slovenian Red List of endangered plant and animal species (Uradni list RS št. 82/02 in 42/10), in the category R. The vast majority of this species' records are located within Special Areas of Conservation (SAC SI3000232 Notranjski trikotnik, SAC SI3000255 Trnovski gozd - Nanos, SAC SI3000256 Krimsko hribovje - Meni).

##### Conservation actions

Conservation action type: In Place

Conservation actions: 1.1. Land/water protection - Site/area protection1.2. Land/water protection - Resource & habitat protection2.1. Land/water management - Site/area management

#### Other

##### Use and trade

Use type: International

##### Ecosystem services

Ecosystem service type: Important

##### Research needed

Research needed: 1.2. Research - Population size, distribution & trends1.3. Research - Life history & ecology1.5. Research - Threats

Justification for research needed: Research on basic information such as distribution, ecology, life cycle and possible threats throughout the range would be needed.

### Troglohyphantes juris

#### Species information

Scientific name: Troglohyphantesjuris

Species authority: Thaler, 1982

Kingdom: Animalia

Phylum: Arthropoda

Class: Arachnida

Order: Araneae

Family: Linyphiidae

Region for assessment: Global

#### Editor & Reviewers

##### Reviewers

Reviewers: Marc MilnePaulo Borges

##### Editor

Editor: Pedro Cardoso

#### Geographic range

Biogeographic realm: Palearctic

Countries: Italy

Map of records (Google Earth): Suppl. material 29

Basis of EOO and AOO: Observed

Basis (narrative): In light of its high level of subterranean specialisation, we assume that the known records of *T.juris* are good proxies for defining the EOO and AOO of this species.

Min Elevation/Depth (m): 480

Max Elevation/Depth (m): 801

Range description: *Troglohyphantesjuris* was found in six caves in the Carnic Prealps (Friuli-Venezia Giulia, north-eastern Italy) (detailed occurrences and relative references in Suppl. material 67).

#### Extent of occurrence

EOO (km2): 55

Trend: Decline (inferred)

Justification for trend: According to Mammola et al. (2018a), climate change will significantly affect the distribution of subterranean specialised *Troglohyphantes* in the future. Moreover, given the low tolerance to habitat changes of these species as well as their very low dispersal ability, a possible extreme reduction of the geographic range is expected in the future.

Causes ceased?: No

Causes understood?: Yes

Causes reversible?: No

Extreme fluctuations?: No

#### Area of occupancy

Trend: Decline (inferred)

Justification for trend: According to Mammola et al. (2018a), climate change will significantly affect the distribution of subterranean specialised *Troglohyphantes* in the future. Moreover, given the low tolerance to habitat changes of these species as well as their very low dispersal ability, a possible extreme reduction of the geographic range is expected in the future.

Causes ceased?: No

Causes understood?: Yes

Causes reversible?: No

Extreme fluctuations?: No

AOO (km2): 24

#### Locations

Number of locations: 1

Justification for number of locations: Even though this species occurs in six caves, these are interpreted as a single location as they are all affected by changes in subterranean microclimatic conditions due to climate change.

Trend: Stable

Extreme fluctuations?: No

#### Population

Number of individuals: Unknown

Trend: Decline (inferred)

Justification for trend: In view of the reduced thermal tolerance of subterranean specialised *Troglohyphantes* species (Mammola et al. 2019a), alterations of the microclimatic conditions of the habitat due to climate change are expected to impact the whole population of this species.

Basis for decline: (c) a decline in area of occupancy, extent of occurrence and/or quality of habitat

Causes ceased?: No

Causes understood?: Yes

Causes reversible?: No

Extreme fluctuations?: No

#### Subpopulations

Number of subpopulations: 3

Trend: Decline (inferred)

Justification for trend: Because of the adaptation to the subterranean habitat and the narrow thermal tolerance of highly adapted *Troglohyphantes*, likely hampering dispersal through non-subterranean habitats, each subpopulation of *T.juris* can reasonably occur in a single isolated or few contiguous localities. Accordingly, for this species we identified three subpopulations occurring in the province of Pordenone, in Friuli-Venezia Giulia. These subpopulations are likely to be impacted by climate change.

Extreme fluctuations?: No

Severe fragmentation?: No

#### Habitat

System: Terrestrial

Habitat specialist: Yes

Habitat (narrative): This species was collected in deep cave habitats. No additional information on the habitat has been provided.

Trend in extent, area or quality?: Decline (inferred)

Justification for trend: As seen in Mammola et al. (2018a) for the species of the genus *Troglohyphantes* occurring in the Western Alps, a drastic decline in the habitat suitability of the *T.juris* as a consequence of climate change is expected.

##### Habitat

Habitat importance: Major Importance

Habitats: 7. Caves and Subterranean Habitats (non-aquatic)

#### Ecology

Size: 2.7 mm

Generation length (yr): 4

Dependency of single sp?: No

Ecology and traits (narrative): This spider shows a considerable eye reduction and absence of pigmentation (Mammola et al. 2022a).

#### Threats

Justification for threats: This species is potentially exposed due to its extremely narrow geographic distribution range and its presumably low dispersal capacity. As seen for the *Troglohyphantes* species of the Western Alps (Mammola et al. 2018a), climate warming is expected to reduce the currently suitable habitat for this spider.

##### Threats

Threat type: Future

Threats: 11.1. Climate change & severe weather - Habitat shifting & alteration11.2. Climate change & severe weather - Droughts11.3. Climate change & severe weather - Temperature extremes

#### Other

##### Use and trade

Use type: International

##### Ecosystem services

Ecosystem service type: Important

##### Research needed

Research needed: 1.2. Research - Population size, distribution & trends1.3. Research - Life history & ecology1.5. Research - Threats

Justification for research needed: Research on basic information such as distribution, ecology, life cycle and possible threats throughout the range would be needed.

### Troglohyphantes karawankorum

#### Species information

Scientific name: Troglohyphanteskarawankorum

Species authority: Deeleman-Reinhold, 1978

Kingdom: Animalia

Phylum: Arthropoda

Class: Arachnida

Order: Araneae

Family: Linyphiidae

Region for assessment: Global

#### Editor & Reviewers

##### Reviewers

Reviewers: Marc MilnePaulo Borges

##### Editor

Editor: Pedro Cardoso

#### Geographic range

Biogeographic realm: Palearctic

Countries: SloveniaAustria

Map of records (Google Earth): Suppl. material 30

Basis of EOO and AOO: Observed

Basis (narrative): This species was collected in very few localities. Its low level of subterranean specialisation, together with the high altimetric range observed in its known distribution, possibly reflects a higher dispersal capacity when compared to subterranean specialised species. Consequently, it may be possible that the present known range of this species is underestimated.

Min Elevation/Depth (m): 700

Max Elevation/Depth (m): 1880

Range description: This species is only known from the type locality, Jama na Babi, Jezersko (Upper Carniola, northern Slovenia), and from two localities in Carinthia (southern Austria) (detailed occurrences and relative references in Suppl. material 67).

#### Extent of occurrence

EOO (km2): 12

Trend: Stable

Justification for trend: This species is not strictly relegated to deep subterranean habitats, being collected both at a cave entrance and in epigean habitats. It is plausible that anthropogenic climate change may affect the habitat suitability of this species. However, in view of the relatively wide thermal tolerance and the relatively high dispersal ability of non-specialised *Troglohyphantes* (Mammola et al. 2019a), the distribution range of *T.karawankorum* is not expected to undergo significant reduction in the near future. A deeper study on the current distribution of this species and on the potential impacts of climate change is required.

Causes ceased?: Yes

Causes understood?: Yes

Causes reversible?: Yes

Extreme fluctuations?: No

#### Area of occupancy

Trend: Stable

Justification for trend: This species is not strictly relegated to deep subterranean habitats, being collected both at a cave entrance and in epigean habitats. It is plausible that anthropogenic climate change may affect the habitat suitability of this species. However, in view of the relatively wide thermal tolerance and the relatively high dispersal ability of non-specialised *Troglohyphantes* (Mammola et al. 2019a), the distribution range of *T.karawankorum* is not expected to undergo significant reduction in the near future. A deeper study on the current distribution of this species and on the potential impacts of climate change is required.

Causes ceased?: Yes

Causes understood?: Yes

Causes reversible?: Yes

Extreme fluctuations?: No

AOO (km2): 12

#### Locations

Number of locations: Not applicable

Justification for number of locations: No known threats to this species.

Trend: Stable

Extreme fluctuations?: No

#### Population

Number of individuals: Unknown

Trend: Stable

Justification for trend: There are no currently known threats to the species.

Causes ceased?: Yes

Causes understood?: Yes

Causes reversible?: Yes

Extreme fluctuations?: No

#### Subpopulations

Number of subpopulations: Unknown

Trend: Unknown

Extreme fluctuations?: No

Severe fragmentation?: No

#### Habitat

System: Terrestrial

Habitat specialist: No

Habitat (narrative): Specimens collected in the type locality, Jama na babi in Upper Carniola, have been found near the entrance of the cave, in a chamber with the floor covered by wood, stony debris, and leaf litter. The specimens from Carinthia have been collected in montane and subalpine zone, among crevices in boulder screes or in alpine grasslands.

Trend in extent, area or quality?: Unknown

##### Habitat

Habitat importance: Major Importance

Habitats: 4.4. Grassland - Temperate6. Rocky areas (e.g. inland cliffs, mountain peaks)7. Caves and Subterranean Habitats (non-aquatic)

#### Ecology

Size: 2.8 mm

Generation length (yr): 2

Dependency of single sp?: No

Ecology and traits (narrative): Specimens show eye regression and absence of pigmentation (Mammola et al. 2022a).

#### Threats

Justification for threats: This species is potentially exposed due to its extremely narrow geographic distribution range. However, the existence of direct threats to this species is unknown.

##### Threats

Threat type: Past

Threats: 12. Other options - Other threat

#### Conservation

Justification for conservation actions: This species was considered as potentially threatened due to its rarity and included in the category R in the Slovenian Red List (Uradni list RS št. 82/02 in 42/10). *Troglohyphanteskarawankorum* is considered one of the rarest spider species in Austria (Komposch 2009), and figures in the category R of the Red List of endangered spiders for Carinthia (Komposch and Steinberger 1999). In addition, this species figures in the 59th Regulation of the Carinthian State Government of 2015 (LGBl. Nr. 59/2015), and it is fully protected by the Carinthian Nature Conservation Act 2002 (LGBl. Nr. 79/2002).

##### Conservation actions

Conservation action type: In Place

#### Other

##### Use and trade

Use type: International

##### Ecosystem services

Ecosystem service type: Important

##### Research needed

Research needed: 1.2. Research - Population size, distribution & trends1.3. Research - Life history & ecology1.5. Research - Threats

Justification for research needed: Research on basic information such as distribution, ecology, life cycle and possible threats throughout the range would be needed.

### Troglohyphantes konradi

#### Species information

Scientific name: Troglohyphanteskonradi

Species authority: Brignoli, 1975

Kingdom: Animalia

Phylum: Arthropoda

Class: Arachnida

Order: Araneae

Family: Linyphiidae

**Figure 6.**
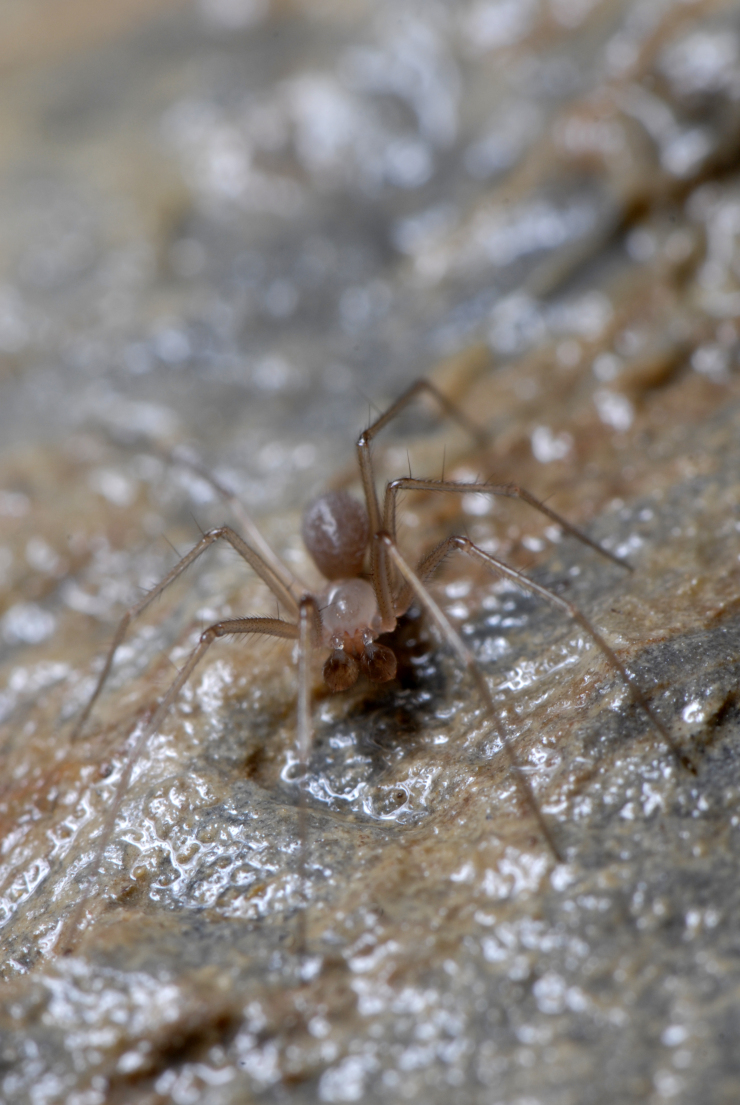
*Troglohyphanteskonradi* Brignoli, 1975. Photo credit: Francesco Tomasinelli.

Figure(s) or Photo(s): Fig. 6

Region for assessment: Global

#### Editor & Reviewers

##### Reviewers

Reviewers: Marc MilnePaulo Borges

##### Editor

Editor: Pedro Cardoso

#### Geographic range

Biogeographic realm: Palearctic

Countries: FranceItaly

Map of records (Google Earth): Suppl. material 31

Basis of EOO and AOO: Observed

Basis (narrative): Caves in Western Alps have been extensively sampled, allowing to define EOO and AOO of this species with reasonable confidence.

Min Elevation/Depth (m): 825

Max Elevation/Depth (m): 1493

Range description: This species is known from several caves and military bunkers in the Maritime Alps, between Gesso and Vermenagna Valleys (Piemonte, north-western Italy). Moreover, it was recently found in France, in a blockhouse near Brigue (Provence-Alpes-Côte d'Azur). Detailed occurrences and relative references are in Suppl. material 67.

#### Extent of occurrence

EOO (km2): 156

Trend: Decline (inferred)

Justification for trend: As seen in Mammola et al. (2018a) for other subterranean specialised *Troglohyphantes* of the Western Alps, climate change is expected to affect the distribution of this species in the future. Given the reduced thermal tolerance of this species and its low dispersal ability (Mammola et al. 2019a), a reduction of its geographic distribution range is expected in the future.

Causes ceased?: No

Causes understood?: Yes

Causes reversible?: No

Extreme fluctuations?: No

#### Area of occupancy

Trend: Decline (inferred)

Justification for trend: As seen in Mammola et al. (2018a) for other subterranean specialised *Troglohyphantes* of the Western Alps, climate change is expected to affect the distribution of this species in the future. Given the reduced thermal tolerance of this organism and its low dispersal ability (Mammola et al. 2019a), a reduction of its geographic distribution range is expected in the future.

Causes ceased?: No

Causes understood?: Yes

Causes reversible?: No

Extreme fluctuations?: No

AOO (km2): 20

#### Locations

Number of locations: 1

Justification for number of locations: The habitat where this species occurs is affected by changes in subterranean microclimatic conditions due to climate change, which is expected to impact the whole population (see Mammola et al. 2018a).

Trend: Stable

Extreme fluctuations?: No

#### Population

Number of individuals: Unknown

Trend: Decline (inferred)

Justification for trend: In view of the reduced thermal tolerance of this species (Mammola et al. 2019a), alterations of the microclimatic conditions of the habitat due to climate change are expected to impact the whole population of this species.

Basis for decline: (c) a decline in area of occupancy, extent of occurrence and/or quality of habitat

Causes ceased?: No

Causes understood?: Yes

Causes reversible?: No

Extreme fluctuations?: No

#### Subpopulations

Number of subpopulations: 4

Trend: Decline (inferred)

Justification for trend: Because of the subterranean adaptation and the narrow physiological tolerance of this species, hampering dispersal through non-subterranean habitats, each subpopulation reasonably occurs in a single isolated or in a few contiguous localities. Accordingly, for this species we identified four subpopulations in the range, three of them occurring in Valle Vermenagna and Valle Gesso, and a southern isolated subpopulation in the French Maritime Alps. All the subpopulations are likely to be impacted by climate change.

Extreme fluctuations?: No

Severe fragmentation?: No

#### Habitat

System: Terrestrial

Habitat specialist: Yes

Habitat (narrative): This species has been found at the ground level amid stones, in small interstices or on webs on the walls in caves, military bunkers, and blockhouses. Most of the cave openings are located in beech forests.

Trend in extent, area or quality?: Decline (inferred)

Justification for trend: As seen in Mammola et al. (2018a) for other species of the genus *Troglohyphantes* of the Western Alps, a drastic decline in the habitat suitability of *T.konradi* as a consequence of climate change is expected.

##### Habitat

Habitat importance: Major Importance

Habitats: 7. Caves and Subterranean Habitats (non-aquatic)

#### Ecology

Size: 3.5 mm

Generation length (yr): 4

Dependency of single sp?: No

Ecology and traits (narrative): This high subterranean specialised spider is totally depigmented, microphthalmic, and shows a rich spination (Mammola et al. 2022a). According to thermal tests, *T.konradi* shows a narrow thermal tolerance, reaching 50% mortality at temperature values 1°C above its cave temperature (Mammola et al. 2019a).

#### Threats

Justification for threats: This species is potentially exposed due to its extremely narrow geographic distribution range. As seen for other species of the genus *Troglohyphantes* of the Western Alps (Mammola et al. 2018a), climate warming is expected to reduce the currently suitable habitat for this spider. Moreover, in view of its reduced thermal tolerance (Mammola et al. 2019a), this species has a limited dispersal ability, which represents an additional concern in face of the ongoing increase of temperature.

##### Threats

Threat type: Future

Threats: 11.1. Climate change & severe weather - Habitat shifting & alteration11.2. Climate change & severe weather - Droughts11.3. Climate change & severe weather - Temperature extremes

#### Conservation

Justification for conservation actions: Part of the distribution of this species is within the borders of the Special Area of Conservation and Special Protection Area of the Maritime Alps (SAC/SPA IT1160056).

##### Conservation actions

Conservation action type: In Place

Conservation actions: 1.1. Land/water protection - Site/area protection1.2. Land/water protection - Resource & habitat protection

#### Other

##### Use and trade

Use type: International

##### Ecosystem services

Ecosystem service type: Important

##### Research needed

Research needed: 1.2. Research - Population size, distribution & trends1.3. Research - Life history & ecology1.5. Research - Threats

Justification for research needed: Research on basic information such as distribution, ecology, life cycle and possible threats throughout the range would be needed.

### Troglohyphantes kordunlikanus

#### Species information

Scientific name: Troglohyphanteskordunlikanus

Species authority: Deeleman-Reinhold, 1978

Kingdom: Animalia

Phylum: Arthropoda

Class: Arachnida

Order: Araneae

Family: Linyphiidae

**Figure 7.**
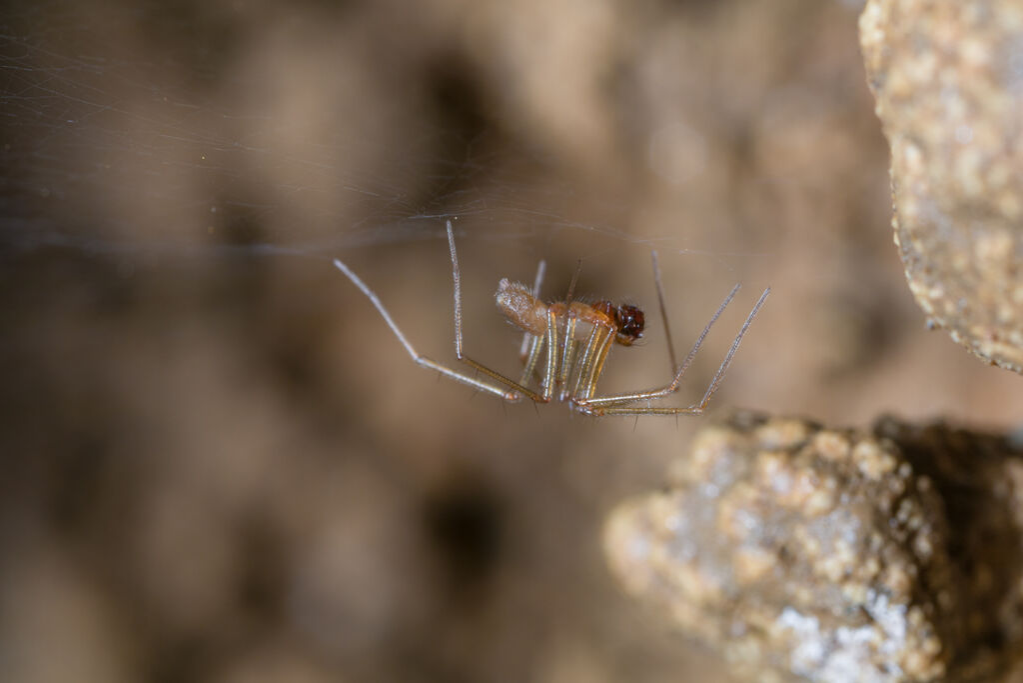
*Troglohyphanteskordunlikanus* Deeleman-Reinhold, 1978. Photo credit: Martina Pavlek.

Figure(s) or Photo(s): Fig. 7

Region for assessment: Global

#### Editor & Reviewers

##### Reviewers

Reviewers: Marc MilnePaulo Borges

##### Editor

Editor: Pedro Cardoso

#### Geographic range

Biogeographic realm: Palearctic

Countries: Bosnia and HerzegovinaCroatia

Map of records (Google Earth): Suppl. material 32

Basis of EOO and AOO: Species Distribution Model

Basis (narrative): Multiple collection sites are recorded for this species. Therefore, it was possible to perform species distribution modelling to predict its potential range with confidence limits. See Methods for details.

Min Elevation/Depth (m): 136

Max Elevation/Depth (m): 1353

Range description: This species has been found in the Primorje-Gorski Kotar, Karlovac, Lika-Senj, and Zadar counties in Croatia, and in north-western Bosnia and Herzegovina (detailed occurrences and relative references in Suppl. material 67).

#### Extent of occurrence

EOO (km2): 18072-27428,19817

Trend: Stable

Justification for trend: This species has been collected in caves and shallow subterranean habitats as well as under stones and in animal burrows in moist leaf litter. It is plausible that anthropogenic climate change may affect the habitat suitability of this species. However, in view of the relatively wide thermal tolerance and the relatively high dispersal ability of non-specialised species of *Troglohyphantes* (Mammola et al. 2019a), the distribution range of *T.kordunlikanus* is not expected to undergo significant reduction in the near future. A deeper study on the impacts of climate change on this species is required.

Causes ceased?: Yes

Causes understood?: Yes

Causes reversible?: Yes

Extreme fluctuations?: No

#### Area of occupancy

Trend: Stable

Justification for trend: This species has been collected in caves and shallow subterranean habitats as well as under stones and in animal burrows in moist leaf litter. It is plausible that anthropogenic climate change may affect the habitat suitability of this species. However, in view of the relatively wide thermal tolerance and the relatively high dispersal ability of non-specialised species of *Troglohyphantes* (Mammola et al. 2019a), the distribution range of *T.kordunlikanus* is not expected to undergo significant reduction in the near future. A deeper study on the impacts of climate change on this species is required.

Causes ceased?: Yes

Causes understood?: Yes

Causes reversible?: Yes

Extreme fluctuations?: No

AOO (km2): 13328-21464,15368

#### Locations

Number of locations: Not applicable

Justification for number of locations: No known threats to this species.

Trend: Stable

Extreme fluctuations?: No

#### Population

Number of individuals: Unknown

Trend: Stable

Justification for trend: There are no currently known threats to the species.

Causes ceased?: Yes

Causes understood?: Yes

Causes reversible?: Yes

Extreme fluctuations?: No

#### Subpopulations

Number of subpopulations: Unknown

Trend: Stable

Extreme fluctuations?: No

Severe fragmentation?: No

#### Habitat

System: Terrestrial

Habitat specialist: No

Habitat (narrative): This species has been found on the ceiling and the walls of caves, mainly at no more than 50 meters from the entrance, and in small animal burrows and under stones in forest habitats. In Lika, in the western part of the range, this species was found only in caves. Deeleman-Reinhold (1978) pointed out that in historical times this area was almost completely stripped of the original forest cover, and that the present vegetation consists mainly of Mediterranean shrubs and dry woods of *Carpinus* or hornbeam-oak, possibly not suitable for this species.

Trend in extent, area or quality?: Stable

##### Habitat

Habitat importance: Major Importance

Habitats: 1.4. Forest - Temperate7. Caves and Subterranean Habitats (non-aquatic)

#### Ecology

Size: 2.4 mm

Generation length (yr): 2

Dependency of single sp?: No

Ecology and traits (narrative): Not much is known about the ecology of this species. All the individuals are depigmented (even the ones collected outside the caves), and have normally developed eyes circled with black pigmentation of variable width (Mammola et al. 2022a).

#### Threats

Justification for threats: This species is potentially exposed due to its restricted geographic distribution range. However, the existence of threats is unknown for this species.

##### Threats

Threat type: Past

Threats: 12. Other options - Other threat

#### Conservation

Justification for conservation actions: This species has been found in several protected areas and sites of the Natura 2000 network. The species distribution modelling predicts that it could also be present in further protected areas.

##### Conservation actions

Conservation action type: In Place

Conservation actions: 1.1. Land/water protection - Site/area protection1.2. Land/water protection - Resource & habitat protection

#### Other

##### Use and trade

Use type: International

##### Ecosystem services

Ecosystem service type: Important

##### Research needed

Research needed: 1.2. Research - Population size, distribution & trends1.3. Research - Life history & ecology1.5. Research - Threats

Justification for research needed: Research on basic information such as distribution, ecology, life cycle and possible threats throughout the range would be needed.

### Troglohyphantes lanai

#### Species information

Scientific name: Troglohyphanteslanai

Species authority: Isaia & Pantini, 2010

Kingdom: Animalia

Phylum: Arthropoda

Class: Arachnida

Order: Araneae

Family: Linyphiidae

Region for assessment: Global

#### Editor & Reviewers

##### Reviewers

Reviewers: Marc MilnePaulo Borges

##### Editor

Editor: Pedro Cardoso

#### Geographic range

Biogeographic realm: Palearctic

Countries: Italy

Map of records (Google Earth): Suppl. material 33

Basis of EOO and AOO: Observed

Basis (narrative): Caves in Western Alps have been extensively sampled, allowing to define EOO and AOO of this species with reasonable confidence.

Min Elevation/Depth (m): 640

Max Elevation/Depth (m): 864

Range description: This species is restricted to six caves of the Monte Fenera hypogean complex, a limestone cave complex in the Northwestern Alps (province of Vercelli, Piemonte, north-western Italy) (detailed occurrences and relative references in Suppl. material 67).

#### Extent of occurrence

EOO (km2): 8

Trend: Decline (inferred)

Justification for trend: As seen in Mammola et al. (2018a) for all the subterranean specialised *Troglohyphantes* species of the Western Alps, climate change is expected to affect the distribution of this species in the future. Given the reduced thermal tolerance of this organism and its low dispersal ability (Mammola et al. 2019a), a reduction of its geographic distribution range is expected in the future.

Causes ceased?: No

Causes understood?: Yes

Causes reversible?: No

Extreme fluctuations?: No

#### Area of occupancy

Trend: Decline (inferred)

Justification for trend: As seen in Mammola et al. (2018a) for all the subterranean specialised *Troglohyphantes* species of the Western Alps, climate change is expected to affect the distribution of this species in the future. Given the reduced thermal tolerance of this organism and its low dispersal ability (Mammola et al. 2019a), a reduction of its geographic distribution range is expected in the future.

Causes ceased?: No

Causes understood?: Yes

Causes reversible?: No

Extreme fluctuations?: No

AOO (km2): 8

#### Locations

Number of locations: 1

Justification for number of locations: The habitat where this species occurs is affected by changes in subterranean microclimatic conditions due to climate change, which is expected to impact the whole population (see Mammola et al. 2018a).

Trend: Stable

Extreme fluctuations?: No

#### Population

Number of individuals: Unknown

Trend: Decline (inferred)

Justification for trend: In view of the reduced thermal tolerance of this species (Mammola et al. 2019a), alterations of the microclimatic conditions of the habitat due to climate change are expected to impact the whole population of this species.

Basis for decline: (c) a decline in area of occupancy, extent of occurrence and/or quality of habitat

Causes ceased?: No

Causes understood?: Yes

Causes reversible?: No

Extreme fluctuations?: No

#### Subpopulations

Number of subpopulations: 1

Trend: Stable

Justification for trend: Because of the adaptation to the subterranean habitat and of the narrow thermal tolerance of this species, likely hampering dispersal through non-subterranean habitats, each locality can reasonably host a single isolated subpopulation. Accordingly, for this species we identified a single subpopulation occurring in the Monte Fenera hypogean complex.

Extreme fluctuations?: No

Severe fragmentation?: No

#### Habitat

System: Terrestrial

Habitat specialist: Yes

Habitat (narrative): Specimens of *T.lanai* have been found among stony debris in the caves of the complex of Monte Fenera (limestone with intercalated sandstone), in the Pennine Alps (north-western Italy). All caves have openings into beech woods, with a prevalent northerly aspect to the cave opening, The temperatures in the caves are constantly about 9°C (Isaia and Pantini 2010).

Trend in extent, area or quality?: Decline (inferred)

Justification for trend: As seen in Mammola et al. (2018a) for all the subterranean specialised *Troglohyphantes* of the Western Alps, a drastic decline in the habitat suitability of *T.lanai* as a consequence of climate change is expected.

##### Habitat

Habitat importance: Major Importance

Habitats: 7. Caves and Subterranean Habitats (non-aquatic)

#### Ecology

Size: 3.1 mm

Generation length (yr): 4

Dependency of single sp?: No

Ecology and traits (narrative): This species shows particularly remarkable subterranean traits, such as pronounced eye regression and absence of pigmentation (Mammola et al. 2022a). According to thermal tests, *T.lanai* shows a narrow thermal tolerance, reaching 50% mortality at temperature values 2°C above its cave temperature (Mammola et al. 2019a).

#### Threats

Justification for threats: This species is potentially exposed due to its extremely narrow geographic distribution range and its presumably low dispersal capacity. As seen for other western alpine *Troglohyphantes* species (Mammola et al. 2018a), climate warming is expected to reduce the currently suitable habitat for this spider.

##### Threats

Threat type: Future

Threats: 11.1. Climate change & severe weather - Habitat shifting & alteration11.2. Climate change & severe weather - Droughts11.3. Climate change & severe weather - Temperature extremes

#### Conservation

Justification for conservation actions: The species range falls entirely within the Natural Park of Monte Fenera (SAC IT1120003). However, there are no conservation measures in place for this species.

##### Conservation actions

Conservation action type: In Place

Conservation actions: 1.1. Land/water protection - Site/area protection1.2. Land/water protection - Resource & habitat protection

#### Other

##### Use and trade

Use type: International

##### Ecosystem services

Ecosystem service type: Important

##### Research needed

Research needed: 1.2. Research - Population size, distribution & trends1.3. Research - Life history & ecology1.5. Research - Threats

Justification for research needed: Research on basic information such as natural history, ecology and possible threats of the species would be needed.

### Troglohyphantes latzeli

#### Species information

Scientific name: Troglohyphanteslatzeli

Species authority: Thaler, 1986

Kingdom: Animalia

Phylum: Arthropoda

Class: Arachnida

Order: Araneae

Family: Linyphiidae

Region for assessment: Global

#### Editor & Reviewers

##### Reviewers

Reviewers: Marc MilnePaulo Borges

##### Editor

Editor: Pedro Cardoso

#### Geographic range

Biogeographic realm: Palearctic

Countries: SloveniaAustria

Map of records (Google Earth): Suppl. material 34

Basis of EOO and AOO: Observed

Basis (narrative): This species was collected in very few localities. Its low level of subterranean specialisation, together with the high altimetric range found in its known distribution, possibly reflects a higher dispersal capacity when compared to subterranean specialised species. Consequently, it may be possible that the present known range of this species is underestimated.

Min Elevation/Depth (m): 560

Max Elevation/Depth (m): 1300

Range description: This species has been documented only in a few localities between Carinthia (southern Austria) and Upper Carniola (northern Slovenia) (detailed occurrences and relative references in Suppl. material 67).

#### Extent of occurrence

EOO (km2): 467

Trend: Decline (inferred)

Justification for trend: Habitat loss and land use change due to urbanisation and infrastructure development in some of the localities where this species has been collected (Komposch 2009), indicate the species is likely declining.

Causes ceased?: No

Causes understood?: Yes

Causes reversible?: Yes

Extreme fluctuations?: No

#### Area of occupancy

Trend: Decline (inferred)

Justification for trend: Habitat loss and land use change due to urbanisation and infrastructure development in some of the localities where this species has been collected (Komposch 2009), indicate the species is likely declining.

Causes ceased?: No

Causes understood?: Yes

Causes reversible?: Yes

Extreme fluctuations?: No

AOO (km2): 46

#### Locations

Number of locations: 2

Justification for number of locations: Two locations, corresponding to the Austrian subpopulations, are threatened by urbanisation and infrastructure development.

Trend: Decline (inferred)

Extreme fluctuations?: No

#### Population

Number of individuals: Unknown

Trend: Decline (inferred)

Justification for trend: The population is threatened by habitat loss and land use change due to urbanisation and infrastructure development in part of its range.

Causes ceased?: No

Causes understood?: Yes

Causes reversible?: Yes

Extreme fluctuations?: No

#### Subpopulations

Number of subpopulations: 3

Trend: Decline (inferred)

Justification for trend: Based on the data available, for this species we identified three subpopulations: two of them occurring in southern Austria and one in northern Slovenia. The Austrian subpopulations are affected by habitat loss and land use change due to urban and transport infrastructure development.

Extreme fluctuations?: No

Severe fragmentation?: No

#### Habitat

System: Terrestrial

Habitat specialist: No

Habitat (narrative): This species has been collected in different habitats in the medium montane belt, from 500 up to 1,300 m, such as pastures, prairies, broadleaf, and conifer forests. It has also been found in rock crevices in boulder fields.

Trend in extent, area or quality?: Decline (inferred)

Justification for trend: Some of the habitats where this species has been found are threatened by urban, industrial, and transport infrastructure development (Komposch 2009).

##### Habitat

Habitat importance: Major Importance

Habitats: 1.4. Forest - Temperate4.4. Grassland - Temperate6. Rocky areas (e.g. inland cliffs, mountain peaks)

#### Ecology

Size: 2.5 mm

Generation length (yr): 2

Dependency of single sp?: No

Ecology and traits (narrative): This spider shows minor specialisation to subterranean life (Mammola et al. 2022a).

#### Threats

Justification for threats: This species currently faces threats of habitat loss and land use change due to urbanisation and due to railway and road infrastructure development (Komposch 2009).

##### Threats

Threat type: Ongoing

Threats: 1.1. Residential & commercial development - Housing & urban areas1.2. Residential & commercial development - Commercial & industrial areas4.1. Transportation & service corridors - Roads & railroads

#### Conservation

Justification for conservation actions: The records from Slovenia fall within the Special Area of Conservation and Special Protection Area of the Julian Alps (SAC SI3000253, SPA SI5000019). In Austria, *T.latzeli* has been listed in the 59th Regulation of the Carinthian State Government of 2015 (LGBl. Nr. 59/2015), which amends the Carinthian Nature Conservation Act 2002 (LGBl. Nr. 79/2002).

##### Conservation actions

Conservation action type: In Place

Conservation actions: 1.1. Land/water protection - Site/area protection1.2. Land/water protection - Resource & habitat protection2.1. Land/water management - Site/area management

#### Other

##### Use and trade

Use type: International

##### Ecosystem services

Ecosystem service type: Important

##### Research needed

Research needed: 1.2. Research - Population size, distribution & trends1.3. Research - Life history & ecology1.5. Research - Threats

Justification for research needed: Research on basic information such as natural history, ecology and possible threats of the species would be needed.

### Troglohyphantes lessinensis

#### Species information

Scientific name: Troglohyphanteslessinensis

Species authority: Caporiacco, 1936

Kingdom: Animalia

Phylum: Arthropoda

Class: Arachnida

Order: Araneae

Family: Linyphiidae

Region for assessment: Global

#### Editor & Reviewers

##### Reviewers

Reviewers: Marc MilnePaulo Borges

##### Editor

Editor: Pedro Cardoso

#### Geographic range

Biogeographic realm: Palearctic

Countries: Italy

Map of records (Google Earth): Suppl. material 35

Basis of EOO and AOO: Observed

Basis (narrative): Despite the relatively high number of records of this non-specialised species, the species distribution predicted by the models was found to be unreliable by our own expert opinion. In view of this, only the observed distribution range is presented.

Min Elevation/Depth (m): 197

Max Elevation/Depth (m): 2599

Range description: This species has been found in several caves in the Lessini Mountains, Monte Baldo, and other localities of the Venetian Prealps, and in the southern Trentino-Alto Adige (north-eastern Italy). New records were recently collected in epigean habitats in the Brenta Dolomites, extending considerably the distribution of this species northwards (detailed occurrences and relative references in Suppl. material 67).

#### Extent of occurrence

EOO (km2): 2269

Trend: Stable

Justification for trend: This species has been mainly found in caves, but several specimens have been recorded in epigean habitats. It is plausible that anthropogenic climate change may affect the habitat suitability of this species. However, in view of the relatively wide thermal tolerance and the relatively high dispersal ability of non-specialised species of *Troglohyphantes* (Mammola et al. 2019a), the distribution range of *T.lessinensis* is not expected to undergo significant reduction in the near future. A deeper study on the current distribution of this species and on the potential impacts of climate change is required.

Causes ceased?: Yes

Causes understood?: Yes

Causes reversible?: Yes

Extreme fluctuations?: No

#### Area of occupancy

Trend: Stable

Justification for trend: This species has been mainly found in caves, but several specimens have been recorded in epigean habitats. It is plausible that anthropogenic climate change may affect the habitat suitability of this species. However, in view of the relatively wide thermal tolerance and the relatively high dispersal ability of non-specialised species of *Troglohyphantes* (Mammola et al. 2019a), the distribution range of *T.lessinensis* is not expected to undergo significant reduction in the near future. A deeper study on the current distribution of this species and on the potential impacts of climate change is required.

Causes ceased?: Yes

Causes understood?: Yes

Causes reversible?: Yes

Extreme fluctuations?: No

AOO (km2): 104

#### Locations

Number of locations: Not applicable

Justification for number of locations: No known threats to this species.

Trend: Stable

Extreme fluctuations?: No

#### Population

Number of individuals: Unknown

Trend: Stable

Justification for trend: There are no currently known threats to the species.

Causes ceased?: Yes

Causes understood?: Yes

Causes reversible?: Yes

Extreme fluctuations?: No

#### Subpopulations

Number of subpopulations: Unknown

Trend: Unknown

Extreme fluctuations?: No

Severe fragmentation?: No

#### Habitat

System: Terrestrial

Habitat specialist: No

Habitat (narrative): *Troglohyphanteslessinensis* was collected mainly in natural and artificial hypogean habitats. However, specimens have been recently found also in high altitude rocky lands and alpine screes.

Trend in extent, area or quality?: Stable

Justification for trend: The habitats colonised by *T.lessinensis* are as yet not threatened by direct human activities.

##### Habitat

Habitat importance: Major Importance

Habitats: 1.4. Forest - Temperate6. Rocky areas (e.g. inland cliffs, mountain peaks)7. Caves and Subterranean Habitats (non-aquatic)

#### Ecology

Size: 3.2 mm

Generation length (yr): 2

Dependency of single sp?: No

Ecology and traits (narrative): Specimens are characterised by a pronounced microphthalmy (Mammola et al. 2022a).

#### Threats

Justification for threats: This species is potentially exposed due to its restricted geographic distribution range. However, the existence of threats is unknown for this species.

##### Threats

Threat type: Past

Threats: 12. Other options - Other threat

#### Conservation

Justification for conservation actions: This species has been found within the Natura 2000 network (SAC/SPA IT3210040 Monti Lessini - Pasubio - Piccole Dolomiti Vicentine, SAC/SPA IT3210039 Monte Baldo Ovest, SAC/SPA IT3210041 Monte Baldo Est, EUAP0930 and SAC IT3120177 Dolomiti di Brenta, SPA IT3120159 Brenta).

##### Conservation actions

Conservation action type: In Place

Conservation actions: 1.1. Land/water protection - Site/area protection1.2. Land/water protection - Resource & habitat protection2.1. Land/water management - Site/area management

#### Other

##### Use and trade

Use type: International

##### Ecosystem services

Ecosystem service type: Important

##### Research needed

Research needed: 1.2. Research - Population size, distribution & trends1.3. Research - Life history & ecology1.5. Research - Threats

Justification for research needed: Research on basic information such as natural history, ecology and possible threats of the species would be needed.

### Troglohyphantes liburnicus

#### Species information

Scientific name: Troglohyphantesliburnicus

Species authority: Caporiacco, 1927

Kingdom: Animalia

Phylum: Arthropoda

Class: Arachnida

Order: Araneae

Family: Linyphiidae

**Figure 8.**
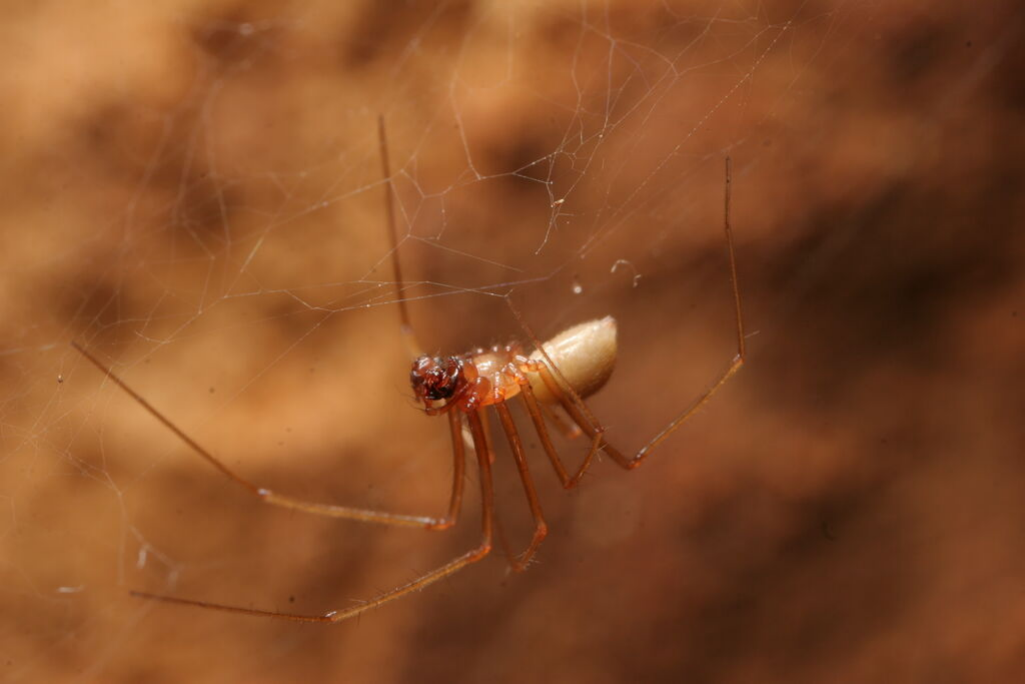
*Troglohyphantesliburnicus* Caporiacco, 1927. Photo credit: Helena Bilandžija.

Figure(s) or Photo(s): Fig. 8

Region for assessment: Global

#### Editor & Reviewers

##### Reviewers

Reviewers: Marc MilnePaulo Borges

##### Editor

Editor: Pedro Cardoso

#### Geographic range

Biogeographic realm: Palearctic

Countries: Croatia

Map of records (Google Earth): Suppl. material 36

Basis of EOO and AOO: Observed

Basis (narrative): In light of its subterranean specialisation, we assume that the known records of *T.liburnicus* are good proxies for defining the AOO and EOO of this species.

Min Elevation/Depth (m): 132

Max Elevation/Depth (m): 929

Range description: *Troglohyphantesliburnicus* has been collected in several caves in the Primorje-Gorski Kotar county and in the northern part of the Island of Cres (detailed occurrences and relative references in Suppl. material 67).

#### Extent of occurrence

EOO (km2): 727

Trend: Decline (inferred)

Justification for trend: Most of the potential distribution of this species corresponds to the coastal area of the Croatian Littoral, where the urban infrastructure development and the industrial pollution are resulting in large-scale alterations of the natural environment, which affect the habitat of this species (Ozimec et al. 2009). In addition, according to Mammola et al. (2018a), climate change will significantly affect the distribution of subterranean specialised *Troglohyphantes* in the future. Moreover, given the low tolerance to habitat changes of these species as well as their very low dispersal ability, a possible extreme reduction of the geographic range is expected in the future.

Causes ceased?: No

Causes understood?: Yes

Causes reversible?: No

Extreme fluctuations?: No

#### Area of occupancy

Trend: Decline (inferred)

Justification for trend: Most of the potential distribution of this species corresponds to the coastal area of the Croatian Littoral, where the urban infrastructure development and the industrial pollution are resulting in large-scale alterations of the natural environment, which affect the habitat of this species (Ozimec et al. 2009). In addition, according to Mammola et al. (2018a), climate change will significantly affect the distribution of subterranean specialised *Troglohyphantes* in the future. Moreover, given the low tolerance to habitat changes of these species as well as their very low dispersal ability, a possible extreme reduction of the geographic range is expected in the future.

Causes ceased?: No

Causes understood?: Yes

Causes reversible?: No

Extreme fluctuations?: No

AOO (km2): 64

#### Locations

Number of locations: 1

Justification for number of locations: Some of the caves where this species occurs are located in an area heavily impacted by urban development, tourism and industrial pollution. However, all the caves where this species occurs are affected by changes in subterranean microclimatic conditions due to climate change, which is considered the most serious plausible threat expected to impact the whole population (see Mammola et al. 2018a).

Trend: Stable

Extreme fluctuations?: No

#### Population

Number of individuals: Unknown

Trend: Decline (inferred)

Justification for trend: Habitat loss and degradation due to urban development and pollution affects the population of *T.liburnicus*. In addition, in view of the reduced thermal tolerance of subterranean specialised *Troglohyphantes* species (Mammola et al. 2019a), alterations of the microclimatic conditions of the habitat due to climate change are expected to impact the whole population of this species.

Basis for decline: (c) a decline in area of occupancy, extent of occurrence and/or quality of habitat

Causes ceased?: No

Causes understood?: Yes

Causes reversible?: No

Extreme fluctuations?: No

#### Subpopulations

Number of subpopulations: 3-6

Trend: Decline (inferred)

Justification for trend: Because of the adaptation to the subterranean habitat and the narrow thermal tolerance of highly adapted *Troglohyphantes*, likely hampering dispersal through non-subterranean habitats, each subpopulation of *T.liburnicus* reasonably occurs in a single isolated or few contiguous localities. Accordingly, for this species we identified three to six subpopulations. Urbanisation and industrialisation are affecting most of them throughout this species' range.

Extreme fluctuations?: No

Severe fragmentation?: No

#### Habitat

System: Terrestrial

Habitat specialist: No

Habitat (narrative): This species has been found both in the twilight zone and in the deepest part of the caves, in the recesses or between rocks and stalagmites on the ground. In the pit Jama kod Škalnice, specimens occurred in webs on a steep slope covered with wood debris and pieces of rock.

Trend in extent, area or quality?: Decline (inferred)

Justification for trend: Most of the localities where this species occurs are threatened by habitat loss and degradation due to urban infrastructure development and residential and industrial pollution (Ozimec et al. 2009). Moreover, as seen in Mammola et al. (2018a) for other species of the genus *Troglohyphantes* of the Western Alps, a drastic decline in the habitat suitability of *T.liburnicus* as a consequence of climate change is expected.

##### Habitat

Habitat importance: Major Importance

Habitats: 7. Caves and Subterranean Habitats (non-aquatic)

#### Ecology

Size: 4.5 mm

Generation length (yr): 4

Dependency of single sp?: No

Ecology and traits (narrative): Specimens show pronounced morphological specialisation to the subterranean habitat, such as eyes regression, leg elongation, and absence of pigmentation (Mammola et al. 2022a).

#### Threats

Justification for threats: This species is potentially exposed due to its extremely narrow geographic distribution range and its presumably low dispersal capacity. Habitat loss and degradation due to urban infrastructure development and due to pollution from commercial and industrial facilities, represents an ongoing threat to *T.liburnicus*. Pollution by solid and liquid municipal waste from illegal landfills causes the degradation of cave ecosystems (Ozimec et al. 2009). The type locality is located near both the railway and the major road connecting the city of Rijeka with the Slovenian border. This area, corresponding to the municipality of Matulji, is highly urbanized, with the urban infrastructure growth increasing rapidly in recent years. In addition, as seen for other species of the genus *Troglohyphantes* of the Western Alps (Mammola et al. 2018a), climate warming is expected to reduce the currently suitable habitat for this spider.

##### Threats

Threat type: Ongoing

Threats: 1.1. Residential & commercial development - Housing & urban areas1.2. Residential & commercial development - Commercial & industrial areas9.1. Pollution - Domestic & urban waste water9.2. Pollution - Industrial & military effluents

##### Threats

Threat type: Future

Threats: 11.1. Climate change & severe weather - Habitat shifting & alteration11.2. Climate change & severe weather - Droughts11.3. Climate change & severe weather - Temperature extremes

#### Conservation

Justification for conservation actions: This species is listed as Vulnerable in the Red Book of Croatian Cave Dwelling Fauna (Ozimec et al. 2009). As a cave species, *T.liburnicus* is strictly legally protected according to the existing Nature Protection Act (Official Gazette 70/05, NN 139/2008), which prohibits collection, killing, and disturbance of specimens and destruction of their habitats without permission from the Ministry in charge of nature protection. The distribution of this species falls within several protected areas (SPA HR1000033 Kvarnerski otoci, SCI HR2001358 Otok Cres, SPA HR1000018 Učka i Ćićarija, SCI HR2000601 Park prirode Učka, SCI/SPA HR5000019 Gorski kotar i sjeverna Lika).

##### Conservation actions

Conservation action type: In Place

Conservation actions: 1.1. Land/water protection - Site/area protection1.2. Land/water protection - Resource & habitat protection

##### Conservation actions

Conservation action type: Needed

Conservation actions: 2.1. Land/water management - Site/area management2.3. Land/water management - Habitat & natural process restoration

#### Other

##### Use and trade

Use type: International

##### Ecosystem services

Ecosystem service type: Important

##### Research needed

Research needed: 1.2. Research - Population size, distribution & trends1.3. Research - Life history & ecology1.5. Research - Threats3.1. Monitoring - Population trends3.4. Monitoring - Habitat trends

Justification for research needed: Research on basic information such as natural history, ecology and possible threats of the species would be needed. Population status and habitat quality should be monitored.

### Troglohyphantes lucifer

#### Species information

Scientific name: Troglohyphanteslucifer

Species authority: Isaia, Mammola & Pantini, 2017

Kingdom: Animalia

Phylum: Arthropoda

Class: Arachnida

Order: Araneae

Family: Linyphiidae

**Figure 9.**
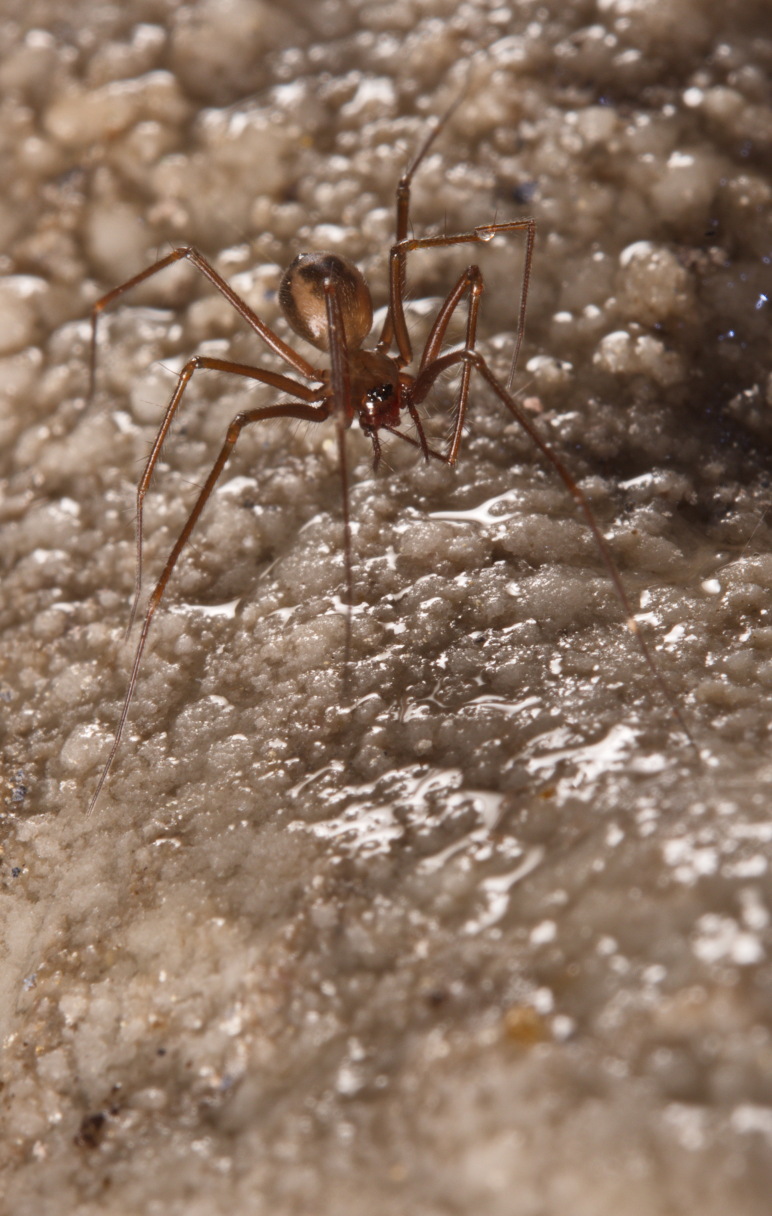
*Troglohyphanteslucifer* Isaia, Mammola & Pantini, 2017. Photo credit: Francesco Tomasinelli.

Figure(s) or Photo(s): Fig. 9

Region for assessment: Global

#### Editor & Reviewers

##### Reviewers

Reviewers: Marc MilnePaulo Borges

##### Editor

Editor: Pedro Cardoso

#### Geographic range

Biogeographic realm: Palearctic

Countries: Italy

Map of records (Google Earth): Suppl. material 37

Basis of EOO and AOO: Observed

Basis (narrative): This species was collected in a few localities in the Western Alps. Its low level of subterranean specialisation, together with the high altimetric range found in its known distribution, possibly reflects a higher dispersal capacity when compared to subterranean specialised species. Consequently, it may be possible that the present known range of this species is underestimated. Given this situation, any modelling of the current habitat suitability is unreliable.

Min Elevation/Depth (m): 410

Max Elevation/Depth (m): 1777

Range description: This species occurs both in hypogean and epigean localities in the alpine districts of Northern Cottian and Southern Graian Alps (Piemonte, north-western Italy) (detailed occurrences and relative references in Suppl. material 67).

#### Extent of occurrence

EOO (km2): 1176

Trend: Stable

Justification for trend: This species is not strictly relegated to deep subterranean habitats, being collected in the vicinity of the cave entrance or in shallow subterranean habitats. It is plausible that anthropogenic climate change may affect the habitat suitability of this species. However, in view of the relatively wide thermal tolerance and the relatively high dispersal ability of non-specialised *Troglohyphantes* (Mammola et al. 2019a), the distribution range of *T.lucifer* is not expected to undergo significant reduction in the near future. A deeper study on the current distribution of this species and on the potential impacts of climate change is required.

Causes ceased?: Yes

Causes understood?: Yes

Causes reversible?: Yes

Extreme fluctuations?: No

#### Area of occupancy

Trend: Stable

Justification for trend: This species is not strictly relegated to deep subterranean habitats, being collected in the vicinity of the cave entrance or in shallow subterranean habitats. It is plausible that anthropogenic climate change may affect the habitat suitability of this species. However, in view of the relatively wide thermal tolerance and the relatively high dispersal ability of non-specialised *Troglohyphantes* (Mammola et al. 2019a), the distribution range of *T.lucifer* is not expected to undergo significant reduction in the near future. A deeper study on the current distribution of this species and on the potential impacts of climate change is required.

Causes ceased?: Yes

Causes understood?: Yes

Causes reversible?: Yes

Extreme fluctuations?: No

AOO (km2): 40

#### Locations

Number of locations: Not applicable

Justification for number of locations: No known threats to this species.

Trend: Stable

Extreme fluctuations?: No

#### Population

Number of individuals: Unknown

Trend: Stable

Justification for trend: There are no currently known threats to this species.

Causes ceased?: Yes

Causes understood?: Yes

Causes reversible?: Yes

Extreme fluctuations?: No

#### Subpopulations

Number of subpopulations: Unknown

Trend: Unknown

Extreme fluctuations?: No

Severe fragmentation?: No

#### Habitat

System: Terrestrial

Habitat specialist: No

Habitat (narrative): *Troglohyphanteslucifer* has been collected in floors and walls of the twilight zone of natural caves, in block fields in beech forests, and in various shallow subterranean habitats, leaf litter, deep soil strata, MSS, and rocky accumulations. According to Isaia et al. (2017), in certain localities *T.lucifer* is able to coexist with other *Troglohyphantes* spiders. In this regard, Deeleman-Reinhold (1978) suggested that the co-occurrence of more species of *Troglohyphantes* is rare and may occur exclusively in phylogenetically distant lineages.

Trend in extent, area or quality?: Unknown

##### Habitat

Habitat importance: Major Importance

Habitats: 1.4. Forest - Temperate6. Rocky areas (e.g. inland cliffs, mountain peaks)7. Caves and Subterranean Habitats (non-aquatic)

#### Ecology

Size: 3.5 mm

Generation length (yr): 2

Dependency of single sp?: No

Ecology and traits (narrative): This species is not exclusively restricted to deep subterranean habitats, being collected in the vicinity of the cave entrance or in shallow subterranean habitats. Specimens show minor morphological specialisation to subterranean life, with eyes normally developed and abdominal pattern present (Mammola et al. 2022a). According to thermal tests, *T.lucifer* shows a great thermal tolerance, reaching 50% mortality at temperature values 19°C above its cave temperature (Mammola et al. 2019a).

#### Threats

Justification for threats: The existence of threats is unknown for this species.

##### Threats

Threat type: Past

Threats: 12. Other options - Other threat

#### Conservation

Justification for conservation actions: This species has been recorded in the Natura 2000 sites SAC/SPA IT1110006 Orsiera Rocciavré, SAC IT1110029 Pian della Mussa (Balme), and SAC IT1110048 Grotta del Pugnetto. In one case, Grotte del Pugnetto, the entrance is strictly regulated, accessible by permission only and in guided tours, and is restricted to 8 months a year. It is possible that its true range would be covered by other protected ares.

##### Conservation actions

Conservation action type: In Place

Conservation actions: 1.1. Land/water protection - Site/area protection1.2. Land/water protection - Resource & habitat protection

#### Other

##### Use and trade

Use type: International

##### Ecosystem services

Ecosystem service type: Important

##### Research needed

Research needed: 1.2. Research - Population size, distribution & trends1.3. Research - Life history & ecology1.5. Research - Threats

Justification for research needed: Research on basic information such as natural history, ecology and possible threats of the species would be needed.

### Troglohyphantes lucifuga

#### Species information

Scientific name: Troglohyphanteslucifuga

Species authority: (Simon, 1884)

Kingdom: Animalia

Phylum: Arthropoda

Class: Arachnida

Order: Araneae

Family: Linyphiidae

Region for assessment: Global

#### Editor & Reviewers

##### Reviewers

Reviewers: Marc MilnePaulo Borges

##### Editor

Editor: Pedro Cardoso

#### Geographic range

Biogeographic realm: Palearctic

Countries: SwitzerlandFranceItaly

Map of records (Google Earth): Suppl. material 38

Basis of EOO and AOO: Species Distribution Model

Basis (narrative): Multiple collection sites are recorded for this species. Therefore, it was possible to perform species distribution modelling to predict its potential range with confidence limits. See Methods for details.

Min Elevation/Depth (m): 392

Max Elevation/Depth (m): 2371

Range description: This species is distributed in Val d’Aosta and northern Piemonte (north-eastern Italy). The presence of this species in Switzerland is testified by historical records in Wallis (Bourg Saint Pierre and Zermatt) and Tessin (Frasco). *Troglohyphanteslucifuga* has also been found in Haute-Savoie (south-eastern France). Conversely, the record of this species in Seine-et-Marne (northern France), is unreliable. Detailed occurrences and relative references in Suppl. material 67.

#### Extent of occurrence

EOO (km2): 11212-15676,13178

Trend: Stable

Justification for trend: This species is not strictly relegated to deep subterranean habitats, being collected in the vicinity of the cave entrance or in shallow subterranean habitats. It is plausible that anthropogenic climate change may affect the habitat suitability of this species. However, in view of the relatively wide thermal tolerance and the relatively high dispersal ability of non-specialised species of *Troglohyphantes* (Mammola et al. 2019a), the distribution range of *T.lucifuga* is not expected to undergo significant reduction in the near future. A deeper study on the impacts of climate change on this species is required.

Causes ceased?: Yes

Causes understood?: Yes

Causes reversible?: Yes

Extreme fluctuations?: No

#### Area of occupancy

Trend: Stable

Justification for trend: This species is not strictly relegated to deep subterranean habitats, being collected in the vicinity of the cave entrance or in shallow subterranean habitats. It is plausible that anthropogenic climate change may affect the habitat suitability of this species. However, in view of the relatively wide thermal tolerance and the relatively high dispersal ability of non-specialised species of *Troglohyphantes* (Mammola et al. 2019a), the distribution range of *T.lucifuga* is not expected to undergo significant reduction in the near future. A deeper study on the impacts of climate change on this species is required.

Causes ceased?: Yes

Causes understood?: Yes

Causes reversible?: Yes

Extreme fluctuations?: No

AOO (km2): 5568-8892,7224

#### Locations

Number of locations: Not applicable

Justification for number of locations: No known major threats to this species.

Trend: Stable

Extreme fluctuations?: No

#### Population

Number of individuals: Unknown

Trend: Stable

Justification for trend: There are no currently known threats to the species.

Causes ceased?: Yes

Causes understood?: Yes

Causes reversible?: Yes

Extreme fluctuations?: No

#### Subpopulations

Number of subpopulations: Unknown

Trend: Unknown

Extreme fluctuations?: No

Severe fragmentation?: No

#### Habitat

System: Terrestrial

Habitat specialist: No

Habitat (narrative): *Troglohyphanteslucifuga* was generally found in debris and among rocks very close to cave entrances, but also in moist shaded places, deep leaf litter, and other shallow subterranean habitats. Cave openings are located in different habitats, such as *Castanea* and *Fagus* woods, alpine screes, rocky lands, and alpine grasslands.

Trend in extent, area or quality?: Stable

Justification for trend: The habitats colonised by *T.lucifuga* are as yet not threatened by direct human activities.

##### Habitat

Habitat importance: Major Importance

Habitats: 1.4. Forest - Temperate7. Caves and Subterranean Habitats (non-aquatic)

#### Ecology

Size: 4.1 mm

Generation length (yr): 2

Dependency of single sp?: No

Ecology and traits (narrative): This spider shows minor specialisation to subterranean life (Mammola et al. 2022a). According to thermal tests, *T.lucifuga* shows a moderate thermal tolerance, reaching 50% mortality at temperature values 7°C above its cave temperature (Mammola et al. 2019a).

#### Threats

Justification for threats: This species is potentially exposed due to its restricted geographic distribution range. However, the existence of threats is unknown for this species.

##### Threats

Threat type: Past

Threats: 12. Other options - Other threat

#### Conservation

Justification for conservation actions: Part of the potential range of this species is inside protected areas and sites of the Natura 2000 network.

##### Conservation actions

Conservation action type: In Place

Conservation actions: 1.1. Land/water protection - Site/area protection1.2. Land/water protection - Resource & habitat protection2.1. Land/water management - Site/area management

#### Other

##### Use and trade

Use type: International

##### Ecosystem services

Ecosystem service type: Important

##### Research needed

Research needed: 1.2. Research - Population size, distribution & trends1.3. Research - Life history & ecology1.5. Research - Threats

Justification for research needed: Research on basic information such as distribution, ecology, life cycle and possible threats throughout the range would be needed.

### Troglohyphantes microcymbium

#### Species information

Scientific name: Troglohyphantesmicrocymbium

Species authority: Pesarini, 2001

Kingdom: Animalia

Phylum: Arthropoda

Class: Arachnida

Order: Araneae

Family: Linyphiidae

Region for assessment: Global

#### Editor & Reviewers

##### Reviewers

Reviewers: Marc MilnePaulo Borges

##### Editor

Editor: Pedro Cardoso

#### Geographic range

Biogeographic realm: Palearctic

Countries: Italy

Map of records (Google Earth): Suppl. material 39

Basis of EOO and AOO: Observed

Basis (narrative): There are only two records known for this subterranean species. It may be possible that the species occurs in other caves in the area. The known distribution range should be taken with caution.

Min Elevation/Depth (m): 559

Max Elevation/Depth (m): 1796

Range description: This species is restricted to two caves in the Bergamasque Prealps (Lombardia, northern Italy): Grotta di Nala di Cà Maquela, in the municipality of Sant'Omobono (Province of Bergamo), and Grotta I Ching, in the municipality of Mandello del Lario (province of Lecco) (see Suppl. material 67).

#### Extent of occurrence

EOO (km2): 8

Trend: Decline (inferred)

Justification for trend: According to Mammola et al. (2018a), climate change will significantly affect the distribution of subterranean specialised *Troglohyphantes* in the future. Moreover, given the low tolerance to habitat changes of these species as well as their very low dispersal ability, a possible extreme reduction of the geographic range of this species is expected in the future.

Causes ceased?: No

Causes understood?: Yes

Causes reversible?: No

Extreme fluctuations?: No

#### Area of occupancy

Trend: Decline (inferred)

Justification for trend: According to Mammola et al. (2018a), climate change will significantly affect the distribution of subterranean specialised *Troglohyphantes* in the future. Moreover, given the low tolerance to habitat changes of these species as well as their very low dispersal ability, a possible extreme reduction of the geographic range of this species is expected in the future.

Causes ceased?: No

Causes understood?: Yes

Causes reversible?: No

Extreme fluctuations?: No

AOO (km2): Unknown

#### Locations

Number of locations: 1

Justification for number of locations: The habitat where this species occurs is affected by changes in subterranean microclimatic conditions due to climate change, which is expected to impact the whole population (see Mammola et al. 2018a).

Trend: Stable

Extreme fluctuations?: No

#### Population

Number of individuals: Unknown

Trend: Decline (inferred)

Justification for trend: The population size is unknown. However, in view of the reduced thermal tolerance of subterranean specialised *Troglohyphantes* species (Mammola et al. 2019a), alterations of the microclimatic conditions of the habitat due to climate change are expected to impact the whole population of this species.

Basis for decline: (c) a decline in area of occupancy, extent of occurrence and/or quality of habitat

Causes ceased?: No

Causes understood?: Yes

Causes reversible?: No

Extreme fluctuations?: No

#### Subpopulations

Number of subpopulations: Unknown

Trend: Unknown

Extreme fluctuations?: No

Severe fragmentation?: No

#### Habitat

System: Terrestrial

Habitat specialist: Yes

Habitat (narrative): There is poor information on species habitat. Specimens have been collected in caves.

Trend in extent, area or quality?: Decline (inferred)

Justification for trend: As seen in Mammola et al. (2018a) for the *Troglohyphantes* species of the Western Alps, a drastic decline in the habitat suitability of *T.microcymbium* as a consequence of climate change is expected.

##### Habitat

Habitat importance: Major Importance

Habitats: 7. Caves and Subterranean Habitats (non-aquatic)

#### Ecology

Size: 2.8 mm

Generation length (yr): 4

Dependency of single sp?: No

Ecology and traits (narrative): This species shows a high degree of adaptation to deep subterranean habitats, with absence of pigmentation and pronounced eye regression (Mammola et al. 2022a).

#### Threats

Justification for threats: As seen for other subterranean specialised species of *Troglohyphantes* of the Western Alps (Mammola et al. 2018a), climate warming is expected to reduce the currently suitable habitat for this spider.

##### Threats

Threat type: Future

Threats: 11. Climate change & severe weather11.1. Climate change & severe weather - Habitat shifting & alteration11.2. Climate change & severe weather - Droughts11.3. Climate change & severe weather - Temperature extremes

#### Conservation

Justification for conservation actions: Part of the ditribution range of this species falls within the Regional Park of Grigna Settentrionale (SAC IT2030001).

##### Conservation actions

Conservation action type: In Place

Conservation actions: 1.1. Land/water protection - Site/area protection1.2. Land/water protection - Resource & habitat protection2.1. Land/water management - Site/area management

#### Other

##### Use and trade

Use type: International

##### Ecosystem services

Ecosystem service type: Important

##### Research needed

Research needed: 1.2. Research - Population size, distribution & trends1.3. Research - Life history & ecology1.5. Research - Threats

Justification for research needed: Research on basic information such as distribution, ecology, life cycle and possible threats throughout the range would be needed.

### Troglohyphantes nigraerosae

#### Species information

Scientific name: Troglohyphantesnigraerosae

Species authority: Brignoli, 1971

Kingdom: Animalia

Phylum: Arthropoda

Class: Arachnida

Order: Araneae

Family: Linyphiidae

Region for assessment: Global

#### Editor & Reviewers

##### Reviewers

Reviewers: Marc MilnePaulo Borges

##### Editor

Editor: Pedro Cardoso

#### Geographic range

Biogeographic realm: Palearctic

Countries: Italy

Map of records (Google Earth): Suppl. material 40

Basis of EOO and AOO: Observed

Basis (narrative): Caves in Western Alps have been extensively sampled, allowing to define EOO and AOO of this species with reasonable confidence.

Min Elevation/Depth (m): 1097

Max Elevation/Depth (m): 2900

Range description: This species has been recorded in some hypogean localities of the Graian Alps (Gran Paradiso Massif and Lanzo Valleys in province of Torino, and Valle dell'Elvo in province of Biella, Piemonte), and in an epigeic locality in Colle dell’Arietta (detailed occurrences and relative references in Suppl. material 67).

#### Extent of occurrence

EOO (km2): 498

Trend: Decline (inferred)

Justification for trend: As seen in Mammola et al. (2018a) for all the subterranean specialised *Troglohyphantes* species of the Western Alps, climate change is expected to affect the distribution of this species in the future. Given the reduced thermal tolerance of this organism and its low dispersal ability (Mammola et al. 2019a, Mammola et al. 2022a), a reduction of its geographic distribution range is expected in the future.

Causes ceased?: No

Causes understood?: Yes

Causes reversible?: No

Extreme fluctuations?: No

#### Area of occupancy

Trend: Decline (inferred)

Justification for trend: As seen in Mammola et al. (2018a) for all the subterranean specialised *Troglohyphantes* species of the Western Alps, climate change is expected to affect the distribution of this species in the future. Given the reduced thermal tolerance of this organism and its low dispersal ability (Mammola et al. 2019a, Mammola et al. 2022a), a reduction of its geographic distribution range is expected in the future.

Causes ceased?: No

Causes understood?: Yes

Causes reversible?: No

Extreme fluctuations?: No

AOO (km2): 28

#### Locations

Number of locations: 1

Justification for number of locations: The habitat where this species occurs is affected by changes in subterranean microclimatic conditions due to climate change, which is expected to impact the whole population (see Mammola et al. 2018a).

Trend: Stable

Extreme fluctuations?: No

#### Population

Number of individuals: Unknown

Trend: Decline (inferred)

Justification for trend: In view of the reduced thermal tolerance of this species (Mammola et al. 2019a), alterations of the microclimatic conditions of the habitat due to climate change are expected to impact the whole population of this species.

Basis for decline: (c) a decline in area of occupancy, extent of occurrence and/or quality of habitat

Causes ceased?: No

Causes understood?: Yes

Causes reversible?: No

Extreme fluctuations?: No

#### Subpopulations

Number of subpopulations: 7

Trend: Decline (inferred)

Justification for trend: Because of the adaptation to the subterranean habitat and of the narrow therrmal tolerance of this species, likely hampering dispersal through non-subterranean habitats, each locality can reasonably host a single isolated subpopulation. Accordingly, for this species we identified seven subpopulations occurring in the Graian Alps, north-western Italy. All these subpopulations are likely to be impacted by climate change.

Extreme fluctuations?: No

Severe fragmentation?: No

#### Habitat

System: Terrestrial

Habitat specialist: No

Habitat (narrative): Specimens have been found hanging on webs on cave walls. All records refer to cold caves, ranging from 2°C to maximum 6°C mean annual temperature. Holotype comes from an epigeic locality at 2,900 m. Most of the cave openings are located in alpine screes or *Fagus* woods.

Trend in extent, area or quality?: Decline (inferred)

Justification for trend: As seen in Mammola et al. (2018a) for other species of the genus *Troglohyphantes* of the Western Alps, a drastic decline in the habitat suitability of *T.nigraerosae* as a consequence of climate change is expected

##### Habitat

Habitat importance: Major Importance

Habitats: 6. Rocky areas (e.g. inland cliffs, mountain peaks)7. Caves and Subterranean Habitats (non-aquatic)

#### Ecology

Size: 3.3 mm

Generation length (yr): 4

Dependency of single sp?: No

Ecology and traits (narrative): This spider lives mainly on webs on cave walls. Specimens show a moderate degree of morphological specialisation, with absence of pigmentation and eye regression (Mammola et al. 2022a). According to thermal tests, *T.nigraerosae* shows a narrow thermal tolerance, reaching 50% mortality at temperature values 2°C above its cave temperature (Mammola et al. 2019a).

#### Threats

Justification for threats: This species is potentially exposed due to its narrow geographic distribution range. As seen for other western alpine species of *Troglohyphantes* (Mammola et al. 2018a), climate warming is expected to reduce the currently suitable habitat for this spider. Moreover, in view of its reduced thermal tolerance (Mammola et al. 2019a), this species has a limited dispersal ability, which represents an additional concern in face of the ongoing increase of temperature.

##### Threats

Threat type: Future

Threats: 11.1. Climate change & severe weather - Habitat shifting & alteration11.2. Climate change & severe weather - Droughts11.3. Climate change & severe weather - Temperature extremes

#### Conservation

Justification for conservation actions: Part of the distribution range of this species is included in the National Park, Special Area of Conservation and Special Protected Area of Gran Paradiso (EUAP0006 and SAC/SPA IT1201000).

##### Conservation actions

Conservation action type: In Place

Conservation actions: 1.1. Land/water protection - Site/area protection1.2. Land/water protection - Resource & habitat protection2.1. Land/water management - Site/area management

#### Other

##### Use and trade

Use type: International

##### Ecosystem services

Ecosystem service type: Important

##### Research needed

Research needed: 1.2. Research - Population size, distribution & trends1.3. Research - Life history & ecology1.5. Research - Threats

Justification for research needed: Research on basic information such as distribution, ecology, life cycle and possible threats throughout the range would be needed.

### Troglohyphantes noricus

#### Species information

Scientific name: Troglohyphantesnoricus

Species authority: (Thaler & Polenec, 1974)

Kingdom: Animalia

Phylum: Arthropoda

Class: Arachnida

Order: Araneae

Family: Linyphiidae

**Figure 10.**
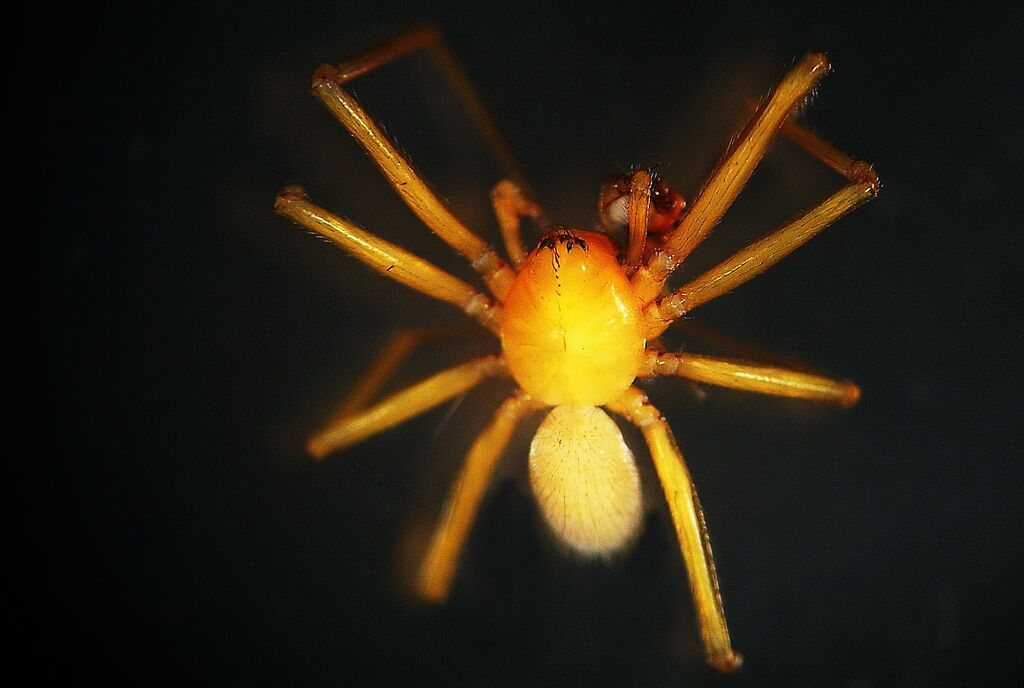
*Troglohyphantesnoricus* (Thaler & Polenec, 1974). Photo credit: Christian Komposch.

Figure(s) or Photo(s): Fig. 10

Region for assessment: Global

#### Editor & Reviewers

##### Reviewers

Reviewers: Marc MilnePaulo Borges

##### Editor

Editor: Pedro Cardoso

#### Geographic range

Biogeographic realm: Palearctic

Countries: GermanyAustria

Map of records (Google Earth): Suppl. material 41

Basis of EOO and AOO: Species Distribution Model

Basis (narrative): Multiple collection sites are recorded for this species. Therefore, it was possible to perform species distribution modelling to predict its potential range with confidence limits. See Methods for details.

Min Elevation/Depth (m): 290

Max Elevation/Depth (m): 2109

Range description: *Troglohyphantesnoricus* has been frequently recorded in Austria. It is also known from Bavaria, in southern Germany (detailed occurrences and relative references in Suppl. material 67).

#### Extent of occurrence

EOO (km2): 17353-34801,21001

Trend: Stable

Justification for trend: This species has been mainly collected in forests and alpine prairies. Many of the environments that this spider inhabits are affected by forestry and silvicultural measures, and by land use change due to agriculture and road infrastructure development (Komposch 2009). In addition, it is plausible that anthropogenic climate change may affect the habitat suitability of this species. However, in view of the relatively wide thermal tolerance and the relatively high dispersal ability of non-specialised species of *Troglohyphantes* (Mammola et al. 2019a), the distribution range of *T.noricus* is not expected to undergo significant reduction in the near future. A deeper study on the impacts of climate change on this species is required.

Causes ceased?: Yes

Causes understood?: Yes

Causes reversible?: Yes

Extreme fluctuations?: No

#### Area of occupancy

Trend: Stable

Justification for trend: This species has been mainly collected in forests and alpine prairies. Many of the environments that this spider inhabits are affected by forestry and silvicultural measures, and by land use change due to agriculture and road infrastructure development (Komposch 2009). In addition, it is plausible that anthropogenic climate change may affect the habitat suitability of this species. However, in view of the relatively wide thermal tolerance and the relatively high dispersal ability of non-specialised species of *Troglohyphantes* (Mammola et al. 2019a), the distribution range of *T.noricus* is not expected to undergo significant reduction in the near future. A deeper study on the impacts of climate change on this species is required.

Causes ceased?: Yes

Causes understood?: Yes

Causes reversible?: Yes

Extreme fluctuations?: No

AOO (km2): 7844-24048,12432

#### Locations

Number of locations: Not applicable

Justification for number of locations: No known major threats to this species.

Trend: Stable

Extreme fluctuations?: No

#### Population

Number of individuals: Unknown

Trend: Stable

Justification for trend: There are no currently known threats to the species.

Causes ceased?: Yes

Causes understood?: Yes

Causes reversible?: Yes

Extreme fluctuations?: No

#### Subpopulations

Number of subpopulations: Unknown

Trend: Unknown

Extreme fluctuations?: No

Severe fragmentation?: No

#### Habitat

System: Terrestrial

Habitat specialist: No

Habitat (narrative): Specimens of *T.noricus* occur under dead wood, in moist soil, in small mammal burrows, and in deep leaf layers of mixed beech-fir and beech-oak forests, of ravine forests, rocky gorges, spring meadows and avalanche erosion gullies, which have a microclimatically favourable crevice system in the soil due to blocks, rocky elements, or deadwood. In addition, some records have been collected in larch-spruce forests at higher altitudes. In the Berchtesgaden Alps and in the Tennen Mountains, this species also lives in the alpine belt far above the timberline.

Trend in extent, area or quality?: Stable

Justification for trend: Even though some of the habitats where this species occurs are affected by land use change (Komposch 2009), *T.noricus* has a wide geographic and altimetric range and seems to adapt to various habitat types, and therefore is not expected to experience any decline.

##### Habitat

Habitat importance: Major Importance

Habitats: 1.4. Forest - Temperate3.4. Shrubland - Temperate4.4. Grassland - Temperate6. Rocky areas (e.g. inland cliffs, mountain peaks)7.2. Caves and Subterranean Habitats (non-aquatic) - Other Subterranean Habitats

#### Ecology

Size: 3.4 mm

Generation length (yr): 2

Dependency of single sp?: No

Ecology and traits (narrative): Not much is known about the ecology and life history of this species. This spider shows a lower degree of specialisation to subterranean life (Mammola et al. 2022a).

#### Threats

Justification for threats: No known major threats to this species.

##### Threats

Threat type: Past

Threats: 12. Other options - Other threat

#### Conservation

Justification for conservation actions: There are several national parks, protected areas, and sites of the Natura 2000 network within the potential geographic range of the species. In Germany, *T.noricus* is listed in the category R of the national Red List of spiders (Blick et al. 2016) and in the regional Red List of Bavaria (Blick and Scheidler 2004).

##### Conservation actions

Conservation action type: In Place

Conservation actions: 1.1. Land/water protection - Site/area protection1.2. Land/water protection - Resource & habitat protection2.1. Land/water management - Site/area management

#### Other

##### Use and trade

Use type: International

##### Ecosystem services

Ecosystem service type: Important

##### Research needed

Research needed: 1.2. Research - Population size, distribution & trends1.3. Research - Life history & ecology1.5. Research - Threats

Justification for research needed: Research on basic information such as distribution, ecology, life cycle and possible threats throughout the range would be needed.

### Troglohyphantes novicordis

#### Species information

Scientific name: Troglohyphantes novicordis

Species authority: Thaler, 1978

Kingdom: Animalia

Phylum: Arthropoda

Class: Arachnida

Order: Araneae

Family: Linyphiidae

Region for assessment: Global

#### Editor & Reviewers

##### Reviewers

Reviewers: Marc MilnePaulo Borges

##### Editor

Editor: Pedro Cardoso

#### Geographic range

Biogeographic realm: Palearctic

Countries: SloveniaAustria

Map of records (Google Earth): Suppl. material 42

Basis of EOO and AOO: Observed

Basis (narrative): This species was collected in very few localities. Its low level of subterranean specialisation, together with the high altimetric range found in its known distribution, possibly reflects a higher dispersal capacity when compared to subterranean specialised species. Consequently, it may be possible that the present known range of this species is underestimated.

Min Elevation/Depth (m): 550

Max Elevation/Depth (m): 1924

Range description: This rare species is known from one cave (Raudnerhöhle) and a few epigean localities in Styria and Carinthia (southern Austria), and from one cave in an unspecified locality in northern Slovenia (detailed occurrences and relative references in Suppl. material 67).

#### Extent of occurrence

EOO (km2): 756

Trend: Stable

Justification for trend: This species is not strictly relegated to deep subterranean habitats, being collected both in hypogean and epigean localities. It is plausible that anthropogenic climate change may affect the habitat suitability of this species. However, in view of the relatively wide thermal tolerance and the relatively high dispersal ability of non-specialised species of *Troglohyphantes* (Mammola et al. 2019a), the distribution range of *T.novicordis* is not expected to undergo significant reduction in the near future. A deeper study on the current distribution of this species and on the potential impacts of climate change is required.

Causes ceased?: Yes

Causes understood?: Yes

Causes reversible?: Yes

Extreme fluctuations?: No

#### Area of occupancy

Trend: Stable

Justification for trend: This species is not strictly relegated to deep subterranean habitats, being collected both in hypogean and epigean localities. It is plausible that anthropogenic climate change may affect the habitat suitability of this species. However, in view of the relatively wide thermal tolerance and the relatively high dispersal ability of non-specialised species of *Troglohyphantes* (Mammola et al. 2019a), the distribution range of *T.novicordis* is not expected to undergo significant reduction in the near future. A deeper study on the current distribution of this species and on the potential impacts of climate change is required.

Causes ceased?: Yes

Causes understood?: Yes

Causes reversible?: Yes

Extreme fluctuations?: No

AOO (km2): 20

#### Locations

Number of locations: Not applicable

Justification for number of locations: No known major threats to this species.

Trend: Stable

Extreme fluctuations?: No

#### Population

Number of individuals: Unknown

Trend: Stable

Justification for trend: There are no currently known threats to this species.

Causes ceased?: Yes

Causes understood?: Yes

Causes reversible?: Yes

Extreme fluctuations?: No

#### Subpopulations

Number of subpopulations: Unknown

Trend: Unknown

Extreme fluctuations?: No

Severe fragmentation?: No

#### Habitat

System: Terrestrial

Habitat specialist: No

Habitat (narrative): In the type locality, *T.novicordis* has been found in hypogean habitat. Other records have been collected in moss and under the rocks in spruce forests, and in high altitude shrublands and rocky lands.

Trend in extent, area or quality?: Unknown

##### Habitat

Habitat importance: Major Importance

Habitats: 1.4. Forest - Temperate3.4. Shrubland - Temperate6. Rocky areas (e.g. inland cliffs, mountain peaks)7. Caves and Subterranean Habitats (non-aquatic)

#### Ecology

Size: 2.7 mm

Generation length (yr): 2

Dependency of single sp?: No

Ecology and traits (narrative): This spider shows minor specialisation to subterranean life (Mammola et al. 2022a).

#### Threats

Justification for threats: The existence of threats for this species is unknown.

##### Threats

Threat type: Past

Threats: 12. Other options - Other threat

#### Conservation

Justification for conservation actions: This species has been listed in the 59th Regulation of the Carinthian State Government of 2015 (LGBl. Nr. 59/2015), among the species fully protected according to the Carinthian Nature Conservation Act 2002 (LGBl. Nr. 79/2002).

##### Conservation actions

Conservation action type: In Place

#### Other

##### Use and trade

Use type: International

##### Ecosystem services

Ecosystem service type: Important

##### Research needed

Research needed: 1.2. Research - Population size, distribution & trends1.3. Research - Life history & ecology1.5. Research - Threats

Justification for research needed: Basic research is needed to know the current distribution, population size and trends, ecology and traits of this species, and its possible threats.

### Troglohyphantes pavesii

#### Species information

Scientific name: Troglohyphantespavesii

Species authority: Pesarini, 1988

Kingdom: Animalia

Phylum: Arthropoda

Class: Arachnida

Order: Araneae

Family: Linyphiidae

Region for assessment: Global

#### Editor & Reviewers

##### Reviewers

Reviewers: Marc MilnePaulo Borges

##### Editor

Editor: Pedro Cardoso

#### Geographic range

Biogeographic realm: Palearctic

Countries: Italy

Map of records (Google Earth): Suppl. material 43

Basis of EOO and AOO: Unknown

Basis (narrative): This species was collected in two localities. Its low level of subterranean specialisation, possibly reflects a higher dispersal capacity when compared to subterranean specialised species. It may be possible that the species occurs in other localities in the area. The true range is therefore unknown and not possible to model with confidence.

Min Elevation/Depth (m): 373

Max Elevation/Depth (m): 900

Range description: This species was previously recorded only from the type locality in the Monte Ragogna, municipality of Muris (province of Udine, Friuli-Venezia Giulia, north-eastern Italy). More recently, this species was found also in Lavina, municipality of Tambre (province of Belluno, Veneto, north-eastern Italy), extending considerably its distribution westwards (detailed occurrences and relative references in Suppl. material 67). The female is still unknown (Isaia and Pantini 2010).

#### Extent of occurrence

EOO (km2): Unknown

Trend: Unknown

Causes ceased?: Unknown

Causes understood?: Unknown

Causes reversible?: Unknown

Extreme fluctuations?: No

#### Area of occupancy

Trend: Unknown

Causes ceased?: Unknown

Causes understood?: Unknown

Causes reversible?: Unknown

Extreme fluctuations?: No

AOO (km2): Unknown

#### Locations

Number of locations: Unknown

Justification for number of locations: The data available are not enough to estimate the number of locations for this species.

Trend: Unknown

Extreme fluctuations?: No

#### Population

Number of individuals: Unknown

Trend: Unknown

Justification for trend: The population size and trend are unknown.

Causes ceased?: Unknown

Causes understood?: Unknown

Causes reversible?: Unknown

Extreme fluctuations?: No

#### Subpopulations

Number of subpopulations: Unknown

Trend: Unknown

Extreme fluctuations?: No

Severe fragmentation?: No

#### Habitat

System: Terrestrial

Habitat specialist: No

Habitat (narrative): This species has been found in epigean habitats. No additional information on the habitat has been provided.

Trend in extent, area or quality?: Unknown

##### Habitat

Habitat importance: Major Importance

Habitats: 1.4. Forest - Temperate

#### Ecology

Size: 3 mm

Generation length (yr): 2

Dependency of single sp?: No

Ecology and traits (narrative): Specimens lack pigmentation (Mammola et al. 2022a). The female of this species is unknown.

#### Threats

Justification for threats: Unknown threats.

##### Threats

Threat type: Past

Threats: 12. Other options - Other threat

#### Other

##### Use and trade

Use type: International

##### Ecosystem services

Ecosystem service type: Important

##### Research needed

Research needed: 1.2. Research - Population size, distribution & trends1.3. Research - Life history & ecology1.5. Research - Threats

Justification for research needed: Research on basic information such as distribution, ecology, life cycle and possible threats throughout the range would be needed.

### Troglohyphantes pedemontanus

#### Species information

Scientific name: Troglohyphantespedemontanus

Species authority: (Gozo, 1908)

Kingdom: Animalia

Phylum: Arthropoda

Class: Arachnida

Order: Araneae

Family: Linyphiidae

**Figure 11.**
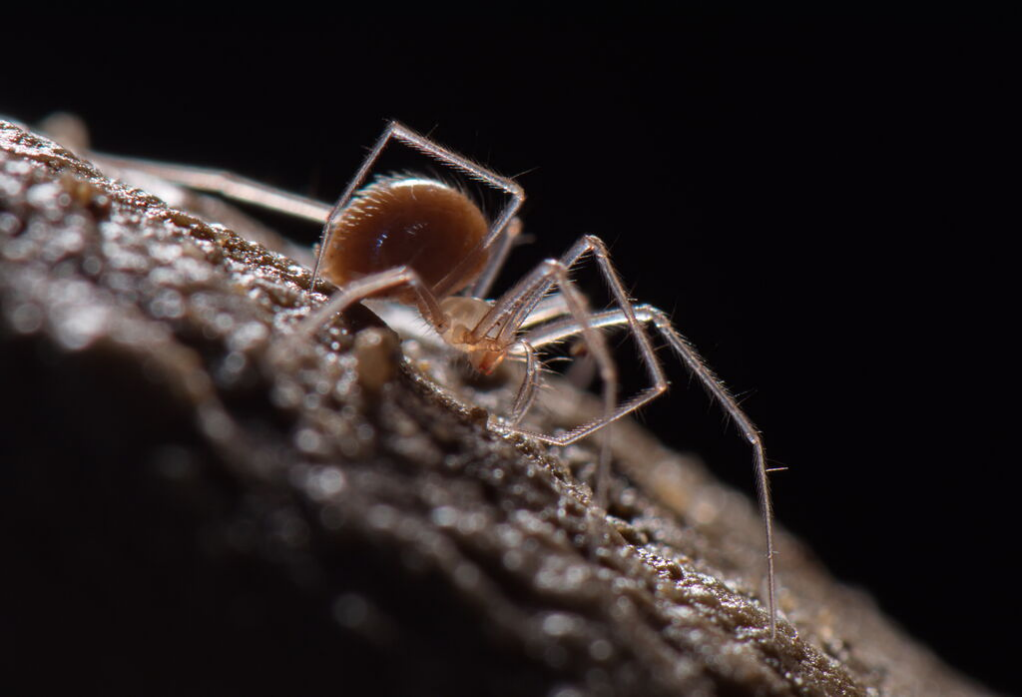
*Troglohyphantespedemontanus* (Gozo, 1908). Photo credit: Francesco Tomasinelli.

Figure(s) or Photo(s): Fig. 11

Region for assessment: Global

#### Editor & Reviewers

##### Reviewers

Reviewers: Marc MilnePaulo Borges

##### Editor

Editor: Pedro Cardoso

#### Geographic range

Biogeographic realm: Palearctic

Countries: Italy

Map of records (Google Earth): Suppl. material 44

Basis of EOO and AOO: Observed

Basis (narrative): Caves in Southwestern Alps have been extensively sampled, allowing to define EOO and AOO of this species with reasonable confidence.

Min Elevation/Depth (m): 854

Max Elevation/Depth (m): 971

Range description: This subterranean specialised species is restricted to three caves in Piemonte (north-western Italy), namely Grotta di Bossea and Pozzo del Rospo in Corsaglia Valley, and Pozzo del Villaretto in Tanaro Valley (see Suppl. material 67).

#### Extent of occurrence

EOO (km2): 46

Trend: Decline (inferred)

Justification for trend: As seen in Mammola et al. (2018a) for all the subterranean specialised *Troglohyphantes* species of the Western Alps, climate change is expected to affect the distribution of this species in the future. Given the reduced thermal tolerance of this organism and its low dispersal ability (Mammola et al. 2019a), a reduction of its geographic distribution range is expected in the future.

Causes ceased?: No

Causes understood?: Yes

Causes reversible?: No

Extreme fluctuations?: No

#### Area of occupancy

Trend: Decline (inferred)

Justification for trend: As seen in Mammola et al. (2018a) for all the subterranean specialised *Troglohyphantes* species of the Western Alps, climate change is expected to affect the distribution of this species in the future. Given the reduced thermal tolerance of this organism and its low dispersal ability (Mammola et al. 2019a), a reduction of its geographic distribution range is expected in the future.

Causes ceased?: No

Causes understood?: Yes

Causes reversible?: No

Extreme fluctuations?: No

AOO (km2): 12

#### Locations

Number of locations: 1

Justification for number of locations: Even though this species occurs in different caves, these are interpreted as a single location, as they are all affected by changes in subterranean microclimatic conditions due to climate change.

Trend: Stable

Extreme fluctuations?: No

#### Population

Number of individuals: Unknown

Trend: Decline (inferred)

Justification for trend: In view of the reduced thermal tolerance of this species (Mammola et al. 2019a), alterations of the microclimatic conditions of the habitat due to climate change are expected to impact the whole population of this species.

Basis for decline: (c) a decline in area of occupancy, extent of occurrence and/or quality of habitat

Causes ceased?: No

Causes understood?: Yes

Causes reversible?: No

Extreme fluctuations?: No

#### Subpopulations

Number of subpopulations: 3

Trend: Decline (inferred)

Justification for trend: Because of the adaptation to the subterranean habitat and the narrow thermal tolerance of this species, likely hampering dispersal through non-subterranean habitats, each locality can reasonably host a single isolated subpopulation. Accordingly, for this species we identified three subpopulations occurring in the province of Cuneo (Piemonte, north-western Italy). All the subpopulations are likely to be impacted by climate change.

Extreme fluctuations?: No

Severe fragmentation?: No

#### Habitat

System: Terrestrial

Habitat specialist: Yes

Habitat (narrative): Specimens have been found in the deepest part of the cave among stony debris on the ground or on webs among stalactites. Cave openings are located in beech woods.

Trend in extent, area or quality?: Decline (inferred)

Justification for trend: As seen in Mammola et al. (2018a) for other *Troglohyphantes* species of the Western Alps, a drastic decline in the habitat suitability of *T.pedemontanus* as a consequence of climate change is expected.

##### Habitat

Habitat importance: Major Importance

Habitats: 7. Caves and Subterranean Habitats (non-aquatic)

#### Ecology

Size: 2.9 mm

Generation length (yr): 4

Dependency of single sp?: No

Ecology and traits (narrative): This subterranean specialised species shows pronounced eye reduction and absence of abdominal pigmentation (Mammola et al. 2022a). According to thermal tests, *T.pedemontanus* shows a narrow thermal tolerance, reaching 50% mortality at temperature values 4°C above its cave temperature (Mammola et al. 2019a).

#### Threats

Justification for threats: This species is potentially exposed due to its extremely narrow geographic distribution range. As seen for other western alpine species of the genus *Troglohyphantes* (Mammola et al. 2018a), climate warming is expected to reduce the currently suitable habitat for this spider. Moreover, in view of its reduced thermal tolerance (Mammola et al. 2019a), this species has a limited dispersal ability, which represents an additional concern in face of the ongoing increase of temperature. One of the caves where the species occurs (Grotta di Bossea) is opened to tourism (approximately 16,000 tourists per year). Consequently, a secondary impact for this subpopulation could derive from touristic activities.

##### Threats

Threat type: Future

Threats: 11.1. Climate change & severe weather - Habitat shifting & alteration11.2. Climate change & severe weather - Droughts11.3. Climate change & severe weather - Temperature extremes

##### Threats

Threat type: Ongoing

Threats: 1.3. Residential & commercial development - Tourism & recreation areas6.1. Human intrusions & disturbance - Recreational activities

#### Conservation

Justification for conservation actions: Part of the distribution of this species is included in a Special Area of Conservation (SAC IT1160026 Grotte di Bossea). Management of the subpopulation occurring in the touristic cave would be needed by means of a strict code of conduct for the activities in caves, and of both communication to the general public and training of touristic agents.

##### Conservation actions

Conservation action type: In Place

Conservation actions: 1.1. Land/water protection - Site/area protection

##### Conservation actions

Conservation action type: Needed

Conservation actions: 2.1. Land/water management - Site/area management4. Education & awareness

#### Other

##### Use and trade

Use type: International

##### Ecosystem services

Ecosystem service type: Important

##### Research needed

Research needed: 1.2. Research - Population size, distribution & trends1.3. Research - Life history & ecology1.5. Research - Threats3.1. Monitoring - Population trends3.4. Monitoring - Habitat trends

Justification for research needed: Research on basic information such as distribution, natural history, ecology and possible threats of the species would be needed. Monitoring of population and habitat are important to confirm projected trends.

### Troglohyphantes pluto

#### Species information

Scientific name: Troglohyphantespluto

Species authority: Caporiacco, 1938

Kingdom: Animalia

Phylum: Arthropoda

Class: Arachnida

Order: Araneae

Family: Linyphiidae

**Figure 12.**
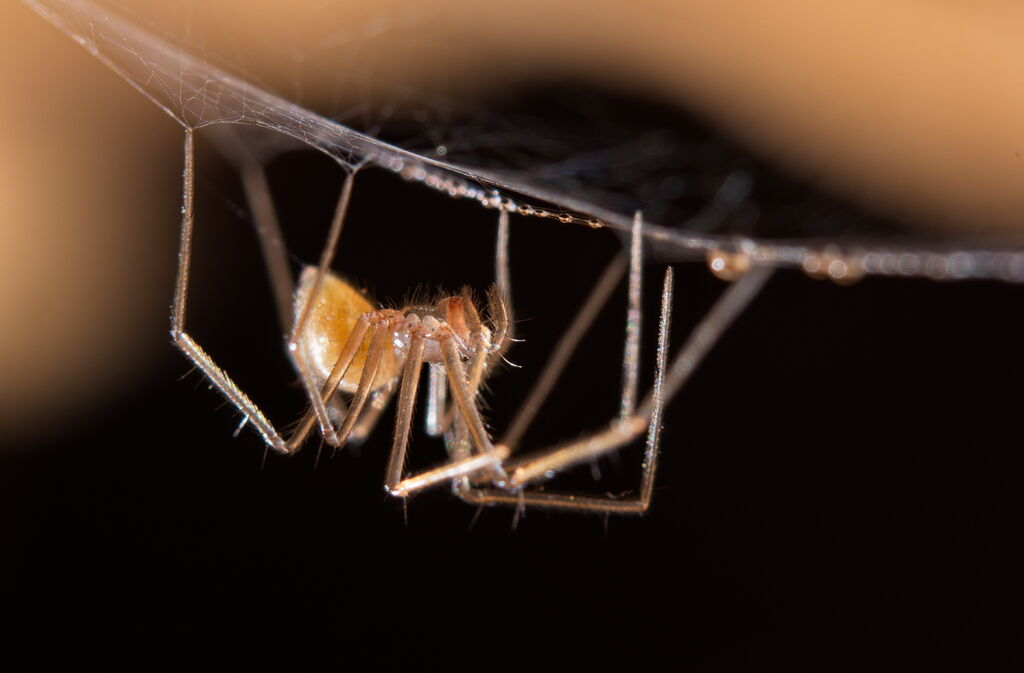
*Troglohyphantespluto* Caporiacco, 1938. Photo credit: Francesco Tomasinelli.

Figure(s) or Photo(s): Fig. 12

Region for assessment: Global

#### Editor & Reviewers

##### Reviewers

Reviewers: Marc MilnePaulo Borges

##### Editor

Editor: Pedro Cardoso

#### Geographic range

Biogeographic realm: Palearctic

Countries: Italy

Map of records (Google Earth): Suppl. material 45

Basis of EOO and AOO: Observed

Basis (narrative): This non-specialised species was collected in a few localities. Its low level of subterranean specialisation, together with the high altimetric range found in its known distribution, possibly reflects a higher dispersal capacity when compared to subterranean specialised species. Consequently, it may be possible that the present known range of this species is underestimated.

Min Elevation/Depth (m): 798

Max Elevation/Depth (m): 2236

Range description: This species has been found in some caves of Corsaglia Valley and the high Tanaro Valley (province of Cuneo, Piemonte, north-western Italy) (detailed occurrences and relative references in Suppl. material 67).

#### Extent of occurrence

EOO (km2): 98

Trend: Stable

Justification for trend: This species is not strictly relegated to deep subterranean habitats. It is plausible that anthropogenic climate change may affect the habitat suitability of this species. However, in view of the relatively wide thermal tolerance and the relatively high dispersal ability of non-specialised species of *Troglohyphantes* (Mammola et al. 2019a), the distribution range of *T.pluto* is not expected to undergo significant reduction in the near future. A deeper study on the current distribution of this species and on the potential impacts of climate change is required.

Causes ceased?: Yes

Causes understood?: Yes

Causes reversible?: Yes

Extreme fluctuations?: No

#### Area of occupancy

Trend: Stable

Justification for trend: This species is not strictly relegated to deep subterranean habitats. It is plausible that anthropogenic climate change may affect the habitat suitability of this species. However, in view of the relatively wide thermal tolerance and the relatively high dispersal ability of non-specialised species of *Troglohyphantes* (Mammola et al. 2019a), the distribution range of *T.pluto* is not expected to undergo significant reduction in the near future. A deeper study on the current distribution of this species and on the potential impacts of climate change is required.

Causes ceased?: Yes

Causes understood?: Yes

Causes reversible?: Yes

Extreme fluctuations?: No

AOO (km2): 28

#### Locations

Number of locations: Not applicable

Justification for number of locations: There are no currently known threats to this species.

Trend: Stable

Extreme fluctuations?: No

#### Population

Number of individuals: Unknown

Trend: Stable

Justification for trend: There are no currently known threats to the species.

Causes ceased?: Yes

Causes understood?: Yes

Causes reversible?: Yes

Extreme fluctuations?: No

#### Subpopulations

Number of subpopulations: Unknown

Trend: Unknown

Extreme fluctuations?: No

Severe fragmentation?: No

#### Habitat

System: Terrestrial

Habitat specialist: No

Habitat (narrative): This species has been mostly collected in the nearby of the cave entrance. However, in several cases, specimens have been also observed in true hypogean habitats. Caves hosting this species encompass a broad altitudinal gradient, from roughly 800 to more than 2,000 m. The population of Balma Ghiacciata del Mondolé is found in the twilight zone of the cave. The cave is characterised by very cool microclimatic condition, sustaining a perennial snowfield near the entrance (Isaia et al. 2017).

Trend in extent, area or quality?: Unknown

##### Habitat

Habitat importance: Major Importance

Habitats: 1.4. Forest - Temperate7. Caves and Subterranean Habitats (non-aquatic)

#### Ecology

Size: 3.7 mm

Generation length (yr): 2

Dependency of single sp?: No

Ecology and traits (narrative): This spider shows minor morphological specialisation to the subterranean habitat, with partially pigmented abdominal pattern and eyes normally developed (Mammola et al. 2022a). According to thermal tests, *T.pluto* shows a narrow thermal tolerance, reaching 50% mortality at temperature values 4°C above its cave temperature (Mammola et al. 2019a).

#### Threats

Justification for threats: The existence of threats is unknown for this species. One of the caves where the species occurs (Grotte del Caudano) is opened to tourism (approximately 3,000 tourists per year). Consequently, a potential impact for this subpopulation could derive from touristic activities.

##### Threats

Threat type: Past

Threats: 12. Other options - Other threat

#### Other

##### Use and trade

Use type: International

##### Ecosystem services

Ecosystem service type: Important

##### Research needed

Research needed: 1.2. Research - Population size, distribution & trends1.3. Research - Life history & ecology1.5. Research - Threats

Justification for research needed: Research on basic information such as distribution, natural history, ecology and possible threats of the species would be needed.

### Troglohyphantes poleneci

#### Species information

Scientific name: Troglohyphantespoleneci

Species authority: Wiehle, 1964

Kingdom: Animalia

Phylum: Arthropoda

Class: Arachnida

Order: Araneae

Family: Linyphiidae

Region for assessment: Global

#### Editor & Reviewers

##### Reviewers

Reviewers: Marc MilnePaulo Borges

##### Editor

Editor: Pedro Cardoso

#### Geographic range

Biogeographic realm: Palearctic

Countries: SloveniaAustriaItaly

Map of records (Google Earth): Suppl. material 46

Basis of EOO and AOO: Species Distribution Model

Basis (narrative): Multiple sites are recorded for this non-specialised species. Therefore, it was possible to perform species distribution modelling to predict its potential range with confidence limits. See Methods for details.

Min Elevation/Depth (m): 315

Max Elevation/Depth (m): 1754

Range description: This species has a widespread distributional range in Upper Carniola (Slovenia). It has also been recorded in Carinthia (southern Austria) and in Friuli-Venezia Giulia (north-eastern Italy) (detailed occurrences and relative references in Suppl. material 67).

#### Extent of occurrence

EOO (km2): 7766-10026,8752

Trend: Stable

Justification for trend: *Troglohyphantespoleneci* has been mainly collected in epigean habitat. It is plausible that anthropogenic climate change may affect the habitat suitability of this species. However, in view of the relatively wide thermal tolerance and the relatively high dispersal ability of non-specialised species of *Troglohyphantes* (Mammola et al. 2019a), the distribution range of *T.poleneci* is not expected to undergo significant reduction in the near future. A deeper study on the impacts of climate change on this species is required.

Causes ceased?: Yes

Causes understood?: Yes

Causes reversible?: Yes

Extreme fluctuations?: No

#### Area of occupancy

Trend: Stable

Justification for trend: *Troglohyphantespoleneci* has been mainly collected in epigean habitat. It is plausible that anthropogenic climate change may affect the habitat suitability of this species. However, in view of the relatively wide thermal tolerance and the relatively high dispersal ability of non-specialised species of *Troglohyphantes* (Mammola et al. 2019a), the distribution range of *T.poleneci* is not expected to undergo significant reduction in the near future. A deeper study on the impacts of climate change on this species is required.

Causes ceased?: Yes

Causes understood?: Yes

Causes reversible?: Yes

Extreme fluctuations?: No

AOO (km2): 4420-6600,5280

#### Locations

Number of locations: Not applicable

Justification for number of locations: No known threats to this species.

Trend: Stable

Extreme fluctuations?: No

#### Population

Number of individuals: Unknown

Trend: Stable

Justification for trend: There are no currently known threats to the species.

Causes ceased?: Yes

Causes understood?: Yes

Causes reversible?: Yes

Extreme fluctuations?: No

#### Subpopulations

Number of subpopulations: Unknown

Trend: Unknown

Extreme fluctuations?: No

Severe fragmentation?: No

#### Habitat

System: Terrestrial

Habitat specialist: No

Habitat (narrative): This species has been mainly found in beech and mixed beech-fir forests, in small burrows. The record from Carinthia has been collected in alpine rocky slopes with crevice vegetation.

Trend in extent, area or quality?: Stable

Justification for trend: The habitats colonised by *T.poleneci* are as yet not threatened by direct human activities.

##### Habitat

Habitat importance: Major Importance

Habitats: 1.4. Forest - Temperate6. Rocky areas (e.g. inland cliffs, mountain peaks)7.2. Caves and Subterranean Habitats (non-aquatic) - Other Subterranean Habitats

#### Ecology

Size: 2.3 mm

Generation length (yr): 2

Dependency of single sp?: No

Ecology and traits (narrative): This spider shows minor morphological adaptation to subterranean life (Mammola et al. 2022a).

#### Threats

Justification for threats: This species is potentially exposed due to its restricted geographic distribution range. However, the existence of threats is unknown for this species.

##### Threats

Threat type: Past

Threats: 12. Other options - Other threat

#### Conservation

Justification for conservation actions: In Solvenia, *T.poleneci* was considered as potentially threatened due to its rarity and included in the category R of the national Red List (Uradni list RS št. 82/02 in 42/10). This species has been recorded in several sites designated by the Natura 2000 network. In Austria, *T.poleneci* is listed in the 59th Regulation of the Carinthian State Government of 2015 (LGBl. Nr. 59/2015), and fully protected according to the Carinthian Nature Conservation Act 2002 (LGBl. Nr. 79/2002).

##### Conservation actions

Conservation action type: In Place

Conservation actions: 1.1. Land/water protection - Site/area protection1.2. Land/water protection - Resource & habitat protection2.1. Land/water management - Site/area management

#### Other

##### Use and trade

Use type: International

##### Ecosystem services

Ecosystem service type: Important

##### Research needed

Research needed: 1.2. Research - Population size, distribution & trends1.3. Research - Life history & ecology1.5. Research - Threats

Justification for research needed: Research on basic information such as distribution, natural history, ecology and possible threats of the species would be needed.

### Troglohyphantes polyophthalmus

#### Species information

Scientific name: Troglohyphantespolyophthalmus

Species authority: Joseph, 1882

Kingdom: Animalia

Phylum: Arthropoda

Class: Arachnida

Order: Araneae

Family: Linyphiidae

Region for assessment: Global

#### Editor & Reviewers

##### Reviewers

Reviewers: Marc MilnePaulo Borges

##### Editor

Editor: Pedro Cardoso

#### Geographic range

Biogeographic realm: Palearctic

Countries: Slovenia

Map of records (Google Earth): Suppl. material 47

Basis of EOO and AOO: Observed

Basis (narrative): In light of its high level of subterranean specialisation, we assume that the known records of *T.polyophthalmus* are good proxies for defining the AOO and EOO of this species.

Min Elevation/Depth (m): 295

Max Elevation/Depth (m): 1522

Range description: This species has been collected in several hypogean localities in Slovenia (detailed occurrences and relative references in Suppl. material 67).

#### Extent of occurrence

EOO (km2): 2792

Trend: Decline (inferred)

Justification for trend: According to Mammola et al. (2018a), climate change will significantly affect the distribution of subterranean specialised *Troglohyphantes* in the future. Moreover, given the low tolerance to habitat changes of these species as well as their very low dispersal ability, a possible extreme reduction of the geographic range is expected in the future.

Causes ceased?: No

Causes understood?: Yes

Causes reversible?: No

Extreme fluctuations?: No

#### Area of occupancy

Trend: Decline (inferred)

Justification for trend: According to Mammola et al. (2018a), climate change will significantly affect the distribution of subterranean specialised *Troglohyphantes* in the future. Moreover, given the low tolerance to habitat changes of these species as well as their very low dispersal ability, a possible extreme reduction of the geographic range is expected in the future.

Causes ceased?: No

Causes understood?: Yes

Causes reversible?: No

Extreme fluctuations?: No

AOO (km2): 56

#### Locations

Number of locations: 1

Justification for number of locations: Even though this species occurs in several caves, these are interpreted as a single location, as they are all affected by changes in subterranean microclimatic conditions due to climate change.

Trend: Stable

Extreme fluctuations?: No

#### Population

Number of individuals: Unknown

Trend: Decline (inferred)

Justification for trend: In view of the reduced thermal tolerance of subterranean specialised *Troglohyphantes* species (Mammola et al. 2019a), alterations of the microclimatic conditions of the habitat due to climate change are expected to impact the whole population of this species.

Basis for decline: (c) a decline in area of occupancy, extent of occurrence and/or quality of habitat

Causes ceased?: No

Causes understood?: Yes

Causes reversible?: No

Extreme fluctuations?: No

#### Subpopulations

Number of subpopulations: Unknown

Trend: Decline (inferred)

Justification for trend: A decrease in the number of subpopulations is inferred due to the impacts of climate change.

Extreme fluctuations?: No

Severe fragmentation?: No

#### Habitat

System: Terrestrial

Habitat specialist: Yes

Habitat (narrative): This species was mainly collected on the walls and crevices of caves, both at the entrances and in deeper sections of the caves.

Trend in extent, area or quality?: Decline (inferred)

Justification for trend: As seen in Mammola et al. (2018a) for the western alpine species of the genus *Troglohyphantes*, a drastic decline in the habitat suitability of this species as a consequence of climate change is expected.

##### Habitat

Habitat importance: Major Importance

Habitats: 7. Caves and Subterranean Habitats (non-aquatic)

#### Ecology

Size: 3.5 mm

Generation length (yr): 4

Dependency of single sp?: No

Ecology and traits (narrative): This species shows a high degree of adaptation to subterranean habitats, with absence of pigmentation, leg elongation, and eye regression (Mammola et al. 2022a).

#### Threats

Justification for threats: This species is potentially exposed due to its extremely narrow geographic distribution range and its presumably low dispersal capacity. As seen for the species of the genus *Troglohyphantes* of the Western Alps (Mammola et al. 2018a), climate warming is expected to reduce the currently suitable habitat for this spider.

##### Threats

Threat type: Future

Threats: 11.1. Climate change & severe weather - Habitat shifting & alteration11.2. Climate change & severe weather - Droughts11.3. Climate change & severe weather - Temperature extremes

#### Conservation

Justification for conservation actions: This species was considered as potentially threatened due to its rarity, and included in the category R of the Slovenian Red List of endangered plant and animal species (Uradni list RS št. 82/02 in 42/10). Some of the caves where this species has been collected are covered by the Natura 2000 network (SAC SI3000091 Boštonova jama, SAC SI3000093 Ihanska jama, SAC SI3000255 Trnovski gozd - Nanos, SAC SI3000263 Kočevsko, SAC SI3000276 Kras, SPA SI5000023 Kras).

##### Conservation actions

Conservation action type: In Place

Conservation actions: 1.1. Land/water protection - Site/area protection1.2. Land/water protection - Resource & habitat protection2.1. Land/water management - Site/area management

#### Other

##### Use and trade

Use type: International

##### Ecosystem services

Ecosystem service type: Important

##### Research needed

Research needed: 1.2. Research - Population size, distribution & trends1.3. Research - Life history & ecology1.5. Research - Threats

Justification for research needed: Research on basic information such as distribution, ecology, life cycle and possible threats throughout the range would be needed.

### Troglohyphantes regalini

#### Species information

Scientific name: Troglohyphantesregalini

Species authority: Pesarini, 1989

Kingdom: Animalia

Phylum: Arthropoda

Class: Arachnida

Order: Araneae

Family: Linyphiidae

Region for assessment: Global

#### Editor & Reviewers

##### Reviewers

Reviewers: Marc MilnePaulo Borges

##### Editor

Editor: Pedro Cardoso

#### Geographic range

Biogeographic realm: Palearctic

Countries: Italy

Map of records (Google Earth): Suppl. material 48

Basis of EOO and AOO: Observed

Basis (narrative): In light of its high level of subterranean specialisation, we assume that the known records of *T.regalini* are good proxies for defining the AOO and EOO of this species.

Min Elevation/Depth (m): 650

Max Elevation/Depth (m): 1065

Range description: This deep subterranean species is restricted to five caves in the Bergamasque Alps, in northern Italy (detailed occurrences and relative references in Suppl. material 67).

#### Extent of occurrence

EOO (km2): 33

Trend: Decline (inferred)

Justification for trend: According to Mammola et al. (2018a), climate change will significantly affect the distribution of subterranean specialised *Troglohyphantes* in the future. Moreover, given the low tolerance to habitat changes of these species as well as their very low dispersal ability, a possible extreme reduction of the geographic range is expected in the future.

Causes ceased?: No

Causes understood?: Yes

Causes reversible?: No

Extreme fluctuations?: No

#### Area of occupancy

Trend: Decline (inferred)

Justification for trend: According to Mammola et al. (2018a), climate change will significantly affect the distribution of subterranean specialised *Troglohyphantes* in the future. Moreover, given the low tolerance to habitat changes of these species as well as their very low dispersal ability, a possible extreme reduction of the geographic range is expected in the future.

Causes ceased?: No

Causes understood?: Yes

Causes reversible?: No

Extreme fluctuations?: No

AOO (km2): 20

#### Locations

Number of locations: 1

Justification for number of locations: Even though this species occurs in different caves, these are interpreted as a single location, as they are all affected by changes in subterranean microclimatic conditions due to climate change.

Trend: Stable

Extreme fluctuations?: No

#### Population

Number of individuals: Unknown

Trend: Decline (inferred)

Justification for trend: In view of the reduced thermal tolerance of subterranean specialised *Troglohyphantes* species (Mammola et al. 2019a), alterations of the microclimatic conditions of the habitat due to climate change are expected to impact the whole population of this species.

Basis for decline: (c) a decline in area of occupancy, extent of occurrence and/or quality of habitat

Causes ceased?: No

Causes understood?: Yes

Causes reversible?: No

Extreme fluctuations?: No

#### Subpopulations

Number of subpopulations: 5

Trend: Decline (inferred)

Justification for trend: Because of the adaptation to the subterranean habitat and the narrow thermal tolerance of subterranean specialised *Troglohyphantes*, likely hampering dispersal through non-subterranean habitats, each locality can reasonably host a single isolated subpopulation of *T.regalini*. Accordingly, for this species we identified five subpopulations, four of them occurring in the Sebino Bergamasco region, on the western shore of the Iseo Lake (province of Bergamo), and a fifth isolated subpopulation on the eastern shore of the Iseo Lake (province of Brescia). These subpopulations are likely to be impacted by climate change.

Extreme fluctuations?: No

Severe fragmentation?: No

#### Habitat

System: Terrestrial

Habitat specialist: Yes

Habitat (narrative): Specimens have been collected in caves, but there is no additional information about the habitat.

Trend in extent, area or quality?: Decline (inferred)

Justification for trend: As seen in Mammola et al. (2018a) for the western alpine species of *Troglohyphantes*, a drastic decline in the habitat suitability of *T.regalini* as a consequence of climate change is expected.

##### Habitat

Habitat importance: Major Importance

Habitats: 7. Caves and Subterranean Habitats (non-aquatic)

#### Ecology

Size: 2 mm

Generation length (yr): 4

Dependency of single sp?: No

Ecology and traits (narrative): Specimens show a high degree of specialisation to deep subterranean habitats, with pronounced eye regression and absence of pigmentation (Mammola et al. 2022a).

#### Threats

Justification for threats: This species is potentially exposed due to its extremely narrow geographic distribution range and its presumably low dispersal capacity. As seen for the species of *Troglohyphantes* of the Western Alps (Mammola et al. 2018a), climate warming is expected to reduce the currently suitable habitat for this spider.

##### Threats

Threat type: Future

Threats: 11.1. Climate change & severe weather - Habitat shifting & alteration11.2. Climate change & severe weather - Droughts11.3. Climate change & severe weather - Temperature extremes

#### Other

##### Use and trade

Use type: International

##### Ecosystem services

Ecosystem service type: Important

##### Research needed

Research needed: 1.2. Research - Population size, distribution & trends1.3. Research - Life history & ecology1.5. Research - Threats

Justification for research needed: Research on basic information such as distribution, ecology, life cycle and possible threats throughout the range would be needed.

### Troglohyphantes ruffoi

#### Species information

Scientific name: Troglohyphantesruffoi

Species authority: Caporiacco, 1936

Kingdom: Animalia

Phylum: Arthropoda

Class: Arachnida

Order: Araneae

Family: Linyphiidae

Region for assessment: Global

#### Editor & Reviewers

##### Reviewers

Reviewers: Marc MilnePaulo Borges

##### Editor

Editor: Pedro Cardoso

#### Geographic range

Biogeographic realm: Palearctic

Countries: Italy

Map of records (Google Earth): Suppl. material 49

Basis of EOO and AOO: Species Distribution Model

Basis (narrative): Multiple collection sites are available for this non-specialised species. Therefore, it was possible to perform species distribution modelling to predict its potential range with confidence limits. See Methods for details.

Min Elevation/Depth (m): 62

Max Elevation/Depth (m): 1470

Range description: This species had been found in several epigean and hypogean localities in Lessini Mountains, Monte Grappa, Colli Berici and southern Trentino (north-eastern Italy) (detailed occurrences and relative references in Suppl. material 67).

#### Extent of occurrence

EOO (km2): 7378-14872,11658

Trend: Stable

Justification for trend: This species has been collected both in surface and subterranean environments. It is plausible that anthropogenic climate change may affect the habitat suitability of this species. However, in view of the relatively wide thermal tolerance and the relatively high dispersal ability of non-specialised species of *Troglohyphantes* (Mammola et al. 2019a), the distribution range of *T.ruffoi* is not expected to undergo significant reduction in the near future. A deeper study on the impacts of climate change on this species is required.

Causes ceased?: Yes

Causes understood?: Yes

Causes reversible?: Yes

Extreme fluctuations?: No

#### Area of occupancy

Trend: Stable

Justification for trend: This species has been collected both in surface and subterranean environments. It is plausible that anthropogenic climate change may affect the habitat suitability of this species. However, in view of the relatively wide thermal tolerance and the relatively high dispersal ability of non-specialised species of *Troglohyphantes* (Mammola et al. 2019a), the distribution range of *T.ruffoi* is not expected to undergo significant reduction in the near future. A deeper study on the impacts of climate change on this species is required.

Causes ceased?: Yes

Causes understood?: Yes

Causes reversible?: Yes

Extreme fluctuations?: No

AOO (km2): 3760-8492,6560

#### Locations

Number of locations: Not applicable

Justification for number of locations: No known threats to this species.

Trend: Unknown

Extreme fluctuations?: No

#### Population

Number of individuals: Unknown

Trend: Stable

Justification for trend: There are no currently known threats to the species.

Causes ceased?: Yes

Causes understood?: Yes

Causes reversible?: Yes

Extreme fluctuations?: No

#### Subpopulations

Number of subpopulations: Unknown

Trend: Unknown

Extreme fluctuations?: No

Severe fragmentation?: No

#### Habitat

System: Terrestrial

Habitat specialist: No

Habitat (narrative): This species has been collected on cave floors and other subterranean habitats, but it also has been found outside the caves, such as in beech forests and broadleaf woods. No additional information about the habitat was provided.

Trend in extent, area or quality?: Stable

Justification for trend: The habitats colonised by *T.ruffoi* are as yet not threatened by direct human activities.

##### Habitat

Habitat importance: Major Importance

Habitats: 1.4. Forest - Temperate7. Caves and Subterranean Habitats (non-aquatic)

#### Ecology

Size: 2.9 mm

Generation length (yr): 2

Dependency of single sp?: No

Ecology and traits (narrative): Specimens show a very low morphological adaptation to the subterranean life (Mammola et al. 2022a).

#### Threats

Justification for threats: This species is potentially exposed due to its restricted geographic distribution range. However, the existence of threats is unknown for this species.

##### Threats

Threat type: Past

Threats: 12. Other options - Other threat

#### Conservation

Justification for conservation actions: Specimens of *T.ruffoi* have been recorded within the territory of several Natura 2000 sites (EUAP0243 Parco Regionale dei Colli Euganei and SAC/SPA IT3260017 Colli Euganei - Monte Lozzo - Monte Ricco, SAC/SPA IT3210040 Monti Lessini - Pasubio - Piccole Dolomiti Vicentine, SAC/SPA IT3230022 Massiccio del Grappa, SAC IT3220037 Colli Berici, SAC IT3120127 Monti Tremalzo e Tombea, SAC IT3120173 Monte Baldo di Brentonico).

##### Conservation actions

Conservation action type: In Place

Conservation actions: 1.1. Land/water protection - Site/area protection1.2. Land/water protection - Resource & habitat protection2.1. Land/water management - Site/area management

#### Other

##### Use and trade

Use type: International

##### Ecosystem services

Ecosystem service type: Important

##### Research needed

Research needed: 1.2. Research - Population size, distribution & trends1.3. Research - Life history & ecology1.5. Research - Threats

Justification for research needed: Research on basic information such as distribution, ecology, life cycle and possible threats throughout the range would be needed.

### Troglohyphantes sbordonii

#### Species information

Scientific name: Troglohyphantessbordonii

Species authority: Brignoli, 1975

Kingdom: Animalia

Phylum: Arthropoda

Class: Arachnida

Order: Araneae

Family: Linyphiidae

Region for assessment: Global

#### Editor & Reviewers

##### Reviewers

Reviewers: Marc MilnePaulo Borges

##### Editor

Editor: Pedro Cardoso

#### Geographic range

Biogeographic realm: Palearctic

Countries: SloveniaAustriaItaly

Map of records (Google Earth): Suppl. material 50

Basis of EOO and AOO: Species Distribution Model

Basis (narrative): Multiple collection sites are available for this species. Therefore, it was possible to perform species distribution modelling to predict its potential range with confidence limits. See Methods for details.

Min Elevation/Depth (m): 250

Max Elevation/Depth (m): 2120

Range description: This species is predicted to be present in the Venetian Prealps, Julian Alps and Prealps, and Carnic Prealps (detailed occurrences and relative references in Suppl. material 67).

#### Extent of occurrence

EOO (km2): 5441-9686,6731

Trend: Stable

Justification for trend: This species has been collected both in hypogean and epigean environments. It is plausible that climate change may affect the habitat suitability of this species. However, in view of the relatively wide thermal tolerance and the relatively high dispersal ability of non-specialised species of *Troglohyphantes* (Mammola et al. 2019a), the distribution range of *T.sbordonii* is not expected to undergo significant reduction in the near future. A deeper study on the impacts of climate change on this species is required.

Causes ceased?: Yes

Causes understood?: Yes

Causes reversible?: Yes

Extreme fluctuations?: No

#### Area of occupancy

Trend: Stable

Justification for trend: This species has been collected both in hypogean and epigean environments. It is plausible that climate change may affect the habitat suitability of this species. However, in view of the relatively wide thermal tolerance and the relatively high dispersal ability of non-specialised species of *Troglohyphantes* (Mammola et al. 2019a), the distribution range of *T.sbordonii* is not expected to undergo significant reduction in the near future. A deeper study on the impacts of climate change on this species is required.

Causes ceased?: Yes

Causes understood?: Yes

Causes reversible?: Yes

Extreme fluctuations?: No

AOO (km2): 3644-5880,4624

#### Locations

Number of locations: Not applicable

Justification for number of locations: No known threats to this species.

Trend: Stable

Extreme fluctuations?: No

#### Population

Number of individuals: Unknown

Trend: Stable

Justification for trend: There are no currently known threats to the species.

Causes ceased?: Yes

Causes understood?: Yes

Causes reversible?: Yes

Extreme fluctuations?: No

#### Subpopulations

Number of subpopulations: Unknown

Trend: Unknown

Extreme fluctuations?: No

Severe fragmentation?: No

#### Habitat

System: Terrestrial

Habitat specialist: No

Habitat (narrative): In north-eastern Italy and in north-western Slovenia, this species has been mainly found in caves. Conversely, in southern Austria the known localities occur in mixed beech and spruce forests of the montane and subalpine zone, under stones in the soil.

Trend in extent, area or quality?: Stable

Justification for trend: The habitats colonised by *T.sbordonii* are as yet not threatened by direct human activities.

##### Habitat

Habitat importance: Major Importance

Habitats: 1.4. Forest - Temperate7. Caves and Subterranean Habitats (non-aquatic)

#### Ecology

Size: 2.8 mm

Generation length (yr): 2

Dependency of single sp?: No

Ecology and traits (narrative): This species shows minor morphological adaptation to subterranean life (Mammola et al. 2022a). In the artificial tunnel Kluža, near Bovec (Upper Carniola, Slovenia), this species has been found to coexist in the same habitat with *T.gamsi* (Deeleman-Reinhold 1978).

#### Threats

Justification for threats: This species is potentially exposed due to its restricted geographic distribution range. However, the existence of threats is unknown for this species.

##### Threats

Threat type: Past

Threats: 12. Other options - Other threat

#### Conservation

Justification for conservation actions: Part of the potential range of this species is inside protected areas and sites of the Natura 2000 network. In Austria, *T.sbordonii* is listed in the category R of the Red List of endangered spiders for Carinthia (Komposch and Steinberger 1999). In addition, this species figures in the 59th Regulation of the Carinthian State Government of 2015 (LGBl. Nr. 59/2015), which amends the Carinthian Nature Conservation Act 2002 (LGBl. Nr. 79/2002).

##### Conservation actions

Conservation action type: In Place

Conservation actions: 1.1. Land/water protection - Site/area protection1.2. Land/water protection - Resource & habitat protection2.1. Land/water management - Site/area management

#### Other

##### Use and trade

Use type: International

##### Ecosystem services

Ecosystem service type: Important

##### Research needed

Research needed: 1.2. Research - Population size, distribution & trends1.3. Research - Life history & ecology1.5. Research - Threats

Justification for research needed: Research on basic information such as distribution, ecology, life cycle and possible threats throughout the range would be needed.

### Troglohyphantes sciakyi

#### Species information

Scientific name: Troglohyphantessciakyi

Species authority: Pesarini, 1989

Kingdom: Animalia

Phylum: Arthropoda

Class: Arachnida

Order: Araneae

Family: Linyphiidae

Region for assessment: Global

#### Editor & Reviewers

##### Reviewers

Reviewers: Marc MilnePaulo Borges

##### Editor

Editor: Pedro Cardoso

#### Geographic range

Biogeographic realm: Palearctic

Countries: Italy

Map of records (Google Earth): Suppl. material 51

Basis of EOO and AOO: Species Distribution Model

Basis (narrative): Multiple collection sites are known for this species. Therefore, it was possible to perform species distribution modelling to predict its potential range with confidence limits. See Methods for details.

Min Elevation/Depth (m): 1250

Max Elevation/Depth (m): 2636

Range description: The range of *T.sciakyi* encompasses the Bergamasque Alps and Prealps, and the Southern Rhaetian Alps, in Lombardia and Trentino Alto-Adige, northern Italy (detailed occurrences and relative references in Suppl. material 67).

#### Extent of occurrence

EOO (km2): 5949-8984,7715

Trend: Stable

Justification for trend: This species has been mainly collected in Alpine epigean habitats above 1,700 m, which are particularly vulnerable to climatic variations due to climate change. However, considering the low level of specialisation and the high altimetric range of its distribution, we assume a lower risk compared to subterranean specialised species.

Causes ceased?: Yes

Causes understood?: Yes

Causes reversible?: Yes

Extreme fluctuations?: No

#### Area of occupancy

Trend: Stable

Justification for trend: This species has been mainly collected in Alpine epigean habitats above 1,700 m, which are particularly vulnerable to climatic variations due to climate change. However, considering the low level of specialisation and the high altimetric range of its distribution, we assume a lower risk compared to subterranean specialised species.

Causes ceased?: Yes

Causes understood?: Yes

Causes reversible?: Yes

Extreme fluctuations?: No

AOO (km2): 2824-5420,4160

#### Locations

Number of locations: Not applicable

Justification for number of locations: No known major threats to this species.

Trend: Stable

Extreme fluctuations?: No

#### Population

Number of individuals: Unknown

Trend: Stable

Justification for trend: There are no currently known threats to this species.

Causes ceased?: Yes

Causes understood?: Yes

Causes reversible?: Yes

Extreme fluctuations?: No

#### Subpopulations

Number of subpopulations: Unknown

Trend: Unknown

Justification for trend: There is no information on the number of the subpopulations of this species.

Extreme fluctuations?: No

Severe fragmentation?: No

#### Habitat

System: Terrestrial

Habitat specialist: No

Habitat (narrative): This species has been found predominantly in rocky habitats and alpine screes at high altitudes. Some specimens have been collected in hypogean habitat.

Trend in extent, area or quality?: Stable

##### Habitat

Habitat importance: Major Importance

Habitats: 6. Rocky areas (e.g. inland cliffs, mountain peaks)7. Caves and Subterranean Habitats (non-aquatic)

#### Ecology

Size: 3 mm

Generation length (yr): 2

Dependency of single sp?: No

Ecology and traits (narrative): Not much is known about the ecology of this orophile species. This spider shows minor specialisation to subterranean life (Mammola et al. 2022a).

#### Threats

Justification for threats: Some of the habitats where this species has been found are vulnerable to climatic variations due to climate change. However, considering the relatively high dispersal ability of this species, *T.sciakyi* is not expected to experience any decline.

##### Threats

Threat type: Past

Threats: 12. Other options - Other threat

#### Conservation

Justification for conservation actions: Most of the predicted distribution range of this species falls within several protected areas (EUAP0017 and SPA IT2040044 Parco Nazionale dello Stelvio, SPA IT3120157 Stelvio, SAC/SPA IT3110038 Ultimo - Solda nel Parco Nazionale dello Stelvio, EUAP0930 and SAC IT3120177 Dolomiti di Brenta, SPA IT3120159 Brenta, SAC IT2060009 Val Nossana - Cima di Grem, SAC IT2060005 Val Sedornia - Val Zurio - Pizzo della Presolana, SAC IT2060004 Alta Val di Scalve, SPA IT2060401 Parco Regionale Orobie Bergamasche, SAC IT3120001 Alta Val di Rabbi).

##### Conservation actions

Conservation action type: In Place

Conservation actions: 1.1. Land/water protection - Site/area protection1.2. Land/water protection - Resource & habitat protection

#### Other

##### Use and trade

Use type: International

##### Ecosystem services

Ecosystem service type: Important

##### Research needed

Research needed: 1.2. Research - Population size, distribution & trends1.3. Research - Life history & ecology1.5. Research - Threats

Justification for research needed: Research on basic information such as natural history, ecology and possible threats of the species would be needed.

### Troglohyphantes scientificus

#### Species information

Scientific name: Troglohyphantesscientificus

Species authority: Deeleman-Reinhold, 1978

Kingdom: Animalia

Phylum: Arthropoda

Class: Arachnida

Order: Araneae

Family: Linyphiidae

Region for assessment: Global

#### Editor & Reviewers

##### Reviewers

Reviewers: Marc MilnePaulo Borges

##### Editor

Editor: Pedro Cardoso

#### Geographic range

Biogeographic realm: Palearctic

Countries: SloveniaItaly

Map of records (Google Earth): Suppl. material 52

Basis of EOO and AOO: Observed

Basis (narrative): In light of its high level of subterranean specialisation, we assume that the known records of *T.scientificus* are good proxies for defining the EOO and AOO of this species.

Min Elevation/Depth (m): 200

Max Elevation/Depth (m): 909

Range description: This species has been found in a few caves in Friuli Venezia Giulia (north-eastern Italy) and in Upper Carniola (western Slovenia) (detailed occurrences and relative references in Suppl. material 67).

#### Extent of occurrence

EOO (km2): 286

Trend: Decline (inferred)

Justification for trend: According to Mammola et al. (2018a), climate change will significantly affect the distribution of subterranean specialised *Troglohyphantes* in the future. Moreover, given the low tolerance to habitat changes of these species as well as their very low dispersal ability, a possible extreme reduction of the geographic range is expected in the future.

Causes ceased?: No

Causes understood?: Yes

Causes reversible?: No

Extreme fluctuations?: No

#### Area of occupancy

Trend: Decline (inferred)

Justification for trend: According to Mammola et al. (2018a), climate change will significantly affect the distribution of subterranean specialised *Troglohyphantes* in the future. Moreover, given the low tolerance to habitat changes of these species as well as their very low dispersal ability, a possible extreme reduction of the geographic range is expected in the future.

Causes ceased?: No

Causes understood?: Yes

Causes reversible?: No

Extreme fluctuations?: No

AOO (km2): 44

#### Locations

Number of locations: 1

Justification for number of locations: Even though this species occurs in different caves, these are interpreted as a single location, as they are all affected by changes in subterranean microclimatic conditions due to climate change.

Trend: Stable

Extreme fluctuations?: No

#### Population

Number of individuals: Unknown

Trend: Decline (inferred)

Justification for trend: In view of the reduced thermal tolerance of subterranean specialised *Troglohyphantes* species (Mammola et al. 2019a), alterations of the microclimatic conditions of the habitat due to climate change are expected to impact the whole population of this species.

Basis for decline: (c) a decline in area of occupancy, extent of occurrence and/or quality of habitat

Causes ceased?: No

Causes understood?: Yes

Causes reversible?: No

Extreme fluctuations?: No

#### Subpopulations

Number of subpopulations: 11

Trend: Decline (inferred)

Justification for trend: Because of the adaptation to the subterranean habitat and the narrow thermal tolerance of highly adapted *Troglohyphantes*, likely hampering dispersal through non-subterranean habitats, each subpopulation of *T.scientificus* can reasonably occur in a single isolated or few contiguous subpopulations. Accordingly, for this species we identified 11 subpopulations, most of them in the province of Udine, in Friuli-Venezia Giulia (Italy), and a further isolated subpopulation occurring in Upper Carniola (Slovenia). These subpopulations are likely to be impacted by climate change.

Extreme fluctuations?: No

Severe fragmentation?: No

#### Habitat

System: Terrestrial

Habitat specialist: Yes

Habitat (narrative): Not much is known on the habitat of this species. Specimens were collected in hypogean environments.

Trend in extent, area or quality?: Decline (inferred)

Justification for trend: As seen in Mammola et al. (2018a) for the species of the genus *Troglohyphantes* of the Western Alps, a drastic decline in the habitat suitability of this species as a consequence of climate change is expected.

##### Habitat

Habitat importance: Major Importance

Habitats: 7. Caves and Subterranean Habitats (non-aquatic)

#### Ecology

Size: 3.8 mm

Generation length (yr): 4

Dependency of single sp?: No

Ecology and traits (narrative): *Troglohyphantesscientificus* shows pronounced eyes regression and absence of pigmentation (Mammola et al. 2022a).

#### Threats

Justification for threats: This species is potentially exposed due to its extremely narrow geographic distribution range and its presumably low dispersal capacity. As seen for the species of the genus *Troglohyphantes* of the Western Alps (Mammola et al. 2018a), climate warming is expected to reduce the currently suitable habitat for this spider.

##### Threats

Threat type: Future

Threats: 11.1. Climate change & severe weather - Habitat shifting & alteration11.2. Climate change & severe weather - Droughts11.3. Climate change & severe weather - Temperature extremes

#### Conservation

Justification for conservation actions: Due to its rarity in Slovenia, *T.scientificus* was considered as potentially threatened and included in the category R of the national Red List (Uradni list RS št. 82/02 in 42/10). The Slovenian localities of this species fall within the Triglav National Park and within the Special Area of Conservation and Special Protection Area of the Julian Alps (SAC SI3000253, SPA SI5000019).

##### Conservation actions

Conservation action type: In Place

Conservation actions: 1.1. Land/water protection - Site/area protection1.2. Land/water protection - Resource & habitat protection2.1. Land/water management - Site/area management

#### Other

##### Use and trade

Use type: International

##### Ecosystem services

Ecosystem service type: Important

##### Research needed

Research needed: 1.2. Research - Population size, distribution & trends1.3. Research - Life history & ecology1.5. Research - Threats

Justification for research needed: Research on basic information such as distribution, natural history, ecology and possible threats of the species would be needed.

### Troglohyphantes similis

#### Species information

Scientific name: Troglohyphantessimilis

Species authority: Fage, 1919

Kingdom: Animalia

Phylum: Arthropoda

Class: Arachnida

Order: Araneae

Family: Linyphiidae

Region for assessment: Global

#### Editor & Reviewers

##### Reviewers

Reviewers: Marc MilnePaulo Borges

##### Editor

Editor: Pedro Cardoso

#### Geographic range

Biogeographic realm: Palearctic

Countries: Slovenia

Map of records (Google Earth): Suppl. material 53

Basis of EOO and AOO: Observed

Basis (narrative): In light of the high subterranean specialisation of this species, it is not expected that new findings could significantly expand its known geographic range.

Min Elevation/Depth (m): 526

Max Elevation/Depth (m): 638

Range description: This species is known only from the type locality, Lukova jama pri Zdihovem, a cave in Lower Carniola, southern Slovenia, near the border with Croatia (see Suppl. material 67). The records of this species in Italy are considered as doubtful (Pantini and Isaia 2019), likely referring to *T.fagei* or *T.sbordonii* (Gasparo 2000).

#### Extent of occurrence

EOO (km2): 4

Trend: Decline (inferred)

Justification for trend: According to Mammola et al. (2018a), climate change will significantly affect the distribution of subterranean specialised *Troglohyphantes* in the future. Moreover, given the low tolerance to habitat changes of these species as well as their very low dispersal ability, a possible extreme reduction of the geographic range is expected in the future.

Causes ceased?: No

Causes understood?: Yes

Causes reversible?: No

Extreme fluctuations?: No

#### Area of occupancy

Trend: Decline (inferred)

Justification for trend: According to Mammola et al. (2018a), climate change will significantly affect the distribution of subterranean specialised *Troglohyphantes* in the future. Moreover, given the low tolerance to habitat changes of these species as well as their very low dispersal ability, a possible extreme reduction of the geographic range is expected in the future.

Causes ceased?: No

Causes understood?: Yes

Causes reversible?: No

Extreme fluctuations?: No

AOO (km2): 4

#### Locations

Number of locations: 1

Justification for number of locations: The habitat where this species occurs is affected by changes in subterranean microclimatic conditions due to climate change, which is expected to impact the whole population.

Trend: Stable

Extreme fluctuations?: No

#### Population

Number of individuals: Unknown

Trend: Decline (inferred)

Justification for trend: In view of the reduced thermal tolerance of subterranean specialised *Troglohyphantes* (Mammola et al. 2019a), alterations of the microclimatic conditions of the habitat due to climate change are expected to impact the whole population of this species.

Basis for decline: (c) a decline in area of occupancy, extent of occurrence and/or quality of habitat

Causes ceased?: No

Causes understood?: Yes

Causes reversible?: No

Extreme fluctuations?: No

#### Subpopulations

Number of subpopulations: 1

Trend: Stable

Justification for trend: For this species we identified a single subpopulation.

Extreme fluctuations?: No

Severe fragmentation?: No

#### Habitat

System: Terrestrial

Habitat specialist: Yes

Habitat (narrative): Specimens have been collected in caves, but there is no specific information available about the habitat.

Trend in extent, area or quality?: Decline (inferred)

Justification for trend: As seen in Mammola et al. (2018a) for the species of *Troglohyphantes* of the Western Alps, a drastic decline in the habitat suitability of *T.similis* as a consequence of climate change is expected.

##### Habitat

Habitat importance: Major Importance

Habitats: 7. Caves and Subterranean Habitats (non-aquatic)

#### Ecology

Size: 2.5 mm

Generation length (yr): 4

Dependency of single sp?: No

Ecology and traits (narrative): Not much is known about the ecology and life history of this species. Specimens are weakly pigmented (Mammola et al. 2022a).

#### Threats

Justification for threats: This species is potentially exposed due to its extremely narrow geographic distribution range and its presumably low dispersal capacity. As seen for the *Troglohyphantes* species of the Western Alps (Mammola et al. 2018a), climate warming is expected to reduce the currently suitable habitat for this spider.

##### Threats

Threat type: Future

Threats: 11.1. Climate change & severe weather - Habitat shifting & alteration11.2. Climate change & severe weather - Droughts11.3. Climate change & severe weather - Temperature extremes

#### Conservation

Justification for conservation actions: *Troglohyphantessimilis* was listed in the first IUCN Red List (IUCN Conservation Monitoring Centre 1986) due to its restricted geographical distribution, and was assessed as Vulnerable in the 1996 IUCN Red List (World Conservation Monitoring Centre 1996b), but its status has not been updated since. In addition, this species figures in the Slovenian national Red List due to its rarity (category R) (Uradni list RS št. 82/02 in 42/10). The known locality of this species occurs within the protected area of Kočevsko (SAC SI3000263 and SPA SI5000013).

##### Conservation actions

Conservation action type: In Place

Conservation actions: 1.1. Land/water protection - Site/area protection1.2. Land/water protection - Resource & habitat protection2.1. Land/water management - Site/area management

#### Other

##### Use and trade

Use type: International

##### Ecosystem services

Ecosystem service type: Important

##### Research needed

Research needed: 1.1. Research - Taxonomy1.2. Research - Population size, distribution & trends1.3. Research - Life history & ecology1.5. Research - Threats

Justification for research needed: The taxonomical status of the species and possible synonymy with *T.spinipes* is going to be clarified (see Pavlek et al. *in litteris*). Research on basic information such as distribution, natural history, ecology and possible threats of the species would be needed.

### Troglohyphantes sketi

#### Species information

Scientific name: Troglohyphantessketi

Species authority: Deeleman-Reinhold, 1978

Kingdom: Animalia

Phylum: Arthropoda

Class: Arachnida

Order: Araneae

Family: Linyphiidae

Region for assessment: Global

#### Editor & Reviewers

##### Reviewers

Reviewers: Marc MilnePaulo Borges

##### Editor

Editor: Pedro Cardoso

#### Geographic range

Biogeographic realm: Palearctic

Countries: CroatiaSlovenia

Map of records (Google Earth): Suppl. material 54

Basis of EOO and AOO: Observed

Basis (narrative): This species was collected only in three localities, scattered across a relatively wide geographic range. Its low level of subterranean specialisation possibly reflects a higher dispersal capacity when compared to subterranean specialised species. Given this situation, any modelling of the current habitat suitability is unreliable, and the known distribution range should be taken with caution. Further research is needed on this species in order to assess its extinction risk.

Min Elevation/Depth (m): 575

Max Elevation/Depth (m): 648

Range description: There are only three records for this species: two in Slovenia, Mežnarjevo brezno near Lož in Inner Carniola and Svinjska jama near Dolenji in Slovenian Littoral, and one in Croatia, at jama Bedara on Žumberak hill (detailed occurrences and relative references in Suppl. material 67).

#### Extent of occurrence

EOO (km2): 300

Trend: Unknown

Causes ceased?: Unknown

Causes understood?: Unknown

Causes reversible?: Unknown

Extreme fluctuations?: No

#### Area of occupancy

Trend: Unknown

Causes ceased?: Unknown

Causes understood?: Unknown

Causes reversible?: Unknown

Extreme fluctuations?: No

AOO (km2): 12

#### Locations

Number of locations: Unknown

Justification for number of locations: The data available are not enough to estimate the number of locations for this species.

Trend: Unknown

Extreme fluctuations?: No

#### Population

Number of individuals: Unknown

Trend: Unknown

Justification for trend: The population size and trend are unknown.

Causes ceased?: Unknown

Causes understood?: Unknown

Causes reversible?: Unknown

Extreme fluctuations?: No

#### Subpopulations

Number of subpopulations: Unknown

Trend: Unknown

Extreme fluctuations?: No

Severe fragmentation?: No

#### Habitat

System: Terrestrial

Habitat specialist: Yes

Habitat (narrative): Specimens were all found in caves. In the type locality, Mežnarjevo brezno, a vertical cave of about 15 m depth, specimens have been collected on the bottom of a chamber between boulders and pieces of decaying wood, in almost absolute darkness.

Trend in extent, area or quality?: Unknown

##### Habitat

Habitat importance: Major Importance

Habitats: 7. Caves and Subterranean Habitats (non-aquatic)

#### Ecology

Size: 2.8 mm

Generation length (yr): 2

Dependency of single sp?: No

Ecology and traits (narrative): Specimens show a minor specialisation to subterranean life, with a slight eye reduction (Mammola et al. 2022a).

#### Threats

Justification for threats: Unknown threats.

##### Threats

Threat type: Past

Threats: 12. Other options - Other threat

#### Conservation

Justification for conservation actions: In Slovenia, *T.sketi* was considered as potentially threatened due to its rarity and included in the category R of the national Red List (Uradni list RS št. 82/02 in 42/10). One of the two caves where this species has been found in Slovenia, is included in the Natura 2000 network (SAC SI3000232 Notranjski trikotnik, SPA SI5000002 Snežnik - Pivka). The Croatian record of this species falls within the Natural Park of Žumberak and Samobor Mountains (SAC HR2000586), and the cave is not open to public.

##### Conservation actions

Conservation action type: In Place

Conservation actions: 1.1. Land/water protection - Site/area protection1.2. Land/water protection - Resource & habitat protection2.1. Land/water management - Site/area management

#### Other

##### Use and trade

Use type: International

##### Ecosystem services

Ecosystem service type: Important

##### Research needed

Research needed: 1.2. Research - Population size, distribution & trends1.3. Research - Life history & ecology1.5. Research - Threats

Justification for research needed: Research on basic information such as distribution, natural history, ecology and possible threats of the species would be needed.

### Troglohyphantes sordellii

#### Species information

Scientific name: Troglohyphantessordellii

Species authority: (Pavesi, 1875)

Kingdom: Animalia

Phylum: Arthropoda

Class: Arachnida

Order: Araneae

Family: Linyphiidae

Region for assessment: Global

#### Editor & Reviewers

##### Reviewers

Reviewers: Marc MilnePaulo Borges

##### Editor

Editor: Pedro Cardoso

#### Geographic range

Biogeographic realm: Palearctic

Countries: SwitzerlandItaly

Map of records (Google Earth): Suppl. material 55

Basis of EOO and AOO: Observed

Basis (narrative): Despite the relatively high number of records of this non-specialised species, the species distribution predicted by the models was found to be unreliable by our own expert opinion. In view of this, only the observed distribution range is presented.

Min Elevation/Depth (m): 340

Max Elevation/Depth (m): 1990

Range description: The currently known distribution of *T.sordellii* is centred in the Bergamasque Alps and Prealps, Brescia Prealps and Lugano Prealps, in northern Italy and southern Switzerland (detailed occurrences and relative references in Suppl. material 67).

#### Extent of occurrence

EOO (km2): 3249

Trend: Stable

Justification for trend: This species has been collected both in epigean and hypogean environments. It is plausible that anthropogenic climate change may affect the habitat suitability of this species. However, in view of the relatively wide thermal tolerance and the relatively high dispersal ability of non-specialised species of *Troglohyphantes* (Mammola et al. 2019a), the distribution range of *T.sordellii* is not expected to undergo significant reduction in the near future. A deeper study on the current distribution of this species and on the potential impacts of climate change is required.

Causes ceased?: Yes

Causes understood?: Yes

Causes reversible?: Yes

Extreme fluctuations?: No

#### Area of occupancy

Trend: Stable

Justification for trend: This species has been collected both in epigean and hypogean environments. It is plausible that anthropogenic climate change may affect the habitat suitability of this species. However, in view of the relatively wide thermal tolerance and the relatively high dispersal ability of non-specialised species of *Troglohyphantes* (Mammola et al. 2019a), the distribution range of *T.sordellii* is not expected to undergo significant reduction in the near future. A deeper study on the current distribution of this species and on the potential impacts of climate change is required.

Causes ceased?: Yes

Causes understood?: Yes

Causes reversible?: Yes

Extreme fluctuations?: No

AOO (km2): 84

#### Locations

Number of locations: Not applicable

Justification for number of locations: No known threats to this species.

Trend: Stable

Extreme fluctuations?: No

#### Population

Number of individuals: Unknown

Trend: Stable

Justification for trend: There are no currently known threats to the species.

Causes ceased?: Yes

Causes understood?: Yes

Causes reversible?: Yes

Extreme fluctuations?: No

#### Subpopulations

Number of subpopulations: Unknown

Trend: Unknown

Extreme fluctuations?: No

Severe fragmentation?: No

#### Habitat

System: Terrestrial

Habitat specialist: No

Habitat (narrative): At lower altitudes this species is often found in caves, while at higher altitudes it is generally found in cool and shaded rocky habitats.

Trend in extent, area or quality?: Stable

Justification for trend: The habitats colonised by *T.sordellii* are as yet not threatened by direct human activities.

##### Habitat

Habitat importance: Major Importance

Habitats: 6. Rocky areas (e.g. inland cliffs, mountain peaks)7. Caves and Subterranean Habitats (non-aquatic)

#### Ecology

Size: 3.3 mm

Generation length (yr): 2

Dependency of single sp?: No

Ecology and traits (narrative): This spider shows low morphological adaptation to subterranean life (Mammola et al. 2022a).

#### Threats

Justification for threats: This species is potentially exposed due to its restricted distribution range. However, the existence of threats is unknown for this species.

##### Threats

Threat type: Past

Threats: 12. Other options - Other threat

#### Conservation

Justification for conservation actions: The distribution of *T.sordellii* falls within several protected areas (SAC IT2010002 Monte Legnone e Chiusarella, SAC IT2010005 Monte Martica, SAC IT2060001 Valtorta e Valmoresca, SAC IT2060009 Val Nossana - Cima di Grem, SPA IT2010401 Parco Regionale Campo dei Fiori, SPA IT2070402 Alto Garda Bresciano, SPA IT2060401 Parco Regionale Orobie Bergamasche).

##### Conservation actions

Conservation action type: In Place

Conservation actions: 1.1. Land/water protection - Site/area protection1.2. Land/water protection - Resource & habitat protection2.1. Land/water management - Site/area management

#### Other

##### Use and trade

Use type: International

##### Ecosystem services

Ecosystem service type: Important

##### Research needed

Research needed: 1.2. Research - Population size, distribution & trends1.3. Research - Life history & ecology1.5. Research - Threats

Justification for research needed: Research on basic information such as natural history, ecology and possible threats of the species would be needed.

### Troglohyphantes spatulifer

#### Species information

Scientific name: Troglohyphantesspatulifer

Species authority: Pesarini, 2001

Kingdom: Animalia

Phylum: Arthropoda

Class: Arachnida

Order: Araneae

Family: Linyphiidae

Region for assessment: Global

#### Editor & Reviewers

##### Reviewers

Reviewers: Marc MilnePaulo Borges

##### Editor

Editor: Pedro Cardoso

#### Geographic range

Biogeographic realm: Palearctic

Countries: Italy

Map of records (Google Earth): Suppl. material 56

Basis of EOO and AOO: Observed

Basis (narrative): This species was collected in two localities within an isolated forest patch surrounded by intensively-managed agricultural lands. Its low level of subterranean specialisation possibly reflects a relatively high dispersal capacity. However, the intensive agriculture activities dominating the landscape in this area hampers the possible dispersal of this species.

Min Elevation/Depth (m): 335

Max Elevation/Depth (m): 335

Range description: This species was collected only in the cave Laca di Montorfen and in the nearby forests of Monte Orfano, in the province of Brescia (Lombardia, northern Italy) (see Suppl. material 67). It was never recorded after the original description (Pantini and Isaia 2019).

#### Extent of occurrence

EOO (km2): 8

Trend: Decline (inferred)

Justification for trend: This species is restricted to an isolated forest patch embedded in an intensively-managed agricultural area. We assume that high input agricultural activities characterising the area represent a potential threat for the survival of this species. Moreover, the intensive agriculture activities dominating the landscape in this area hampers the possible dispersal of this species towards suitable habitats.

Causes ceased?: No

Causes understood?: Yes

Causes reversible?: No

Extreme fluctuations?: No

#### Area of occupancy

Trend: Decline (inferred)

Justification for trend: This species is restricted to an isolated forest patch embedded in an intensively-managed agricultural area. We assume that high input agricultural activities characterising the area represent a potential threat for the survival of this species. Moreover, the intensive agriculture activities dominating the landscape in this area hampers the possible dispersal of this species towards suitable habitats.

Causes ceased?: No

Causes understood?: Yes

Causes reversible?: No

Extreme fluctuations?: No

AOO (km2): 8

#### Locations

Number of locations: 1

Justification for number of locations: The forested patch of Monte Orfano where this species has been found, is threatened by the intensive agricultural activities of the surrounding human dominated landscape.

Trend: Stable

Extreme fluctuations?: No

#### Population

Number of individuals: Unknown

Trend: Decline (inferred)

Justification for trend: A decline in population size is inferred from potential decline in EOO, AOO and habitat quality due to the high input agricultural activities.

Basis for decline: (c) a decline in area of occupancy, extent of occurrence and/or quality of habitat

Causes ceased?: No

Causes understood?: Yes

Causes reversible?: No

Extreme fluctuations?: No

#### Subpopulations

Number of subpopulations: 1

Trend: Stable

Justification for trend: Examining the known distribution range of this species and considering the intensively-managed agricultural matrix surrounding the forested patch where the species has been found, it is possible to identify a single subpopulation occurring in Monte Orfano.

Extreme fluctuations?: No

Severe fragmentation?: No

#### Habitat

System: Terrestrial

Habitat specialist: No

Habitat (narrative): This species has been collected in a cave and in the nearby forest. No additional information about the habitat has been provided.

Trend in extent, area or quality?: Decline (inferred)

Justification for trend: The habitat of this species is inferred to be declining in area, extent and quality due to the intensive agriculture activities dominating the landscape in this area.

##### Habitat

Habitat importance: Major Importance

Habitats: 1.4. Forest - Temperate7. Caves and Subterranean Habitats (non-aquatic)

#### Ecology

Size: 3.0 mm

Generation length (yr): 2

Dependency of single sp?: No

Ecology and traits (narrative): This spider shows minor morphological specialisation to subterranean life (Mammola et al. 2022a).

#### Threats

Justification for threats: *Troglohyphantesspatulifer* is potentially exposed due to its extremely narrow geographic distribution range. Moreover, the range of the species is restricted to Monte Orfano, a small isolated forested hill embedded within the vineyard region of Franciacorta, a human dominated landscape characterised by an intensive agricultural matrix. This intensively managed landscape may influence the dispersal of this species.

##### Threats

Threat type: Ongoing

Threats: 2.1. Agriculture & aquaculture - Annual & perennial non-timber crops9.3. Pollution - Agricultural & forestry effluents

#### Other

##### Use and trade

Use type: International

##### Ecosystem services

Ecosystem service type: Important

##### Research needed

Research needed: 1.2. Research - Population size, distribution & trends1.3. Research - Life history & ecology1.5. Research - Threats

Justification for research needed: Research on basic information such as distribution, ecology, life cycle and possible threats throughout the range would be needed.

### Troglohyphantes spinipes

#### Species information

Scientific name: Troglohyphantesspinipes

Species authority: Fage, 1919

Kingdom: Animalia

Phylum: Arthropoda

Class: Arachnida

Order: Araneae

Family: Linyphiidae

Region for assessment: Global

#### Editor & Reviewers

##### Reviewers

Reviewers: Marc MilnePaulo Borges

##### Editor

Editor: Pedro Cardoso

#### Geographic range

Biogeographic realm: Palearctic

Countries: Slovenia

Map of records (Google Earth): Suppl. material 57

Basis of EOO and AOO: Observed

Basis (narrative): In light of its high level of subterranean specialisation, we assume that the known records of *T.spinipes* are good proxies for defining the AOO and EOO of this species.

Min Elevation/Depth (m): 530

Max Elevation/Depth (m): 953

Range description: *Troglohyphantesspinipes* is known from a few caves in the Kočevje mountains (Lower Carniola, southern Slovenia) (detailed occurrences and relative references in Suppl. material 67).

#### Extent of occurrence

EOO (km2): 8

Trend: Decline (inferred)

Justification for trend: According to Mammola et al. (2018a), climate change will significantly affect the distribution of subterranean specialised *Troglohyphantes* in the future. Moreover, given the low tolerance to habitat changes of these species as well as their very low dispersal ability, a possible extreme reduction of the geographic range is expected in the future.

Causes ceased?: Yes

Causes understood?: Yes

Causes reversible?: No

Extreme fluctuations?: No

#### Area of occupancy

Trend: Decline (inferred)

Justification for trend: According to Mammola et al. (2018a), climate change will significantly affect the distribution of subterranean specialised *Troglohyphantes* in the future. Moreover, given the low tolerance to habitat changes of these species as well as their very low dispersal ability, a possible extreme reduction of the geographic range is expected in the future.

Causes ceased?: No

Causes understood?: Yes

Causes reversible?: No

Extreme fluctuations?: No

AOO (km2): 8

#### Locations

Number of locations: 1

Justification for number of locations: Even though this species occurs in different caves, these are interpreted as a single location, as they are all affected by changes in subterranean microclimatic conditions due to climate change.

Trend: Stable

Extreme fluctuations?: No

#### Population

Number of individuals: Unknown

Trend: Decline (inferred)

Justification for trend: In view of the reduced thermal tolerance of subterranean specialised *Troglohyphantes* species (Mammola et al. 2019a), alterations of the microclimatic conditions of the habitat due to climate change are expected to impact the whole population of this species.

Basis for decline: (c) a decline in area of occupancy, extent of occurrence and/or quality of habitat

Causes ceased?: No

Causes understood?: Yes

Causes reversible?: No

Extreme fluctuations?: No

#### Subpopulations

Number of subpopulations: 1

Trend: Stable

Justification for trend: Because of the adaptation to the subterranean habitat and the narrow thermal tolerance of highly adapted *Troglohyphantes*, likely hampering dispersal through non-subterranean habitats, each subpopulation of *T.spinipes* can reasonably occur in a single isolated or few contiguous localities. Accordingly, for this species we identified a single subpopulation occurring in a few caves in the Kočevje mountains, in Lower Carniola.

Extreme fluctuations?: No

Severe fragmentation?: No

#### Habitat

System: Terrestrial

Habitat specialist: Yes

Habitat (narrative): Specimens have been collected in caves, no additional information about the habitat was found in literature.

Trend in extent, area or quality?: Decline (inferred)

Justification for trend: As seen in Mammola et al. (2018a) for the *Troglohyphantes* of the Western Alps, a drastic decline in the habitat suitability of *T.spinipes* as a consequence of climate change is expected.

##### Habitat

Habitat importance: Major Importance

Habitats: 7. Caves and Subterranean Habitats (non-aquatic)

#### Ecology

Size: 3.4 mm

Generation length (yr): 4

Dependency of single sp?: No

Ecology and traits (narrative): This spider shows minor morphological specialisation to subterranean life (Mammola et al. 2022a).

#### Threats

Justification for threats: This species is potentially exposed due to its extremely narrow geographic distribution range. As seen for other *Troglohyphantes* species (Mammola et al. 2018a), climate warming is expected to reduce the currently suitable habitat for this spider. Moreover, in view of the reduced thermal tolerance of subterranean specialised species of *Troglohyphantes* (Mammola et al. 2019a), these species have a limited dispersal ability, which represents an additional concern in face of the ongoing increase of temperature.

##### Threats

Threat type: Future

Threats: 11.1. Climate change & severe weather - Habitat shifting & alteration11.2. Climate change & severe weather - Droughts11.3. Climate change & severe weather - Temperature extremes

#### Conservation

Justification for conservation actions: This species was listed in the first IUCN Red List (IUCN Conservation Monitoring Centre 1986) due to its restricted geographical distribution, and was assessed as Vulnerable in the 1996 IUCN Red List (World Conservation Monitoring Centre 1996c), but its status has not been updated since. In addition, this species figures in the Slovenian national Red List due to its rarity (category R) (Uradni list RS št. 82/02 in 42/10). All the known localities of this species occur within the Special Area of Conservation and Special Protection Area of Kočevsko (SAC SI3000263 and SPA SI5000013).

##### Conservation actions

Conservation action type: In Place

Conservation actions: 1.1. Land/water protection - Site/area protection1.2. Land/water protection - Resource & habitat protection2.1. Land/water management - Site/area management

#### Other

##### Use and trade

Use type: International

##### Ecosystem services

Ecosystem service type: Important

##### Research needed

Research needed: 1.1. Research - Taxonomy1.2. Research - Population size, distribution & trends1.3. Research - Life history & ecology1.5. Research - Threats

Justification for research needed: The taxonomical status of the species and possible synonymy with *T.similis* is going to be clarified (see Pavlek et al. *in litteris*). Research on basic information such as natural history, ecology and possible threats of the species would be needed.

### Troglohyphantes subalpinus

#### Species information

Scientific name: Troglohyphantessubalpinus

Species authority: Thaler, 1967

Kingdom: Animalia

Phylum: Arthropoda

Class: Arachnida

Order: Araneae

Family: Linyphiidae

**Figure 13.**
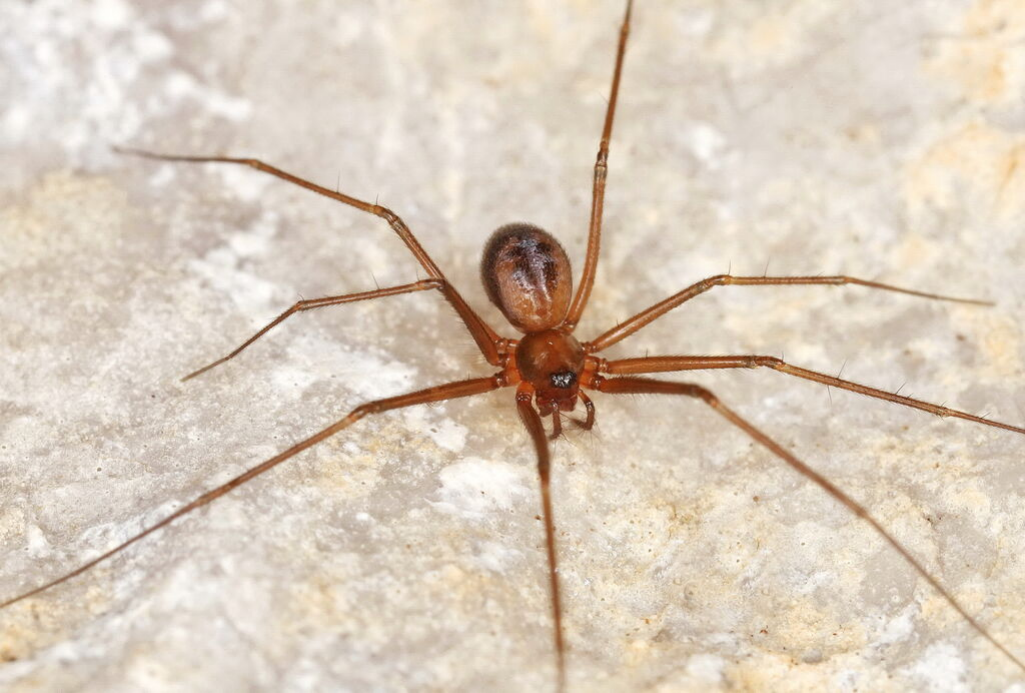
*Troglohyphantessubalpinus* Thaler, 1967. Photo credit: Christian Komposch.

Figure(s) or Photo(s): Fig. 13

Region for assessment: Global

#### Editor & Reviewers

##### Reviewers

Reviewers: Marc MilnePaulo Borges

##### Editor

Editor: Pedro Cardoso

#### Geographic range

Biogeographic realm: Palearctic

Countries: GermanySloveniaAustriaItaly

Map of records (Google Earth): Suppl. material 58

Basis of EOO and AOO: Species Distribution Model

Basis (narrative): This species is known from multiple localities. Therefore, it was possible to perform species distribution modelling to predict its potential range with confidence limits. See Methods for details.

Min Elevation/Depth (m): 372

Max Elevation/Depth (m): 2018

Range description: This species is widespread in Austria. It has also been found in Trentino-Alto Adige (north-eastern Italy), in the Berchtesgaden Alps (southern Germany), and in Slovenia (detailed occurrences and relative references in Suppl. material 67). The records of this species from Croatia (Pavlek and Ozimec 2009) are considered misidentifications, and therefore have not been included in the species distribution model.

#### Extent of occurrence

EOO (km2): 53656-80815,61037

Trend: Stable

Justification for trend: This species has been mainly collected in forests, prairies, shrublands, and in river banks. It is plausible that anthropogenic climate change may affect the habitat suitability of this species. However, in view of the relatively wide thermal tolerance and the relatively high dispersal ability of non-specialised species of *Troglohyphantes* (Mammola et al. 2019a), the distribution range of *T.subalpinus* is not expected to undergo significant reduction in the near future. A deeper study on the impacts of climate change on this species is required.

Causes ceased?: Yes

Causes understood?: Yes

Causes reversible?: Yes

Extreme fluctuations?: No

#### Area of occupancy

Trend: Stable

Justification for trend: This species has been mainly collected in forests, prairies, shrublands, and in river banks. It is plausible that anthropogenic climate change may affect the habitat suitability of this species. However, in view of the relatively wide thermal tolerance and the relatively high dispersal ability of non-specialised species of *Troglohyphantes* (Mammola et al. 2019a), the distribution range of *T.subalpinus* is not expected to undergo significant reduction in the near future. A deeper study on the impacts of climate change on this species is required.

Causes ceased?: Yes

Causes understood?: Yes

Causes reversible?: Yes

Extreme fluctuations?: No

AOO (km2): 33860-50940,43116

#### Locations

Number of locations: Not applicable

Justification for number of locations: No known major threats to this species.

Trend: Stable

Extreme fluctuations?: No

#### Population

Number of individuals: Unknown

Trend: Stable

Justification for trend: There are no currently known threats to the species.

Causes ceased?: Yes

Causes understood?: Yes

Causes reversible?: Yes

Extreme fluctuations?: No

#### Subpopulations

Number of subpopulations: Unknown

Trend: Unknown

Extreme fluctuations?: No

Severe fragmentation?: No

#### Habitat

System: Terrestrial

Habitat specialist: No

Habitat (narrative): This species occurs in forests, prairies, and shrublands (*Rhododendron* and *Vaccinium*) of the montane and subalpine belt, usually in shaded river and stream banks. Specimens have been collected in shaded overgrown rock-faults, in crevices of boulders, in moss, and in soil litter under rocks.

Trend in extent, area or quality?: Stable

Justification for trend: Many of the habitats that this spiders inhabits are affected by direct human activities, such as forestry and silvicultural measures, watercourse exploitation and water dynamics alterations due to hydraulic engineering and water use (Komposch 2009). However, considering its low degree of subterranean specialisation and its wide distribution range, *T.subalpinus* is not expected to experience any decline.

##### Habitat

Habitat importance: Major Importance

Habitats: 1.4. Forest - Temperate3.4. Shrubland - Temperate4.4. Grassland - Temperate5.1. Wetlands (inland) - Permanent Rivers/Streams/Creeks (includes waterfalls)6. Rocky areas (e.g. inland cliffs, mountain peaks)

#### Ecology

Size: 2.8 mm

Generation length (yr): 2

Dependency of single sp?: No

Ecology and traits (narrative): Not much is known about the ecology and life history of this species. This spider shows a lower degree of specialisation to subterranean life (Mammola et al. 2022a).

#### Threats

Justification for threats: No known major threats to this species.

##### Threats

Threat type: Past

Threats: 12. Other options - Other threat

#### Conservation

Justification for conservation actions: The potential distribution of *T.subalpinus* falls within several national parks, protected areas, and sites of the Natura 2000 network. In Austria, this species has been listed in the 59th Regulation of the Carinthian State Government of 2015 (LGBl. Nr. 59/2015), among the species fully protected according to the Carinthian Nature Conservation Act 2002 (LGBl. Nr. 79/2002). In Germany, it figures in the category R of the national Red List of spiders (Blick et al. 2016) and in the regional Red List of Bavaria (Blick and Scheidler 2004).

##### Conservation actions

Conservation action type: In Place

Conservation actions: 1.1. Land/water protection - Site/area protection1.2. Land/water protection - Resource & habitat protection2.1. Land/water management - Site/area management

#### Other

##### Use and trade

Use type: International

##### Ecosystem services

Ecosystem service type: Important

##### Research needed

Research needed: 1.2. Research - Population size, distribution & trends1.3. Research - Life history & ecology1.5. Research - Threats

Justification for research needed: Research on basic information such as distribution, ecology, life cycle and possible threats throughout the potential range would be needed.

### Troglohyphantes tauriscus

#### Species information

Scientific name: Troglohyphantestauriscus

Species authority: Thaler, 1982

Kingdom: Animalia

Phylum: Arthropoda

Class: Arachnida

Order: Araneae

Family: Linyphiidae

**Figure 14.**
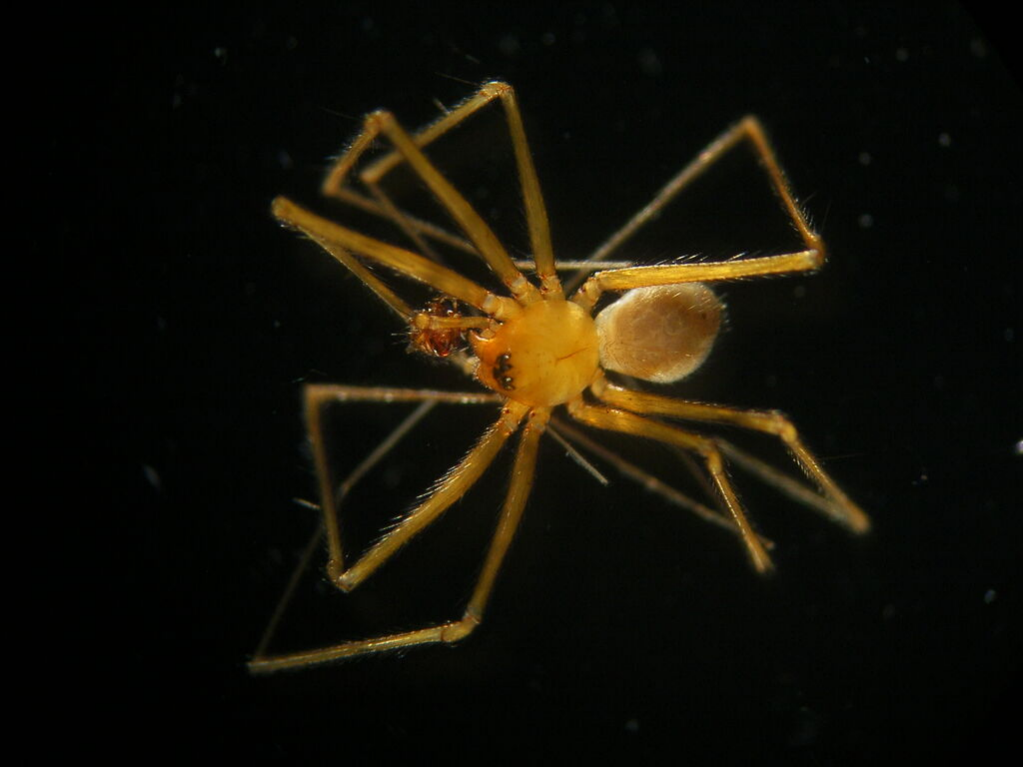
*Troglohyphantestauriscus* Thaler, 1982. Photo credit: Christian Komposch.

Figure(s) or Photo(s): Fig. 14

Region for assessment: Global

#### Editor & Reviewers

##### Reviewers

Reviewers: Marc MilnePaulo Borges

##### Editor

Editor: Pedro Cardoso

#### Geographic range

Biogeographic realm: Palearctic

Countries: Austria

Map of records (Google Earth): Suppl. material 59

Basis of EOO and AOO: Observed

Basis (narrative): This species was collected in a few localities. Its low level of subterranean specialisation, together with the high altimetric range found in its distribution, possibly reflects a higher dispersal capacity when compared to subterranean specialised species. Consequently, it may be possible that the present known range of this species is underestimated.

Min Elevation/Depth (m): 750

Max Elevation/Depth (m): 1960

Range description: This species is known from a few epigean localities occurring in a small range in the Austrian Central-eastern Alps, from the High Tauern to the Lower Tauern, with a single southern record in a cave in the Villacher Alps (detailed occurrences and relative references in Suppl. material 67).

#### Extent of occurrence

EOO (km2): 5789

Trend: Decline (inferred)

Justification for trend: Many of the habitats that this spiders inhabits are affected by forestry and silvicultural measures.

Causes ceased?: No

Causes understood?: Yes

Causes reversible?: Yes

Extreme fluctuations?: No

#### Area of occupancy

Trend: Decline (inferred)

Justification for trend: Many of the habitats that this spiders inhabits are affected by forestry and silvicultural measures.

Causes ceased?: No

Causes understood?: Yes

Causes reversible?: Yes

Extreme fluctuations?: No

AOO (km2): 44

#### Locations

Number of locations: Unknown

Justification for number of locations: Data on the distribution range of this species are not enough to estimate the precise number of locations

Trend: Decline (inferred)

Justification for trend: Many of the localities of this spider are affected by forestry and silvicultural measures.

Extreme fluctuations?: No

#### Population

Number of individuals: Unknown

Trend: Decline (inferred)

Justification for trend: The population size is inferred to be declining probably due to the degradation of habitat caused by forestery and silvicultural practices.

Basis for decline: (c) a decline in area of occupancy, extent of occurrence and/or quality of habitat

Causes ceased?: No

Causes understood?: Yes

Causes reversible?: Yes

Extreme fluctuations?: No

#### Subpopulations

Number of subpopulations: Unknown

Trend: Unknown

Extreme fluctuations?: No

Severe fragmentation?: No

#### Habitat

System: Terrestrial

Habitat specialist: No

Habitat (narrative): This species has been mainly found in rock crevices, among rocky debris, and in moss under roots in montane forests. This species has been also found in a cave near Villach, in Dobratsch.

Trend in extent, area or quality?: Unknown

Justification for trend: Many of the forest habitats where this spiders has been found are affected by forestry and silvicultural measures (Komposch 2009).

##### Habitat

Habitat importance: Major Importance

Habitats: 1.4. Forest - Temperate3.4. Shrubland - Temperate7. Caves and Subterranean Habitats (non-aquatic)

#### Ecology

Size: 2.5 mm

Generation length (yr): 2

Dependency of single sp?: No

Ecology and traits (narrative): This spider shows minor specialisation to subterranean life (Mammola et al. 2022a).

#### Threats

Justification for threats: The habitats where this species occurs are currently affected by alteration due to forestry and silvicultural measures (Komposch 2009).

##### Threats

Threat type: Ongoing

Threats: 5.3. Biological resource use - Logging & wood harvesting

#### Conservation

Justification for conservation actions: Some of the localities fall within protected areas (WDPA ID 555596205, SAC/SPA AT3210001 Hohe Tauern, SPA AT2209000 Niedere Tauern, SAC/SPA AT2120000 Schütt - Graschelitzen). *Troglohyphantestauriscus* was assessed in the category R of the Red List of endangered spiders for Carinthia, as considered extremely rare in the region (Komposch and Steinberger 1999). In addition, it figures in the 59th Regulation of the Carinthian State Government of 2015 (LGBl. Nr. 59/2015), among the species fully protected from capture, collection, killing, and disturbance according to the Carinthian Nature Conservation Act 2002 (LGBl. Nr. 79/2002).

##### Conservation actions

Conservation action type: In Place

Conservation actions: 1.1. Land/water protection - Site/area protection1.2. Land/water protection - Resource & habitat protection2.1. Land/water management - Site/area management

#### Other

##### Use and trade

Use type: International

##### Ecosystem services

Ecosystem service type: Important

##### Research needed

Research needed: 1.2. Research - Population size, distribution & trends1.3. Research - Life history & ecology1.5. Research - Threats

Justification for research needed: Research on basic information such as distribution, natural history, ecology and possible threats of the species would be needed.

### Troglohyphantes thaleri

#### Species information

Scientific name: Troglohyphantesthaleri

Species authority: Miller & Polenec, 1975

Kingdom: Animalia

Phylum: Arthropoda

Class: Arachnida

Order: Araneae

Family: Linyphiidae

Region for assessment: Global

#### Editor & Reviewers

##### Reviewers

Reviewers: Marc MilnePaulo Borges

##### Editor

Editor: Pedro Cardoso

#### Geographic range

Biogeographic realm: Palearctic

Countries: SloveniaAustria

Map of records (Google Earth): Suppl. material 60

Basis of EOO and AOO: Species Distribution Model

Basis (narrative): Multiple collection sites are recorded for this species. Therefore, it was possible to perform species distribution modelling to predict its potential range with confidence limits. See Methods for details.

Min Elevation/Depth (m): 210

Max Elevation/Depth (m): 2212

Range description: This species has been mainly recorded in the Austrian Eastern Alps. In Slovenia, *T.thaleri* has been only found in a forest south of Ljutomer, Styria (detailed occurrences and relative references in Suppl. material 67).

#### Extent of occurrence

EOO (km2): 20847-29372,23156

Trend: Stable

Justification for trend: *Troglohyphantesthaleri* has been collected in a wide range of epigean habitats, from forests to alpine pastures and rocky lands. It is plausible that climate change may affect the habitat suitability of this species. However, in view of the relatively wide thermal tolerance and the relatively high dispersal ability of non-specialised species of *Troglohyphantes* (Mammola et al. 2019a), the distribution range of *T.thaleri* is not expected to undergo significant reduction in the near future. A deeper study on the impacts of climate change on this species is required.

Causes ceased?: Yes

Causes understood?: Yes

Causes reversible?: Yes

Extreme fluctuations?: No

#### Area of occupancy

Trend: Stable

Justification for trend: *Troglohyphantesthaleri* has been collected in a wide range of epigean habitats, from forests to alpine pastures and rocky lands. It is plausible that climate change may affect the habitat suitability of this species. However, in view of the relatively wide thermal tolerance and the relatively high dispersal ability of non-specialised species of *Troglohyphantes* (Mammola et al. 2019a), the distribution range of *T.thaleri* is not expected to undergo significant reduction in the near future. A deeper study on the impacts of climate change on this species is required.

Causes ceased?: Yes

Causes understood?: Yes

Causes reversible?: Yes

Extreme fluctuations?: No

AOO (km2): 11592-16012,14508

#### Locations

Number of locations: Not applicable

Justification for number of locations: There are no currently known threats to this species.

Trend: Stable

Extreme fluctuations?: No

#### Population

Number of individuals: Unknown

Trend: Stable

Justification for trend: There are no currently known threats to this species.

Causes ceased?: Yes

Causes understood?: Yes

Causes reversible?: Yes

Extreme fluctuations?: No

#### Subpopulations

Number of subpopulations: Unknown

Trend: Unknown

Extreme fluctuations?: No

Severe fragmentation?: No

#### Habitat

System: Terrestrial

Habitat specialist: No

Habitat (narrative): This spider has been recorded in a wide range of habitats, such as in the litter of mixed beech-fir forests, in scrubby alpine pastures with mountain pines, in sparse spruce forests, and in rocky habitats such as at the base of detritus slope slides, in alpine screes and in dolines.

Trend in extent, area or quality?: Stable

Justification for trend: Despite some of the habitats where this species occurs are affected by direct human activities, such as forestry, silvicultural measures and watercourse exploitation (Komposch 2009), *T.thaleri* has a wide distribution range and seems to adapt to various habitat types, therefore is not expected to experience any decline.

##### Habitat

Habitat importance: Major Importance

Habitats: 1.4. Forest - Temperate3.4. Shrubland - Temperate4.4. Grassland - Temperate6. Rocky areas (e.g. inland cliffs, mountain peaks)7.2. Caves and Subterranean Habitats (non-aquatic) - Other Subterranean Habitats

#### Ecology

Size: 2.9 mm

Generation length (yr): 2

Dependency of single sp?: No

Ecology and traits (narrative): Specimens show a low degree of morphological specialisation to subterranean life (Mammola et al. 2022a).

#### Threats

Justification for threats: Some of the habitats in which *T.thaleri* occurs are currently affected by alteration due to forestry and silvicultural measures, and by watercourse exploitation and water dynamics alterations due to hydraulic engineering and water use (Komposch 2009). However, considering its wide distribution, these impacts do not constitute major threats to this species.

##### Threats

Threat type: Past

Threats: 12. Other options - Other threat

#### Conservation

Justification for conservation actions: The potential distribution of *T.thaleri* is covered by several protected areas and sites of the Natura 2000 network. In Slovenia, due to its extreme rarity, this species was considered as potentially threatened and included in the category R of the Slovenian Red List of endangered plant and animal species (Uradni list RS št. 82/02 in 42/10). In Austria, also, this species is listed in the category R of the Red List of endangered spiders for Carinthia (Komposch and Steinberger 1999). In addition, this species has been listed in the 59th Regulation of the Carinthian State Government of 2015 (LGBl. Nr. 59/2015), among the species fully protected from capture, collection, killing, and disturbance according to the Carinthian Nature Conservation Act 2002 (LGBl. Nr. 79/2002).

##### Conservation actions

Conservation action type: In Place

Conservation actions: 1.1. Land/water protection - Site/area protection1.2. Land/water protection - Resource & habitat protection2.1. Land/water management - Site/area management

#### Other

##### Use and trade

Use type: International

##### Ecosystem services

Ecosystem service type: Important

##### Research needed

Research needed: 1.2. Research - Population size, distribution & trends1.3. Research - Life history & ecology1.5. Research - Threats

Justification for research needed: Research on basic information such as distribution, ecology, life cycle and possible threats throughout the range would be needed.

### Troglohyphantes trispinosus

#### Species information

Scientific name: Troglohyphantestrispinosus

Species authority: Miller & Polenec, 1975

Kingdom: Animalia

Phylum: Arthropoda

Class: Arachnida

Order: Araneae

Family: Linyphiidae

Region for assessment: Global

#### Editor & Reviewers

##### Reviewers

Reviewers: Marc MilnePaulo Borges

##### Editor

Editor: Pedro Cardoso

#### Geographic range

Biogeographic realm: Palearctic

Countries: Slovenia

Map of records (Google Earth): Suppl. material 61

Basis of EOO and AOO: Observed

Basis (narrative): This species was collected in a few localities. Its low level of subterranean specialisation, together with the high altimetric range found in its distribution, possibly reflects a higher dispersal capacity when compared to subterranean specialised species. Consequently, it may be possible that the present known range of this species is underestimated.

Min Elevation/Depth (m): 650

Max Elevation/Depth (m): 1622

Range description: *Troglohyphantestrispinosus* has been collected in a few epigean localities in Upper Carniola, Slovenia (detailed occurrences and relative references in Suppl. material 67).

#### Extent of occurrence

EOO (km2): 207

Trend: Stable

Justification for trend: This species is not strictly relegated to deep subterranean habitats, being collected in epigean habitats. It is plausible that anthropogenic climate change may affect the habitat suitability of this species. However, in view of the relatively wide thermal tolerance and the relatively high dispersal ability of non-specialised species of *Troglohyphantes* (Mammola et al. 2019a), the distribution range of *T.trispinosus* is not expected to undergo significant reduction in the near future. A deeper study on the current distribution of this species and on the potential impacts of climate change is required.

Causes ceased?: Yes

Causes understood?: Yes

Causes reversible?: Yes

Extreme fluctuations?: No

#### Area of occupancy

Trend: Stable

Justification for trend: This species is not strictly relegated to deep subterranean habitats, being collected in epigean habitats. It is plausible that anthropogenic climate change may affect the habitat suitability of this species. However, in view of the relatively wide thermal tolerance and the relatively high dispersal ability of non-specialised species of *Troglohyphantes* (Mammola et al. 2019a), the distribution range of *T.trispinosus* is not expected to undergo significant reduction in the near future. A deeper study on the current distribution of this species and on the potential impacts of climate change is required.

Causes ceased?: Yes

Causes understood?: Yes

Causes reversible?: Yes

Extreme fluctuations?: No

AOO (km2): 36

#### Locations

Number of locations: Not applicable

Justification for number of locations: No known threats to this species.

Trend: Stable

Extreme fluctuations?: No

#### Population

Number of individuals: Unknown

Trend: Stable

Justification for trend: There are no currently known threats to the species.

Causes ceased?: Yes

Causes understood?: Yes

Causes reversible?: Yes

Extreme fluctuations?: No

#### Subpopulations

Number of subpopulations: Unknown

Trend: Unknown

Extreme fluctuations?: No

Severe fragmentation?: No

#### Habitat

System: Terrestrial

Habitat specialist: No

Habitat (narrative): Specimens have been found in humus and burrows of small mammals in thermophile beech forests.

Trend in extent, area or quality?: Unknown

##### Habitat

Habitat importance: Major Importance

Habitats: 1.4. Forest - Temperate

#### Ecology

Size: 2 mm

Generation length (yr): 2

Dependency of single sp?: No

Ecology and traits (narrative): Specimens show a low degree of morphological specialisation to subterranean life (Mammola et al. 2022a).

#### Threats

Justification for threats: The existence of threats is unknown for this species.

##### Threats

Threat type: Past

Threats: 12. Other options - Other threat

#### Conservation

Justification for conservation actions: This species was assessed as potentially threatened due to its rarity and listed in the category R of the Slovenian Red List of endangered plant and animal species (Uradni list RS št. 82/02 in 42/10). Some records have been collected inside the Natura 2000 network (SAC SI3000110 Ratitovec, SAC SI3000119 Porezen, SAC SI3000381 Slatnik, SAC SI3000253 Julijske Alpe, SPA SI5000019 Julijci, SAC SI3000102 Ledina na Jelovici, SPA SI5000001 Jelovica).

##### Conservation actions

Conservation action type: In Place

Conservation actions: 1.1. Land/water protection - Site/area protection1.2. Land/water protection - Resource & habitat protection2.1. Land/water management - Site/area management

#### Other

##### Use and trade

Use type: International

##### Ecosystem services

Ecosystem service type: Important

##### Research needed

Research needed: 1.2. Research - Population size, distribution & trends1.3. Research - Life history & ecology1.5. Research - Threats

Justification for research needed: Research on basic information such as distribution, natural history, ecology and possible threats of the species would be needed.

### Troglohyphantes typhlonetiformis

#### Species information

Scientific name: Troglohyphantestyphlonetiformis

Species authority: Absolon & Kratochvil, 1932

Kingdom: Animalia

Phylum: Arthropoda

Class: Arachnida

Order: Araneae

Family: Linyphiidae

Region for assessment: Global

#### Editor & Reviewers

##### Reviewers

Reviewers: Marc MilnePaulo Borges

##### Editor

Editor: Pedro Cardoso

#### Geographic range

Biogeographic realm: Palearctic

Countries: SloveniaAustria

Map of records (Google Earth): Suppl. material 62

Basis of EOO and AOO: Observed

Basis (narrative): In light of its high level of subterranean specialisation, we assume that the known records of *T.typhlonetiformis* are good proxies for defining the AOO and EOO of this species.

Min Elevation/Depth (m): 480

Max Elevation/Depth (m): 800

Range description: This species is known from four hypogean localities in Upper Carniola (northern Slovenia) and in Carinthia (southern Austria) (detailed occurrences and relative references in Suppl. material 67).

#### Extent of occurrence

EOO (km2): 170

Trend: Decline (inferred)

Justification for trend: According to Mammola et al. (2018a), climate change will significantly affect the distribution of subterranean specialised *Troglohyphantes* in the future. Moreover, given the low tolerance to habitat changes of these species as well as their very low dispersal ability, a possible extreme reduction of the geographic range is expected in the future.

Causes ceased?: No

Causes understood?: Yes

Causes reversible?: No

Extreme fluctuations?: No

#### Area of occupancy

Trend: Decline (inferred)

Justification for trend: According to Mammola et al. (2018a), climate change will significantly affect the distribution of subterranean specialised *Troglohyphantes* in the future. Moreover, given the low tolerance to habitat changes of these species as well as their very low dispersal ability, a possible extreme reduction of the geographic range is expected in the future.

Causes ceased?: No

Causes understood?: Yes

Causes reversible?: No

Extreme fluctuations?: No

AOO (km2): 16

#### Locations

Number of locations: 1

Justification for number of locations: Even though this species occurs in different caves, these are interpreted as a single location, as they are all affected by changes in subterranean microclimatic conditions due to climate change.

Trend: Stable

Extreme fluctuations?: No

#### Population

Number of individuals: Unknown

Trend: Decline (inferred)

Justification for trend: In view of the reduced thermal tolerance of subterranean specialised *Troglohyphantes* species (Mammola et al. 2019a), alterations of the microclimatic conditions of the habitat due to climate change are expected to impact the whole population of this species.

Basis for decline: (c) a decline in area of occupancy, extent of occurrence and/or quality of habitat

Causes ceased?: No

Causes understood?: Yes

Causes reversible?: No

Extreme fluctuations?: No

#### Subpopulations

Number of subpopulations: 3

Trend: Decline (inferred)

Justification for trend: Because of the adaptation to the subterranean habitat and the narrow thermal tolerance of highly adapted *Troglohyphantes*, likely hampering dispersal through non-subterranean habitats, each subpopulation of *T.typhlonetiformis* can reasonably occur in single isolated or few contiguous localities. Accordingly, for this species we identified three subpopulations, two of them occurring in Upper Carniola (northern Slovenia), near the municipalities of Škofja Loka and Kranji, respectively (Absolon and Kratochvíl 1932, Deeleman-Reinhold 1978), and a further, isolated northern subpopulation occurring in one cave near Eisenkappel, in Carinthia (southern Austria) (Thaler 1999). All subpopulations are likely to be impacted by climate change.

Extreme fluctuations?: No

Severe fragmentation?: No

#### Habitat

System: Terrestrial

Habitat specialist: Yes

Habitat (narrative): This species has been found exclusively in cave habitats. No additional information on the habitat was provided.

Trend in extent, area or quality?: Decline (inferred)

Justification for trend: As seen in Mammola et al. (2018a) for the western alpine *Troglohyphantes* species, a drastic decline in the habitat suitability of *T.typhlonetiformis* as a consequence of climate change is expected.

##### Habitat

Habitat importance: Major Importance

Habitats: 7. Caves and Subterranean Habitats (non-aquatic)

#### Ecology

Size: 2.4 mm

Generation length (yr): 4

Dependency of single sp?: No

Ecology and traits (narrative): This species shows a high degree of specialisation to subterranean habitats, with absence of pigmentation and high regression of the eyes (Mammola et al. 2022a).

#### Threats

Justification for threats: This species is potentially exposed due to its extremely narrow geographic distribution range and its presumably low dispersal capacity. As seen for the *Troglohyphantes* species of the Western Alps (Mammola et al. 2018a), climate warming is expected to reduce the currently suitable habitat for this spider.

##### Threats

Threat type: Future

Threats: 11.1. Climate change & severe weather - Habitat shifting & alteration11.2. Climate change & severe weather - Droughts11.3. Climate change & severe weather - Temperature extremes

#### Conservation

Justification for conservation actions: In Slovenia, this species has been considered as potentially threatened and included in the category R of the national Red List of endangered species (Uradni list RS št. 82/02 in 42/10). The type locality of this species (Kevderca na Lubniku), falls within the Special Area of Conservation of the Lubnik (SAC SI3000206). In Austria, this species was assessed in the Red List of endangered spiders for Carinthia, as considered extremely rare in the region (Komposch and Steinberger 1999). In addition, *T.typhlonetiformis* figures in the 59th Regulation of the Carinthian State Government of 2015 (LGBl. Nr. 59/2015), which amends the Carinthian Nature Conservation Act (LGBl. Nr. 79/2002).

##### Conservation actions

Conservation action type: In Place

Conservation actions: 1.1. Land/water protection - Site/area protection1.2. Land/water protection - Resource & habitat protection2.1. Land/water management - Site/area management

#### Other

##### Use and trade

Use type: International

##### Ecosystem services

Ecosystem service type: Important

##### Research needed

Research needed: 1.2. Research - Population size, distribution & trends1.3. Research - Life history & ecology1.5. Research - Threats

Justification for research needed: Research on basic information such as natural history, ecology and possible threats of the species would be needed.

### Troglohyphantes vicinus

#### Species information

Scientific name: Troglohyphantesvicinus

Species authority: Miller & Polenec, 1975

Kingdom: Animalia

Phylum: Arthropoda

Class: Arachnida

Order: Araneae

Family: Linyphiidae

Region for assessment: Global

#### Editor & Reviewers

##### Reviewers

Reviewers: Marc MilnePaulo Borges

##### Editor

Editor: Pedro Cardoso

#### Geographic range

Biogeographic realm: Palearctic

Countries: Slovenia

Map of records (Google Earth): Suppl. material 63

Basis of EOO and AOO: Observed

Basis (narrative): This species was collected only in two localities. Its low level of subterranean specialisation, possibly reflects a higher dispersal capacity when compared to subterranean specialised species. Given this situation, any modelling of the current habitat suitability is unreliable, and the known distribution range should be taken with caution. Further research is needed on this species in order to assess its extinction risk.

Min Elevation/Depth (m): 1300

Max Elevation/Depth (m): 1600

Range description: This species is known exclusively from the Mount Ratitovec, in Upper Carniola, Slovenia (see Suppl. material 67).

#### Extent of occurrence

EOO (km2): 8

Trend: Unknown

Causes ceased?: Unknown

Causes understood?: Unknown

Causes reversible?: Unknown

Extreme fluctuations?: No

#### Area of occupancy

Trend: Unknown

Causes ceased?: Unknown

Causes understood?: Unknown

Causes reversible?: Unknown

Extreme fluctuations?: No

AOO (km2): 8

#### Locations

Number of locations: Unknown

Justification for number of locations: The data available are not enough to estimate the number of locations for this species.

Trend: Unknown

Extreme fluctuations?: No

#### Population

Number of individuals: Unknown

Trend: Unknown

Justification for trend: The population size and trend are unknown.

Causes ceased?: Unknown

Causes understood?: Unknown

Causes reversible?: Unknown

Extreme fluctuations?: No

#### Subpopulations

Number of subpopulations: Unknown

Trend: Unknown

Extreme fluctuations?: No

Severe fragmentation?: No

#### Habitat

System: Terrestrial

Habitat specialist: No

Habitat (narrative): Specimens have been found in small mammal burrows, in beech forest litter.

Trend in extent, area or quality?: Unknown

##### Habitat

Habitat importance: Major Importance

Habitats: 1.4. Forest - Temperate

#### Ecology

Size: 2.3 mm

Generation length (yr): 2

Dependency of single sp?: No

Ecology and traits (narrative): Not much is known about the ecology and life history of this species.

#### Threats

Justification for threats: Unknown threats.

##### Threats

Threat type: Past

Threats: 12. Other options - Other threat

#### Conservation

Justification for conservation actions: *T.vicinus* was considered as potentially threatened due to its rarity and included in the category R of the Slovenian Red List of endangered plant and animal species (Uradni list RS št. 82/02 in 42/10). The known records of this species fall within the Natura 2000 network (SAC SI3000110 Ratitovec, SPA SI5000001 Jelovica).

##### Conservation actions

Conservation action type: In Place

Conservation actions: 1.1. Land/water protection - Site/area protection1.2. Land/water protection - Resource & habitat protection2.1. Land/water management - Site/area management

#### Other

##### Use and trade

Use type: International

##### Ecosystem services

Ecosystem service type: Important

##### Research needed

Research needed: 1.2. Research - Population size, distribution & trends1.3. Research - Life history & ecology1.5. Research - Threats

Justification for research needed: Research on basic information such as distribution, natural history, ecology and possible threats of the species would be needed.

### Troglohyphantes vignai

#### Species information

Scientific name: Troglohyphantesvignai

Species authority: Brignoli, 1971

Kingdom: Animalia

Phylum: Arthropoda

Class: Arachnida

Order: Araneae

Family: Linyphiidae

**Figure 15.**
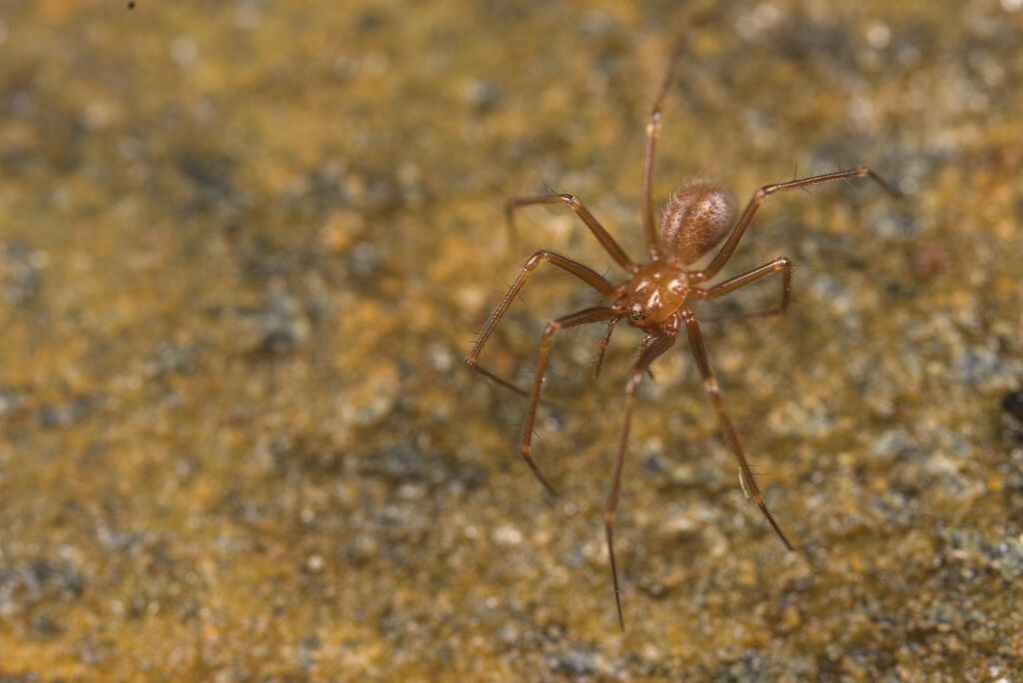
*Troglohyphantesvignai* Brignoli, 1971. Photo credit: Emanuele Biggi.

Figure(s) or Photo(s): Fig. 15

Region for assessment: Global

#### Editor & Reviewers

##### Reviewers

Reviewers: Marc MilnePaulo Borges

##### Editor

Editor: Pedro Cardoso

#### Geographic range

Biogeographic realm: Palearctic

Countries: Italy

Map of records (Google Earth): Suppl. material 64

Basis of EOO and AOO: Observed

Basis (narrative): Caves in Western Alps have been extensively sampled, allowing to define EOO and AOO of this species with reasonable confidence.

Min Elevation/Depth (m): 1140

Max Elevation/Depth (m): 2471

Range description: *Troglohyphantesvignai* has an area of distribution ranging from the Cottian Alps to the Northern Ligurian Alps, with a large distribution gap in the Maritime Alps (detailed occurrences and relative references in Suppl. material 67).

#### Extent of occurrence

EOO (km2): 832

Trend: Decline (inferred)

Justification for trend: As seen in Mammola et al. (2018a) for other species of the genus *Troglohyphantes* of the Western Alps, climate change is expected to affect the distribution of this species in the future. Given the reduced thermal tolerance of this organism and its low dispersal ability (Isaia et al. 2022), a reduction of its geographic distribution range is expected in the future.

Causes ceased?: No

Causes understood?: Yes

Causes reversible?: No

Extreme fluctuations?: No

#### Area of occupancy

Trend: Decline (inferred)

Justification for trend: As seen in Mammola et al. (2018a) for other species of the genus *Troglohyphantes* of the Western Alps, climate change is expected to affect the distribution of this species in the future. Given the reduced thermal tolerance of this organism and its low dispersal ability (Isaia et al. 2022), a reduction of its geographic distribution range is expected in the future.

Causes ceased?: No

Causes understood?: Yes

Causes reversible?: No

Extreme fluctuations?: No

AOO (km2): 48

#### Locations

Number of locations: 1

Justification for number of locations: The habitat where this species occurs is affected by changes in subterranean microclimatic conditions due to climate change, which is expected to impact the whole population (see Mammola et al. 2018a).

Trend: Stable

Extreme fluctuations?: No

#### Population

Number of individuals: Unknown

Trend: Decline (inferred)

Justification for trend: In view of the reduced thermal tolerance of this species (Isaia et al. 2022), alterations of the microclimatic conditions of the habitat due to climate change are expected to impact the whole population of this species.

Basis for decline: (c) a decline in area of occupancy, extent of occurrence and/or quality of habitat

Causes ceased?: No

Causes understood?: Yes

Causes reversible?: No

Extreme fluctuations?: No

#### Subpopulations

Number of subpopulations: 10

Trend: Decline (inferred)

Justification for trend: Because of the adaptation to the subterranean habitat and of the narrow physiological tolerance of this species, hampering dispersal through non-subterranean habitats (data on gene flow from Mammola et al. 2015), each subpopulation reasonably occurs in a single isolated or few contiguous localities. Accordingly, for this species we assumed at least ten subpopulations in the range, all of them occurring in the province of Cuneo (Piemonte, north-western Italy). All subpopulations are likely to be impacted by climate change.

Extreme fluctuations?: No

Severe fragmentation?: No

#### Habitat

System: Terrestrial

Habitat specialist: Yes

Habitat (narrative): *Troglohyphantesvignai* has been mainly found in the dark zone of natural caves or artificial subterranean habitats such as mines and bunkers. The caves where this species has been found are located at the medium alpine montane belt, from 1,140 up to 2,471 m, and are characterised by mean annual temperature values ranging from 7.3 to 12.1°C. In very few occasions, specimens were collected outside, in the vicinity of cave entrances in deep litter or under big stones (Isaia et al. 2022).

Trend in extent, area or quality?: Decline (inferred)

Justification for trend: As seen in Mammola et al. (2018a) for other species of the genus *Troglohyphantes* of the Western Alps, a drastic decline in the habitat suitability of *T.vignai* as a consequence of climate change is expected.

##### Habitat

Habitat importance: Major Importance

Habitats: 7. Caves and Subterranean Habitats (non-aquatic)

#### Ecology

Size: 3.2 mm

Generation length (yr): 4

Dependency of single sp?: No

Ecology and traits (narrative): Specimens of the southern subpopulations (Northern Ligurian Alps) exhibit a higher degree of specialisation to deep subterranean habitats compared with the northern subpopulations (Cottian Alps), with absence of pigmentation, eye reduction, and lowering of the cephalothorax profile (Isaia et al. 2022, Mammola et al. 2022a). According to thermal tests, *T.vignai* shows a great thermal tolerance, reaching 50% mortality at temperature values 8°C above its cave temperature (Isaia et al. 2022).

#### Threats

Justification for threats: This species is potentially exposed due to its extremely narrow geographic distribution range. As seen for other species of the genus *Troglohyphantes* of the Western Alps (Mammola et al. 2018a), climate warming is expected to reduce the currently suitable habitat for this spider. Moreover, in view of its reduced thermal tolerance (Isaia et al. 2022), this species has a limited dispersal ability, which represents an additional concern in face of the ongoing increase of temperature.

##### Threats

Threat type: Future

Threats: 11.1. Climate change & severe weather - Habitat shifting & alteration11.2. Climate change & severe weather - Droughts11.3. Climate change & severe weather - Temperature extremes

#### Conservation

Justification for conservation actions: Some of the caves where *T.vignai* has been recorded are included in the Natura 2000 network (EUAP0214 and SAC/SPA IT1160057 Alte Valli Pesio e Tanaro, SAC/SPA IT1160058 Gruppo del Monviso e Bosco dell'Alevè, SAC IT1160035 M. Antoroto).

##### Conservation actions

Conservation action type: In Place

Conservation actions: 1.1. Land/water protection - Site/area protection1.2. Land/water protection - Resource & habitat protection

#### Other

##### Use and trade

Use type: International

##### Ecosystem services

Ecosystem service type: Important

##### Research needed

Research needed: 1.2. Research - Population size, distribution & trends1.3. Research - Life history & ecology1.5. Research - Threats

Justification for research needed: Research on basic information such as natural history, ecology and possible threats of the species would be needed.

### Troglohyphantes wiehlei

#### Species information

Scientific name: Troglohyphanteswiehlei

Species authority: Miller & Polenec, 1975

Kingdom: Animalia

Phylum: Arthropoda

Class: Arachnida

Order: Araneae

Family: Linyphiidae

Region for assessment: Global

#### Editor & Reviewers

##### Reviewers

Reviewers: Marc MilnePaulo Borges

##### Editor

Editor: Pedro Cardoso

#### Geographic range

Biogeographic realm: Palearctic

Countries: CroatiaSloveniaAustria

Map of records (Google Earth): Suppl. material 65

Basis of EOO and AOO: Species Distribution Model

Basis (narrative): Multiple collection sites are known for this species. Therefore, it was possible to perform species distribution modelling to predict its potential range with confidence limits. See Methods for details.

Min Elevation/Depth (m): 148

Max Elevation/Depth (m): 1870

Range description: The distribution range of *T.wiehlei* extends from southern Austria across Slovenia to north-western Croatia (detailed occurrences and relative references in Suppl. material 67).

#### Extent of occurrence

EOO (km2): 10483-17564,13418

Trend: Stable

Justification for trend: This species has been mainly found in epigean environments, although a few records have been collected in hypogean habitats. In Croatia, the most south-eastern part of the distribution, specimens have been collected exclusively in caves, not far from the cave entrance. It is plausible that anthropogenic climate change may affect the habitat suitability of this species. However, in view of the relatively wide thermal tolerance and the relatively high dispersal ability of non-specialised species of *Troglohyphantes* (Mammola et al. 2019a), the distribution range of *T.wiehlei* is not expected to undergo significant reduction in the near future. A deeper study on the impacts of climate change on this species is required.

Causes ceased?: Yes

Causes understood?: Yes

Causes reversible?: Yes

Extreme fluctuations?: No

#### Area of occupancy

Trend: Stable

Justification for trend: This species has been mainly found in epigean environments, although a few records have been collected in hypogean habitats. In Croatia, the most south-eastern part of the distribution, specimens have been collected exclusively in caves, not far from the cave entrance. It is plausible that anthropogenic climate change may affect the habitat suitability of this species. However, in view of the relatively wide thermal tolerance and the relatively high dispersal ability of non-specialised species of *Troglohyphantes* (Mammola et al. 2019a), the distribution range of *T.wiehlei* is not expected to undergo significant reduction in the near future. A deeper study on the impacts of climate change on this species is required.

Causes ceased?: Yes

Causes understood?: Yes

Causes reversible?: Yes

Extreme fluctuations?: No

AOO (km2): 5152-12924,8444

#### Locations

Number of locations: Not applicable

Justification for number of locations: No known threats to this species.

Trend: Stable

Extreme fluctuations?: No

#### Population

Number of individuals: Unknown

Trend: Stable

Justification for trend: There are no currently known threats to the species.

Causes ceased?: Yes

Causes understood?: Yes

Causes reversible?: Yes

Extreme fluctuations?: No

#### Subpopulations

Number of subpopulations: Unknown

Trend: Unknown

Extreme fluctuations?: No

Severe fragmentation?: No

#### Habitat

System: Terrestrial

Habitat specialist: No

Habitat (narrative): This species has been mainly found in small mammal burrows, in moss, and under stones in mixed forests and shrublands. In the southern part of its range, it has been also collected in caves.

Trend in extent, area or quality?: Stable

Justification for trend: The habitats colonised by *T.wiehlei* are as yet not threatened by direct human activities.

##### Habitat

Habitat importance: Major Importance

Habitats: 1.4. Forest - Temperate3.4. Shrubland - Temperate4.4. Grassland - Temperate7. Caves and Subterranean Habitats (non-aquatic)

#### Ecology

Size: 2.5 mm

Generation length (yr): 2

Dependency of single sp?: No

Ecology and traits (narrative): Specimens show minor morphological adaptations to the subterranean life, with pigmentation and eyes normally developed (Mammola et al. 2022a).

#### Threats

Justification for threats: The threats to this species are unknown.

##### Threats

Threat type: Past

Threats: 12. Other options - Other threat

#### Conservation

Justification for conservation actions: The potential distribution of *T.wiehlei* is covered by several national parks, protected areas and sites of the Natura 2000 network. In Austria, this species was listed in the category R of the Red List of endangered spiders for Carinthia (Komposch and Steinberger 1999). In addition, this species has been listed in the 59th Regulation of the Carinthian State Government of 2015 (LGBl. Nr. 59/2015), and is fully protected by the Carinthian Nature Conservation Act 2002 (LGBl. Nr. 79/2002). In Slovenia, *T.wiehlei* was assessed as potentially threatened and included in the category R of the national Red List (Uradni list RS št. 82/02 in 42/10).

##### Conservation actions

Conservation action type: In Place

Conservation actions: 1.1. Land/water protection - Site/area protection1.2. Land/water protection - Resource & habitat protection2.1. Land/water management - Site/area management

#### Other

##### Use and trade

Use type: International

##### Ecosystem services

Ecosystem service type: Important

##### Research needed

Research needed: 1.2. Research - Population size, distribution & trends1.3. Research - Life history & ecology1.5. Research - Threats

Justification for research needed: Research on basic information such as natural history, ecology and possible threats of the species would be needed.

### Troglohyphantes zanoni

#### Species information

Scientific name: Troglohyphanteszanoni

Species authority: Pesarini, 1988

Kingdom: Animalia

Phylum: Arthropoda

Class: Arachnida

Order: Araneae

Family: Linyphiidae

Region for assessment: Global

#### Editor & Reviewers

##### Reviewers

Reviewers: Marc MilnePaulo Borges

##### Editor

Editor: Pedro Cardoso

#### Geographic range

Biogeographic realm: Palearctic

Countries: Italy

Map of records (Google Earth): Suppl. material 66

Basis of EOO and AOO: Observed

Basis (narrative): Despite the relatively high number of records of this non-specialised species, the species distribution predicted by the models was found to be unreliable by our own expert opinion. In view of this, only the observed distribution range is presented.

Min Elevation/Depth (m): 116

Max Elevation/Depth (m): 2020

Range description: This epigean species has a disjunct distribution in the Central Lombard Prealps and in the Colli Euganei, possibly relegated to the fragmentation of the original forest of the Po plain (*Quercus–Carpinetum*) (detailed occurrences and relative references in Suppl. material 67).

#### Extent of occurrence

EOO (km2): 7664

Trend: Stable

Justification for trend: This species has been mainly found in epigean environments, although a few records have been collected in hypogean habitats. It is plausible that climate change may affect the habitat suitability of this species. However, in view of the relatively wide thermal tolerance and the relatively high dispersal ability of non-specialised species of *Troglohyphantes* (Mammola et al. 2019a), the distribution range of *T.zanoni* is not expected to undergo significant reduction in the near future. A deeper study on the current distribution of this species and on the potential impacts of climate change is required.

Causes ceased?: Yes

Causes understood?: Yes

Causes reversible?: Yes

Extreme fluctuations?: No

#### Area of occupancy

Trend: Stable

Justification for trend: This species has been mainly found in epigean environments, although a few records have been collected in hypogean habitats. It is plausible that climate change may affect the habitat suitability of this species. However, in view of the relatively wide thermal tolerance and the relatively high dispersal ability of non-specialised species of *Troglohyphantes* (Mammola et al. 2019a), the distribution range of *T.zanoni* is not expected to undergo significant reduction in the near future. A deeper study on the current distribution of this species and on the potential impacts of climate change is required.

Causes ceased?: Yes

Causes understood?: Yes

Causes reversible?: Yes

Extreme fluctuations?: No

AOO (km2): 144

#### Locations

Number of locations: Not applicable

Justification for number of locations: No known threats to this species.

Trend: Stable

Extreme fluctuations?: No

#### Population

Number of individuals: Unknown

Trend: Stable

Justification for trend: There are no currently known threats to the species.

Causes ceased?: Yes

Causes understood?: Yes

Causes reversible?: Yes

Extreme fluctuations?: No

#### Subpopulations

Number of subpopulations: Unknown

Trend: Unknown

Justification for trend: The subpopulation occurring in the southern part of the range (Colli Euganei), is highly isolated within an intensively-managed agricultural matrix, hampering exchanges with other subpopulations occurring north, in the Central Lombard Prealps.

Extreme fluctuations?: No

Severe fragmentation?: No

#### Habitat

System: Terrestrial

Habitat specialist: No

Habitat (narrative): Specimens of *T.zanoni* have been frequently found at lower elevation in forests, while at higher elevation they have been found in a variety of cool habitats. Individuals have been collected also in hypogean habitats.

Trend in extent, area or quality?: Stable

Justification for trend: The habitats colonised by *T.zanoni* are as yet not threatened by direct human activities.

##### Habitat

Habitat importance: Major Importance

Habitats: 1.4. Forest - Temperate7. Caves and Subterranean Habitats (non-aquatic)

#### Ecology

Size: 2.4 mm

Generation length (yr): 2

Dependency of single sp?: No

Ecology and traits (narrative): Specimens show minor morphological specialisation to subterranean life, showing slight eye regression (Mammola et al. 2022a).

#### Threats

Justification for threats: The existence of threats is unknown for this species.

##### Threats

Threat type: Past

Threats: 12. Other options - Other threat

#### Conservation

Justification for conservation actions: The known records of *T.zanoni* fall within several sites of the Natura 2000 network (EUAP0243 Parco Regionale dei Colli Euganei and SAC/SPA IT3260017 Colli Euganei - Monte Lozzo - Monte Ricco, SAC IT2030002 Grigna Meridionale, SAC IT2060004 Alta Val di Scalve, SAC IT2060005 Val Sedornia - Val Zurio - Pizzo della Presolana, SAC IT2060009 Val Nossana - Cima di Grem, SPA IT2030601 Grigne, SPA IT2060401 Parco Regionale Orobie Bergamasche).

##### Conservation actions

Conservation action type: In Place

Conservation actions: 1.1. Land/water protection - Site/area protection1.2. Land/water protection - Resource & habitat protection2.1. Land/water management - Site/area management

#### Other

##### Use and trade

Use type: International

##### Ecosystem services

Ecosystem service type: Important

##### Research needed

Research needed: 1.2. Research - Population size, distribution & trends1.3. Research - Life history & ecology1.5. Research - Threats

Justification for research needed: Research on basic information such as natural history, ecology and possible threats of the species would be needed.

## Discussion

**Figure 16.**
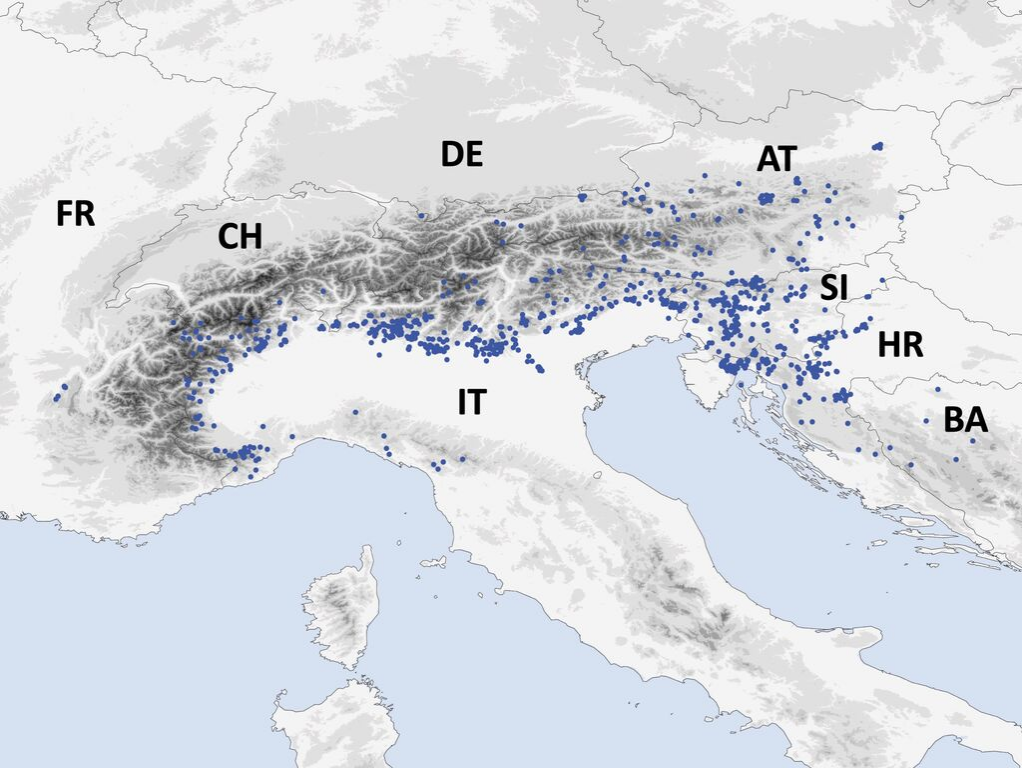
Map of the Alps and the north-western Dinarides, showing all records of *Troglohyphantes* spiders considered in this work. AT = Austria; BA = Bosnia and Herzegovina; CH = Switzerland; DE = Germany; FR = France; HR = Croatia; IT = Italy; SI = Slovenia.

In this work, we assessed the extinction risk of 66 species of spiders belonging to the genus *Troglohyphantes* occurring in the Alps and in the north-western Dinarides (Fig. 16) according to the IUCN Red List Criteria (IUCN 2001, IUCN 2012). In most cases (62 out of 66 species), the data in hand allowed us to calculate the Extent Of Occurrence (EOO) and the Area Of Occupancy (AOO).

For 46 species, we derived geographic information, based on the known occurrences. In other cases (16 species), the number of occurrences allowed the application of Species Distribution Models. Four species were missing critical information to assess their status, being known on one or very few specimens or never being recorded after their original description (*T.cavadinii*, *T.comottii*, *T.cruentus* and *T.pavesii*).

Most of the species considered have a very narrow distribution range (14 species occurring in only 1–2 localities), with an estimated EOO < 20,000 km^2^ and AOO < 2,000 km^2^, meeting the thresholds for the inclusion in the threatened categories (Fig. 17). Only five species are more widespread, with an estimated EOO > 20,000 km^2^ (Fig. 17).

**Figure 18.**
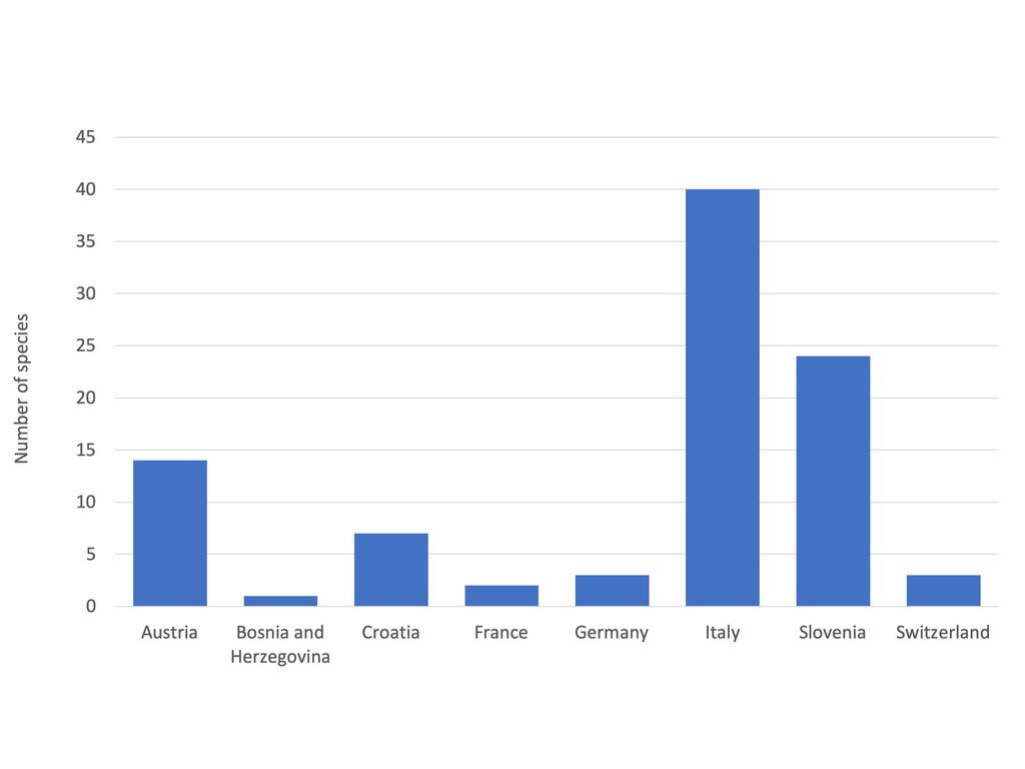
Number of the *Troglohyphantes* species considered by country.

Twenty species cover more than one country (seven of them more than two), whereas most of the species are restricted to one single country: 30 species occur only in Italy, 11 in Slovenia, three in Croatia, one in Austria and one in France (Fig. 18).

**Figure 19.**
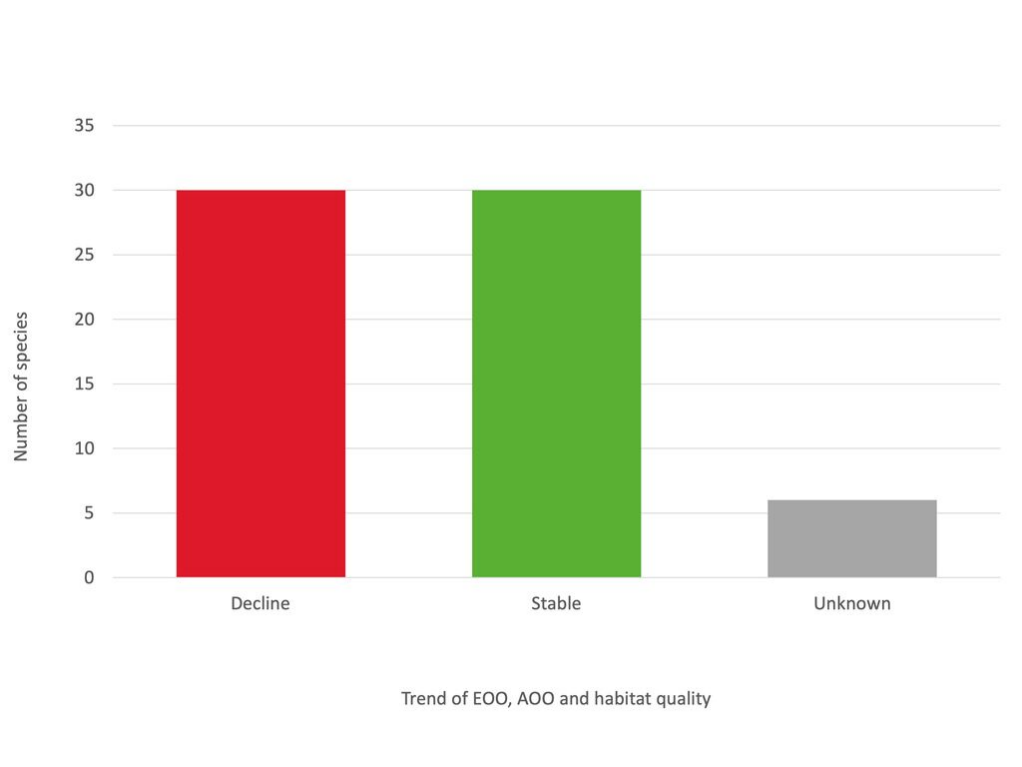
Trend of Extent Of Occurrence (EOO), Area Of Occupancy (AOO) and habitat quality of the *Troglohyphantes* species considered.

Trends in EOO, AOO and habitat quality were considered to be stable for 30 species (Fig. 19). These show minor adaptation to subterranean life, being mainly found in the vicinity of the cave entrance or in shallow subterranean habitats and for which no current major threats are known. It seems likely that these species are more able to withstand ecological variations and higher temperature increases compared to highly adapted subterranean species (Mammola et al. 2019a).

Although most of the species showing poor adaptation to subterranean life have relatively low extinction risk in view of their relatively wide thermal tolerance and the relatively high dispersal ability, there are a number of species with a very restricted distribution, requiring further research attention and protection measures.

For 30 species, we inferred a decline in the EOO, AOO and habitat quality (Fig. 19). The majority of them are subterranean specialised species, thus showing fine-tuned thermal tolerance to the constant and narrow temperature ranges of the subterranean habitat (Mammola et al. 2019a). In a climate change perspective, this ecological feature turns out to be a strong limitation, preventing subterranean adapted species to accommodate changing conditions by dispersal or to persist *in situ* (Mammola et al. 2018a, Mammola et al. 2019b). Subterranean habitats are more sensitive to perturbation than other habitat types. Anthropogenic global warming is expected to significantly influence and modify the underground climate (Badino 2004, Domínguez-Villar et al. 2014, Mammola et al. 2019b). Considering their high sensitivity to the potential subterranean climatic variation induced by climate change, a reduction in the extent of the distribution of *Troglohyphantes* specialised species is expected, as demonstrated by Mammola et al. (2018a) for the species of the Western Alps.

Quarrying activities represented the major threat to one species, *T.exul*, a highly adapted subterranean species occurring in a single cave, for which the nearby quarries are expected to cause critical damage to cave habitat, altering microclimate and decreasing overall habitat quality.

Habitat loss and land-use change due to urbanisation and infrastructure development are also considered to be important drivers of increased extinction risk in cave animals, although they were identified as the main reason for the declining AOO and EOO for two species only (*T.latzeli* and *T.liburnicus*). Urban development can impact habitats through physical degradation and fragmentation, pollution by solid and liquid municipal waste and railway and road construction, leading to drastic declines in natural populations.

The main factor in the decline of *T.tauriscus* was identified as forestry and silvicultural practices, which is expected to lead to a loss of habitat suitability in some subpopulations across the species’ range.

The high input agricultural activities represent the main threat for *T.spatulifer*, a species restricted to an isolated forest patch embedded in an intensively-managed agricultural landscape.

Forestry and intensive agricultural activities are also expected to negatively affect the habitat quality of other *Troglohyphantes* species in several areas, but it remains unclear whether they may represent major threats to the survival of these species.

For several subterranean species, a secondary impact has been recognised to be driven by tourism in caves. Cave tourism can have several impacts on the cave environment, such as physical modifications to cave habitats, marked fluctuations in temperature, changes in relative humidity and carbon dioxide concentrations and the possible accumulation of litter left by tourists in the cave (Fernandez-Cortes et al. 2011). These impacts negatively affect the subterranean ecological processes with cascade effects on all trophic levels.

**Figure 20.**
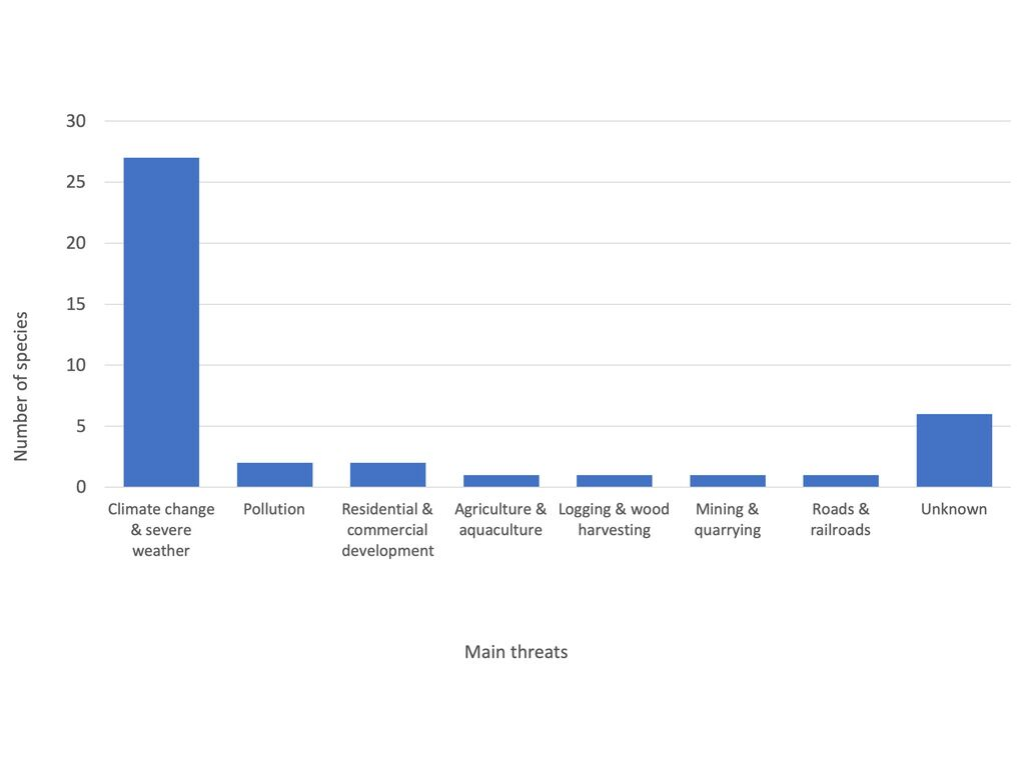
Main threats to the *Troglohyphantes* species assessed in this study.

The main threats to *Troglohyphantes* species are summarised in Fig. 20.

A considerable proportion of the species assessed has been found in protected areas and in sites of the Natura 2000 network. More than 80% of the species (53) here considered had at least one record in a site belonging to the Natura 2000 network and many of the subterranean occurrences have been collected in sites identified as Annex I habitats “Caves not open to the public” (Habitat 8310). However, most of the species assessed have a narrow distribution range that seldom overlaps with protected areas and many habitats of high conservation value for the *Troglohyphantes* species are poorly represented in the Natura 2000 network. Therefore, several species do not benefit from the Habitats Directive.

In addition, only 14 species (21%) are mentioned in national and sub-national legislation (Austria and Croatia). Almost all of them (13 species) have been listed in the 59th Regulation of the Carinthian State Government of 2015 (LGBl. Nr. 59/2015) and are fully protected from capture, collection, killing and disturbance according to the Carinthian Nature Conservation Act 2002 (LGBl. Nr. 79/2002). One species, *T.liburnicus*, is strictly legally protected in Croatia according to the Nature Protection Act (Official Gazette 70/05, NN 139/2008).

Of all species considered in this study, 25 (38%) are mentioned in regional Red Lists. Fifteen species are listed in the Red List of Slovenia (Uradni list RS št. 82/02 in 42/10) and considered potentially threatened due to their rarity (category R). Eight species are assessed in the Red List of endangered spiders for Carinthia (Komposch and Steinberger 1999), seven of which are considered extremely rare in the region (category R) and one is assessed as generally threatened (category G). Three species are listed in the category R of both the national Red List of spiders of Germany (Blick et al. 2016) and the regional Red List of Bavaria (Blick and Scheidler 2004). One species is assessed as Vulnerable in the Red Book of Croatian Cave Dwelling Fauna (Ozimec et al. 2009).

Three species (*T.gracilis*, *T.similis* and *T.spinipes*) are listed as Vulnerable in the IUCN Red List, based on their restricted geographical distribution (World Conservation Monitoring Centre 1996a, World Conservation Monitoring Centre 1996b, World Conservation Monitoring Centre 1996c). However, they were assessed before the publication of the new IUCN standards (version 3.1, 2001) and their evaluation is outdated and hard to compare with the assessments provided in this work.

One of the main shortfalls in the conservation of *Troglohyphantes* species is the general lack of biological information. As pointed out for spiders in Europe (Milano et al. 2021) and more generally for invertebrates (Cardoso et al. 2011), the majority of species are poorly known and the information is usually limited to their geographic distributions. Reliable data on population size are not available for any species of *Troglohyphantes* and their trends were mainly inferred by the decline in EOO and AOO. This makes the determination of the conservation status of these species difficult, with the risk of providing incomplete assessments and leaving many endangered species out from conservation planning and conservation priorities.

Long-term monitoring of species populations would be essential to provide key information on the population dynamics and their trends when assessing the species conservation (Mammola et al. 2022). Given the limited resources available for conservation and the need to maximise their allocation, monitoring the health of populations through a trait-based approach, such as the estimation of the stenothermic profiles of the species in relation to the variations of cave temperatures (Mammola et al. 2019b, Isaia et al. 2022) or the measurement of the species performance via morphological traits (Mammola et al. 2019a, Milano et al. in press), could help to reduce the knowledge gap (Lowe et al. 2020).

According to the threats identified for *Troglohyphantes* species, active management of the habitats more sensitive to perturbation is considered a priority for the conservation of the species. Subterranean specialised species, in particular, could benefit from general effective measures aiming at preserving the subterranean ecosystems (see Mammola et al. 2019c and Mammola et al. 2022). Although the protection of these habitats cannot limit the impact of climate change, the prevention or reduction of additional stress can increase the resilience of habitats and species to the effects of climate change.

Spiders have already been shown to be efficient and effective bioindicators in many terrestrial ecosystems (Bonte et al. 2002, Scott et al. 2006). Given their adaptations to the stringent conditions of the hypogean habitat (Deeleman-Reinhold 1978, Isaia et al. 2017, Mammola et al. 2019a, Mammola et al. 2020) and their vulnerability to microclimatic variations (Mammola et al. 2019b), subterranean specialised *Troglohyphantes* species could be potentially used as adequate bioclimatic indicators.

Discussion

## Supplementary Material

C621484E-EEB0-56C5-930A-10BCB55CBF50 10.3897/BDJ.10.e87261.suppl1 Supplementary material 1 Distribution of *Troglohyphantesachillis* Isaia & Mammola, 2021 Data type Distribution File: oo_615540.kml https://binary.pensoft.net/file/615540 Milano F., Borio L., Komposch C., Mammola S., Pantini P., Pavlek M., Isaia M.

ED9E8A13-9BD8-5157-8DB9-71D5A47789E7 10.3897/BDJ.10.e87261.suppl2 Supplementary material 2 Distribution of *Troglohyphantesalbopictus* Pesarini, 1989 Data type Distribution File: oo_615541.kml https://binary.pensoft.net/file/615541 Milano F., Borio L., Komposch C., Mammola S., Pantini P., Pavlek M., Isaia M.

A73DEDE5-08DD-58D3-AE1C-82991FA2A186 10.3897/BDJ.10.e87261.suppl3 Supplementary material 3 Distribution of *Troglohyphantesapenninicus* Isaia, Mammola & Pantini, 2017 Data type Distribution File: oo_615542.kml https://binary.pensoft.net/file/615542 Milano F., Borio L., Komposch C., Mammola S., Pantini P., Pavlek M., Isaia M.

66A9C9EA-C88C-524D-8044-90548797E25B 10.3897/BDJ.10.e87261.suppl4 Supplementary material 4 Distribution of *Troglohyphantesbolognai* Brignoli, 1975 Data type Distribution File: oo_615543.kml https://binary.pensoft.net/file/615543 Milano F., Borio L., Komposch C., Mammola S., Pantini P., Pavlek M., Isaia M.

314EAE41-1A39-5709-9447-4B590F9E8A1E 10.3897/BDJ.10.e87261.suppl5 Supplementary material 5 *Troglohyphantesbornensis* Isaia & Pantini, 2008 Data type Distribution File: oo_632084.kml https://binary.pensoft.net/file/632084 Milano F., Borio L., Komposch C., Mammola S., Pantini P., Pavlek M., Isaia M.

64821D5C-FDBB-53C3-9CFA-D162D5272596 10.3897/BDJ.10.e87261.suppl6 Supplementary material 6 Distribution of *Troglohyphantesbrignolii* Deeleman-Reinhold, 1978 Data type Distribution File: oo_615545.kml https://binary.pensoft.net/file/615545 Milano F., Borio L., Komposch C., Mammola S., Pantini P., Pavlek M., Isaia M.

A252730B-908D-563F-8706-CC9D087F88AC 10.3897/BDJ.10.e87261.suppl7 Supplementary material 7 Distribution of *Troglohyphantescaligatus* Pesarini, 1989 Data type Distribution File: oo_615546.kml https://binary.pensoft.net/file/615546 Milano F., Borio L., Komposch C., Mammola S., Pantini P., Pavlek M., Isaia M.

8F3F92A5-3DA5-5235-B6FA-02189D51E2B5 10.3897/BDJ.10.e87261.suppl8 Supplementary material 8 Distribution of *Troglohyphantescaporiaccoi* Brignoli, 1971 Data type Distribution File: oo_615557.kml https://binary.pensoft.net/file/615557 Milano F., Borio L., Komposch C., Mammola S., Pantini P., Pavlek M., Isaia M.

1734614B-77B4-5B42-9AF4-6C5DE46519F8 10.3897/BDJ.10.e87261.suppl9 Supplementary material 9 Distribution of *Troglohyphantescavadinii* Pesarini, 1989 Data type Distribution File: oo_615558.kml https://binary.pensoft.net/file/615558 Milano F., Borio L., Komposch C., Mammola S., Pantini P., Pavlek M., Isaia M.

455697B8-D3E0-59EB-9BAA-4946D41394E3 10.3897/BDJ.10.e87261.suppl10 Supplementary material 10 Distribution of *Troglohyphantescomottii* Pesarini, 1989 Data type Distribution File: oo_615560.kml https://binary.pensoft.net/file/615560 Milano F., Borio L., Komposch C., Mammola S., Pantini P., Pavlek M., Isaia M.

A24FFF38-E7FF-56D9-9230-5E0EBE4BEA54 10.3897/BDJ.10.e87261.suppl11 Supplementary material 11 Distribution of *Troglohyphantesconfusus* Kratochvíl, 1939 Data type Distribution File: oo_651142.kml https://binary.pensoft.net/file/651142 Milano F., Borio L., Komposch C., Mammola S., Pantini P., Pavlek M., Isaia M.

A72E5173-E0FF-5EDE-A03F-3960ACDFC664 10.3897/BDJ.10.e87261.suppl12 Supplementary material 12 Distribution of *Troglohyphantescroaticus* (Chyzer, 1894) Data type Distribution File: oo_615561.kml https://binary.pensoft.net/file/615561 Milano F., Borio L., Komposch C., Mammola S., Pantini P., Pavlek M., Isaia M.

05E63CA0-7F41-599A-A828-A3C04D42983D 10.3897/BDJ.10.e87261.suppl13 Supplementary material 13 Distribution of *Troglohyphantescruentus* Brignoli, 1971 Data type Distribution File: oo_615562.kml https://binary.pensoft.net/file/615562 Milano F., Borio L., Komposch C., Mammola S., Pantini P., Pavlek M., Isaia M.

6F717FB9-C85D-50F8-B2D1-A3638315774D 10.3897/BDJ.10.e87261.suppl14 Supplementary material 14 Distribution of *Troglohyphantesdelphinicus* Isaia & Mammola, 2021 Data type Distribution File: oo_615565.kml https://binary.pensoft.net/file/615565 Milano F., Borio L., Komposch C., Mammola S., Pantini P., Pavlek M., Isaia M.

C636D9C1-4AA4-5CDD-ACD3-84C37B1A639D 10.3897/BDJ.10.e87261.suppl15 Supplementary material 15 Distribution of *Troglohyphantesdiabolicus* Deeleman-Reinhold, 1978 Data type Distribution File: oo_684744.kml https://binary.pensoft.net/file/684744 Milano F., Borio L., Komposch C., Mammola S., Pantini P., Pavlek M., Isaia M.

36CDA094-3310-5273-B46B-FE71CE1C0AF5 10.3897/BDJ.10.e87261.suppl16 Supplementary material 16 Distribution of *Troglohyphantesdominici* Pesarini, 1988 Data type Distribution File: oo_652831.kml https://binary.pensoft.net/file/652831 Milano F., Borio L., Komposch C., Mammola S., Pantini P., Pavlek M., Isaia M.

F12230FF-AB0A-572C-907C-8880FEE0207A 10.3897/BDJ.10.e87261.suppl17 Supplementary material 17 Distribution of *Troglohyphantesexcavatus* Fage, 1919 Data type Distribution File: oo_615579.kml https://binary.pensoft.net/file/615579 Milano F., Borio L., Komposch C., Mammola S., Pantini P., Pavlek M., Isaia M.

7273AAB7-7A4E-5640-953A-1E27735012C4 10.3897/BDJ.10.e87261.suppl18 Supplementary material 18 Distribution of *Troglohyphantesexul* Thaler, 1987 Data type Distribution File: oo_615581.kml https://binary.pensoft.net/file/615581 Milano F., Borio L., Komposch C., Mammola S., Pantini P., Pavlek M., Isaia M.

11A13E4B-DD56-59C8-A0FC-92C22AF50987 10.3897/BDJ.10.e87261.suppl19 Supplementary material 19 Distribution of *Troglohyphantesfagei* Roewer, 1931 Data type Distribution File: oo_615584.kml https://binary.pensoft.net/file/615584 Milano F., Borio L., Komposch C., Mammola S., Pantini P., Pavlek M., Isaia M.

7CE83390-4D09-54E2-9C2A-D847DBA9AE3D 10.3897/BDJ.10.e87261.suppl20 Supplementary material 20 Distribution of *Troglohyphantesfatalis* Pesarini, 1988 Data type Distribution File: oo_652966.kml https://binary.pensoft.net/file/652966 Milano F., Borio L., Komposch C., Mammola S., Pantini P., Pavlek M., Isaia M.

31E5D914-F786-5D89-A73F-83EC6D24DAD7 10.3897/BDJ.10.e87261.suppl21 Supplementary material 21 Distribution of *Troglohyphantesgamsi* Deeleman-Reinhold, 1978 Data type Distribution File: oo_615591.kml https://binary.pensoft.net/file/615591 Milano F., Borio L., Komposch C., Mammola S., Pantini P., Pavlek M., Isaia M.

9C42E7EB-E378-5FE5-9F59-A77EADED0330 10.3897/BDJ.10.e87261.suppl22 Supplementary material 22 Distribution of *Troglohyphantesgestroi* Fage, 1933 Data type Distribution File: oo_678351.kml https://binary.pensoft.net/file/678351 Milano F., Borio L., Komposch C., Mammola S., Pantini P., Pavlek M., Isaia M.

3AFB2C65-FB15-5B0D-9FF8-DDC4138D457B 10.3897/BDJ.10.e87261.suppl23 Supplementary material 23 *Troglohyphantesgiachinoi* Isaia & Mammola, 2018 Data type Distribution File: oo_632088.kml https://binary.pensoft.net/file/632088 Milano F., Borio L., Komposch C., Mammola S., Pantini P., Pavlek M., Isaia M.

AA6D2A45-FFED-58B1-9662-DF44E2864802 10.3897/BDJ.10.e87261.suppl24 Supplementary material 24 Distribution of *Troglohyphantesgracilis* Fage, 1919 Data type Distribution File: oo_615594.kml https://binary.pensoft.net/file/615594 Milano F., Borio L., Komposch C., Mammola S., Pantini P., Pavlek M., Isaia M.

DDCEA889-F3FB-56B2-B552-2BD808749272 10.3897/BDJ.10.e87261.suppl25 Supplementary material 25 Distribution of *Troglohyphanteshelsdingeni* Deeleman-Reinhold, 1978 Data type Distribution File: oo_615595.kml https://binary.pensoft.net/file/615595 Milano F., Borio L., Komposch C., Mammola S., Pantini P., Pavlek M., Isaia M.

F90E83BB-D23E-57BC-A6BF-AB92AB1002AD 10.3897/BDJ.10.e87261.suppl26 Supplementary material 26 Distribution of *Troglohyphanteshenroti* Dresco, 1956 Data type Distribution File: oo_615596.kml https://binary.pensoft.net/file/615596 Milano F., Borio L., Komposch C., Mammola S., Pantini P., Pavlek M., Isaia M.

25480F94-493F-5856-AE1A-FE4280FD83A7 10.3897/BDJ.10.e87261.suppl27 Supplementary material 27 Distribution of *Troglohyphantesiulianae* Brignoli, 1971 Data type Distribution File: oo_615597.kml https://binary.pensoft.net/file/615597 Milano F., Borio L., Komposch C., Mammola S., Pantini P., Pavlek M., Isaia M.

EB494494-CAC4-53EE-B24B-D3FA8941DB51 10.3897/BDJ.10.e87261.suppl28 Supplementary material 28 Distribution of *Troglohyphantesjamatus* Roewer, 1931 Data type Distribution File: oo_615598.kml https://binary.pensoft.net/file/615598 Milano F., Borio L., Komposch C., Mammola S., Pantini P., Pavlek M., Isaia M.

9976A83D-354E-5102-985D-5673B26846C6 10.3897/BDJ.10.e87261.suppl29 Supplementary material 29 Distribution of *Troglohyphantesjuris* Thaler, 1982 Data type Distribution File: oo_615600.kml https://binary.pensoft.net/file/615600 Milano F., Borio L., Komposch C., Mammola S., Pantini P., Pavlek M., Isaia M.

7830CDF7-53D5-585D-8E05-3E6295AA5538 10.3897/BDJ.10.e87261.suppl30 Supplementary material 30 Distribution of *Troglohyphanteskarawankorum* Deeleman-Reinhold, 1978 Data type Distribution File: oo_615601.kml https://binary.pensoft.net/file/615601 Milano F., Borio L., Komposch C., Mammola S., Pantini P., Pavlek M., Isaia M.

2A025B64-6DEE-596F-948C-A2EBAC3A2C1F 10.3897/BDJ.10.e87261.suppl31 Supplementary material 31 Distribution of *Troglohyphanteskonradi* Brignoli, 1975 Data type Distribution File: oo_615602.kml https://binary.pensoft.net/file/615602 Milano F., Borio L., Komposch C., Mammola S., Pantini P., Pavlek M., Isaia M.

F776655C-3ECA-5F7E-ABF3-3E27FDA0D37B 10.3897/BDJ.10.e87261.suppl32 Supplementary material 32 Distribution of *Troglohyphanteskordunlikanus* Deeleman-Reinhold, 1978 Data type Distribution File: oo_615604.kml https://binary.pensoft.net/file/615604 Milano F., Borio L., Komposch C., Mammola S., Pantini P., Pavlek M., Isaia M.

9ADFCEF0-AAD2-5675-9283-30891BD1CDA7 10.3897/BDJ.10.e87261.suppl33 Supplementary material 33 *Troglohyphanteslanai* Isaia & Pantini, 2010 Data type Distribution File: oo_632090.kml https://binary.pensoft.net/file/632090 Milano F., Borio L., Komposch C., Mammola S., Pantini P., Pavlek M., Isaia M.

EBA323A1-6BC6-5262-8C71-AAEEA657406C 10.3897/BDJ.10.e87261.suppl34 Supplementary material 34 Distribution of *Troglohyphanteslatzeli* Thaler, 1986 Data type Distribution File: oo_615607.kml https://binary.pensoft.net/file/615607 Milano F., Borio L., Komposch C., Mammola S., Pantini P., Pavlek M., Isaia M.

B0BDA937-DCB6-5B8B-9B40-1AB05A324312 10.3897/BDJ.10.e87261.suppl35 Supplementary material 35 Distribution of *Troglohyphanteslessinensis* Caporiacco, 1936 Data type Distribution File: oo_667110.kml https://binary.pensoft.net/file/667110 Milano F., Borio L., Komposch C., Mammola S., Pantini P., Pavlek M., Isaia M.

6AE40555-5F4D-5DAE-9B0B-28AB9878D206 10.3897/BDJ.10.e87261.suppl36 Supplementary material 36 Distribution of *Troglohyphantesliburnicus* Caporiacco, 1927 Data type Distribution File: oo_615611.kml https://binary.pensoft.net/file/615611 Milano F., Borio L., Komposch C., Mammola S., Pantini P., Pavlek M., Isaia M.

731E3ED1-53FC-5718-8EB4-043983D40961 10.3897/BDJ.10.e87261.suppl37 Supplementary material 37 Distribution of *Troglohyphanteslucifer* Isaia, Mammola & Pantini, 2017 Data type Distribution File: oo_615612.kml https://binary.pensoft.net/file/615612 Milano F., Borio L., Komposch C., Mammola S., Pantini P., Pavlek M., Isaia M.

6A258585-6CF4-59AA-958A-5D1C124BF211 10.3897/BDJ.10.e87261.suppl38 Supplementary material 38 Distribution of *Troglohyphanteslucifuga* (Simon, 1884) Data type Distribution File: oo_615613.kml https://binary.pensoft.net/file/615613 Milano F., Borio L., Komposch C., Mammola S., Pantini P., Pavlek M., Isaia M.

11D8D056-B2C3-5777-A832-01D7BF80C278 10.3897/BDJ.10.e87261.suppl39 Supplementary material 39 Distribution of *Troglohyphantesmicrocymbium* Pesarini, 2001 Data type Distribution File: oo_615614.kml https://binary.pensoft.net/file/615614 Milano F., Borio L., Komposch C., Mammola S., Pantini P., Pavlek M., Isaia M.

FDE3FCEF-5864-56ED-8A3F-23D74CB4822F 10.3897/BDJ.10.e87261.suppl40 Supplementary material 40 Distribution of *Troglohyphantesnigraerosae* Brignoli, 1971 Data type Distribution File: oo_615616.kml https://binary.pensoft.net/file/615616 Milano F., Borio L., Komposch C., Mammola S., Pantini P., Pavlek M., Isaia M.

8A5B56DB-C1AE-535E-AA3F-0EDBDA6C29D6 10.3897/BDJ.10.e87261.suppl41 Supplementary material 41 Distribution of *Troglohyphantesnoricus* (Thaler & Polenec, 1974) Data type Distribution File: oo_615628.kml https://binary.pensoft.net/file/615628 Milano F., Borio L., Komposch C., Mammola S., Pantini P., Pavlek M., Isaia M.

C5C0E4B6-DAD9-5AC5-BFB7-9E9C8CECDBCC 10.3897/BDJ.10.e87261.suppl42 Supplementary material 42 Distribution of Troglohyphantes novicordis Thaler, 1978 Data type Distribution File: oo_615629.kml https://binary.pensoft.net/file/615629 Milano F., Borio L., Komposch C., Mammola S., Pantini P., Pavlek M., Isaia M.

051805F6-3CC4-5384-9F86-941D0034464D 10.3897/BDJ.10.e87261.suppl43 Supplementary material 43 Distribution of *Troglohyphantespavesii* Pesarini, 1988 Data type Distribution File: oo_615630.kml https://binary.pensoft.net/file/615630 Milano F., Borio L., Komposch C., Mammola S., Pantini P., Pavlek M., Isaia M.

789701D1-1DAA-577E-BB71-339E962AB714 10.3897/BDJ.10.e87261.suppl44 Supplementary material 44 Distribution of *Troglohyphantespedemontanus* (Gozo, 1908) Data type Distribution File: oo_615631.kml https://binary.pensoft.net/file/615631 Milano F., Borio L., Komposch C., Mammola S., Pantini P., Pavlek M., Isaia M.

521096E2-1D75-5608-A41C-EC4985BAA67F 10.3897/BDJ.10.e87261.suppl45 Supplementary material 45 Distribution of *Troglohyphantespluto* Caporiacco, 1938 Data type Distribution File: oo_615632.kml https://binary.pensoft.net/file/615632 Milano F., Borio L., Komposch C., Mammola S., Pantini P., Pavlek M., Isaia M.

BE6AF4F0-1732-5D5E-9EFA-B2E8C5599A2C 10.3897/BDJ.10.e87261.suppl46 Supplementary material 46 Distribution of *Troglohyphantespoleneci* Wiehle, 1964 Data type Distribution File: oo_617741.kml https://binary.pensoft.net/file/617741 Milano F., Borio L., Komposch C., Mammola S., Pantini P., Pavlek M., Isaia M.

E2B795AE-B1A6-589E-A368-1E1F533A6E6D 10.3897/BDJ.10.e87261.suppl47 Supplementary material 47 Distribution of *Troglohyphantespolyophthalmus* Joseph, 1882 Data type Distribution File: oo_615644.kml https://binary.pensoft.net/file/615644 Milano F., Borio L., Komposch C., Mammola S., Pantini P., Pavlek M., Isaia M.

60BE71AD-E30A-511C-948D-8305E4DED042 10.3897/BDJ.10.e87261.suppl48 Supplementary material 48 Distribution of *Troglohyphantesregalini* Pesarini, 1989 Data type Distribution File: oo_615646.kml https://binary.pensoft.net/file/615646 Milano F., Borio L., Komposch C., Mammola S., Pantini P., Pavlek M., Isaia M.

009C9BA2-7D53-5340-8156-B5CF1863954B 10.3897/BDJ.10.e87261.suppl49 Supplementary material 49 Distribution of *Troglohyphantesruffoi* Caporiacco, 1936 Data type Distribution File: oo_615647.kml https://binary.pensoft.net/file/615647 Milano F., Borio L., Komposch C., Mammola S., Pantini P., Pavlek M., Isaia M.

884DC604-500D-5F90-9BB5-465EA64E1E53 10.3897/BDJ.10.e87261.suppl50 Supplementary material 50 Distribution of *Troglohyphantessbordonii* Brignoli, 1975 Data type Distribution File: oo_615649.kml https://binary.pensoft.net/file/615649 Milano F., Borio L., Komposch C., Mammola S., Pantini P., Pavlek M., Isaia M.

697956FD-DE7E-5618-A42B-D8F10C5B5731 10.3897/BDJ.10.e87261.suppl51 Supplementary material 51 Distribution of *Troglohyphantessciakyi* Pesarini, 1989 Data type Distribution File: oo_615651.kml https://binary.pensoft.net/file/615651 Milano F., Borio L., Komposch C., Mammola S., Pantini P., Pavlek M., Isaia M.

AF995254-459D-59D3-9C71-A204AE5DE9A9 10.3897/BDJ.10.e87261.suppl52 Supplementary material 52 Distribution of *Troglohyphantesscientificus* Deeleman-Reinhold, 1978 Data type Distribution File: oo_615654.kml https://binary.pensoft.net/file/615654 Milano F., Borio L., Komposch C., Mammola S., Pantini P., Pavlek M., Isaia M.

B2208101-DD81-5E0F-8718-7CAB49EF821F 10.3897/BDJ.10.e87261.suppl53 Supplementary material 53 Distribution of *Troglohyphantessimilis* Fage, 1919 Data type Distribution File: oo_651141.kml https://binary.pensoft.net/file/651141 Milano F., Borio L., Komposch C., Mammola S., Pantini P., Pavlek M., Isaia M.

D6CC68DA-765A-57CC-B095-81F5D1D013CA 10.3897/BDJ.10.e87261.suppl54 Supplementary material 54 Distribution of *Troglohyphantessketi* Deeleman-Reinhold, 1978 Data type Distribution File: oo_615660.kml https://binary.pensoft.net/file/615660 Milano F., Borio L., Komposch C., Mammola S., Pantini P., Pavlek M., Isaia M.

40DD74FD-4DC4-5900-B4B8-DB131A28E391 10.3897/BDJ.10.e87261.suppl55 Supplementary material 55 Distribution of *Troglohyphantessordellii* (Pavesi, 1875) Data type Distribution File: oo_667111.kml https://binary.pensoft.net/file/667111 Milano F., Borio L., Komposch C., Mammola S., Pantini P., Pavlek M., Isaia M.

3FB00363-D41B-5D45-AAF5-9C65B36B3339 10.3897/BDJ.10.e87261.suppl56 Supplementary material 56 *Troglohyphantesspatulifer* Pesarini, 2001 Data type Distribution File: oo_632091.kml https://binary.pensoft.net/file/632091 Milano F., Borio L., Komposch C., Mammola S., Pantini P., Pavlek M., Isaia M.

6DB9194D-E684-54E1-9BD5-BFD5DAE5643B 10.3897/BDJ.10.e87261.suppl57 Supplementary material 57 Distribution of *Troglohyphantesspinipes* Fage, 1919 Data type Distribution File: oo_615664.kml https://binary.pensoft.net/file/615664 Milano F., Borio L., Komposch C., Mammola S., Pantini P., Pavlek M., Isaia M.

6F83DC78-7317-5FDD-BF10-E0032CEE18AD 10.3897/BDJ.10.e87261.suppl58 Supplementary material 58 Distribution of *Troglohyphantessubalpinus* Thaler, 1967 Data type Distribution File: oo_673013.kml https://binary.pensoft.net/file/673013 Milano F., Borio L., Komposch C., Mammola S., Pantini P., Pavlek M., Isaia M.

B86495BD-91E8-5057-90BF-E367FCEE2484 10.3897/BDJ.10.e87261.suppl59 Supplementary material 59 Distribution of *Troglohyphantestauriscus* Thaler, 1982 Data type Distribution File: oo_615666.kml https://binary.pensoft.net/file/615666 Milano F., Borio L., Komposch C., Mammola S., Pantini P., Pavlek M., Isaia M.

2A593394-96A5-5FDA-AA8D-42B02439A060 10.3897/BDJ.10.e87261.suppl60 Supplementary material 60 Distribution of *Troglohyphantesthaleri* Miller & Polenec, 1975 Data type Distribution File: oo_673046.kml https://binary.pensoft.net/file/673046 Milano F., Borio L., Komposch C., Mammola S., Pantini P., Pavlek M., Isaia M.

E60BBC1D-14D1-554A-9229-AF72D66C713C 10.3897/BDJ.10.e87261.suppl61 Supplementary material 61 Distribution of *Troglohyphantestrispinosus* Miller & Polenec, 1975 Data type Distribution File: oo_615670.kml https://binary.pensoft.net/file/615670 Milano F., Borio L., Komposch C., Mammola S., Pantini P., Pavlek M., Isaia M.

D0633679-3778-52F4-B16F-B9FDF9CF9AB7 10.3897/BDJ.10.e87261.suppl62 Supplementary material 62 Distribution of *Troglohyphantestyphlonetiformis* Absolon & Kratochvil, 1932 Data type Distribution File: oo_615672.kml https://binary.pensoft.net/file/615672 Milano F., Borio L., Komposch C., Mammola S., Pantini P., Pavlek M., Isaia M.

45E8C6D1-6BC2-5EDD-9BA9-C178B0865340 10.3897/BDJ.10.e87261.suppl63 Supplementary material 63 Distribution of *Troglohyphantesvicinus* Miller & Polenec, 1975 Data type Distribution File: oo_615675.kml https://binary.pensoft.net/file/615675 Milano F., Borio L., Komposch C., Mammola S., Pantini P., Pavlek M., Isaia M.

20204590-14BA-589A-87E1-78EF63D66049 10.3897/BDJ.10.e87261.suppl64 Supplementary material 64 Distribution of *Troglohyphantesvignai* Brignoli, 1971 Data type Distribution File: oo_615676.kml https://binary.pensoft.net/file/615676 Milano F., Borio L., Komposch C., Mammola S., Pantini P., Pavlek M., Isaia M.

03AE8F79-CE54-557C-B44B-B5EF4C0C662B 10.3897/BDJ.10.e87261.suppl65 Supplementary material 65 Distribution of *Troglohyphanteswiehlei* Miller & Polenec, 1975 Data type Distribution File: oo_615677.kml https://binary.pensoft.net/file/615677 Milano F., Borio L., Komposch C., Mammola S., Pantini P., Pavlek M., Isaia M.

F93C3918-7626-5E06-8E00-99737581DCA6 10.3897/BDJ.10.e87261.suppl66 Supplementary material 66 Distribution of *Troglohyphanteszanoni* Pesarini, 1988 Data type Distribution File: oo_667112.kml https://binary.pensoft.net/file/667112 Milano F., Borio L., Komposch C., Mammola S., Pantini P., Pavlek M., Isaia M.

B1A3A435-7AED-5DB0-949D-BE334BF8E108 10.3897/BDJ.10.e87261.suppl67 Supplementary material 67 Occurrence records of *Troglohyphantes* species considered in this work Data type Occurrences File: oo_684741.xlsx https://binary.pensoft.net/file/684741 Milano F., Borio L., Komposch C., Mammola S., Pantini P., Pavlek M., Isaia M.

## Figures and Tables

**Figure 17a.**
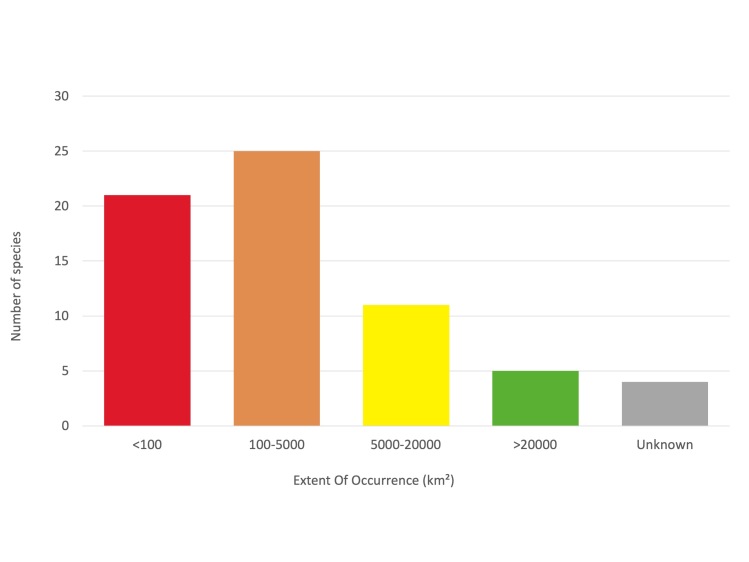


**Figure 17b.**
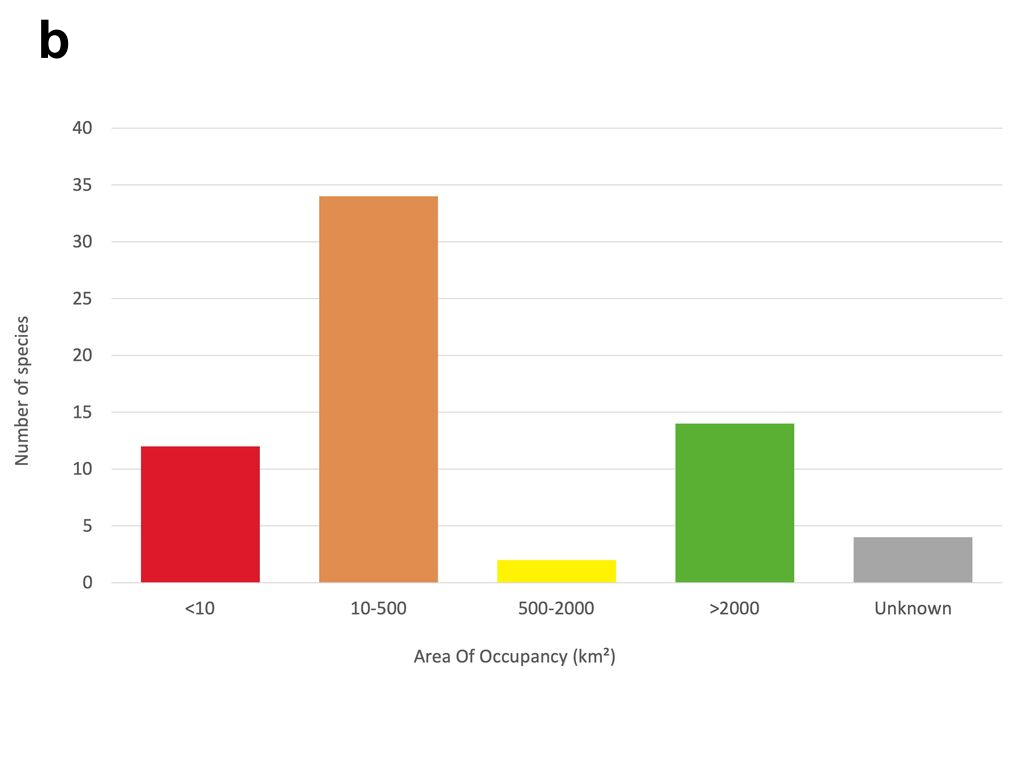


## References

[B7520397] Absolon K., Kratochvíl J. (1932). Zur Kenntnis der höhlenbewohnenden Araneae der illyrischen Karstgebiete.. Mitteilungen über Höhlen-und Karstforschung.

[B7490427] Badino Giovanni (2004). Cave temperatures and global climatic change. International Journal of Speleology.

[B7490883] Blick T., Scheidler M. (2004). Rote Liste gefährdeter Spinnen (Arachnida: Araneae) Bayerns. Schriftenreihe Bayerisches Landesamt für Umweltschutz.

[B7490868] Blick T., Finch O. D., Harms K. H., Kiechle J., Kielhorn K. H., Kreuels M., Malten A., Martin D., Muster C., Nährig D. (2016). Rote Liste und Gesamtartenliste der Spinnen (Arachnida: Araneae) Deutschlands. 3. Fassung, Stand: April 2008, einzelne Änderungen und Nachträge bis August 2015. Naturschutz und Biologische Vielfalt.

[B7861608] Bonte Dries, Baert Leon, Maelfait Jean-Pierre (2002). Spider assemblage structure and stability in a heterogeneous coastal dune system (Belgium). Journal of Arachnology.

[B7964629] Borges Paulo, Crespo Luis, Cardoso Pedro (2016). Species conservation profile of the cave spider Turinyphia cavernicola (Araneae, Linyphiidae) from Terceira Island, Azores, Portugal. Biodiversity Data Journal.

[B7964638] Borges Paulo, Lamelas-Lopez Lucas, Amorim Isabel, Danielczak Anja, Boieiro Mário, Rego Carla, Wallon Sophie, Nunes Rui, Cardoso Pedro, Hochkirch Axel (2019). Species conservation profiles of cave-dwelling arthropods from Azores, Portugal. Biodiversity Data Journal.

[B7636890] Branco Vasco Veiga, Cardoso Pedro (2020). An expert-based assessment of global threats and conservation measures for spiders. Global Ecology and Conservation.

[B7640181] Cardoso Pedro, Gaspar Clara, Pereira Luis C., Silva Israel, Henriques Sérgio S., da Silva Ricardo R., Sousa Pedro (2008). Assessing spider species richness and composition in Mediterranean cork oak forests. Acta Oecologica.

[B7490485] Cardoso Pedro, Erwin Terry L., Borges Paulo A. V., New Tim R. (2011). The seven impediments in invertebrate conservation and how to overcome them. Biological Conservation.

[B7111165] Cardoso Pedro (2017). red - an R package to facilitate species red list assessments according to the IUCN criteria. Biodiversity Data Journal.

[B7640193] Coddington Jonathan A., Levi Herbert W. (1991). Systematics and evolution of spiders (Araneae). Annual Review of Ecology and Systematics.

[B7640277] Coddington Jonathan A., Agnarsson Ingi, Miller Jeremy A., Kuntner Matjaž, Hormiga Gustavo (2009). Undersampling bias: the null hypothesis for singleton species in tropical arthropod surveys. Journal of Animal Ecology.

[B7899559] Condé Sophie, Richard Dominique, Liamine Nathalie (2005). Europe’s biodiversity–biogeographical regions and seas.

[B7633623] Culver D., Pipan T. (2019). The biology of caves and other subterranean habitats.

[B7273430] Deeleman-Reinhold C L (1978). Revision of the cave-dwelling and related spiders of the genus *Troglohyphantes* Joseph (Linyphiidae) with special reference to the Yugoslav species.

[B7527928] Deltshev C. (2008). Faunistic diversity and zoogeography of cave-dwelling spiders on the Balkan Peninsula.. Advances in Arachnology and Developmental Biology.

[B7490444] Domínguez-Villar David, Lojen Sonja, Krklec Kristina, Baker Andy, Fairchild Ian J. (2014). Is global warming affecting cave temperatures? Experimental and model data from a paradigmatic case study. Climate Dynamics.

[B7640341] Dunlop J. A., Penney D., Jekel D. A summary list of fossil spiders and their relatives. In: World Spider Catalog. Natural History Museum Bern. http://wsc.nmbe.ch,version20.5.

[B7640439] Fage L. (1919). Biospelogica XL. Etudes sur les araignées cavernicoles. III. Sur le genre *Troglohyphantes*. Archives de Zoologie Expérimentale et Générale.

[B7869515] Fernandez-Cortes Angel, Cuezva Soledad, Sanchez-Moral Sergio, Cañaveras Juan Carlos, Porca Estefania, Jurado Valme, Martin-Sanchez Pedro Maria, Saiz-Jimenez Cesareo (2011). Detection of human-induced environmental disturbances in a show cave. Environmental Science and Pollution Research.

[B7195185] Fick Stephen E., Hijmans Robert J. (2017). WorldClim 2: new 1‐km spatial resolution climate surfaces for global land areas. International Journal of Climatology.

[B7640376] Foelix Rainer F. (2011). Biology of Spiders.

[B7640384] Garrison Nicole L., Rodriguez Juanita, Agnarsson Ingi, Coddington Jonathan A., Griswold Charles E., Hamilton Christopher A., Hedin Marshal, Kocot Kevin M., Ledford Joel M., Bond Jason E. (2016). Spider phylogenomics: untangling the Spider Tree of Life. PeerJ.

[B7570784] Gasparo F. (2000). Note sinonimiche e corologiche su due specie del genere *Troglohyphantes* Joseph, 1881, delle Alpi orientali (Araneae, Linyphiidae).. Gortania - Atti del Museo Friulano di Storia Naturale.

[B7640173] Hijmans R. J., Phillips S., Leathwick J., Elith J. (2021). dismo: Species distribution modeling. https://rspatial.org/raster/sdm/.

[B7195039] Isaia Marco, Pantini Paolo (2010). New data on the spider genus *Troglohyphantes* (Araneae, Linyphiidae) in the Italian Alps, with the description of a new species and a new synonymy. Zootaxa.

[B7490977] Isaia Marco, Lana Enrico, Pantini Paolo (2010). Ecology and distribution of the genus *Troglohyphantes* Joseph, 1881 in the Western Italian Alps.

[B7570270] Isaia M., Paschetta M., Lana E., Pantini P., Schönhofer A. L., Christian E., Badino G. (2011). Aracnidi sotterranei delle Alpi Occidentali italiane (Arachnida: Araneae, Opiliones, Palpigradi, Pseudoscorpiones)/Subterranean Arachnids of the Western Italian Alps (Arachnida: Araneae, Opiliones, Palpigradi, Pseudoscorpiones)..

[B7195011] Isaia Marco, Mammola Stefano, Mazzuca Paola, Arnedo Miquel A., Pantini Paolo (2017). Advances in the systematics of the spider genus *Troglohyphantes* (Araneae, Linyphiidae). Systematics and Biodiversity.

[B7861681] Isaia Marco, Arnedo Miquel A., Mammola Stefano (2022). A multi-layered approach uncovers overlooked taxonomic and physiological diversity in Alpine subterranean spiders (Araneae: Linyphiidae: *Troglohyphantes*). Invertebrate Systematics.

[B7861560] IUCN (2001). IUCN Red List categories and criteria: Version 3.1.

[B7861568] IUCN (2012). IUCN Red List categories and criteria: Version 3.1. Second edition.

[B7570299] Centre IUCN Conservation Monitoring (1986). 1986 IUCN Red List of threatened animals.. Cambridge, UK.

[B7273303] Committee IUCN Standards and Petitions (2019). Guidelines for using the IUCN Red List categories and criteria. Version 14. http://www.iucnredlist.org/documents/RedListGuidelines.pdf.

[B7640399] Jocqué R., Alderweireldt M., Dippenaar-Schoeman A., Penney D. (2013). Spider research in the 21st Century: trends and perspective.

[B7574508] Komposch C., Steinberger K. H., Holzinger W. E., Mildner P., Rottenburg T., Wieser C. (1999). Rote Listen gefährdeter Tiere Kärntens.

[B7646159] Komposch C., Rabitsch W., Essl F. (2009). Endemiten. Kostbarkeiten in Österreichs Tier- und Pflanzenwelt.

[B7861576] Lowe Elizabeth C., Wolff Jonas O., Aceves-Aparicio Alfonso, Birkhofer Klaus, Branco Vasco Veiga, Cardoso Pedro, Chichorro Filipe, Fukushima Caroline Sayuri, Gonçalves-Souza Thiago, Haddad Charles R., Isaia Marco, Krehenwinkel Henrik, Audisio Tracy Lynn, Macías-Hernández Nuria, Malumbres-Olarte Jagoba, Mammola Stefano, McLean Donald James, Michalko Radek, Nentwig Wolfgang, Pekár Stano, Pétillon Julien, Privet Kaïna, Scott Catherine, Uhl Gabriele, Urbano-Tenorio Fernando, Wong Boon Hui, Herberstein Marie E. (2020). Towards establishment of a centralized spider traits database. The Journal of Arachnology.

[B7502119] Mammola Stefano, Isaia Marco, Arnedo Miquel A. (2015). Alpine endemic spiders shed light on the origin and evolution of subterranean species. PeerJ.

[B7603484] Mammola Stefano, Isaia Marco (2016). The ecological niche of a specialized subterranean spider. Invertebrate Biology.

[B7884508] Mammola Stefano, Giachino Pier Mauro, Piano Elena, Jones Alexandra, Barberis Marcel, Badino Giovanni, Isaia Marco (2016). Ecology and sampling techniques of an understudied subterranean habitat: the Milieu Souterrain Superficiel (MSS).. The Science of Nature.

[B7490364] Mammola Stefano, Isaia Marco (2017). Spiders in caves. Proceedings of the Royal Society B: Biological Sciences.

[B7635989] Mammola Stefano, Leroy Boris (2017). Applying species distribution models to caves and other subterranean habitats. Ecography.

[B7640412] Mammola Stefano, Michalik Peter, Hebets Eileen A., Isaia Marco (2017). Record breaking achievements by spiders and the scientists who study them. PeerJ.

[B7502183] Mammola Stefano, Piano Elena, Giachino Pier Mauro, Isaia Marco (2017). An ecological survey of the invertebrate community at the epigean/hypogean interface. Subterranean Biology.

[B7527657] Mammola Stefano, Goodacre Sara L., Isaia Marco (2018). Climate change may drive cave spiders to extinction. Ecography.

[B7527737] Mammola Stefano, Cardoso Pedro, Ribera Carles, Pavlek Martina, Isaia Marco (2018). A synthesis on cave-dwelling spiders in Europe. Journal of Zoological Systematics and Evolutionary Research.

[B7527621] Mammola Stefano, Arnedo Miquel A., Pantini Paolo, Piano Elena, Chiappetta Nicolò, Isaia Marco (2018). Ecological speciation in darkness? Spatial niche partitioning in sibling subterranean spiders (Araneae: Linyphiidae: *Troglohyphantes*). Invertebrate Systematics.

[B7964542] Mammola Stefano, Milano Filippo, Vignal Matthieu, Andrieu Julien, Isaia Marco (2019). Associations between habitat quality, body size and reproductive fitness in the alpine endemic spider<i>Vesubiajugorum</i>. Global Ecology and Biogeography.

[B7195203] Mammola Stefano, Piano Elena, Malard Florian, Vernon Philippe, Isaia Marco (2019). Extending Janzen’s hypothesis to temperate regions: a test using subterranean ecosystems. Functional Ecology.

[B7195101] Mammola Stefano, Piano Elena, Cardoso Pedro, Vernon Philippe, Domínguez-Villar David, Culver David C, Pipan Tanja, Isaia Marco (2019). Climate change going deep: the effects of global climatic alterations on cave ecosystems. The Anthropocene Review.

[B7603412] Mammola Stefano, Cardoso Pedro, Culver David C, Deharveng Louis, Ferreira Rodrigo L, Fišer Cene, Galassi Diana M P, Griebler Christian, Halse Stuart, Humphreys William F, Isaia Marco, Malard Florian, Martinez Alejandro, Moldovan Oana T, Niemiller Matthew L, Pavlek Martina, Reboleira Ana Sofia P S, Souza-Silva Marconi, Teeling Emma C, Wynne J Judson, Zagmajster Maja (2019). Scientists' warning on the conservation of subterranean ecosystems. BioScience.

[B7527562] Mammola Stefano, Cardoso Pedro, Angyal Dorottya, Balázs Gergely, Blick Theo, Brustel Hervé, Carter Julian, Ćurčić Srećko, Danflous Samuel, Dányi László, Déjean Sylvain, Deltshev Christo, Elverici Mert, Fernández Jon, Gasparo Fulvio, Komnenov Marjan, Komposch Christian, Kováč L’ubomír, Kunt Kadir, Mock Andrej, Moldovan Oana, Naumova Maria, Pavlek Martina, Prieto Carlos, Ribera Carles, Rozwałka Robert, Růžička Vlastimil, Vargovitsh Robert, Zaenker Stefan, Isaia Marco (2019). Continental data on cave-dwelling spider communities across Europe (Arachnida: Araneae). Biodiversity Data Journal.

[B7603391] Mammola Stefano, Arnedo Miquel A., Fišer Cene, Cardoso Pedro, Dejanaz Andrea J., Isaia Marco (2020). Environmental filtering and convergent evolution determine the ecological specialization of subterranean spiders. Functional Ecology.

[B7869528] Mammola Stefano, Pavlek Martina, Huber Bernhard A., Isaia Marco, Ballarin Francesco, Tolve Marco, Čupić Iva, Hesselberg Thomas, Lunghi Enrico, Mouron Samuel, Graco-Roza Caio, Cardoso Pedro (2022). A trait database and updated checklist for European subterranean spiders. Scientific data.

[B7816230] Mammola Stefano, Meierhofer Melissa B., Borges Paulo A. V., Colado Raquel, Culver David C., Deharveng Louis, Delić Teo, Di Lorenzo Tiziana, Dražina Tvrtko, Ferreira Rodrigo L., Fiasca Barbara, Fišer Cene, Galassi Diana M. P., Garzoli Laura, Gerovasileiou Vasilis, Griebler Christian, Halse Stuart, Howarth Francis G., Isaia Marco, Johnson Joseph S., Komerički Ana, Martínez Alejandro, Milano Filippo, Moldovan Oana T., Nanni Veronica, Nicolosi Giuseppe, Niemiller Matthew L., Pallarés Susana, Pavlek Martina, Piano Elena, Pipan Tanja, Sanchez‐Fernandez David, Santangeli Andrea, Schmidt Susanne I., Wynne J. Judson, Zagmajster Maja, Zakšek Valerija, Cardoso Pedro (2022). Towards evidence‐based conservation of subterranean ecosystems. Biological Reviews.

[B7550848] Marazzi S. (2005). Atlante orografico delle Alpi. SOIUSA..

[B7490506] Merow Cory, Smith Matthew J., Silander John A. (2013). A practical guide to MaxEnt for modeling species' distributions: what it does, and why inputs and settings matter. Ecography.

[B7636866] Milano Filippo, Blick Theo, Cardoso Pedro, Chatzaki Maria, Fukushima Caroline Sayuri, Gajdoš Peter, Gibbons Alastair T., Henriques Sergio, Macías-Hernández Nuria, Mammola Stefano, Nentwig Wolfgang, Nolan Myles, Pétillon Julien, Polchaninova Nina, Řezáč Milan, Sandström Jonas, Smith Helen, Wiśniewski Konrad, Isaia Marco (2021). Spider conservation in Europe: a review. Biological Conservation.

[B7964533] Milano Filippo, Tolve Marco, Isaia Marco (in press). Natural history and conservation of the wolf spider *Vesubiajugorum* (Araneae: Lycosidae), assessed as Endangered in the the IUCN Red List. Zoosystema.

[B7635841] Nagy L., Thompson D., Grabherr G., Körner C. (2003). Alpine biodiversity in Europe: an introduction.

[B7640421] Nentwig W. (2013). Spider ecophysiology.

[B7494533] Nentwig Wolfgang, Blick Theo, Bosmans Robert, Gloor Daniel, Hänggi Ambros, Kropf Christian Spiders of Europe. Version 04.2022. https://www.araneae.nmbe.ch.

[B7574111] Ozimec R., Bedek J., Gottstein S., Jalžić B., Slapnik R., Štamol V., Bilandžija H., Dražina T., Kletečki E., Komerički A., Lukić M., Pavlek M. (2009). Crvena knjiga špiljske faune Hrvatske.

[B7195057] Pantini P, Isaia M (2019). Araneae.it: the online Catalog of Italian spiders with addenda on other Arachnid Orders occurring in Italy (Arachnida: Araneae, Opiliones, Palpigradi, Pseudoscorpionida, Scorpiones, Solifugae).. https://www.araneae.it/.

[B7603380] Parimuchová Andrea, Dušátková Lenka Petráková, Kováč Ľubomír, Macháčková Táňa, Slabý Ondřej, Pekár Stano (2021). The food web in a subterranean ecosystem is driven by intraguild predation. Scientific Reports.

[B7273421] Pavlek Martina, Ozimec Roman (2009). New cave-dwelling species of genus *Troglohyphantes* (Araneae, Linyphiidae) for Croatian fauna. Natura Croatica.

[B7741993] Pavlek M., Gauthier J., Tonzo V., Bilat J., Arnedo M., Alvarez N. (in press). Life-history traits drive spatial genetic structuring in Dinaric cave spiders. (Submitted).

[B7550798] Pekár Stano, Wolff Jonas O, Černecká Ľudmila, Birkhofer Klaus, Mammola Stefano, Lowe Elizabeth C, Fukushima Caroline S, Herberstein Marie E, Kučera Adam, Buzatto Bruno A, Djoudi El Aziz, Domenech Marc, Enciso Alison Vanesa, Piñanez Espejo Yolanda M G, Febles Sara, García Luis F, Gonçalves-Souza Thiago, Isaia Marco, Lafage Denis, Líznarová Eva, Macías-Hernández Nuria, Magalhães Ivan, Malumbres-Olarte Jagoba, Michálek Ondřej, Michalik Peter, Michalko Radek, Milano Filippo, Munévar Ana, Nentwig Wolfgang, Nicolosi Giuseppe, Painting Christina J, Pétillon Julien, Piano Elena, Privet Kaïna, Ramírez Martín J, Ramos Cândida, Řezáč Milan, Ridel Aurélien, Růžička Vlastimil, Santos Irene, Sentenská Lenka, Walker Leilani, Wierucka Kaja, Zurita Gustavo Andres, Cardoso Pedro (2021). The World Spider Trait database: a centralized global open repository for curated data on spider traits. Database.

[B7273344] Pesarini Carlo (2001). Note sui *Troglohyphantes* italiani, con descrizione di quattro nuove specie (Araneae
Linyphiidae). Atti della Società italiana di scienze naturali e del Museo civico di storia naturale di Milano.

[B7490515] Peterson A. Townsend, Soberón Jorge, Pearson Richard G., Anderson Robert P., Martínez-Meyer Enrique, Nakamura Miguel (2011). Ecological niches and geographic distributions (MPB-49).

[B7633693] Team R Core (2021). R: A language and environment for statistical computing. https://www.R-project.org/.

[B7603402] Sánchez-Fernández David, Galassi Diana M. P., Wynne J. Judson, Cardoso Pedro, Mammola Stefano (2021). Don’t forget subterranean ecosystems in climate change agendas. Nature Climate Change.

[B7490525] Saupe E. E., Barve V., Myers C. E., Soberón J., Barve N., Hensz C. M., Peterson A. T., Owens H. L., Lira-Noriega A. (2012). Variation in niche and distribution model performance: The need for a priori assessment of key causal factors. Ecological Modelling.

[B7861617] Scott Alan G., Oxford Geoff S., Selden Paul A. (2006). Epigeic spiders as ecological indicators of conservation value for peat bogs. Biological Conservation.

[B7520364] Thaler K. (1999). *Troglohyphantestyphlonetiformis* Absolon et Kratochvil-neu für Österreich (Arachnida, Araneae, Linyphiidae).. Carinthia II.

[B7640430] Turnbull A. L. (1973). Ecology of the true spiders (Araneomorphae). Annual Review of Entomology.

[B7490593] RS Uradni list (št. 82/02 in 42/10). Pravilnik o uvrstitvi ogroženih rastlinskih in živalskih vrst v rdeči seznam. https://www.uradni-list.si/#.

[B7574386] Centre World Conservation Monitoring (1996). Troglohyphantesgracilis. IUCN Red List of Threatened Species.

[B7574377] Centre World Conservation Monitoring (1996). Troglohyphantessimilis. IUCN Red List of Threatened Species.

[B7574359] Centre World Conservation Monitoring (1996). Troglohyphantesspinipes. IUCN Red List of Threatened Species.

[B7570944] Catalog World Spider World Spider Catalogue Version 23.0. Natural History Museum Bern. http://wsc.nmbe.ch.

